# Chemistry of Materials Underpinning Photoelectrochemical Solar Fuel Production

**DOI:** 10.1021/acs.chemrev.4c00258

**Published:** 2025-05-06

**Authors:** Zebulon G. Schichtl, O. Quinn Carvalho, Jeiwan Tan, Simran S. Saund, Debjit Ghoshal, Logan M. Wilder, Melissa K. Gish, Adam C. Nielander, Michaela Burke Stevens, Ann L. Greenaway

**Affiliations:** † Materials Chemical and Computational Science Directorate, 53405 National Renewable Energy Laboratory, Golden, Colorado 80401, United States; ‡ SUNCAT Center for Interface Science and Catalysis, 497525 SLAC National Accelerator Laboratory, 2575 Sand Hill Road, Menlo Park, California 94025, United States

## Abstract

Since its inception, photoelectrochemistry has sought to power the generation of fuels, particularly hydrogen, using energy from sunlight. Efficient and durable photoelectrodes, however, remain elusive. Here we review the current state of the art, focusing our discussion on advances in photoelectrodes made in the past decade. We open by briefly discussing fundamental photoelectrochemical concepts and implications for photoelectrode function. We next review a broad range of semiconductor photoelectrodes broken down by material class (oxides, nitrides, chalcogenides, and mature photovoltaic semiconductors), identifying intrinsic properties and discussing their influence on performance. We then identify innovative in situ and operando techniques to directly probe the photoelectrode|electrolyte interface, enabling direct assessment of structure–property relationships for catalytic surfaces in active reaction environments. We close by considering more complex photoelectrochemical fuel-forming reactions (carbon dioxide and nitrogen reduction, as well as alternative oxidation reactions), where product selectivity imposes additional criteria on electrochemical driving force and photoelectrode architecture. By contextualizing recent literature within a fundamental framework, we seek to provide direction for continued progress toward achieving efficient and stable fuel-forming photoelectrodes.

## Graphical abstract

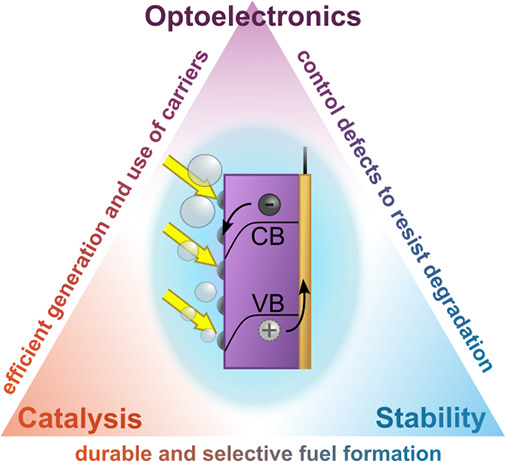

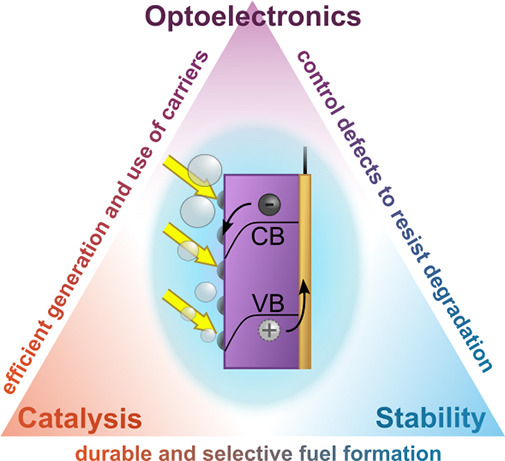

## 1 Introduction

Electricity generation from abundant but intermittent sources such as wind and solar increases every year,^1^ but there is not yet a clear path to renewable fuel production,^2–4^ which is important for both long-term renewable energy storage and integration of renewables in existing energy infrastructure.^5,6^ Photoelectrochemistry is a direct route to generate renewable fuels by capturing solar energy and storing it indefinitely as stable chemical bonds.^7^ Early demonstrations of photoelectrochemical (PEC) H_2_ generation via water splitting date back 50 years,^8,9^ but in many ways that seminal work was ahead of its time, occurring when the science underlying photovoltaic (PV) energy conversion and electrolysis was still nascent. With the rapid development of these fields over the last half-century, there is now a unique opportunity to carefully analyze and overcome the scientific challenges to PEC fuel generation to advance toward deployment of this technology.^6,10^

In its simplest form, a PEC fuel-forming system has only a few components (Figure 1a): a photoelectrode that converts solar photons to energetic charge carriers, in contact with an electrolyte containing the appropriate chemical precursors, and a counter electrode that balances charge passed at the photoelectrode by driving a complementary half-reaction. In principle, the simplest photoelectrode comprises a single semiconductor able to absorb enough of the solar spectrum, based on its band gap and absorption coefficient, to generate sufficient electrochemical potential difference to drive current toward a fuel-forming reaction at a sufficient rate.^10,11^ Such an ideal photoelectrode would be photoactive (efficiently converting above-gap photons to long-lived charge carriers), catalytic (driving fuel-forming reactions with little energy loss), and stable in electrolyte, both under illumination and in the dark. In practice, these properties are not found in a single material but can be assembled from multiple inorganic semiconductors and metals, where the sum of the photoelectrode properties enables PEC fuel formation. However, design of architectures combining *optoelectronics* (band gap, band edge positions, charge carrier transport), *catalysis* (maximized use of kinetic energy, product selectivity), and chemical *stability* (photoelectrode operating lifetime) necessary for sustained PEC fuel generation has proven a considerable challenge (Figure 1b).^5,12^

**Figure 1:**
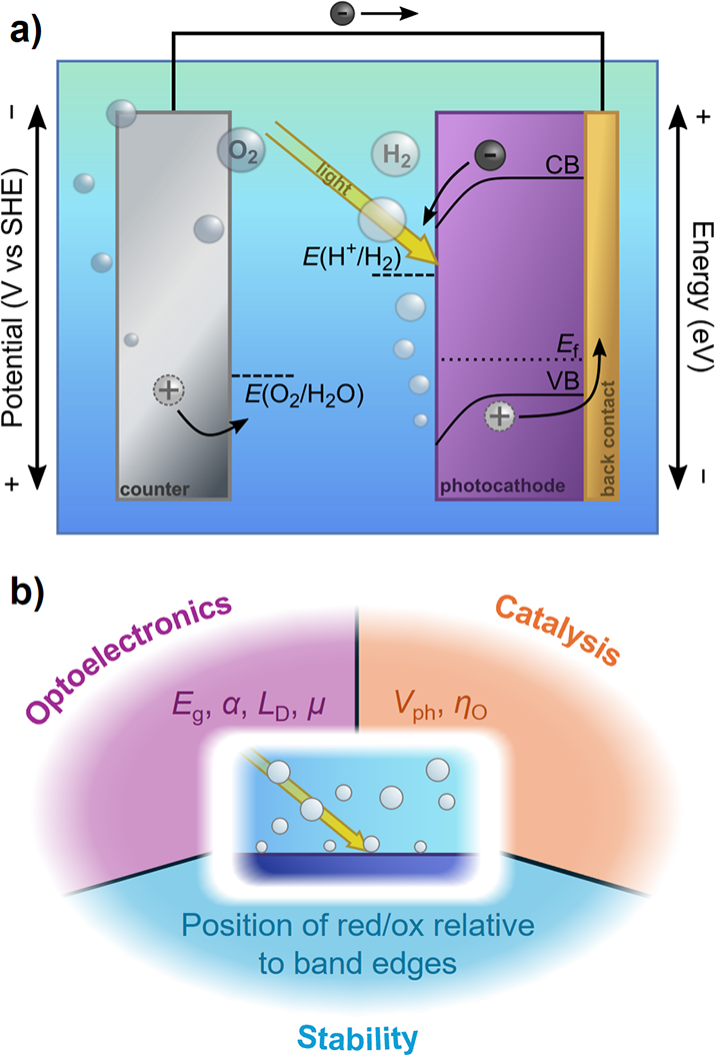
(a) The simplest formulation of a PEC water-splitting system, using a single, wide band gap semiconductor photocathode and a metallic counter electrode. Light is absorbed in the semiconductor, exciting an electron to the conduction band (CB) where it then is used to drive the hydrogen evolution reaction (HER) at the photoelectrode surface. Photocurrent provides holes to the counter electrode that are sufficiently energetic to drive the oxygen evolution reaction (OER). b) Schematic illustration of the competing design criteria around which photoelectrodes must be designed: optoelectronics (band gap, *E*_g_; absorptivity, α; carrier diffusion length, *L*_D_; and carrier mobility, μ), catalysis (photovoltage, *V*_ph_; and overpotential, η_O_), and stability (position of fuel-forming reactions and deleterious surface reactions relative to the band edge positions of the semiconductor).

Disentangling how various processes (photoabsorption and charge transport, catalysis, stability) in a photoelectrode dictate the observed performance is critical to developing rational design strategies for improving that performance. Optoelectronic properties determine wavelength-dependent absorption and charge transport, dictating the likelihood a photogenerated charge carrier reaches the electrode|electrolyte interface where catalysis can occur. Catalytic properties determine potential energy barriers for reactions, dictating reaction efficiency by tuning rates and selectivity. Stability influences photoelectrode operating lifetimes, bearing further implications for catalytic rates, and product selectivity. While ex situ characterization methods can link properties and behaviors, they do not always accurately capture photoelectrode behavior under operating conditions. The development of in situ and operando experimental techniques has helped to address the “disentanglement” challenge, enabling correlation of changes in macroscopic and atomic scale structure, oxidation state, composition, morphology, etc. with PEC performance.^13,14^ In turn, such measurements have guided and will continue to enable the development of new semiconductors, protective schemes, and catalysts in photoelectrodes.

Efficient PEC water splitting requires optimization of both the hydrogen and oxygen evolution reactions (HER and OER, respectively), from control over catalysis^15^ to ensuring photoelectrode stability in corrosive electrolyte.^16^ Although H_2_ remains the primary target of PEC fuel production research, there are now substantial efforts dedicated to PEC carbon dioxide reduction (CO_2_RR)^17^ and to generating ammonia by dinitrogen or nitrate reduction (N_2_RR or NO_3_^–^RR).^18^ Given the sluggish kinetics and poor economic incentive for OER, interest is increasing in alternative oxidation reactions capable of generating other chemical products, often with lower equilibrium potentials or lower kinetic barriers.^19–21^ As the number of targeted fuels to be synthesized by PEC methods expands, so too does the range of semiconductor photoelectrodes which can potentially be used in such systems, as the demands on optoelectronics, catalysis and stability change.

The fundamentals of photoelectrochemistry and the challenges facing the field have been detailed in many excellent reviews;^22–28^ here, we specifically intend to ground the researcher in the chemistry and progress of inorganic semiconductor photoelectrodes for PEC. Faced with a dizzying array of candidate photoelectrodes and device configurations, entangled performance and stability metrics, and investigations into ever more reactions, it can be difficult to even start understanding the status of the PEC field. The remainder of the introduction provides a brief overview of the fundamental processes of inorganic semiconductor photoelectrodes for PEC. We then turn to recent innovations on photoelectrode chemistry and design. Grounded in that context, we discuss state-of-the-art in situ and operando characterization techniques that disentangle the many overlapping structure–property relationships undergirding device performance. Following discussion of advances in PEC reactions beyond HER and OER, we close by highlighting remaining challenges and promising future directions in the chemistry of photoelectrodes.

### 1.1 Fundamentals Dictating Efficiency of PEC Fuel Formation

While electrochemical fuel-forming reactions can be powered by directly applying a voltage between two electrodes, the power required to perform these reactions must be externally provided. PEC fuel-forming reactions can in principle be performed without an externally applied bias, directly capturing and converting photons into electrochemical potential difference to drive electrochemical reactions. Here, a photoelectrode must supply enough photovoltage (*V*_ph_, the potential difference between the photoelectrode|solution contact and the photoelectrode back contact) from captured photons to overcome the thermodynamic minimum cell potential, *E* ^0^, for both half-reactions. Note that in cases where fast redox couples are present, *V*_ph_ can approach the open-circuit potential, *V*_OC_.^29^ For so-called “water splitting”, a whole-cell thermodynamic potential of 1.23 V is required to drive both the cathodic HER and anodic OER:^30^

$$2H^{+}+2e^{-}⇌H_{2}\;E^{0}=0\;V_{\mathrm{SHE}}$$ 1

$$2H_{2}O⇌O_{2}+4e^{-}+4H^{+}\;E^{0}=1.23\;V_{\mathrm{SHE}}$$ 2

Under the standard condition of 1 M strong acid (pH 0) in aqueous electrolytes at 25 °C, the equilibrium between protons and hydrogen molecules (eq 1) is defined as the *standard hydrogen electrode* (SHE). While eqs 1 and 2 are written as performed in acidic environments, sourcing protons from hydronium ions (H_3_O^+^ for HER, shown as protons in equation) or water (H_2_O for OER), the same reactions can be considered in alkaline electrolytes,

$$2H_{2}O+2e^{-}⇌H_{2}+2OH^{-}\;E^{0}=-0.828\;V_{\mathrm{SHE}}$$ 3

$$4OH^{-}⇌O_{2}+2H_{2}O+4e^{-}\;E^{0}=0.402\;V_{\mathrm{SHE}}$$ 4

with either water (for HER) or hydroxide anions (OH^–^ for OER) serving as proton donors. Differences in the *E* ^0^ between the HER and OER performed in acidic (eqs 1 and 2) and alkaline electrolytes (eqs 3 and 4) are due to the Nernstian dependence of *E* ^0^ on proton activity, ∼59 mV per pH unit.^31^ To address the pH-dependence of *E* ^0^ on electrochemical kinetics, the (photo)­electrochemical community instead frequently refers to the *reversible hydrogen electrode* (RHE), which assumes a reference potential of 0 V_RHE_ for the equilibrium between protons and molecular hydrogen at any pH.

Whether illuminated or not (“dark” electrocatalysis), HER is often benchmarked in acidic electrolytes,^32^ where abundant hydronium ions serve as more facile proton donors than water, improving reaction kinetics.^33,34^ In contrast, alkaline electrolytes are chosen for OER, where (photo)­electrodes are generally more stable;^35^ OER kinetics are nominally comparable in acidic and alkaline electrolytes.^36^ However, achieving overall water splitting requires operation of both electrodes in a connected system, either at the same pH or with some method to stably maintain a pH gradient. Put simply, the thermodynamic requirement of *E* ^0^ cannot be avoided: at least 1.23 V will be required to drive both hydrogen and oxygen evolution in any (photo)­electrochemical cell. Conducting both reactions at the same pH frequently leads to additional overpotentials on at least one (photo)­electrode, or on both if operating in near-neutral conditions. This is also true for systems that can passively maintain pH gradients between anode and cathode: appropriately designed bipolar membrane-based systems can maintain alkaline conditions for OER and acidic conditions for HER indefinitely. This does not lead to a lower cell voltage requirement due to the energetic requirement to drive water dissociation (H_2_O → H^+^ + OH^–^) at the bipolar membrane.^37^ Chemical biases (e.g., pH gradients across a cell that can spontaneously neutralize)^38^ can be used to reduce the electric bias required to drive a reaction but should be viewed as an additional energy input to the system.

The solar-to-fuel efficiency (η_STF_) of a photoelectrode can be calculated simply for band gap sizes above the minimum cell potential for a given reaction:^39^

$$\eta_{\mathrm{STF}}=\frac{j_{\mathrm{SC}}E^{0}\eta_{F}}{P_{\mathrm{total}}}$$ 5

where *j*_SC_ is the short-circuit photocurrent density, η_F_ is the Faradaic efficiency (FE) of the system (the fraction of current that goes toward fuel generation), and *P*_total_ is the input power density from illumination. This calculation can be performed both for theoretical and demonstrated photoelectrodes, resulting in the frequent comparison of realized performance against η_STF_, and implicitly captures the role of optoelectronic properties in PEC efficiency. A single semiconductor must have either a wide enough band gap to generate the needed *V*_ph_ (as in Figure 1) or be coupled with another semiconductor to provide the additional driving force. While wide band gap semiconductors can provide sufficiently energetic charge carriers necessary to perform both half-reactions, they only absorb the most energetic portion of the solar spectrum and have correspondingly poor theoretical *j*_SC_ (e.g., TiO_2_ and BiVO_4_ in Figure 2a). Conversely, narrowing the band gap utilizes more of the solar spectrum and increases *j*_SC_, but decreases the potential of photogenerated charge carriers (e.g., Si in Figure 2a). Thus, while some semiconductors have sufficiently wide band gaps to drive both half-reactions for fuel formation, some with narrower band gaps are routinely investigated to drive only one half-reaction, assuming that a complementary photoelectrode will be found to drive the coupled oxidation or reduction process.

**Figure 2:**
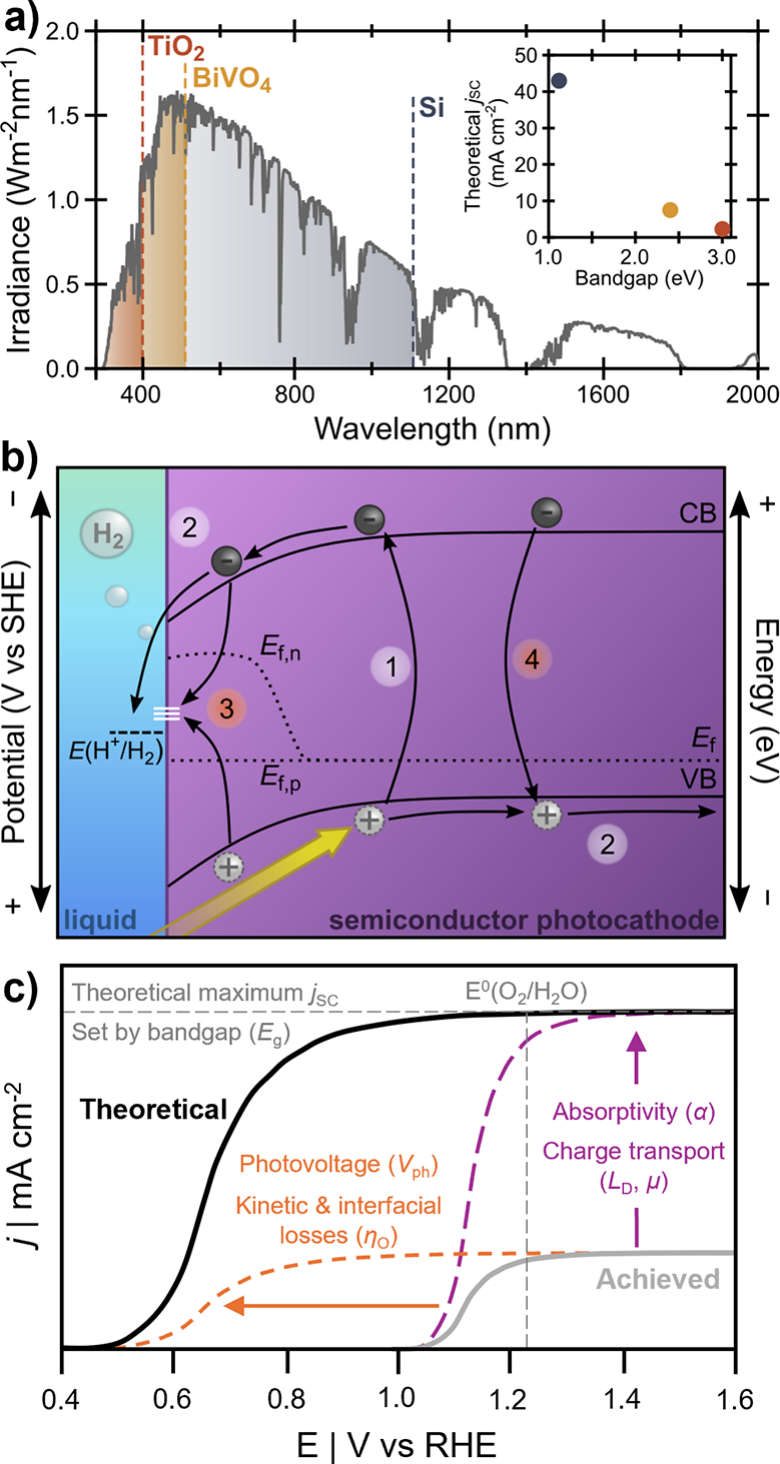
(a) Irradiance (air mass 1.5 global, AM 1.5G)^40^ with overlaid band gaps of three prototypical semiconductors, with the maximum theoretical *j*_SC_ of those semiconductors inset. (b) Favorable (white) and unfavorable (red) processes in a semiconductor photocathode. (1) Light absorption excites an electron to the conduction band (CB), leaving behind a corresponding hole in the valence band (VB). (2) The electron diffuses to the semiconductor|electrolyte interface to drive HER while the hole diffuses to the back contact where it is collected. (3) Photogenerated carriers are lost to recombination at surface states (white lines), which may be caused by electronic traps or diversion to nonproductive redox reactions such as corrosion. (4) Carriers recombine radiatively or nonradiatively in the bulk of the semiconductor. (c) Illustration of the influence of different design strategies on a hypothetical photoanode, where tuning optoelectronic properties (purple) increases the fraction of utilized solar photons toward theoretical maximum *j*_SC_, and tuning catalytic properties (orange) enhances electrochemical driving force or decreases kinetic barriers.

Even if the band gap is wide enough to drive both oxidation and reduction half-reactions, the positions of a semiconductor’s valence and conduction band edges constrain the application of charge carriers to a PEC reaction.^15,41^ Consider a photocathode driving a reduction half-reaction (Figure 2b), which must have a conduction band edge with a sufficiently negative electrochemical potential to drive the reaction with any necessary overpotential (discussed in more detail below); more specifically, under steady-state illumination, the electron quasi-Fermi level (*E*_fn_) must be positioned sufficiently negative to generate a photovoltage that meets the thermodynamic requirement. In an ideal photocathode, (1) photoexcited charge carriers separate and (2) electrons are transported toward a catalytically active surface to drive a reduction reaction, while holes migrate in the opposite direction to drive an oxidation reaction. Deleterious (3) interfacial and (4) bulk recombination processes consume photogenerated charge carriers, reducing *j*_SC_ and *V*_OC_ (and ultimately η_STF_) by decreasing the population of charge carriers available to drive the fuel-forming reactions.^22,42^ A photoanode driving an oxidation half-reaction is subject to similar requirements that the valence band edge and hole quasi-Fermi level (*E*_fp_) be sufficiently positive of the thermodynamic minimum for an oxidation half-reaction. Photoelectrode Fermi levels must encompass not only the thermodynamic potential of their corresponding half-reactions, but also the kinetic potential energy barrier for those reactions (discussed further below).

The relevant semiconductor flat band potential (*E*_fb_, the potential where there is no electric potential drop between the semiconductor and electrolyte and the bands of the semiconductor are flat, as opposed to “bent” in the presence of a net electric field) is often compared to the thermodynamic potential of a fuel-forming half-reaction as a preliminary assessment of the ability of the semiconductor to drive that reaction.^43,44^ However, caution is warranted for both the use of vacuum-derived band structures and experimental determination of *E*_fb_.^45^ Direct comparison of vacuum-derived band structures to solution-based thermodynamic equilibrium potentials is often challenging due to the significant error introduced by band edge shifting effects, including (un)intentional surface modifications and heterogeneity, interfacial charging, and/or solution reorganization that can substantially modify *E*_fb_.^41,46,47^ Measurements in contact with electrolyte can address some of these challenges, but the complexity of measuring *E*_fb_ (through electrochemical impedance spectroscopy and Mott–Schottky analysis as well as other methods such as spectroelectrochemistry) has recently been discussed, highlighting both the care that must be taken in data interpretation and the variety of factors (e.g., semiconductor surface, electrolyte composition, etc.) that can influence the data output.^43,44,48–50^

While the band gap sets a fundamental limit on the current density and photovoltage that a semiconductor photoelectrode can produce under defined illumination, interfaces driving selective charge carrier transport are also critical to efficient *V*_ph_ generation. These interfaces can be designed through a number of strategies, including the commonly invoked interfacial “band bending” at rectifying semiconductor|electrolyte contacts in which an electric field selectively supports transport of electrons or holes across the interface,^22^ or carrier-selective semiconductor|semiconductor junctions, where a mismatch between band edges traps carriers of one sign while transporting the other.^51,52^ Use of these strategies has demonstrated the highest PEC device performances to date.^53,54^ By controlling and intentionally designing the optoelectronic properties of semiconductor photoelectrodes, the number of photons converted into charge carriers that are sufficiently energetic to drive PEC fuel-forming reactions can be improved, contributing to increased η_STF_ (Figure 2c).

### 1.2 Thermodynamic and Kinetic Barriers to Fuel-Forming Reactions on Photoelectrodes

Although optoelectronic properties—band gap, band edge positions, and charge transport properties—are crucial to determining PEC performance, there are substantial barriers to implementing all known semiconductors as photoelectrodes. Alternative reaction pathways can divert charge carriers to nonproductive processes, including carrier loss (recombination or trapping; Figure 2b, 4) or chemical changes at the semiconductor surface (degradation, reconstruction, or oxidation; Figure 2b, 3),^55,56^ both of which negatively impact η_STF._ Selecting a semiconductor for a photoelectrode must then address these issues in sequence: first, what fraction of the solar spectrum will be utilized (optoelectronics), and second, promotion of desirable reaction kinetics (catalysis) and prevention of deleterious reactions (stability).

Quantum efficiency (the fraction of incident photons that are converted to current) of a semiconductor is a function of absorption coefficient, photogeneration yield, and spatially dependent charge carrier collection efficiency.^39,55^ Collection efficiency can be improved by improving bulk and surface electronic properties (e.g., mobility, recombination), but photogeneration yield, the ratio of electron–hole pairs generated per absorption event, is an intrinsic material property. Understanding such intrinsic properties provides tremendous insight into the fundamental limitations of PEC device efficiency. However, deconvoluting photogeneration yield from quantum efficiency is challenged by the spatial variance of charge carrier collection efficiency:^55^ semiconductors early in development frequently suffer from uncontrolled defects that can act as recombination sites, in comparison with highly developed semiconductors where defect concentrations are low.

Once a photogenerated charge carrier arrives at the semiconductor|electrolyte interface, it either participates in a Faradaic process (e.g., fuel-forming or degradation reaction) or recombines.^15,56^ The rate of Faradaic charge transfer, which often involves concerted proton transfer for fuel-forming reactions,^57^ depends greatly on the energetic landscape of reactants at the semiconductor|electrolyte interface,^58^ where semiconductors frequently bind rate-limiting intermediates either too strongly or weakly to facilitate turnover.^59,60^ Activation barriers for rate-limiting steps impose an additional overpotential (η_O_) beyond that required by thermodynamics (i.e., *E* ^0^) to drive a reaction. Kinetics of reactions with many steps tend to be slower than simpler processes, the prototypical example of which is the contrast between kinetically sluggish, four-electron OER and facile, two-electron HER.^61^ OER η_O_ between 0.3–0.5 V are often required to produce 10 mA cm^–2^ in dark electrocatalysis. Similar assessments of overpotential for photoelectrochemistry are challenged by the convolution of catalytic and optoelectronic properties, but assuming a similar kinetic barrier for PEC results in a band gap of at least 1.6–1.8 V required to drive unassisted water splitting.^15,56,59^ Facile redox conversions, often involving outer-shell electron transfer processes, are commonly compared against rates of more complex reactions to assess surface charge collection efficiency, though we note this approach is unable to deconvolve coupled rate dependences of electron vs proton transfer.^57^ Catalysts are often incorporated to decrease η_O_, lowering the potential energy requirements for light-driven chemistry to occur. Introduction of catalysts by layering on or directly engineering active sites into the semiconductor surface affords the possibility of surface protection, though it can also lead to additional routes for carrier recombination.^62^

Degradation processes further compete for photogenerated charge carriers and undermine photoelectrode stability. When semiconductor photoelectrode self-oxidation or reduction potentials lie within the band gap and within the potentials of the fuel-forming half-reactions, the kinetics of degradative self-oxidation or reduction reactions become competitive with fuel-forming reactions.^41,63^ Such degradation can occur in the dark, as near-equilibrium or non-Faradaic processes (i.e., disproportionation),^64^ or under illumination, when photogenerated carriers are directly consumed in corrosion reactions.^65^ In both cases, a thermodynamic treatment of the problem does not provide insight into the relative *rate* of degradation, possibly leading to the exclusion of some semiconductors that in fact degrade very slowly and thus have relatively high practical photostability. This fact has recently become more appreciated in the literature, leading to the development of models to more accurately describe photostability behaviors.^66^

### 1.3 Photoelectrode Design Strategies

PEC fuel formation is closely related to coupled photovoltaic–electrochemical (PV-EC) and photocatalytic (PC) fuel formation (Figure 3a). All three approaches for the generation of fuels from sunlight are dependent on semiconductors for light capture to drive electrochemical reactions. While each of these approaches utilize semiconductors to convert solar photons into charge carriers, we describe their differences arising from the distance between generation of charge carriers and chemical fuels, introducing unique challenges that each approach must address.

**Figure 3:**
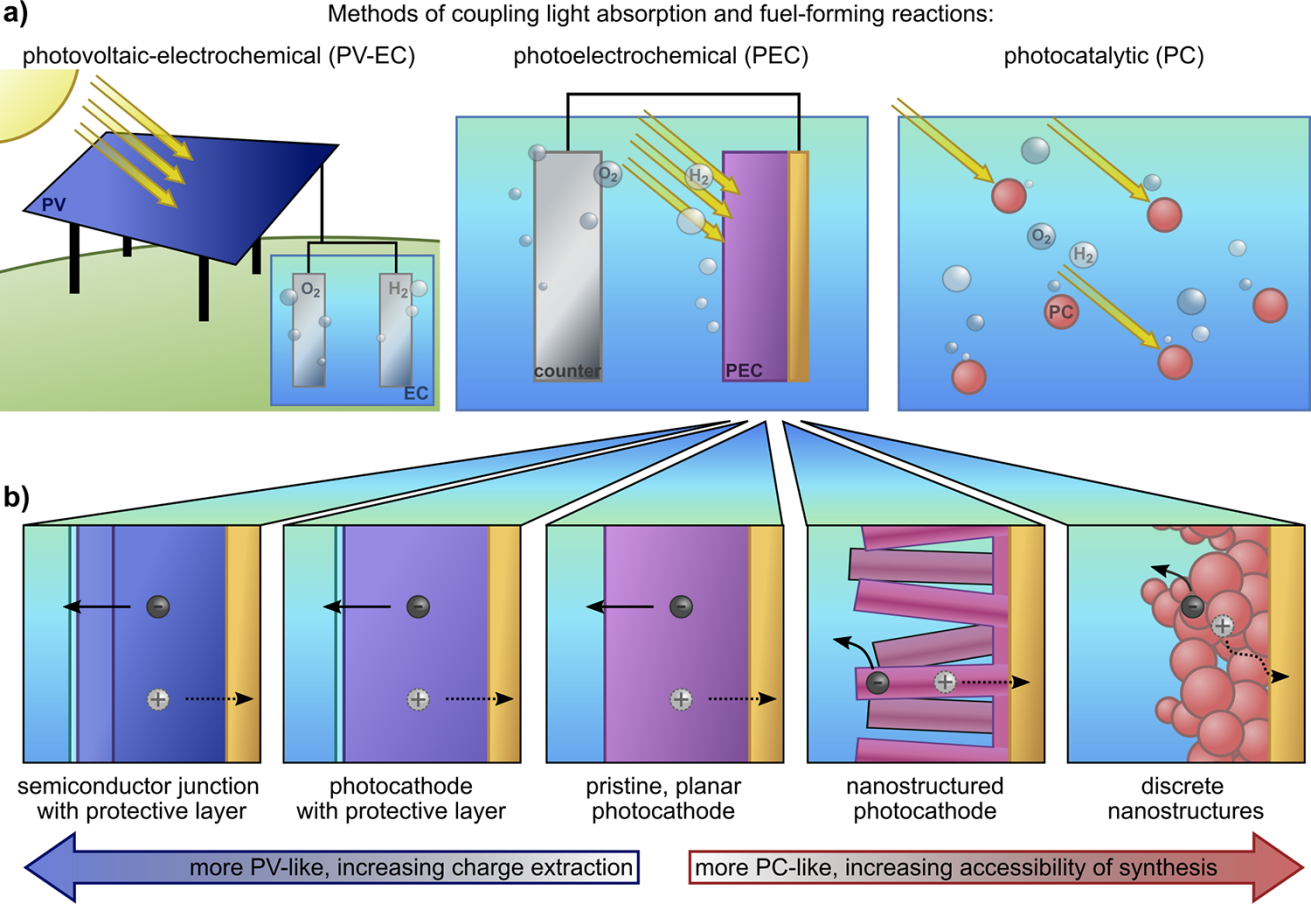
(a) Comparison of photovoltaic-electrochemical (PV-EC), photoelectrochemical (PEC), and photocatalytic (PC) solar fuel generation strategies. (b) Schematic presentation of different photoelectrode structures that can be used for PEC, illustrating the conceptual spectrum between those structures and related PV-EC and PC concepts, respectively. Note that catalysts are incorporated in many photoelectrodes but are omitted here for simplicity.

In PV-EC, light capture and fuel formation are performed by separate, independently optimized systems: a PV device converts sunlight into charge carriers that then perform fuel-forming reactions in an electrolyzer.^11^ Integration requires only consideration of electrical losses between the two systems, as interfacial interactions between semiconductor and catalyst or electrolyte are removed; this means that the performance of the two devices in series can be readily modeled. Although PV-EC systems are technoeconomically mature, in the sense that both technologies are commercially available, coupling a PV device to an electrolyzer can be a challenge due to their different operating points (electrolyzers are frequently designed to operate at much higher currents than those generated by commercial PV).^5,11,25^

In PC, particulate photoabsorbers are dispersed in a liquid phase, driving both reduction and oxidation reactions in nanoscale proximity without any transfer of charge beyond the environment local to an individual particle.^67,68^ In PC, charge carriers need only be transferred to the surface of the photocatalyst particle in order to drive fuel-forming reactions. However, carrier generation cannot easily be directly measured, and η_STF_ is frequently low, whether due to poor light absorption in the nanoparticles (which may not be large enough to absorb a meaningful fraction of incident photons), carrier recombination (due to poor charge separation in the particles), or interfacial reactions of electrolyte and semiconductor.^67,68^ PC systems are plausibly low-cost compared to the highly optimized components of PV-EC systems, but there are few examples of deployed systems.^69^

In PEC, light absorption and catalysis are coupled into a single unit, where a macroscopic photoelectrode is in intimate contact with the electrolyte where fuel-forming reactions occur. As in PC, photogenerated charge carriers need only travel a short distance (<mm scale) to drive reactions, and the light absorber is often exposed directly to deleterious reactions with the electrolyte. Conversely, as in PV-EC, the photoelectrode semiconductor in PEC can have optimized thickness to fully absorb above-band gap light. PEC is in principle advantaged over PV-EC systems in that conversion losses are reduced by directly integrated operation, although the challenges of direct semiconductor|electrolyte interfaces should not be neglected.^25,26^ PEC architectures are generally designed through identification of semiconductors that are stable under operation while demonstrating catalytic activity for the reaction of interest or by judicious coupling of semiconductor, protection, and catalyst layers. To date, the high bar of designing a photoelectrode which operates at high efficiency for long periods has not been met, and PEC fuel formation has yet to achieve deployment.^6,26^

While detailed efforts have been made to define subcategories of PEC devices,^11,23,70^ in practice the distinctions between the three approaches to solar fuels generation detailed above are still imperfect and often overlap. “Photoelectrochemistry” can be performed within two scopes: narrowly, using photoelectrodes where the semiconductor has direct interaction with electrolyte (Figure 3b, center panel); or, more broadly (Figure 3b, all panels), by any “integrated” solar fuels system in which light-absorbing semiconductor, catalysts, and protective schemes are intentionally codesigned to generate solar fuels in a way that is distinct from a “by-parts” configuration (e.g., a PV device electrically wired to an electrolysis unit). A single semiconductor in direct contact with electrolyte forms the simplest possible PEC photoelectrode, where the photovoltage generated at the semiconductor|electrolyte rectifying interface drives fuel-forming reactions. The addition of protective layers or other semiconductor layers creates a photoelectrode with increasing similarities to a PV device, where the photovoltage is derived not from rectification at the semiconductor|electrolyte contact, but rather at a semiconductor|semiconductor junction (Figure 3b, far left). It should be noted that PEC systems where a “buried” photoactive semiconductor junction is present have also been referred to as PV-EC systems in the literature;^11^ for the purposes of this review, we consider such devices photoelectrodes and discuss their chemistry. Alternatively, nanostructuring enhances photoabsorption by lowering reflectance while decreasing the required diffusion length for photogenerated charge carriers to reach a semiconductor|electrolyte interface, resulting in photoelectrodes that are more PC-like but allow for direct potentiostatic control (Figure 3b, far right). In this review, we discuss photoelectrodes falling within the broader scope of photoelectrochemistry while focusing on the underlying chemistry and properties of the semiconductor that forms the majority of the photoelectrode device.

### 1.4 Scope of This Review

This review aims to capture recent developments in the chemistry of photoelectrodes used in PEC fuel formation. While catalysts and protective layers are addressed, the primary focus of the review is the inorganic semiconductor that forms the basis for the photoelectrode, from recently discovered semiconductors to well-established ones. To emphasize studies on the chemical fundamentals underlying the operation of these semiconductors as photoelectrodes, thin-film and otherwise continuous semiconductors are the focus, as those geometries better enable studies of surface chemistry and interfaces compared to nanoparticulate semiconductors.^68,71^

We begin this review by discussing recent progress on the large number of semiconductors used as PEC fuel-forming photoelectrodes, focused particularly on H_2_ generation via water splitting. We then focus on in situ experimental techniques that enable better understanding of fuel-forming processes on photoelectrodes. Finally, we use a reaction-specific perspective to discuss applications of the various photoelectrode semiconductors to the generation of different fuels, highlighting lessons from water splitting that can be broadly applied.

## 2 Progress in Photoelectrode Chemistries

The half-century of PEC research has seen huge advances in the chemical understanding of semiconductors used as photoelectrodes. For the majority of this period, PEC was developed in parallel with PV, with early studies establishing the properties of semiconductor-liquid junctions using Si, III–Vs, and CdTe.^72–77^ Since the start of the 21st century, oxide semiconductors like BiVO_4_ have been targeted as photoanodes, either to drive overall water splitting while performing OER or to support a photocathode in a tandem configuration.^78,79^ Most recently, computational predictions have led to experimental investigation of increasingly complex semiconductors, often specifically tailored in an attempt to overcome deficiencies of previously identified photoelectrodes by, e.g., modifying band gap or improving stability. Further increasing the complexity of the photoelectrode design space, nearly all of these semiconductors can be found integrated with charge-selective contacts, buried junctions, or catalysts in order to improve carrier extraction and reactivity.

Simplifying this complex picture, photoelectrode design must optimize between three separate, and often mutually exclusive, aspects as discussed in the introduction (Figure 1b): *optoelectronics*, *catalysis*, and *stability*. Conceptually (Figure 2c), designing around *optoelectronic* properties pushes *j*_SC_ toward theoretical maximums by utilizing a greater fraction of solar photons, while designing around *catalysis* increases the electrochemical driving force by maximizing *V*_ph_ and minimizing losses (such as η_O_). These performance-driven parameters must further reside in crystalline structures that remain *stable* under operating conditions, with many optoelectronically favorable materials suffering from poor stability.^41^

Because of the complex considerations underlying photoelectrode design, it is hard to succinctly capture metrics related to optoelectronics, catalysis, and stability. Rather, proxy values for efficiency are often used, and we begin our discussion there. Achieved and theoretical *j*_SC_ are compared for semiconductors used as photocathodes (in Figure 4a) and photoanodes (in Figure 4b). In each panel, marker area denotes time each photoelectrode has been investigated and the dashed line denotes parity between achieved and theoretical *j*_SC_. These comparisons capture only examples where there is a single semiconductor light absorber in the photoelectrode (bold labels) or where there is a heterojunction present (italic labels). The benchmark photoelectrode architectures and current densities used in Figure 4 are reported in the Supporting Information (SI), Tables S1–S6, which has additional entries if there are literature demonstrations of both classifications. Of particular note are III–V compounds such as Ga_1–*x*_In_*x*_P (frequently described as GaInP_2_), a moderate *E*_g_ semiconductor frequently used in tandem photocathodes with a GaAs bottom cell (i.e., GaAs|GaInP_2_, where photoelectrode architectures are listed from left to right in order of substrate to electrolyte-facing layers),^80,81^ and chalcogenides such as CuIn_1–*x*_Ga_*x*_Se_2_ (CIGS) and Cu_2_Cd_*x*_Zn_1–*x*_SnS_4_ (CZTS), which are frequently coupled with CdS in heterojunctions.^82,83^ While many of these materials have also recently been investigated to drive other PEC reactions, we focus here on application of photoelectrodes to water splitting (HER and OER) and defer discussion of other reactions to section 4.

**Figure 4:**
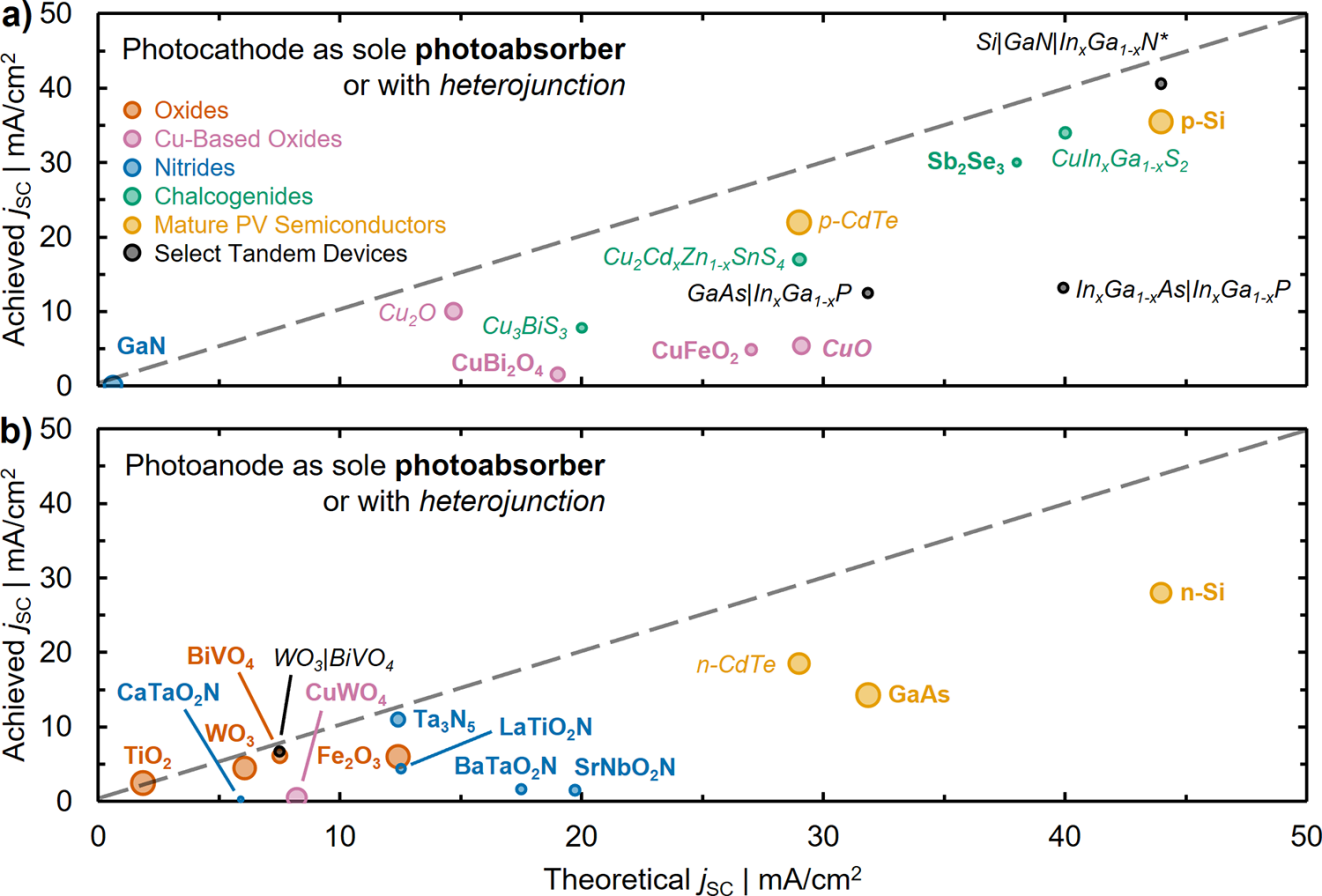
Comparison of achieved vs theoretical photocurrents for each of the (a) photocathodes and (b) photoanodes discussed throughout this review. Marker area is proportional to the amount of time each photoelectrode has been investigated. Photoelectrodes are broken down into classes of metal oxides (orange), copper oxides (pink), nitrides (blue), chalcogenides (green), and mature PV semiconductors (yellow). A selection of tandem devices are also presented (black). Achieved *j*_SC_ are intended to best capture the intrinsic limitations of each photoelectrode, where we distinguish between cases where the photoelectrode is composed only of the semiconductor (bold text; can include cocatalyst) or where heterostructures are incorporated (italicized text). Data underlying this figure, including benchmark photoelectrode architectures, can be found in Tables S1–S6 of the Supporting Information (SI). *Note that the Si|GaN|In_*x*_Ga_1–*x*_N photoelectrode record from (a) is set in 1 M HBr, see SI.

Some overarching trends in the development of photoelectrodes are evident from inspection of Figure 4. Most semiconductors used as photocathodes have theoretical *j*_SC_ > 15 mA cm^–2^, while most used as photoanodes have theoretical *j*_SC_ < 12 mA cm^–2^. This is a direct consequence of the stability of metal oxides as OER photoanodes, which has popularized these wide *E*_g_ semiconductors for this application. Several metal oxides have a long history of use as photoelectrodes (larger circle area), with achieved *j*_SC_ nominally converging on theoretical limitations, even when that limit is low. In contrast, some chalcogenide semiconductors have emerged much more recently and are approaching their theoretical *j*_SC_ applied as photocathodes, despite challenges with stability for those materials. Mature PV semiconductors face similar challenges of stability but focused development for PV applications has led to very high *j*_SC_ when applied as photoelectrodes. The benchmark *j*_SC_ of nitrides as well as copper-based oxides largely falls well short of theoretical values due to their short investigative histories, with Cu_2_O and Ta_3_N_5_ representing two exceptions.^84,85^

To facilitate the discussion of the large group of semiconductors used as photoelectrodes, we consider each from the perspective of chemical factors on the road to high η_STF_. We divide those chemical factors into two categories: fundamental to a semiconductor, and relevant to its application as a photoelectrode (Figure 5, top). Fundamental factors for obtaining semiconductors with high radiative efficiency in charge generation are *synthesis* routes that enable identification of intrinsic *properties* through controlled levels of *defects*, which can span from crystalline defects to common dopants. Although these fundamental factors are important to general semiconductor development, PEC photoelectrode applications further require *stability* in electrolyte, *catalytic activity*, and control over *surface and morphology*. Each of these factors is important to the development of optically active, highly catalytic, and stable photoelectrodes, but they do not improve at the same rate. We divide the progression of fundamental and applied chemical factors into rough periods of early, improving, and mature understanding (Figure 5, bottom). Because some semiconductors have been quickly developed while progress in improving the properties of others has been slow, we do not assign these periods to discrete time intervals, but rather to the extent to which fundamental properties and applications to PEC are understood.

**Figure 5:**
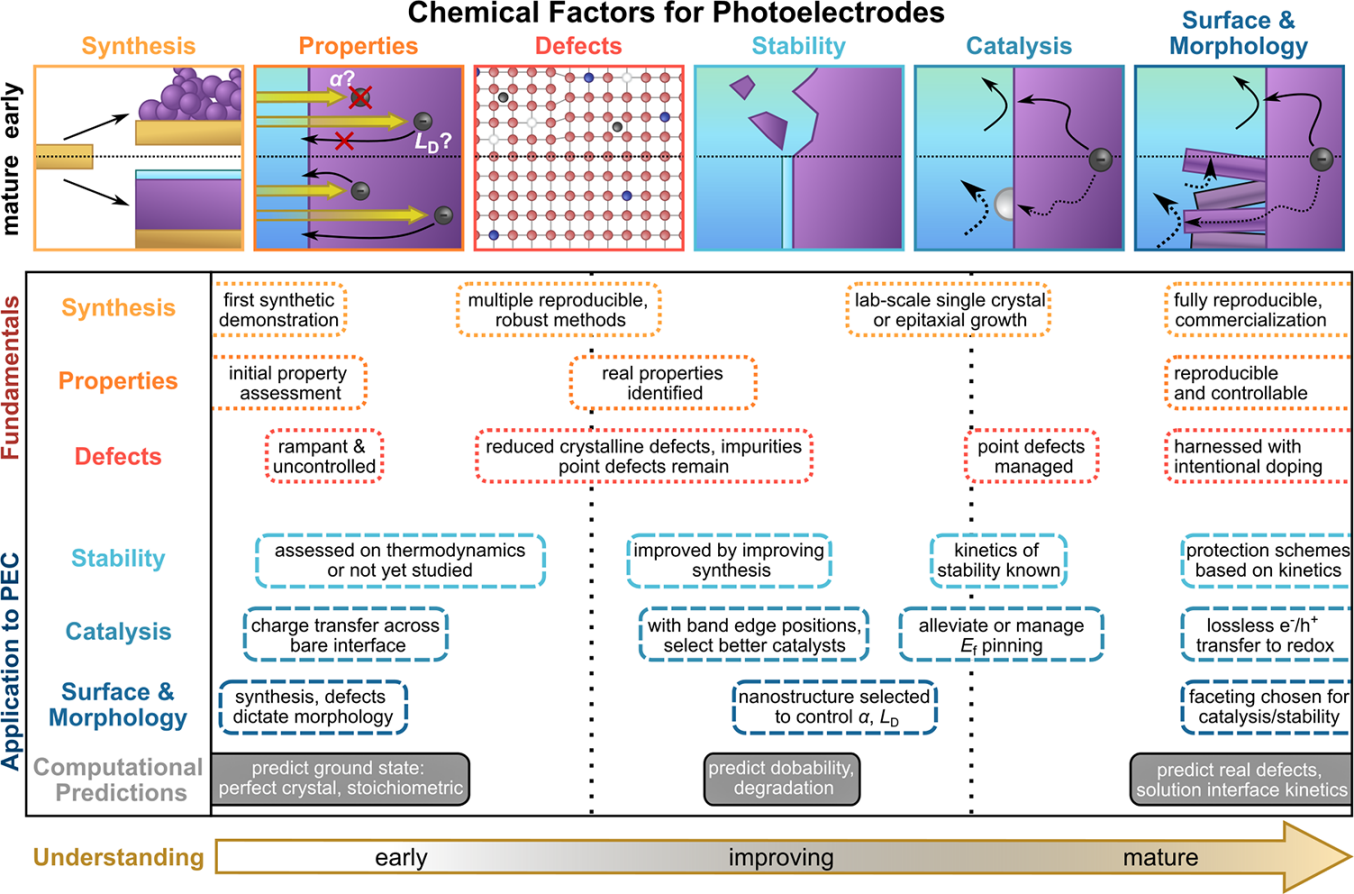
Chemical factors in the development of photoelectrodes for PEC fuel-forming reactions and their common progression in materials development. Schematics for each factor (top) show the general progression from early to mature understanding. Descriptions of factor progression (below) provide high-level stages in development along each factor. Color scheme adapted from ref 86.

We use this framework to understand the progression of research and chemical control of a semiconductor, rather than to bin characteristics as desirable or undesirable. For instance, even when the synthesis, defects, and properties of a semiconductor are fully understood and controlled, it may not be deployable as a photoelectrode because its properties are not well-suited to PEC (e.g., wide-band gap TiO_2_, section 2.1.1). This framework also illustrates how discoveries that improve one factor for a photoelectrode can influence other factors. Improvements in synthesis eliminating particular defects can improve stability, if those defects are particularly prone to corrosion or dissolution. Alternatively, studies of facet stability under operating conditions can improve both catalysis and necessitate the development of alternative synthesis methods to favor the expression of those facets. Although computational studies are not a main focus of this review, it is critical to consider that such studies generally begin with thermodynamic assessment of the ground state of a semiconductor and its stability (Figure 5, bottom), an idealized state that may not accurately describe a physically realizable semiconductor photoelectrode until after many years of development. As a semiconductor is developed, so too is its computational model, such that highly developed semiconductors can meet with computational descriptions that encompass the real properties of the material at the point of mature understanding.

Because these chemical factors are generally investigated in parallel, rather than in sequence, it is difficult to determine a priori a route toward an efficient, stable photoelectrode. The diversity of candidate photoelectrode semiconductors (see SI Figure S1 for an illustration of the range of band energetics) shows that the field has not converged on a single strategy to improve the chemical factors highlighted above, which represent only one perspective on the development of high η_STF_. The myriad semiconductor choices are further complicated by the range of other materials that can be integrated with each semiconductor to build a photoelectrode. In this section, we aim to illuminate the range of semiconductors that are currently investigated as photoelectrodes, highlighting areas where each excel and identifying factors that must be addressed to bring PEC performance closer to its theoretical maximum. We first introduce overarching characteristics of each material class, followed by discussion of recent studies for specific semiconductors belonging to that class. For each semiconductor, we discuss progress across the chemical factors controlling its PEC performance, noting that any of the six factors described above may be more or less critical to future progress. We also highlight integration of the photoelectrode semiconductors with other materials to improve optoelectronic properties, catalysis or stability. For each semiconductor, we provide examples of investigations that advance the state of the art for PEC applications or the understanding of fundamental photoelectrode processes.

### 2.1 Oxides

Metal oxides represent the first class of semiconductor photoelectrodes, beginning with the 1972 demonstration of OER on rutile TiO_2_ single crystals.^8^ Metal oxides display a diverse range of crystal structures (Figure 6a), where oxygen surrounds metal cations in octahedral (*O*_h_ e.g. rutile TiO_2_, corner-sharing monoclinic WO_3_, corundum α-Fe_2_O_3_), tetrahedral (*T*_d_, e.g., monoclinic scheelite BiVO_4_), and more complex geometries (e.g., mixed *O*_h_ (B^3+^) and *T*_d_ (A^2+^) spinel (A^2+^B_2_^3+^O_4_)). The robust, defect-tolerant chemistries of these metal oxides afford high-quality material from multiple synthetic routes, enabling isolation of defect properties by way of well-controlled growth techniques including physical vapor deposition (PVD) strategies such as pulsed laser deposition (PLD) and molecular beam epitaxy (MBE).^87,88^ Cation interstitials, charge compensated by oxygen vacancies, provide intrinsic n-type doping in most metal oxides (e.g., TiO_2_, BiVO_4_, α-Fe_2_O_3_, WO_3_),^89^ though several cuprous oxides display intrinsic p-type doping due to native cation vacancies (see section 2.1.5).^90,91^ Metal oxide photoanodes are generally stable in the highly oxidizing, alkaline conditions of OER;^41^ in contrast, metal oxide photocathodes are thermodynamically unstable at the severely reducing, acidic conditions of HER. In the terms laid out in Figure 5, the field has achieved a mature understanding of many metal oxides. However, despite early promise and their stability in aqueous environments, the typically large band gaps (Figure 6b) and polaronic charge transport properties of metal oxides continue to hinder their use as highly efficient photoelectrodes.^92,93^

**Figure 6:**
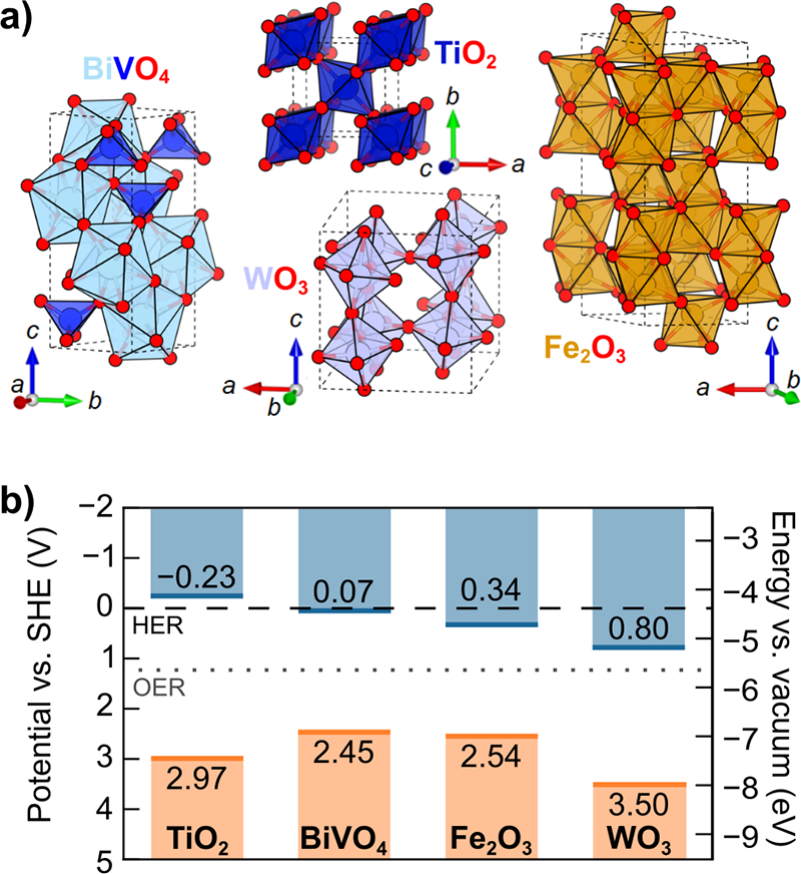
Selected metal oxide semiconductors for photoelectrodes. (a) Unit cells of BiVO_4_, TiO_2_ (rutile), α-Fe_2_O_3_, and WO_3_. (b) Valence band edge maximum (orange line) and conduction band edge minimum (blue line) for each semiconductor with the thermodynamic potentials for HER (black dashed line) and OER (gray dotted line). See SI Tables S7 and S8 for details.

Despite misalignment of the optoelectronic properties of metal oxides and their applications to PEC, the facile synthetic routes and expansive literature on these semiconductors have enabled thorough investigation of the fundamentals underlying the PEC performance of metal oxides. Under light-limited conditions, PEC charge transfer kinetics are conventionally thought of as the product of a potential-independent reaction rate constant and the surface density of accumulated charge carriers (i.e., surface holes for photoanodes).^94–96^ This population-based description assumes a mechanism where each elementary charge transfer event in a reaction is mediated through a single surface site. However, recent spectroelectrochemical investigations of common metal oxide photoanodes have identified that OER rate order increases from first to third order with increasing photogenerated surface charge carrier density (Figure 7).^97–99^ While subtle, this observation bears tremendous mechanistic implications for PEC reactions on oxide surfaces. Rate orders greater than unity suggest the possibility of multisite reaction mechanisms, where charges, or even intermediates via chemical coupling steps, from several adjacent sites can be synchronously transferred. Investigation of this phenomenon may yield additional insight into material-specific catalytic mechanisms. For instance, α-Fe_2_O_3_ demonstrates facet-dependent hydroxylation and oxygen vacancy formation affinity,^100^ while charge carriers localize at oxygen vacancies in other metal oxides (i.e., TiO_2_).^101,102^ By tuning surface facet to colocate accumulated surface charge carriers and reactive adsorbates (i.e., hydroxide ions), the transition point between first and third order reaction rate on surface hole density may prove tunable, facilitating greater photocurrents under fixed fluence and expanding the utility of these oxide semiconductors as photoelectrodes.

**Figure 7:**
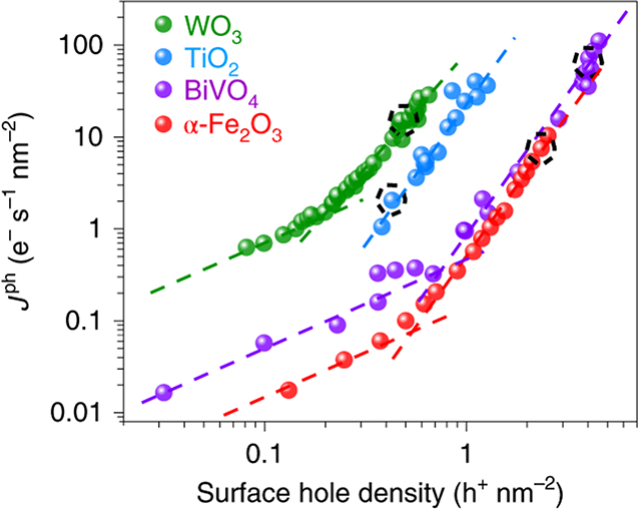
Log–log plot of photocurrent (*J* ^ph^) vs surface hole density, probed by transient absorbance spectroelectrochemistry. As surface hole density increases, the rate order of surface hole density shifts from first to third order. Black dashed circles denote incident photon flux equivalent to ∼one sun. Surface hole density is tuned by changing incident photon flux from a 365 nm light source under the following measurement parameters: pH 3 at 1.4 V_RHE_ for WO_3_ (green); pH 13 at 1.5 V_RHE_ for TiO_2_ (blue); pH 7 at 1.7 V_RHE_ for BiVO_4_ (purple); and pH 13 at 1.5 V_RHE_ for α-Fe_2_O_3_ (red). Reproduced with permission from ref 99. Copyright 2020 Nature Portfolio.

While the appreciable quantum efficiencies of wide band gap semiconductors (e.g., TiO_2_ and SrTiO_3_) drew initial interest,^8^ their inherently poor solar spectrum utilization limits η_STF_ and their application as photoelectrodes.^103^ The polaronic conduction mechanisms of highly ionic metal oxide semiconductors further limit charge carrier collection efficiencies.^92,101^ Thus, metal oxides with large band gaps (≥3.0 eV) have often served as model systems to understand fundamental properties underpinning absorption, charge transfer, and kinetics of semiconductor photoelectrodes. In contrast, intermediate band gap (∼2.0–3.0 eV) metal oxides achieve greater solar photoabsorption cross sections and have emerged as promising photoanode candidates (e.g., α-Fe_2_O_3_ and BiVO_4_). In this section we provide brief overviews of a series of oxide photoelectrodes representing either model systems (e.g., TiO_2_) or semiconductors with promising optoelectronic and catalytic properties (e.g., BiVO_4_, α-Fe_2_O_3_, CuWO_4_, Cu_2_O, CuBi_2_O_4_), focusing on areas of continued exploration for photoelectrode applications.

#### 2.1.1 Titanium Dioxide (TiO_2_)

The well-understood physicochemical properties, controllable dopants, and chemical robustness of TiO_2_ have made it a model photoelectrode for the development of fundamental PEC structure–property relationships, despite its 3.0 eV bandgap^104,105^ manifesting in low solar spectrum utilization and poor theoretical η_STH_ (2.2%).^103^ Here, we discuss design strategies that have been explored for TiO_2_ to enhance visible light absorption, and fundamental investigations into the role of transition metal dopants and oxygen deficient surfaces on OER kinetics, focusing on rutile TiO_2_. We highlight literature that captures the opportunities and limitations of the tunability of TiO_2_ optoelectronic and catalytic properties, aiming to inspire future research directions for other semiconductors that can better utilize the solar spectrum. For more comprehensive discussions of TiO_2_ as a photoelectrode we refer readers to dedicated reviews.^101,106,107^

Facile synthetic routes and chemical stability in both acidic and alkaline electrolytes promote the use of TiO_2_ as a photoelectrode (and as a protective layer, discussed elsewhere). Synthetic methods range from solution-based (e.g., spin coating, hydrothermal)^108,109^ to well-controlled vacuum based deposition techniques (e.g., PLD or MBE),^87,110^ affording broad control over morphology and nanostructure. Similar to other oxides, intrinsic n-type cation interstitial (Ti_*i*_^3+^) concentrations are sufficient to shift *E*_F_ to near the conduction band minimum^111–113^ of 0 V_RHE_.^46,114^ Stoichiometric TiO_2_ is poorly conductive (mobility of 0.3 cm^2^ V^–1^ s^–1^)^115,116^ owing to a primarily polaronic conduction mechanism,^92,101^ though incorporation of dopants (Nb in particular)^117^ and mild reduction increase conductivity. Similar doping and reduction may serve to tune electronic transport properties of other metal oxides exhibiting polaronic conduction mechanisms.^118–120^

Decreasing the large *E*_g_ of oxide semiconductors like TiO_2_ represents one design strategy for increasing solar spectrum utilization. Chemical reduction (i.e., TiO_2–*x*_) or extrinsic doping^121–124^ represent the most successful approaches to decreasing the *E*_g_ of TiO_2_. Both strategies introduce states throughout the gap (Figure 8a) to enhance photocurrents of rutile TiO_2_ up to nearly 3 mA cm^–2^ under AM 1.5G illumination,^108,125^ well above the theoretical limit of stoichiometric TiO_2_ (1.85 mA cm^–2^).^103^ Reducing TiO_2_ increases the concentration of n-type Ti_*i*_^3+^ interstitials and oxygen vacancies,^87,126^ populating Ti 3*d* states just below the conduction band minimum^111–113^ and giving rise to visible photoabsorption.^127,128^ Incident photon-to-current efficiency (IPCE) in the ultraviolet (λ < 400 nm) regime varies with H_2_ annealing temperatures and times (Figure 8b),^125,129^ where increased defect density of nonstoichiometric TiO_2–*x*_ increases the deleterious contribution of impurity scattering (from, e.g., Ti_*i*_^3+^ and corresponding oxygen vacancies) toward mobility.^116,130,131^

**Figure 8:**
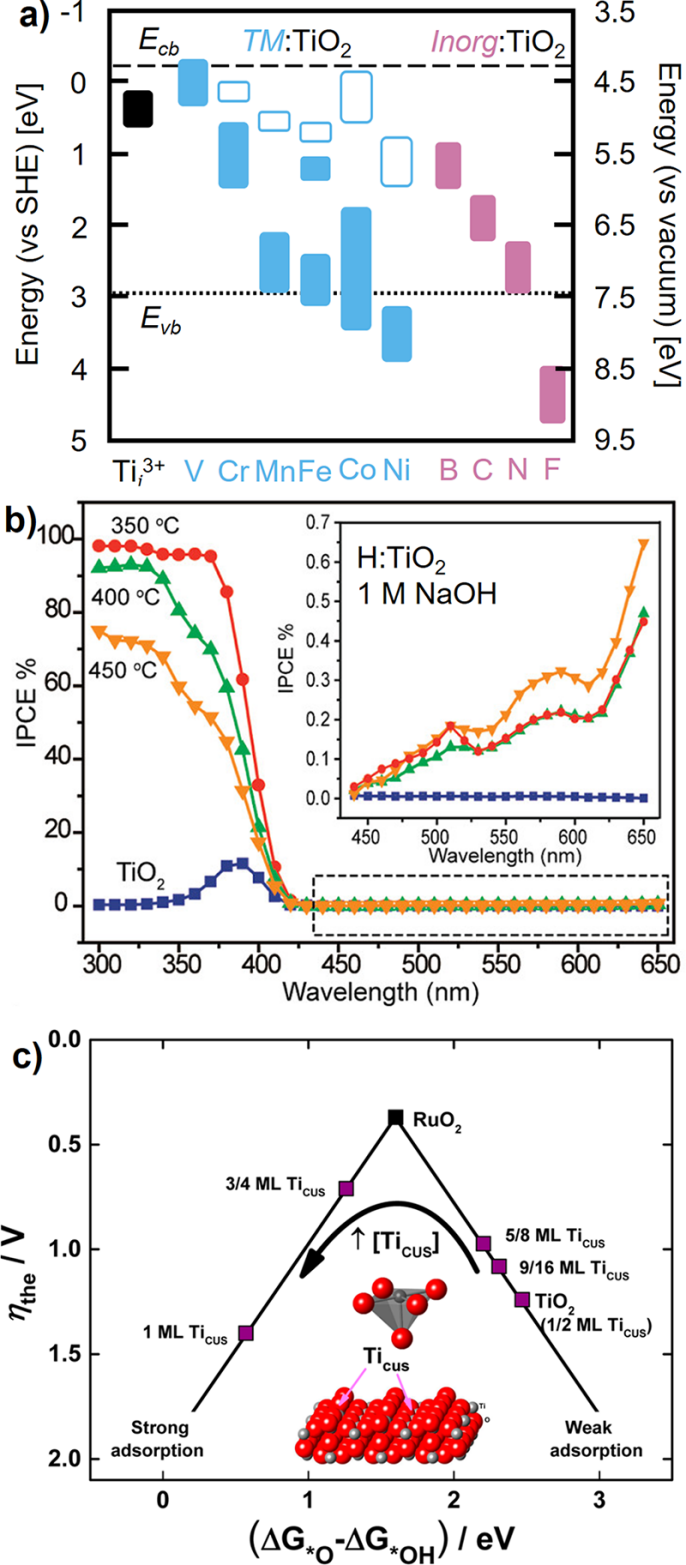
(a) Summary of occupied (filled) and unoccupied (open) calculated intragap state energies introduced by transition metal (blue: V, Cr, Mn, Fe, Co, and Ni) and inorganic (pink: B, C, N, O, and F) doped TiO_2_. Unoccupied states introduced by inorganic dopants may lie above the conduction band and are not shown. Data for transition metals adapted with permission from ref 123. Copyright 2008 IOP Publishing. Data for inorganics adapted with permission from ref 121. Copyright 2013 Elsevier. (b) IPCE of rutile TiO_2_ reduced in a hydrogen atmosphere at the denoted temperatures for 30 min, with selected wavelengths magnified in inset. Reproduced with permission from ref 125. Copyright 2011 American Chemical Society. (c) Density functional theory calculated theoretical OER overpotential against the free energy of hydroxide deprotonation (Δ*G*_O*_ – Δ*G*_OH*_) for stoichiometric and oxygen-deficient TiO_2_. The free energy of Δ*G*_O*_ – Δ*G*_OH*_ decreases with initial surface CUS site formation, increasing again beyond ∼50% surface CUS site density. Adapted with permission from ref 132. Copyright 2016 American Chemical Society.

However, understanding the impact of extrinsic, or additional intrinsic, dopant states is challenged by *j*_SC_ and *V*_OC_ being a convolution of optoelectronic and catalytic properties. OER kinetics on the surface of TiO_2_ are poor,^59,60^ but calculated reaction free-energy landscapes and dark electrochemical kinetic measurements can help evaluate how catalyst design strategies influence photoelectrode kinetics and rutile TiO_2_ can serve as a useful model system. The density of coordinatively undersaturated (CUS) sites, formed on oxygen-deficient metal oxide surfaces, tunes the hydroxide deprotonation free energy barrier that describes OER kinetics (Figure 8c).^132^ The difference in the adsorption free energy of surface oxides and hydroxides (Δ*G*_O*_ – Δ*G*_OH*_) decreases with increasing areal density of CUS sites,^132^ manifesting in greater oxygen evolution kinetics for weak-binding metal oxides (e.g., TiO_2–*x*_) and poorer kinetics for strong-binding metal oxides (e.g., NiO_1–*x*_, MnO_2–*x*_).^59^ Extending this concept to photoelectrochemistry on other metal oxides, coupling chemical reduction with additional doping strategies may then provide a useful strategy to enhance both solar spectrum utilization and intrinsic reaction kinetics.

Extrinsic doping similarly lowers the kinetic losses of TiO_2_ by improving OER kinetics.^133^ Density functional theory calculations identify the deprotonation of weakly bound hydroxide as the rate-limiting step for OER over TiO_2_ (Figure 8c).^59,60^ Substituting Ti sites with stronger-binding transition metal dopants decreases the free energy barrier for hydroxide deprotonation with Cr, Mn, Mo, and Ir dopants predicted to lower OER η_O_.^60^ An empirical survey of hydrothermally doped TiO_2_ nanorods finds *j*_SC_ decreases in order of Fe > Mn > Co dopants, although Mn displayed the greatest visible and ultraviolet (UV) absorptivity.^108^ Despite the poor control over doping densities and morphology afforded by hydrothermal synthesis, these examples illustrate the capacity for dopants to substantially alter the optical properties via formation of intragap states. The disconnect between PEC measurements and theoretical predictions may be due to the influence of charge carrier dynamics; while Fe-doped TiO_2_ demonstrates longer carrier lifetimes than Mn- or Mo-doped TiO_2_, the latter transition metals demonstrate improved OER kinetics.^134^ Similar doping strategies have been explored for inorganic dopants, where N-doping has received considerable attention for its capacity to increase TiO_2_ photocurrent within the visible spectrum.^87,110,135–138^ Atom percent incorporation of N (i.e., TiO_2–*x*_N_*x*_) decreases conductivity due to neutralization of interstitial Ti_*i*_^3+^ charge carriers by trivalent N^3–^ anions,^137^ manifesting in greater bulk recombination and poorer UV IPCE.^110,136,138^ Similar approaches applied to other metal oxide structures may prove beneficial in enhancing the intrinsic optoelectronic (absorptivity, charge transport) and kinetic properties.

#### 2.1.2 Bismuth Vanadate (BiVO_4_)

Although it was discovered more recently, monoclinic scheelite-type BiVO_4_ has quickly risen to become a prominent candidate photoanode. It has a 2.4 eV band gap and the appropriate band alignment to drive OER while absorbing a reasonable portion of the solar spectrum (Figure 6a).^79^ BiVO_4_ also displays good stability across aqueous electrolytes,^139^ and can be easily synthesized from abundant metals. However, the electron mobility of undoped BiVO_4_ is 0.04 cm^2^ V^–1^ s^–1^, nearly 2 orders of magnitude lower than most other metal oxides;^140^ this low stems from a high density of intrinsic trap states that only allow for electron movement with the help of lattice phonons. Because of its low electron mobility compared to hole mobility, BiVO_4_ is usually deposited on transparent substrates and illuminated from the substrate side, such that photogenerated electrons have a shorter distance to diffuse before collection at the contact while holes can diffuse to the electrolyte interface.^141^ BiVO_4_ suffers from poor conductivity, as well as poor oxidation kinetics that result in carrier pile-up at the semiconductor|electrolyte interface and subsequently, photocorrosion.^140,142^ The low electron mobility means BiVO_4_ is poorly suited to drive water splitting alone, so it is usually combined in tandem configurations. Even with these challenges, BiVO_4_ photoanodes are nearing their theoretical η_STF_ for OER, advancements which have been much discussed in the literature.^143–146^

Electrodeposition of BiVO_4_ is a common synthesis strategy and can be accomplished by a variety of precursors (Figure 9a).^147^ Highly porous BiVO_4_ can be made from the electrodeposition of BiOI from a *p*-benzoquinone aqueous solution.^147^ The nanoporous structuring of BiVO_4_ addresses issues with interfacial charge separation and transport by shortening the distance any carrier must travel to be collected at an interface; as a result, substantial efforts have been made to create uniform films of nanoporous BiVO_4_ (Figure 9b).^148^ In the sol–gel approach, Bi^3+^ and V^5+^ precursor solutions are hydrolyzed and then dehydrated to obtain a stable sol–gel for spin-coating onto a substrate.^149^ Metal–organic decomposition can also be used to produce BiVO_4_ photoanodes by spin-coating or spray-coating Bi- and V-containing organic precursors (e.g., bismuth 2-ethylhexanoate and vanadium­(IV) (oxy)­acetylacetonate in acetylacetone) onto substrates such as fluorine-doped tin oxide (FTO), followed by annealing at 400 °C for 30 min. This approach is preferred for doping BiVO_4_, but is overall less reproducible;^145^ the sol–gel approach generally avoids particle agglomeration and produces more uniform films.^149^ Although most studies focus on n-type BiVO_4_, p-type BiVO_4_ can be prepared hydrothermally by mixing Bi­(NO_3_)_3_ and NH_4_VO_3_ in ethylenediaminetetraacetic acid disodium; although the conduction band of this material is not quite negative enough to drive HER.^150^ Finally, thin-film deposition methods such as PLD can be used to synthesize BiVO_4_ by using a nanosecond laser pulse to ablate a BiVO_4_ target and thermally evaporate onto a substrate. However, PLD can result in nonstoichiometric deposition, due to the more volatile components of the target being preferentially ablated.^151,152^

**Figure 9:**
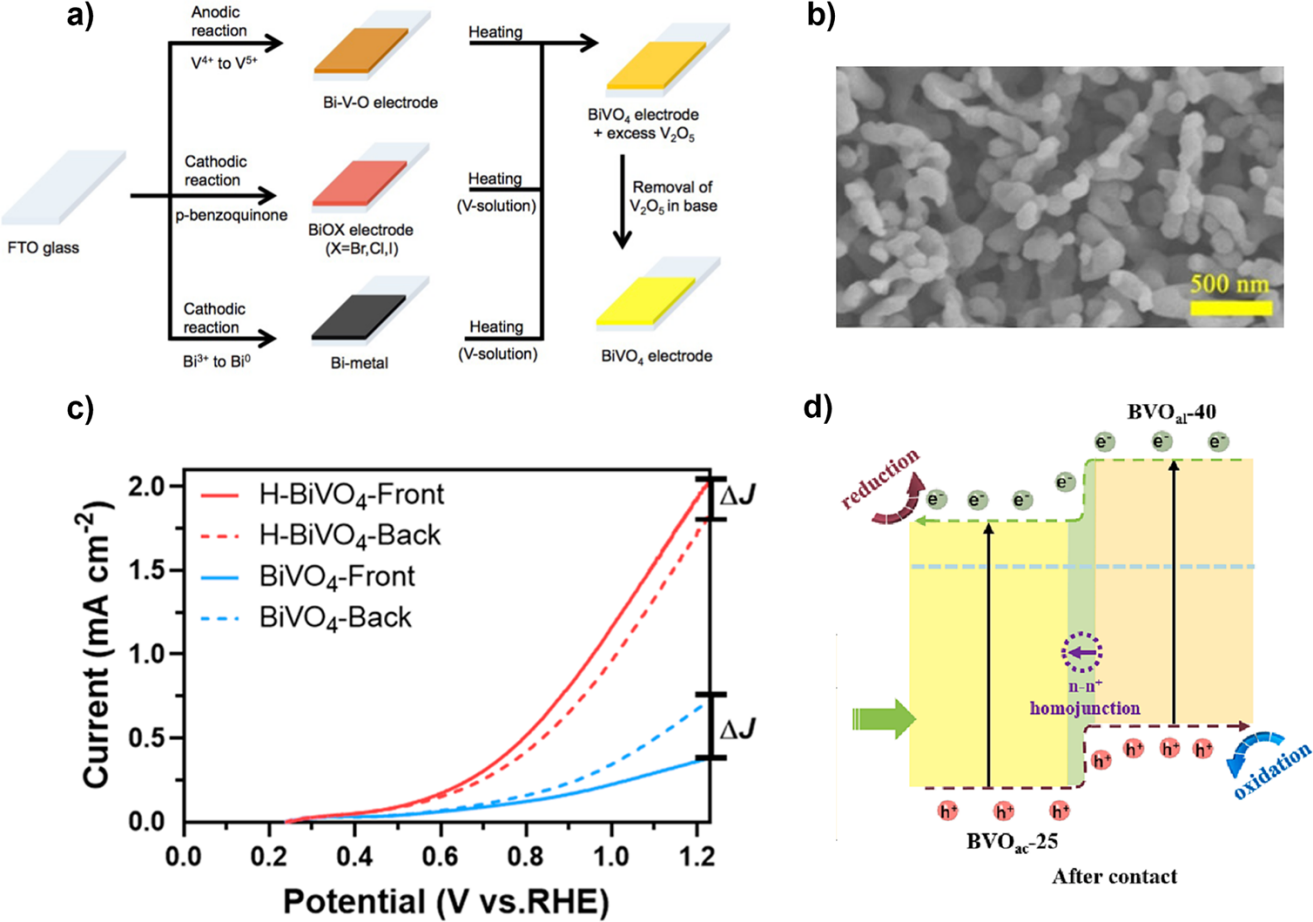
(a) Three electrodeposition routes for BiVO_4_ electrodes: (top) anodic deposition by oxidizing a Bi metal electrode in NH_4_VO_3_ solution to form amorphous BiVO_4_ which is crystallized by annealing; (middle) BiOX nanostructures are electrodeposited and when converting the BiVO_4_ by annealing the morphology remains; (bottom) highly porous Bi is deposited from a nonaqueous ethylene glycol solution and converted to BiVO_4_ in a VO­(acac)_2_ in DMSO solution. Adapted with permission from ref 147. Copyright 2015 American Chemical Society. (b) Scanning electron microscopy (SEM) image of nanoporous BiVO_4_ obtained by annealing electrodeposited nanoporous BiOI film with V^5+^ source. Adapted with permission from ref 153. Copyright 2018 Wiley-VCH. (c) Current density–voltage (*J*–*V*) curves of BiVO_4_ and H-doped BiVO_4_ illuminated from the front or back side. H-doping improves the mobility of electrons and decreases the recombination of carriers, thereby improving the photocurrent regardless of the direction of illumination. Adapted with permission from ref 154. Copyright 2023 American Chemical Society. (d) The electronic band structure of a n–n^+^ BiVO_4_ photoanode, where the n^+^ region is created using oxygen vacancies. Not shown: FTO back contact facilitating reduction using the photogenerated electrons from the BiVO_4_ at a counter electrode. Adapted with permission from ref 155. Copyright 2023 Elsevier.

BiVO_4_ can be doped extrinsically or by leveraging oxygen vacancies, an intrinsic defect common to metal oxides. In BiVO_4_, oxygen vacancies are electron donors, improving carrier concentration and conductivity.^156^ Reducing BiVO_4_ films in a K_2_B_4_O_7_ solution has been shown to increase the concentration of V_O_.^157^ Coupled with a VO_*x*_ catalyst, these photoanodes reached 6.29 mA cm^–2^ at 1.23 V_RHE,_ a 4-fold increase over BiVO_4_ synthesized by the same method without the addition of oxygen vacancies, and were stable under OER operation at pH 9.5 for 40 h at 0.6 V_RHE_. The improved photocurrent density was attributed to oxygen vacancies resulting in V orbitals with unpaired electrons which promote water oxidation, increase conductivity, and improve charge transfer.^157^ Extrinsic doping of BiVO_4_ can be achieved in most synthesis methods by simple addition of metal salts to the precursor solutions. Fe-doped BiVO_4_ thin films can be obtained by adding Fe to the V precursor solution for treatment of a BiOI film prior to annealing.^158^ With the addition of a CoO_*x*_ catalyst to boost the conductivity and charge carrier density, Fe-BiVO_4_ photoanodes achieved 4.0 mA cm^–2^ photocurrent density at 1.23 V_RHE_ in a borate buffered electrolyte at pH 9.5. One atom % doping of Fe was found to be ideal, further Fe doping reduced the BiVO_4_ activity. The Fe-BiVO_4_ photocurrent remained stable at 2 mA cm^–2^ at 0.8 V_RHE_ for 11 h compared to 0.5 h for pristine BiVO_4_, and X-ray diffraction (XRD) and SEM analysis afterward showed little if any change to the morphology and structure of the photoanode. The improved performance and stability were attributed to a synergistic effect between the Fe doping and CoO_*x*_ catalyst.^158^ Finally, BiVO_4_ can also be extrinsically doped with H by placing the photoanode in an autoclave at 3 bar total pressure in a N_2_:H_2_ (2.0:1.0) mixed system for 10 min. Thermally dependent photocurrent measurements suggest the charge transport in BiVO_4_ is a polaron hopping mechanism and that the charge transport energy barrier is decreased with H-doping. H-Doping can occupy oxygen vacancies to suppress nonradiative recombination and facilitate polaron hopping, which was demonstrated by comparing electrolyte-side and substrate-side illumination of H-doped BiVO_4_ photoanodes (Figure 9c).^154^ Current density–voltage (*J*–*V*) measurements of pristine and H-doped BiVO_4_ were compared in 1 M KBi solution at pH 9.5 under 100 mW cm^–2^. The pristine BiVO_4_ had a back-illuminated photocurrent of 0.75 mA cm^–2^ the H-doped BiVO_4_ had a front-illuminated photocurrent density of 2.05 mA cm^–2^. H-Doping improved the photocurrent density and reversed the trend for front- and back-illuminated BiVO_4_ by increasing the overall mobility of electrons (Figure 9c).

Charge extraction from BiVO_4_ can alternatively be improved by formation of a junction, either by gradient doping of BiVO_4_ itself or by interfacing it with another semiconductor. Homojunction formation in BiVO_4_ was first demonstrated using spray pyrolysis. 200 nm thick n-type BiVO_4_ was prepared with varying dopant profiles that distributed a n^+^-n homojunction throughout the layer of by degeneratively doping the BiVO_4_ with W.^159^ Both 1% W-doped BiVO_4_|BiVO_4_ (where the BiVO_4_ was not intentionally doped) and gradient W-doped BiVO_4_ had improved charge carrier separation efficiency compared to 200 nm of 1% W-doped BiVO_4_. More recently, a similar BiVO_4_ homojunction was created by sequential electrodeposition from different electrolytes to vary the concentration of oxygen vacancies.^155^ A n–n^+^ BiVO_4_ photoanode was created by electrodepositing first from an acidic electrolyte and then from an alkaline electrolyte, creating a BiVO_4_ surface layer with more oxygen vacancies and therefore higher doping. The increased band bending in the oxygen vacant layer increased charge extraction to the electrolyte, achieving a photocurrent density of 3.6 mA cm^–2^ at 1.23 V_RHE_, three times the photocurrent of the single layer BiVO_4_ photoanode electrodeposited from the acidic electrolyte.^155^ Similar structures can be fabricated via extrinsic doping, such as by successive spin-coating of Zn- and W-doped BiVO_4_ layers.^160^

A heterojunction can be formed with the addition of another semiconductor, which can also act as a carrier-selective contact to improve charge extraction from the BiVO_4_. Interfacing electrodeposited BiVO_4_ with hydrothermally deposited NiMoO_4_ and adding cobalt phosphate (Co-Pi) improved the performance of the photoanode relative to a control sample, decreasing the OER onset by 180 mV and increasing photocurrent 5-fold, relative to pristine BiVO_4_, to 5.3 mA cm^–2^ at 1.23 V_RHE_ in 0.5 M Na_2_SO_4_ and 1 M Na_2_SO_3_.^153^ Mott–Schottky analysis indicated that BiVO_4_ and NiMoO_4_ form a heterojunction that facilitates separation of charge carriers. In another example, dropcasting copper acetate and vanadyl acetylacetonate in DMSO onto electrodeposited nanoporous BiOI and annealing at 450 °C for 2 h resulted in the formation of copper­(I) vanadate and trace copper­(II) vanadate on the surface of the resulting BiVO_4_ photoanode.^161^ Electrochemical impedance spectroscopy (EIS) analysis suggested incorporation of a low molar ratio of Cu (0.15:1 copper acetate:vanadyl acetylacetonate) improved interfacial charge transfer kinetics.^161^ At higher molar ratios (3.5:1 copper acetate:vanadyl acetylacetonate) of Cu, the vanadium source becomes limiting and leads to minimal formation of BiVO_4_. The excess Bi reacts with Cu^2+^ to form lower bandgap impurities like CuBi_2_O_4_, which decreases the band gap of BiVO_4_ to 2.16 eV. As in the other examples discussed here, the formation of a heterojunction provides improved charge carrier separation and conductivity, reducing recombination of carriers at the photoanode surface and improving overall performance.

Continued improvements and modification of the OER activity of BiVO_4_ push the material closer to its theoretical *j*_SC_ limit of 7.6 mA cm^–2^.^79^ Fundamental advancements in BiVO_4_ construction have allowed for additional freedom in its use as a photoelectrode, such as improvements in doping reversing the back-illuminated paradigm that dominated early work on this photoelectrode. Poor charge separation and electron mobility can be addressed by deliberate interfacial modification of BiVO_4_, which can improve both stability and catalysis by effectively directing charge carriers to the electrolyte and minimizing charge pile up at the interface. Controlling the density of oxygen vacancies in the material could improve the activity by improving the charge transport kinetics within the bulk and at the semiconductor|electrolyte interface. These will likely remain fruitful areas for future development of BiVO_4_.

#### 2.1.3 Hematite (α-Fe_2_O_3_)

Hematite (α-Fe_2_O_3_) is a theoretically appealing semiconductor as a photoanode, with an indirect 2.1 eV bandgap^162^ that can absorb ∼30% of AM 1.5G,^40^ a deep valence band capable of photogenerating holes with a large OER overpotential (1.2–1.3 V),^163^ and stability in neutral and alkaline electrolytes.^164,165^ Synthesis of α-Fe_2_O_3_ is achieved by a range of different wet and dry physical and chemical deposition techniques,^164^ including growth of single crystals which has facilitated understanding of intrinsic physicochemical properties. However, the optoelectronic and surface chemical properties of α-Fe_2_O_3_, rapid charge carrier recombination, inefficient photogeneration yield, and poor intrinsic kinetics for oxygen evolution, inhibit its performance as a photoanode (see Figure 4a).^164,166^ Photoabsorption in α-Fe_2_O_3_ broadly occurs via indirect transitions,^167,168^ limiting practically achievable photogeneration yield. Utilization of photogenerated holes is further challenged by the exceptionally high resistivity and poor electronic properties of stoichiometric α-Fe_2_O_3_, although reasonable conductivity and mobility are achieved with doping.^89,164,169,170^ The origins of intrinsically poor charge transport properties are an active area of research, with the current consensus pointing toward surface polaron mediated recombination that is independent of defect and grain boundary density, and therefore intrinsic to α-Fe_2_O_3_.^171–173^ Even at high doping concentrations (∼10^20^ cm^–3^), photocurrent efficiency is limited by poor electronic properties, namely low charge carrier mobilities (0.1 cm^2^ V^–1^ s^–1^)^174^ and short hole diffusion lengths (2–4 nm).^162^ Strategies to enhance photocurrent by nanostructuring have been met with success, achieving *j*_SC_ of ca. 5 mA cm^–2^,^175–177^ although the intrinsically poor photogeneration yield remains a major impediment to the practical application of α-Fe_2_O_3_ as a photoanode.^178^ Focused reviews on α-Fe_2_O_3_ physicochemical properties and PEC performance can be found in the literature.^164,165,179,180^

Despite being a promising midgap semiconductor, the performance of α-Fe_2_O_3_ as a photoanode is limited by poor optoelectronic properties, with short hole lifetimes and diffusion lengths limiting collection of photogenerated charge carriers.^165^ While hole collection efficiencies can be increased by lowering film thickness (decreasing the required carrier diffusion length), this strategy comes at the cost of decreasing net absorption;^175,176^ absorption depths (inverse absorption coefficient) of ∼40 nm above the absorption edge (<590 nm) and ∼5 μm below.^167^ Nanostructuring^175,177,181,182^ and resonant structuring^176^ strategies overcome this challenge by depositing thin films of α-Fe_2_O_3_ onto roughened surfaces or depositing over surfaces with high reflectance (Figure 10a,b), simultaneously increasing absorption path length while decreasing required hole diffusion lengths.

**Figure 10:**
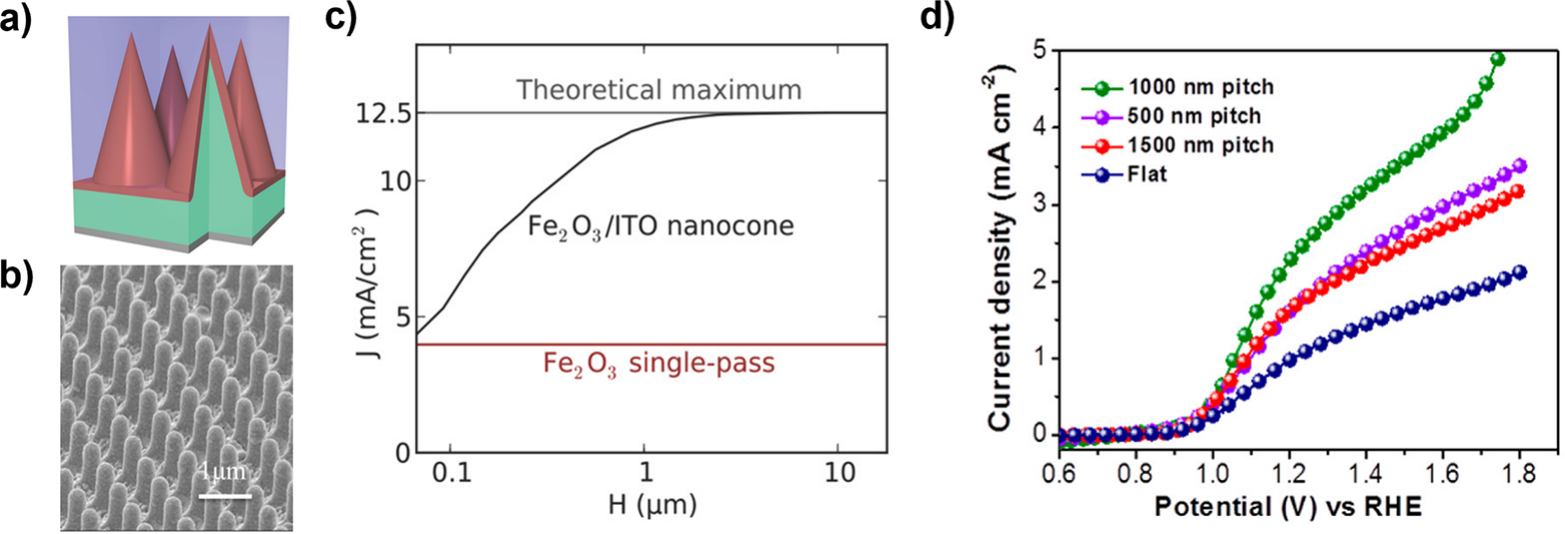
(a) Modeled and (b) patterned α-Fe_2_O_3_ nanostructures designed to enhance photoabsorption path length while minimizing required hole diffusion lengths. (c) Modeled results suggest conical architecture enhances photogeneration yield by decreasing reflectance and charge carrier diffusion length requirements. (d) However, empirical attempts to reproduce these efforts have yielded only moderate advances in α-Fe_2_O_3_ photocurrent. (a,c). Adapted with permission from ref 182. Copyright 2014 American Chemical Society. (b,d) Adapted with permission from ref 175. Copyright 2014 American Chemical Society.

Optoelectronic performance models predict the compromise between hole diffusion and absorption path length is optimized by tuning the aspect ratio of nanostructured α-Fe_2_O_3_ cones (Figure 10a,c).^175^ Increasing the height (Figure 10c) of cones synergistically orthogonalizes the directions of light absorption and charge carrier diffusion and decreases film reflectivity, allowing short hole diffusion and long absorbance pathlengths that approach the theoretical maximum 12.5 mA cm^–2^ predicted for oxygen evolution on α-Fe_2_O_3_ photoanodes. However, modeled results include an optimistic hole diffusion length (20 nm)^183^ and neglect the competition between sluggish oxygen evolution kinetics and rapid surface recombination. In contrast, experimental results for α-Fe_2_O_3_ with similarly engineered architectures (Figure 10b) are unable to exceed 5 mA cm^–2^ regardless of changes in nanorod periodicity (Figure 10d).^175^ Despite a roughly 2-fold increase in photocurrent density relative to a planar α-Fe_2_O_3_ photoelectrode, this result suggests intrinsic limitations exist in either optical or kinetic properties not accounted for in modeled results.

As discussed in section 1.2, quantum efficiency is the product of photogeneration yield and charge carrier collection efficiency. Systematic combinations of optical (ellipsometric) and PEC (quantum efficiency) measurements of α-Fe_2_O_3_ succeed in deconvoluting photogeneration yield and charge carrier collection efficiency (Figure 11a,b).^178,184^ This approach allows deconvolution of absorbance data into events which do and do not generate mobile electron–hole pairs that may contribute to Faradaic processes (i.e., OER; Figure 11b). The majority of noncontributing charge carriers correspond to low energy *d*–*d* transitions (i.e., spin-flip transitions), though a considerable fraction of higher energy ligand–metal transitions also do not contribute (Figure 11c).^167,168^

**Figure 11:**
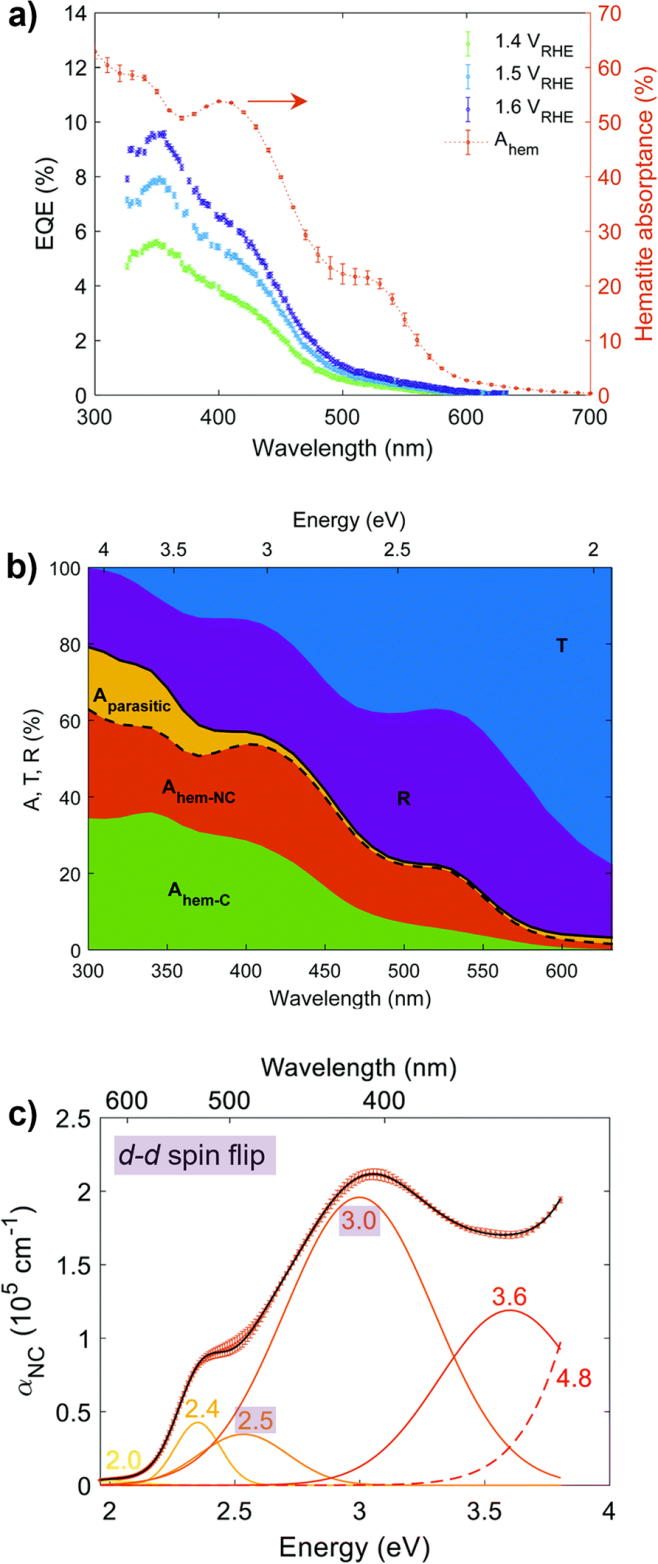
(a) External quantum efficiency (left axis) at 1.4 (green), 1.5 (blue) and 1.6 (purple) V_RHE_ and absorbance (right axis, orange) of a 32 nm 1% Sn:Fe_2_O_3_ thin film deposited over a 10% F:SnO_2_ substrate. (b) Deconvoluted transmission (T), reflectance (R) and absorbance (A) of the same Sn:Fe_2_O_3_ from (a). Absorbance is further deconvoluted into components that do (*A*_hem‑C_) and do not (*A*_hem‑NC_) contribute to the generation of electron–hole pairs and parasitic absorption (*A*_parasitic_) from the substrate or reflection at solid|solid interfaces. (c) Deconvolution of noncontributing absorption spectra (*A*_hem‑NC_ from (b)) illustrate how both *d*–*d* spin flip transitions (shaded purple) and ligand–metal transitions fail to generate separable electron–hole pairs.^167^ Figure adapted with permission from ref 178. Copyright 2021 Royal Society of Chemistry.

Poor photogeneration yield introduces a fundamental limitation to the maximum achievable photocurrent under AM 1.5G illumination, reducing the theoretically obtainable oxygen evolution photocurrent from ∼12.5 mA cm^–2^ to ca. 5.2 mA cm^–2^.^178^ Reframing achievable photocurrent within the fundamental limitations imposed by photogeneration yield, many examples of α-Fe_2_O_3_ approach ∼80% of the 5.2 mA cm^–2^ limitation under light-limited conditions.^175–177^ Development of spectroscopic techniques capable of discriminating the energy and relative cross sections of the manifold transitions of α-Fe_2_O_3_, coupled with computational calculations, may provide additional insight into the poor observed photogeneration yield.^185^ Application of a similarly thorough characterization of photogeneration yield for other metal oxides (e.g., TiO_2_, WO_3_, BiVO_4_) may provide a more accurate assessment of theoretically achievable *j*_SC_ and reframe the limitations of PEC performed by oxide photoelectrodes.

#### 2.1.4 Tungsten Oxide (WO_3_)

Reasonable electronic properties (polaronic hole mobility^186^ 10 cm^2^ V^–1^ s^–1^ and diffusion length 150 nm)^187^ and a deep valence band edge (3.1 V_RHE_) make tetragonal WO_3_ a promising photoanode candidate (Figure 6). As early investigators noted,^188^ however, the combination of instability in alkaline electrolytes^189,190^ and electrochromism (color change under applied potential)^191–193^ displayed by WO_3_ compromise its stability and controllable optoelectronic properties, both being crucial to photoelectrode performance. Synthetic approaches exist for a variety of both vacuum-based sublimation and deposition^191,193–198^ and wet chemical methods.^199–204^ As-synthesized WO_3_ is broadly transparent in the visible spectrum, with a sharp absorption edge occurring at ca. 335 nm.^193^ Ion (H^+^, Li^+^, Na^+^, K^+^) intercalation under either cathodic potentials (<−0.1 V_RHE_)^200^ or through synthesis (intentionally or otherwise)^205^ causes absorption to increase broadly across 500–2300 nm, accompanied by blue coloration.^193,206^ Intensity of X-ray photoelectron spectroscopy (XPS) core levels corresponding to intercalated ions increase in concert with growth of new electronic structure below the conduction band minimum.^191,193^ Similar observations are made in the absence of alkali ions, indicating formation of a hydride (i.e., H_*x*_WO_3_).^192,193^ Electrochromic influence of band edge location may then explain the wide range of reported *E*_g_ (2.5–3.1 eV)^201^ and *E*_fb_ (0.19–0.57 V_SHE_ from Mott–Schottky analysis):^187,188^ as new electronic structure populates below the conduction band minimum, the flat band potential becomes more anodic and the *observed* band gap decreases.

While the electrochromic response of WO_3_ allows tunable absorption of visible photons, challenges remain in utilizing those that are absorbed. OER kinetics impose very large overpotential losses to photovoltage, with onset potential of sodium sulfite oxidation occurring several hundred mV less anodic than that of OER (Figure 12a).^207^ Nanostructured terraces (TW in Figure 12b, pink lines in Figure 12a) demonstrate less anodic onset potentials, and correspondingly higher photocurrents, than porous architectures (PW in Figure 12b, blue in Figure 12a). The characteristic length of terraces in Figure 12b are shorter than the reported hole diffusion length of WO_3_ (150 nm), suggesting either charge carrier collection efficiency or preferential exposure of more active facets. Comparable voltametric profiles in the presence of a hole scavenger (Na_2_SO_3_, dashed lines in Figure 12a) suggest charge carrier collection efficiencies are comparable and that preferential faceting is most likely.

**Figure 12:**
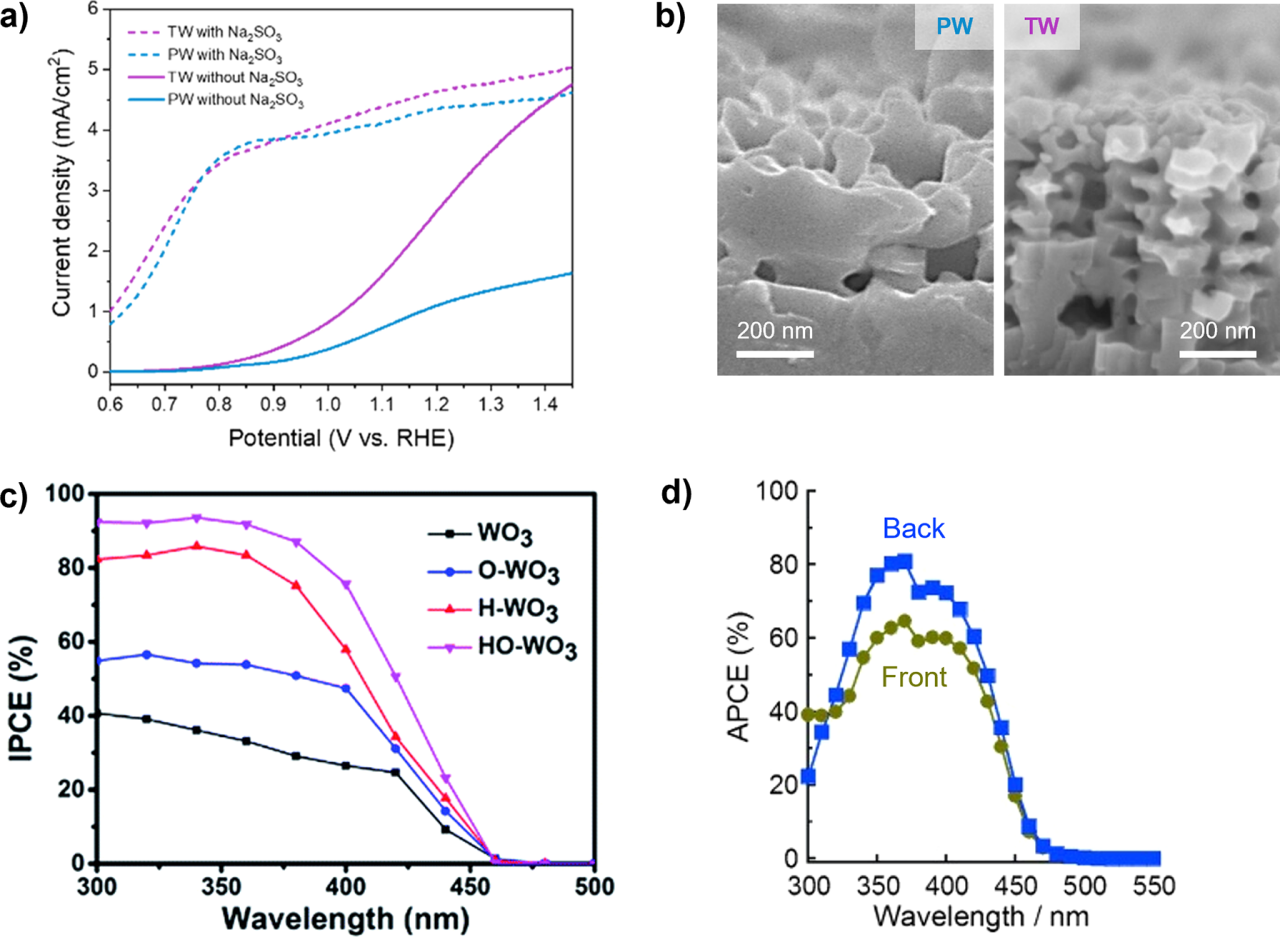
(a) Linear sweep voltammograms collected at 10 mV s^–1^ of terraced (TW, pink) and porous (PW, teal) WO_3_ in 0.5 M Na_2_SO_4_ (pH 7.2) with (dashed) and without (solid lines) 0.5 M sodium sulfite (Na_2_SO_3_) hole scavenger, illustrating the considerable efficiency losses of OER kinetics. (b) Cross-sectional SEM images of porous (PW) and terraced (TW) WO_3_ from (a). (a,b) Adapted with permission from ref 207. Copyright 2023 American Chemical Society. (c) Wavelength-dependent IPCE, measured in 0.1 M Na_2_SO_4_ at 1.23 V_RHE_, of WO_3_ as-synthesized (black squares), after ozone-treatment (O-WO_3_, blue circles), after hydrogen annealing (H-WO_3_, red triangles), and after sequential hydrogen annealing followed by ozone treatment (HO-WO_3_, pink inverted triangles). Adapted with permission from ref 202. Copyright 2018 Royal Society of Chemistry. (d) Wavelength-dependent absorbed photon-to-current efficiency (APCE) for front- (green) and back-side (blue) illuminated WO_3_, measured in Ar-sparged, 1 M H_2_SO_4_ at an unknown potential. Adapted with permission from ref 199. Copyright 2015 Wiley-VCH.

Analogous to the electrochromic behavior of WO_3_, introduction of oxygen vacancies by enhances both conductivity and visible absorption.^202,208^ One of the initial studies of annealing in hydrogen atmosphere improved the current density of WO_3_ at 1.0 V vs Ag/AgCl by an order of magnitude in a pH 6.8 electrolyte, a result of increasing electron-donating oxygen vacancies such that the net donor density increased by 3 orders of magnitude.^209^ However, oxygen vacancies also serve as sites for surface recombination.^202^ Ozone treatment and hydrogen reduction treatments both enhance IPCE relative to as-synthesized WO_3_ (Figure 12c).^202^ Combining hydrogen annealing followed by ozone treatment allows retains conductivity-enhancing bulk oxygen vacancies while removing them from the surface as recombination centers, yield enhanced IPCE (Figure 12c) and less anodic onset potentials.^202^ Backside illumination results in greater photocurrent and IPCE (Figure 12d),^199^ particularly for thicker films.^203^ This behavior suggests excessive recombination in either the bulk, due to poor electron mobility, or at the back contact interface, due to misaligned conduction bands. Similar observations are made on chalcogenide and CdTe thin films,^210,211^ lower interfacial band offsets yield greater photocurrents by facilitating interfacial transport of majority carriers. A similar design strategy may prove fruitful for WO_3_ photoelectrodes, where the conduction band minimum is tuned by electrochromic filling of donor states, resulting in an ill-defined conduction band minimum for photogenerated electron transport.

Although benchmark WO_3_ demonstrates 4.5 mA cm^–2^ under 100 mW cm^–2^ of AM1.5G illumination,^204^ there remains the need to better understand intrinsic optoelectronic properties, especially given the electrochromic nature of WO_3_. Greater photocurrents observed for back- than front-side illumination indicate limitations in majority charge carrier transport through the bulk or back contact, where intelligent design of back contacts is likely to enhance observed photocurrents.^210,211^ Similarly, OER kinetics barriers decrease photovoltage by several hundred mV with correspondingly deleterious effects on photocurrent (Figure 12a).^207^ Incorporation of appropriate catalysts, and careful management of interfacial defects, may drive a significant enhancement of PEC performance. While more controlled synthetic techniques exist (reactive sputtering,^197^ PLD,^194^ chemical vapor deposition^198^), little characterization has been performed on such high-quality films, perhaps explaining why quantification of intrinsic properties remains elusive.

#### 2.1.5 Cu-Based Oxides

Unusually for oxides, Cu-based oxide semiconductors are primarily candidate photocathodes, with many having intrinsic p-type doping and conduction band edges more negative than 0 V_RHE_ (Figure 13).^90,91^ Although the binaries CuO and Cu_2_O have reasonable bandgaps for water splitting, they are not stable, rapidly photocorroding due to self-reduction.^212^ This has led to work modifying and protecting the surfaces of the binary compounds, as well as searches for ternary copper oxides, where incorporation of another metal could modify the band gap and the crystal structure in hopes of inhibiting Cu reduction.^213^ The most-investigated ternary to date is CuBi_2_O_4_, although other ternary copper oxides are also discussed here, including some that are n-type for photoanodes. The negative conduction band edge positions of these Cu-based oxides have further led to interest in application of these semiconductors as photoelectrodes for the cathodic fuel-forming half-reactions discussed in section 4, where the presence of Cu has an additional catalytic benefit.^214,215^

**Figure 13:**
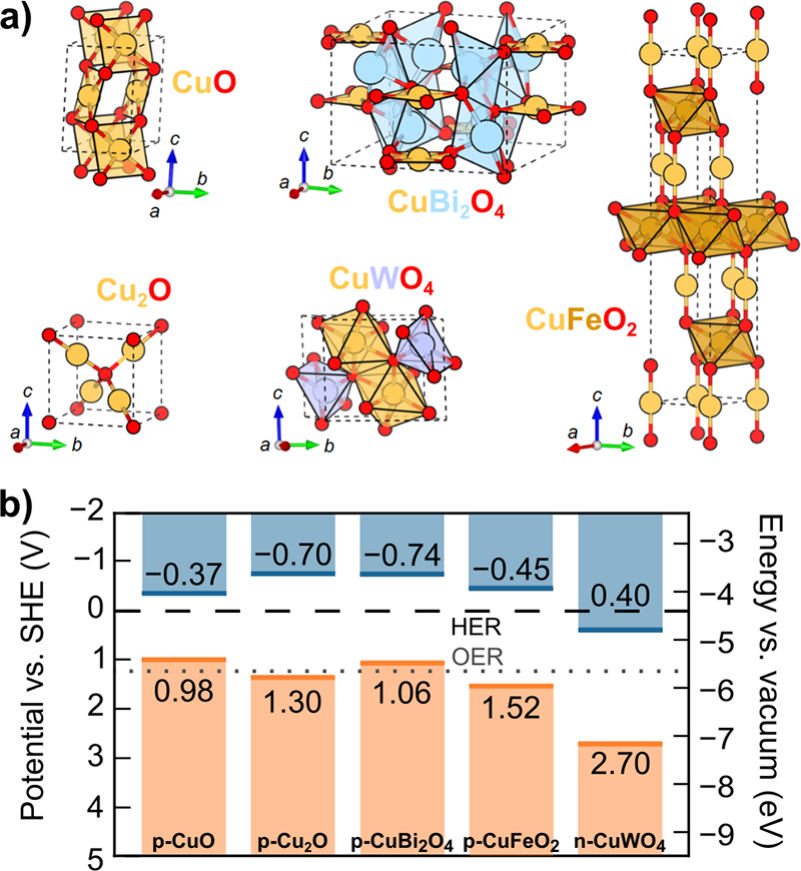
Selected Cu-based oxide semiconductors for photoelectrodes. (a) Unit cells of CuO, Cu_2_O, CuBi_2_O_4_, CuFeO_2_, and CuWO_4_. (b) Valence band edge maximum (orange line) and conduction band edge minimum (blue line) for each semiconductor with the thermodynamic potentials for HER (black dashed line) and OER (gray dotted line). See SI Tables S7 and S8 for details.

##### 2.1.5.1 Binary Copper Oxides (CuO and Cu_2_O)

CuO takes a monoclinic crystal structure with Cu^2+^ 4-fold bound and has a band gap near 1.5 eV; Cu_2_O has a cubic crystal structure with linear coordination on the Cu^1+^ and a band gap of 2.0 eV (Figure 13a,b).^90^ Both phases are long-known to be photoactive p-type semiconductors.^216,217^ However, the reduction potentials of both compounds lie within their respective bandgaps,^41^ leading to facile self-reduction from Cu^2+^ to Cu^1+^ and then to Cu^0^ with photogenerated electrons,^212^ which are often trapped at the surface due to high defect concentrations.^218,219^ The instability of these semiconductors impedes a robust reporting of their intrinsic kinetics for HER, and has led to the broad use of cocatalysts that impart some additional stability to the surfaces.^90^ These semiconductors can be synthesized by a wide range of methods with vacuum-based syntheses outperforming other strategies.^213,220,221^ The history of advances on PEC in both CuO and Cu_2_O has been well-reviewed;^90,91,220,222^ thus, we only briefly consider recent work on these semiconductors to inform the work on ternary compounds and tandem configurations.

The band energetics of CuO make for a promising photocathode, having a conduction band ca. 0.5 V_RHE_ negative of HER and *E*_fb_ of 0.9 V_RHE_.^216^ Despite these promising optical properties, early investigations found poor photocurrent and IPCE, citing recombination centers within the band gap as the reason for photocurrent onset 300 mV less than the flatband potential.^216^ Coupled with the addition of protective layers to protect against inherent instability,^223^ the literature has largely focused on enhancing electronic properties by mitigating synthetic defects. Annealing electrodeposited CuO in O_2_ enhanced photocurrent relative to similar samples either annealed in N_2_ or measured as deposited, though no change in photovoltage is observed.^224^ Photocurrent increases with increased bulk crystallinity (via annealing) and removal of possible oxygen vacancies (annealing in O_2_ vs N_2_), suggesting bulk recombination limits charge transport. Though the unchanged photovoltage suggests a surface-mediated process (i.e., CuO/Cu_2_O equilibrium) is responsible for intergap states and possible Fermi level pinning. Addition of a TiO_2_ protective layer and CdS heterojunction increases photocurrent from – 0.3 mA cm^–2^ for the base CuO to −1.4 mA cm^–2^ at 0 V_RHE_.^223^ Incorporation of Al doping and an Al:ZnO protective layer further enhances photocurrent to −5.25 mA cm^–2^ for CuO|Al:CuO|Al:ZnO|TiO_2_.^225^

Although it has a larger band gap (2.0 eV; Figure 14a) and thus lower theoretical η_STF_ (18%), cuprous oxide (Cu_2_O) has demonstrated greater photocathode performance than narrower band gap CuO.^220,226,227^ Bulk charge transport properties limit IPCE for both low (500–600 nm) and high energy (450–350 nm) incident photons (Figure 14b), where nanostructuring enhances IPCE.^227^ Similar to CuO, the instability of Cu_2_O prohibits long-term exposure to electrolyte, requiring protective layers to enhance stability. Intelligent design of photocathode architecture thus becomes critical to the extraction of charge for fuel forming reactions,^220,228^ lest excessive charge carrier recombination within the bulk and at the solid–solid interfaces with poor band alignment attenuate photocurrents. Improving band alignments for carrier-selective contacts remains an important area of research for Cu_2_O.

**Figure 14:**
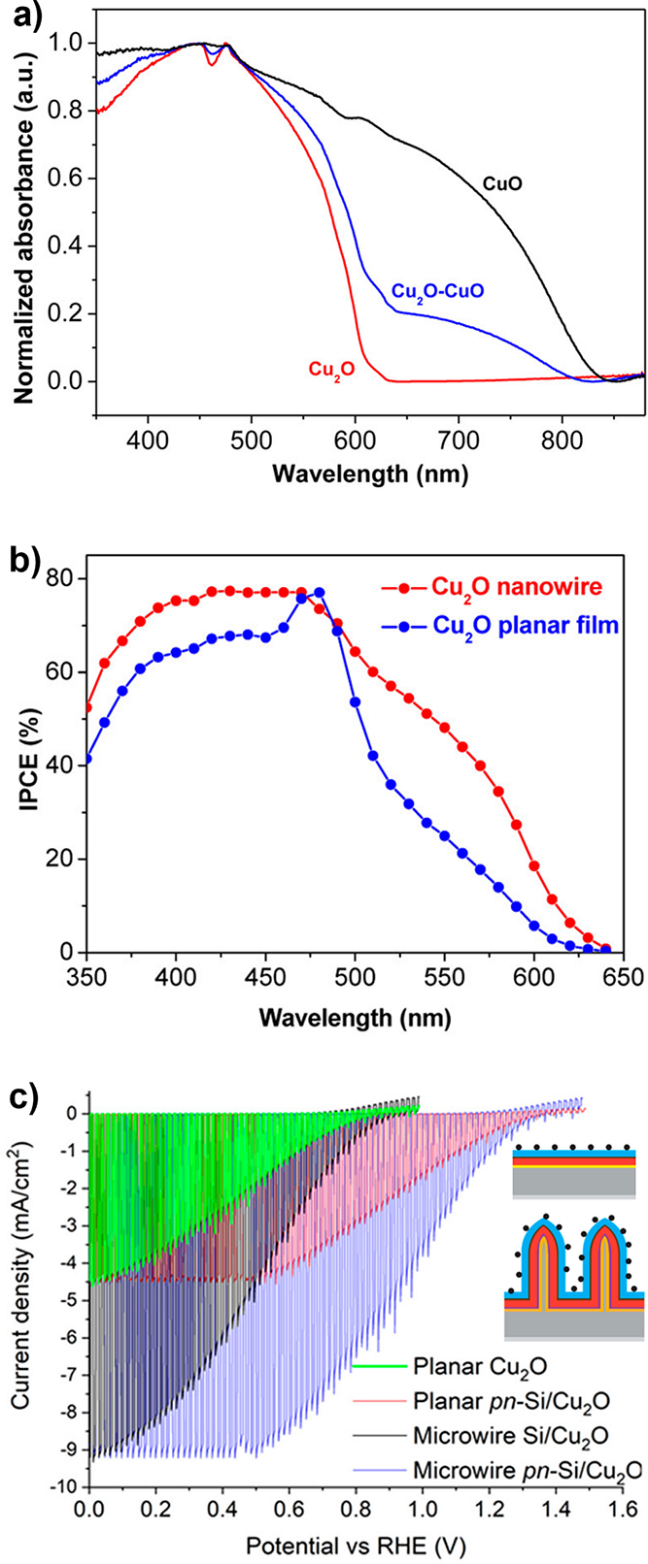
(a) Diffuse reflectance ultraviolet–visible absorption spectrum of CuO (black), Cu_2_O (red), and a mixture of both (Cu_2_O-CuO, blue). (b) IPCE at 0 V_RHE_ of Cu_2_O|TiO_2_|RuO_*x*_ with either planar (blue) or nanowire (red) morphology in 0.5 M Na_2_SO_4_ with 0.1 M KH_2_PO_4_ (pH 5.0). (a,b) Adapted with permission from ref 227. Copyright 2016 American Chemical Society. (c) Chopped AM 1.5G illuminated linear sweep voltammogram of planar Cu_2_O (green), planar pn-Si|Cu_2_O (red), Cu_2_O on a Si microwire substrate (black), and Cu_2_O on a pn-Si microwire substrate (blue). Voltammograms measured in 0.5 M Na_2_SO_4_ with 0.1 M Na_2_HPO_4_ (pH 5.0) at 10 mV s^–1^. Inset: illustrates photocathode architectures for planar (top) and microwire (bottom), where colors denote RuO_*x*_ (black circles), TiO_2_ (blue), Ga_2_O_3_ (brown), Cu_2_O (red), n-Si (orange), p-Si (gray), and an aluminum back contact (light gray). Adapted with permission from ref 228. Copyright 2019 American Chemical Society.

Despite these challenges, Cu_2_O photoabsorbers with carrier-selective heterojunctions produce some of the largest photovoltages of any photocathode,^228^ including producing nearly 3% η_STF_ when coupled with a Mo:BiVO_4_ photoanode to drive overall water splitting.^85^ The Cu_2_O|Ga_2_O_3_|TiO_2_|RuO_*x*_ structure used here provides larger photovoltages than when Ga_2_O_3_ is replaced with Al:ZnO. While the conduction band of Ga_2_O_3_ is 0.26 eV higher than that of Cu_2_O, providing a barrier to electron transfer, the large offset between Cu_2_O and Al:ZnO (−1.08 eV) provides a greater barrier to charge transfer that manifests in the poor observed photovoltages for Cu_2_O|Al:ZnO|TiO_2_|RuO_*x*_. Similar performance is observed when supporting Cu_2_O|n-Ga_2_O_3_|n-TiO_2_|RuO_*x*_ on microstructured pn-junction Si (Figure 14c).^228^ Microstructuring provides enhanced photocurrent (planar Cu_2_O vs microwire Si|Cu_2_O) and the underlying pn Si provides a photovoltage enhancement to Cu_2_O, resulting in V_ph_ exceeding 1.3 V_RHE_ for pn-Si|Cu_2_O|n-Ga_2_O_3_|n-TiO_2_|RuO_*x*_ microwires.

The offset absorption profiles of CuO (<840 nm) and Cu_2_O (<625 nm) suggest formation of CuO|Cu_2_O heterojunctions to absorb a wider range of light.^227^ Heterojunctions of Cu_2_O electrodeposited on FTO and annealed to form a CuO overlayer (FTO|Cu_2_O|CuO) indeed observe enhanced photocurrents when compared to either pure constituent (i.e., FTO|Cu_2_O or FTO|CuO), though stability is still limited to <1 h.^229^ Similar observations are made when controlling CuO thickness by varying air annealing times at 350 °C.^230^ Flat band potentials trend between behavior similar to Cu_2_O (0.63 V_RHE_) and CuO (1.1 V_RHE_) as the CuO nears complete coverage of the Cu_2_O substrate layer, while photocurrent at 0 V_RHE_ reaches a maximum with nominally complete CuO coverage (−1.2 mA cm^–2^, relative to −0.65 mA cm^–2^ with no CuO overlayer).^227,230^ Enhanced photovoltages and photocurrents may be obtained if the heterojunction can either be inverted (i.e., Cu_2_O atop CuO) or back illuminated, as the current architecture involves CuO, having a narrower band gap, sitting atop and attenuating incident photons for Cu_2_O.

##### 2.1.5.2 Copper Bismuthate (CuBiO_4_)

First identified as a candidate photocathode as part of a high-throughput combinatorial screening of Bibased oxides,^231^ CuBi_2_O_4_ takes the spinel^232^ crystal structure, with a band gap of approximately 1.8 eV^233,234^ that straddles the water reduction and oxidation potentials.^90^ Its very positive *E*_fb_ ∼ 1.26 V_RHE_, makes it a particularly interesting candidate photocathode,^233^ but like other oxides, CuBi_2_O_4_ suffers from polaronic charge transport and carrier recombination as well as slow HER kinetics.^234^ A study of nanoplatelet CuBi_2_O_4_ using electrochemical impedance spectroscopy revealed a large number of surface states that trap electrons and pin the electron quasi-Fermi level at the interface, slowing charge separation and requiring larger applied negative potentials in order to generate photocurrent.^232^ The stability of CuBi_2_O_4_ is a challenge for its use as a photocathode, which has been countered in some studies by incorporation of further metals into the lattice and by the use of protective layers or heterojunctions,^90^ although a range of approaches can improve this factor. Recent work focusing on controlling and improving optoelectronic properties has primarily focused on thin-film CuBi_2_O_4_ photoelectrodes synthesized by spin coating from nitrate precursors^235,236^ or using PLD,^237–239^ often with postannealing to increase grain sizes. There is also some interest in the development of CuBi_2_O_4_ for CO_2_RR,^237,240^ where the incorporation of Cu provides catalytic activity.

Initial work found that relatively short carrier lifetimes and diffusion rates were limiting factors in the PEC performance of drop-cast CuBi_2_O_4_,^233^ making these factors prime targets for improvement with controlled synthesis approaches. CuBi_2_O_4_ synthesis from nitrate precursors in an evaporation-decomposition-controlled method with a preanneal step (250 °C for 40 min) yields dense, single-phase films with ∼10^18^ cm^–3^ p-type carrier concentrations.^236^ The dense CuBi_2_O_4_ films displayed improved photocurrent onset, IPCE, and lower dark current compared to porous films, indicating reduced carrier recombination, and maintained photocurrent density even in thick films, indicating improved charge transport and carrier lifetimes (Figure 15a–c).^236^ Similarly, rapid thermal processing (RTP) of PLD Bi_2_O_3_ and CuO layers at 650 °C yielded dense CuBi_2_O_4_ films with a slightly wider band gap than CuBi_2_O_4_ deposited by spray pyrolysis and with similar photovoltage characteristics to a single crystal CuBi_2_O_4_ sample.^238^ In this case, the photocurrent of the PLD phase-pure CuBi_2_O_4_ was lower than films containing secondary phases, attributed to the removal of a photoactive CuO impurity.^238^ Similar results were found for PLD films from a pure CuBi_2_O_4_ target, where RTP produced single-phase CuBi_2_O_4_ films with photocurrent densities that plateaued over 150 nm film thickness, indicating long carrier transport distances, with higher fill factors and IPCE compared to PLD films with secondary phases.^237^

**Figure 15:**
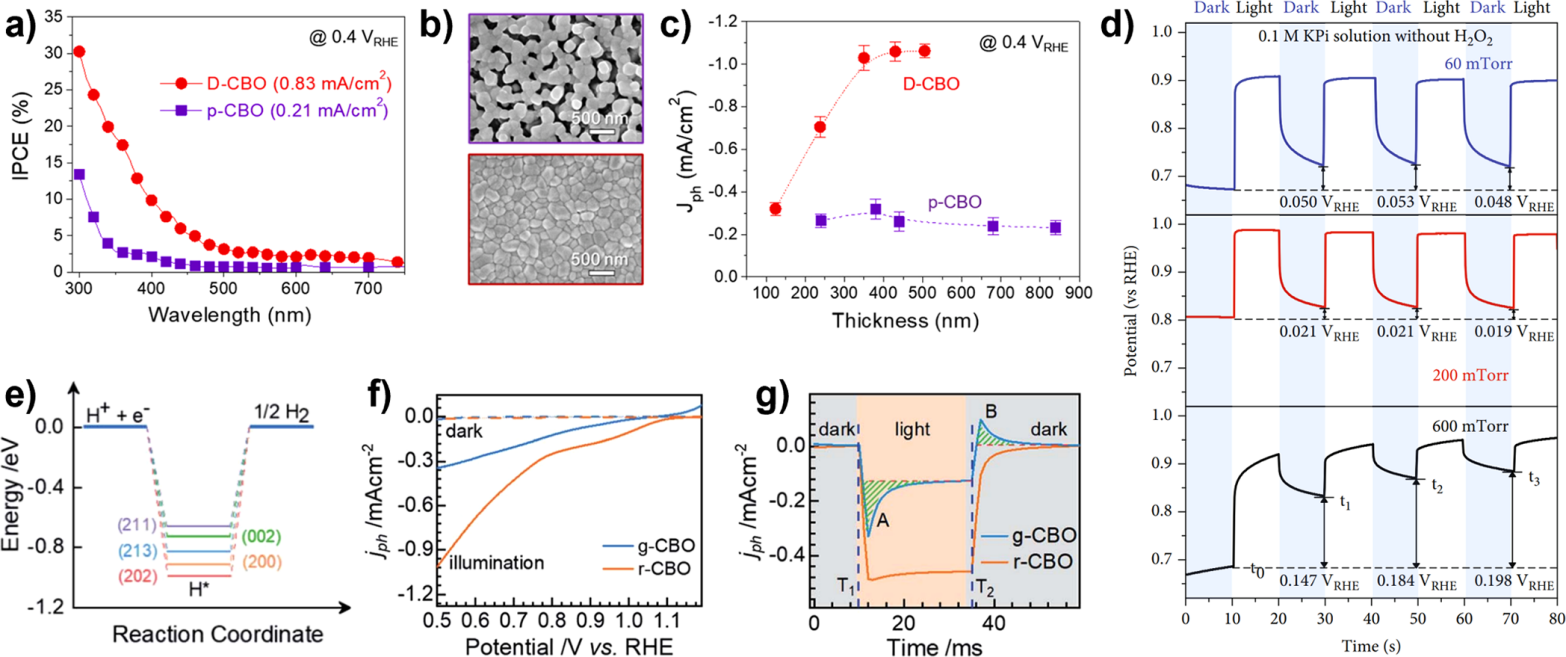
(a) Comparison of IPCE of dense CuBi_2_O_4_ (D-CBO, red) and porous CuBi_2_O_4_ (p-CBO, purple), showing improved photoabsorption of the dense material compared to the porous. (b) Top-down SEMs comparing the dense and porous morphologies from (a). (c) Change in current density at 0.4 V_RHE_ with increasing thickness of CuBi_2_O_4_ films synthesized by the two methods, with dense CuBi_2_O_4_ yielding improved photocurrent densities at higher film thicknesses due to improved carrier transport properties in that material. (a–c) Adapted with permission from ref 236. Copyright 2021 Elsevier. (d) Chopped illumination *V*_OC_ measurements of CuBi_2_O_4_ photocathodes synthesized by PLD under different oxygen partial pressures: 60 mTorr (blue trace), 200 mTorr (red trace), and 600 mTorr (black trace). Although the 600 mTorr sample had an oxygen contentration close to stoichiometry, it displays increasing dark current with charge trapping at its surface, while the 60 mTorr and 200 mTorr samples appear to have fast surface kinetics yielding stable and repeatable photovoltage generation. Adapted with permission from from ref 239. Copyright 2023 Wiley-VCH. (e) Reaction coordinate diagram of HER on CuBi_2_O_4_, showing the different binding energies of different surface terminations of CuBi_2_O_4_. (f) Comparative current–potential measurements of polycrystalline, grained CuBi_2_O_4_ (g-CBO, blue) and (002) facet-dominated, rod-like CuBi_2_O_4_ (r-CBO, orange); the highly oriented, rod-like material results in a higher photocurrent density and improved HER onset. (g) Transient photocurrent measurements of the same samples, showing carrier trapping in the polycrystalline g-CBO samples. (e–g) Adapted with permission from ref 234. Copyright 2022 Royal Society of Chemistry.

Modifications to stoichiometry have also been explored to improve the performance of CuBi_2_O_4_ photocathodes. Polycrystalline CuBi_2_O_4_ thin films were synthesized with PLD under a range of O_2_ partial pressures to deliberately generate Cu off-stoichiometry.^239^ Interestingly, the stoichiometric CuBi_2_O_4_ synthesized with the highest partial pressure of O_2_ showed substantial trapping of photogenerated charge carriers under chopped illumination, while a lower partial pressure of O_2_ showed no such trapping and stabilized the photovoltage of the CuBi_2_O_4_ (Figure 15d). This led the authors to conclude that a slight Cu deficiency (∼3% less than the expected concentration) enhanced the concentration of Cu^2+^ sites (confirmed by XPS), which in turn stabilized the CuBi_2_O_4_ in the dark, possibly by shifting *E*_F_ away from trapping photogenerated electrons.^239^ In another study, the addition of ∼7% Mg in a sol–gel-type CuBi_2_O_4_ synthesis improved the photocurrent and onset potential of HER without phase segregation in the photoelectrode.^241^ The Mg-CuBi_2_O_4_ samples had decreased lifetime of carriers trapped in surface states, indicating improved band bending at the electrolyte interface and a reduction in Fermi level pinning, and bulk carrier lifetimes were also longer. The quality was such that the Mg-CuBi_2_O_4_ photocathode could be coupled with a BiVO_4_ photoanode and successfully drive unbiased water splitting, albeit at ∼0.28 mA cm^–2^.^241^

While recent studies indicate that planar CuBi_2_O_4_ can be utilized as a photoelectrode without extensive nanostructuring to facilitate charge collection, deliberate nanostructuring to promote specific surface binding has also been shown to improve CuBi_2_O_4_ PEC performance. An investigation of CuBi_2_O_4_ surface energies using the computational hydrogen electrode (CHE) framework identified the (002) facet as the most stable surface, with nearly the highest HER activity (Figure 15e).^234^ Highly (002)-faceted CuBi_2_O_4_ nanowires were obtained using chemical bath deposition onto ITO, and transient photocurrent measurements indicated decreased electron accumulation at the electrolyte interface in the faceted CuBi_2_O_4_ as well as greatly improved IPCE, even with similar optical properties to polycrystalline material (Figure 15f,g).^234^ Systematic optimization of PEC-relevant properties of CuBi_2_O_4_ such as faceting may provide a route forward for the continued improvement of this semiconductor as a photocathode.

##### 2.1.5.3 Other Ternary Cu-Based Oxides

Delafossites (CuMO_2_) are a class of p-type metal oxides with tunable optical transitions ranging from 1.43 eV for CuFeO_2_ (Figure 16a for indirect, direct gap at 3.20 eV)^242^ to 2.12 eV for CuCrO_2_ (indirect),^243^ theoretically capable of facilitating up to 25 mA cm^–2^ photocurrent under AM 1.5G illumination as photocathodes.^242^ *E*_fb_ of ca. 1.0 V_RHE_ further illustrate appropriate photocathode band alignment and suggest possibly promising photovoltage generation.^244^ However, poor catalytic performance and optoelectronic properties limit cathodic photocurrents to <20% of this theoretical maximum, even in the presence of a sacrificial electron scavenger.^244^ Predominantly forbidden (*d-d*) optical transitions suggest limitations to the photogeneration yield of delafossite photocathodes (Figure 16a,b), similar to observations made for α-Fe_2_O_3_ photoanodes. Cathodic dark current, indicative of self-reduction, occurs at potentials <0.5 V_RHE_, with decomposition to metallic Cu and magnetite (Fe_3_O_4_) observed.^242,243,245^ Photocurrents are thus often reported at 0.4 or 0.5 V_RHE_, well above the theoretical equilibrium potential for water reduction.

**Figure 16:**
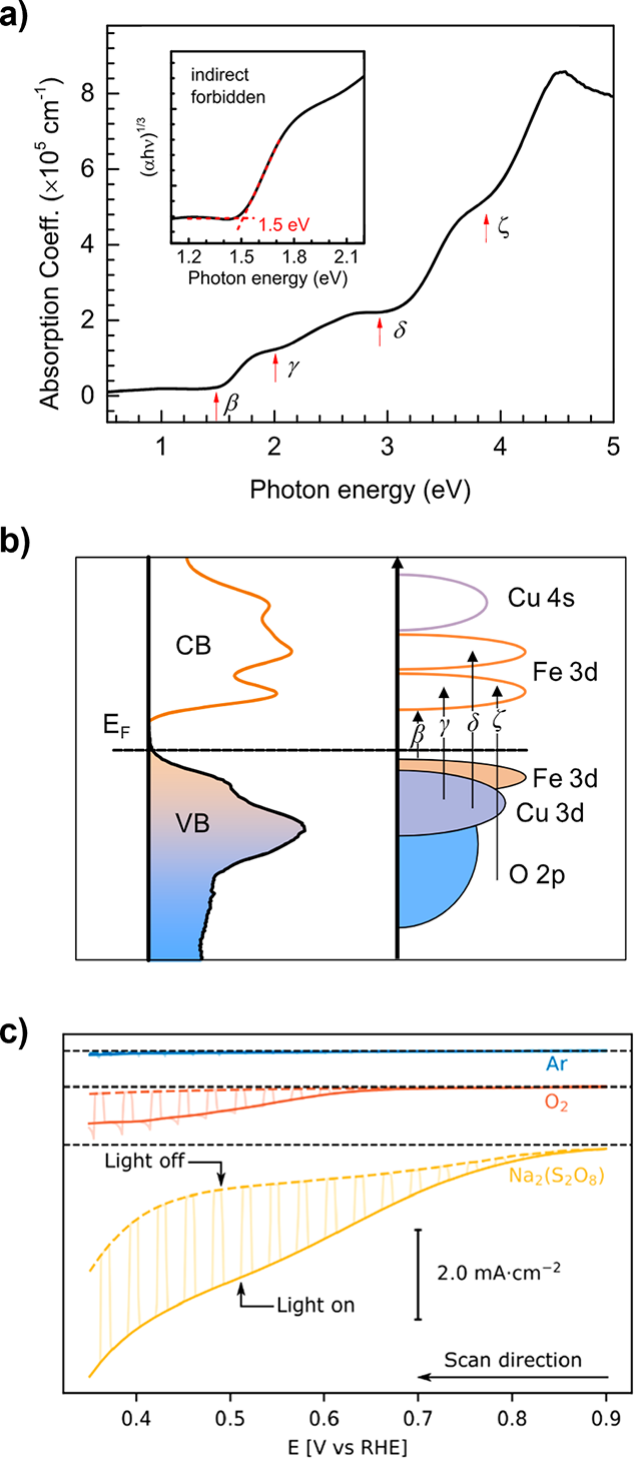
(a) Absorption coefficient of ∼100 nm polycrystalline thin film of CuFeO_2_ grown on Al_2_O_3_ (0001), with inset Tauc plot identifying the indirect forbidden band gap at 1.5 eV. Electronic transitions, denoted by Greek characters, are then (b) characterized by X-ray emission (XES), absorption (XAS), and photoelectron spectroscopy (XPS), identifying manifold dipole forbidden *d*–*d* (β, γ, δ) and allowed O 2*p*-TM 3*d* (ζ) transitions. (a,b) Adapted with permission from ref 246. Copyright 2021 American Chemical Society. (c) Linear sweep voltammograms under chopped AM 1.5G illumination in 1 M NaOH with Ar sparging (blue), O_2_ sparging (red), and 0.5 M sodium sulfamate (Na_2_(S_2_O_8_); orange). Voltammograms collected at a sweep rate of 10 mV s^–1^. Adapted with permission from ref 244. Copyright 2021 American Chemical Society.

Despite nominally large absorptivity (>10^5^ cm^–1^ for λ > 600 nm), IPCE is poor for wavelengths above the direct band gap, suggesting the optoelectronics of CuFeO_2_ impede photogenerated charge carrier transport to the electrode|electrolyte interface.^242,245,247^ Similar to α-Fe_2_O_3_, indirect photoabsorption in CuFeO_2_ occurs by forbidden *d*–*d* transitions (Figure 16b).^242,246^ Surface, and to a lesser extent bulk, charge recombination appear to severely inhibit photocurrents measured at 0.4 V_RHE_ in 1 M NaOH sparged with Ar (−0.06 mA cm^–2^), O_2_ (0.85 mA cm^–2^, likely mass-transfer limited), or Ar with addition of 0.5 M Na_2_S_2_O_8_ as sacrificial electron scavenger (2.5 mA cm^–2^; (Figure 16c)).^244^ While the enticingly intermediate band gap of delafossites suggests promising candidates for earth-abundant metal oxide photocathodes, the consistently poor reported photocurrents suggest bulk and surface recombination hinder application, or, worse, that poor photogeneration yield of indirect *d–d* transitions fundamentally limit the theoretically obtainable photocurrent density.^178,184^

Copper tungstate (CuWO_4_) is a n-type ternary metal oxide with intermediate indirect (2.3–2.4 eV; forbidden Cu 3*d*–Cu 3*d* transition) and direct gaps (2.6–2.7 eV; O 2*p*–Cu 3*d*) affording a theoretical current density of 8.2 mA cm^–2^.^248,249^ Flat band potentials are measured at 0.3 V_RHE_,^249^ with a sufficiently deep valence band maximum (2.85 V_RHE_) for water oxidation. The electronic structure is analogous to that of WO_3_,^250^ with the exception of a slightly decreased band gap from Cu^2+^ intergap states lying just above the O 2*p* valence band states.^249,251^ Photoanodes are chemically stable at neutral pH in unbuffered electrolytes (e.g., pH 7 potassium borate, well removed from its p*K*_a_ = 9.14), but degrade significantly in buffered phosphate solutions of the same pH (p*K*_a_ = 7.21).^251^ Application of anodic currents in unbuffered media decreases pH at the electrode–electrolyte interface,^252–254^ stabilizing CuWO_4_ in more acidic media similar to WO_3_. A recent review provides a more focused optoelectronic properties and recent progress on this semiconductor.^248^

While chemical stability in neutral electrolytes and an intermediate band gap suggest promising photoanode performance, charge transport limitations impede generation of appreciable photocurrents, with current benchmarks achieving <1 mA cm^–2^ at 1.23 V_RHE_.^255^ Mobilities on the order of 10^–3^ cm^2^ V^–1^ s^–1^ are reported for chemical vapor deposition (CVD) grown thin films,^256^ with temperature dependent mobility (increasing with temperature) indicating a polaronic conduction mechanism.^257^ While charge separation efficiency is well below unity for the surface, bulk recombination dominates efficiency losses.^255,258^ Photocurrent and IPCE are greater for backside illumination than for front (i.e., through electrolyte), indicating electron transport in the bulk, or perhaps even at the back-contact|CuWO_4_ interface, is primarily responsible for limiting photocurrent.^255,256,258,259^ Characterizing bulk charge carrier recombination centers, and identifying strategies to limit their formation, may yield enhanced photocurrents, where current benchmarks have only achieved ∼ 10% of the theoretical maximum.

#### 2.1.6 Emerging Oxides

The robust synthesizability of oxide semiconductors and their overall stability in oxidative electrochemical environments has resulted in a wide range of new metal oxides being proposed for photoelectrode applications. These emerging oxides are grouped below by crystal structure, as the synthetic flexibility of oxides enables a broad range of metal chemistries. Although there are many examples of such new oxides, some of which have been explored for electrochemical OER, there is limited depth of investigation into the PEC structure–property relationships for most individual semiconductors. Borrowing from systematic electrochemical studies elucidating structure–property relationships speed further development of these semiconductors as photoelectrodes. Because of the recent emergence of many of these semiconductors, we focus below on their fundamental properties, commenting on stability, catalysis, and morphology when possible.

Similar to CuWO_4_, tin tungstate (SnWO_4_) is an n-type oxide with a crystal structure forming layers of face-sharing SnO_4_ tetrahedra and corner-sharing WO_3_ octahedra.^260^ With indirect *E*_g_ ∼1.9 eV and direct *E*_g_ ∼2.6 eV,^261^ theoretical *j*_SC_ approaches ∼17 mA cm^–2^.^260^ However, benchmark photocurrents of ∼1 mA cm^–2^ measured under AM 1.5G spectrum at 1.23 V_RHE_ highlight severe differences between theoretical and achieved photoelectrode performance.^262^ This is notably driven by the large separation between direct and indirect *E*_g_, causing light between 1.9 and 2.6 eV to be absorbed well into the bulk (>1 μm)^261^ and challenging carrier collection. Further hindering performance are large differences between *E*_fb_ (∼0 V_RHE_)^260^ and OER photocurrent onset potentials, the latter typically being ∼300 mV more anodic.^261^ While the optical properties of SnWO_4_ can be tuned by varying the chemical potential of oxygen during growth by reactive cosputtering,^263^ identifying and resolving sources of carrier recombination is likely to lead to greater performance outcomes.

Spinels (A^2+^B_2_^3+^O_4_) are either p- or n-type oxides where mixed tetrahedral (A^2+^ site) and octahedral (B_2_^3+^ site) symmetry provide wide compositional design space facilitating optical property engineering.^264–266^ Band gaps range from 1.6 (FeAl_2_O_4_) to 4.2 eV (ZnAl_2_O_4_),^265,267^ offering a broad range of theoretically achievable *j*_SC_. Benchmark n-type ZnFe_2_O_4_ nanowire photoanodes (*E*_g_ ∼ 2.0 eV) achieve 1.72 mA cm^–2^ at 1.23 V_RHE_,^268^ while p-type photocathodes remain limited to <1 mA cm^–2^.^269,270^ However, as with most p-type metal oxides, poor stability under the severely reducing conditions of PEC HER leads them to degrade quickly, as demonstrated by a study of CuCo_2_O_4_ and NiCo_2_O_4_.^270^

Similar to spinels, perovskite oxides (A^(3‑*x*)+^B^(3+x)*+*^O_3_) are highly tunable structures with octahedrally centered B sites and 12-fold coordinated A sites.^94^ Mixed valency in A^(3‑x)+^ composition (e.g., La^3+^/Sr^2+^) provides control over the valence state and electronic structure of B^(3+*x*)+^ cations (e.g., Fe^3+^/Fe^4+^).^271^ A-site Sr substitution oxidizes B-site Fe in La_1–*x*_Sr_*x*_FeO_3_, lowering *E*_g_ and increasing photovoltage.^271^ LaFeO_3_ has been used in a np heterojunction with Nb-doped SrTiO_3_ to drive OER, achieving photocurrents <1 mA cm^–2^.^272^ Considerable effort has been dedicated to understanding and linking electronic structure of well-controlled single crystalline surfaces to their dark OER performance,^61,88^ where this insight may provide a framework for furthering development of efficient photoelectrode architectures.

Ilmenites (ABO_3_) share the same stoichiometry as perovskites, but they instead form corundum-like alternating layers of face-sharing metal-centered oxygen octahedra.^273^ Band gaps decrease for the titanate series (ATiO_3_) as the A-site is substituted for 3*d* transition metals with increasing *d*-state occupation (A = Mn (3.1 eV) to Ni (2.18 eV)),^274^ with absorptivity increasing in a similar trend.^273^ While appreciable n-type photovoltages are observed, poor photocurrent (<50 μA cm^–2^) will need to be overcome for ilmenites to be utilized as photoanodes.

### 2.2 Nitrides

There are dramatically fewer known and synthesizable nitrides compared to oxides across all semiconductor classes and even fewer of interest for PEC fuel formation, although some compounds have recently been proposed.^275,276^ Nitrides are potentially interesting as photoelectrode semiconductors from a design perspective: because both oxygen and nitrogen primarily contribute to the valence band, substitution of nitrogen can change the energy of that band without affecting the conduction band edge, moving the valence band edge closer to OER and reducing the band gap (Figure 17).^277^ However, the ease of oxygen substitution on nitrogen sites can make nitride optoelectronic properties difficult to control, nitride surfaces are generally poorly catalytic, and nitrides are often easily degraded in aqueous conditions, with self-oxidation or -reduction potentials lying within the band gap and kinetically easier than fuel-forming reactions.^41,278^ The most-studied nitride photoelectrode materials are GaN and its related compounds; Ta_3_N_5_; and perovskite-structure oxynitrides. Like the oxide photoelectrodes described previously, these compounds span the range of photoelectrode development shown in Figure 5: GaN is a commercialized semiconductor and benefits greatly from the understanding of its synthesis and properties that has been developed in other fields, while the perovskite oxynitrides are just emerging as potential photoelectrodes. Ta_3_N_5_ is an interesting example in this context, as it has quickly been brought from a relatively unknown and poorly understood semiconductor to nearly reaching its theoretical maximum *j*_SC_ in only a few years.

**Figure 17:**
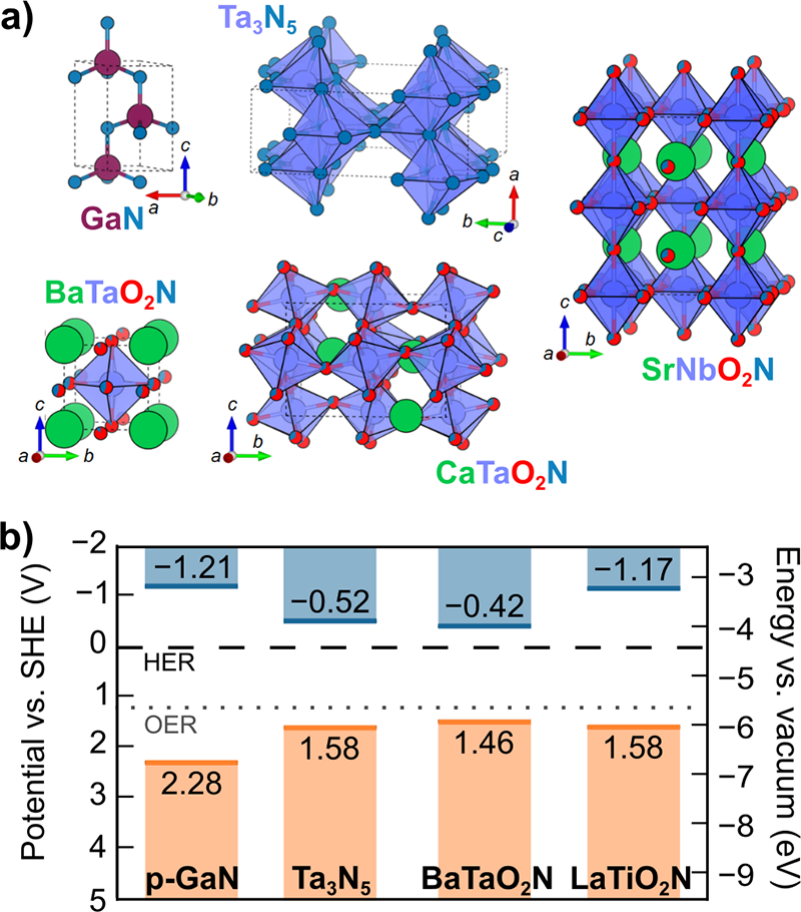
Selected nitride semiconductors for photoelectrodes. (a) Unit cells of GaN, Ta_3_N_5_, and three oxynitride perovskites (BaTaO_2_N, CaTaO_2_N, and SrNbO_2_N). (b) Valence band edge maximum (orange line) and conduction band edge minimum (blue line) for each semiconductor with the thermodynamic potentials for HER (black dashed line) and OER (gray dotted line). See SI Tables S7 and S8 for details.

#### 2.2.1 Gallium Nitride (GaN)

Like the semiconductors described later in section 2.4, GaN is well-developed for its primary use in light-emitting diodes. Due to that commercial use, the synthesis of GaN by MBE, metal–organic vapor phase epitaxy (MOVPE) and hydride vapor phase epitaxy (HVPE) is common, and thin-film single-crystal GaN is readily available. GaN can easily be n-doped over a range of concentrations, while p-type doping remains challenging and can result in lower crystal quality;^279^ however, GaN is more stable under the reductive conditions of HER than the oxidative conditions of OER, making its application as a n-type photoanode difficult.^280^ Although GaN absorbs little of the solar spectrum, its 3.4 eV band gap straddles the HER and OER potentials (Figure 17). The synthesis, properties, and defects of GaN are well-understood compared to its morphology for PEC, stability, and integration with catalysts, which are the major areas of investigation for improving the performance of GaN photoelectrodes.

The primary remaining area of research for the fundamental chemical aspects of GaN for PEC is improving its optical absorption by alloying with InN. In_*x*_Ga_1–*x*_N can drive overall water splitting, but often has high concentrations of crystalline defects due to the large lattice parameter mismatch between GaN and InN that can act as recombination sites for charge carriers and contribute to photocorrosion.^281^ Several approaches have emerged for utilizing In_*x*_Ga_1–*x*_N in photoelectrodes. One strategy takes advantage of the higher stability of GaN during PEC, using n-In_0.065_Ga_0.935_N with p-GaN layers of varying thickness as photoanodes.^282^ Addition of the p-GaN layer improved the *V*_OC_ of the photoanodes and η_F_ toward hydrogen production. However, the photocurrent of the samples with p-GaN fell with increasing p-GaN thickness, which was attributed to light loss in the p-GaN layer due to defects created from the Mg doping (Figure 18a).^282^ Another approach is to synthesize nanowires, which can avoid some of the defects that emerge in the growth of thick In_*x*_Ga_1–*x*_N layers. One example is the growth of In_0.3_Ga_0.7_N nanowires directly on Si using chemical vapor deposition from metal sources and NH_3_.^283^ As photoanodes, the Si|In_30_Ga_70_N achieved a photocurrent density of 8.08 mA cm^–2^ at 0.5 V_RHE_ with an onset potential of −1.04 V_RHE_. At 0.5 V_RHE_, the applied-bias photon-to-current efficiency (ABPE) of the photoanode was 5.89%, a five-times enhancement compared to Si|GaN nanowires. After 3.5 h of PEC operation in 1 M NaOH, no change was observed in the In_0.3_Ga_0.7_N nanowires, suggesting that this is a viable route to improving light absorption without sacrificing stability in GaN-based photoanodes.

**Figure 18:**
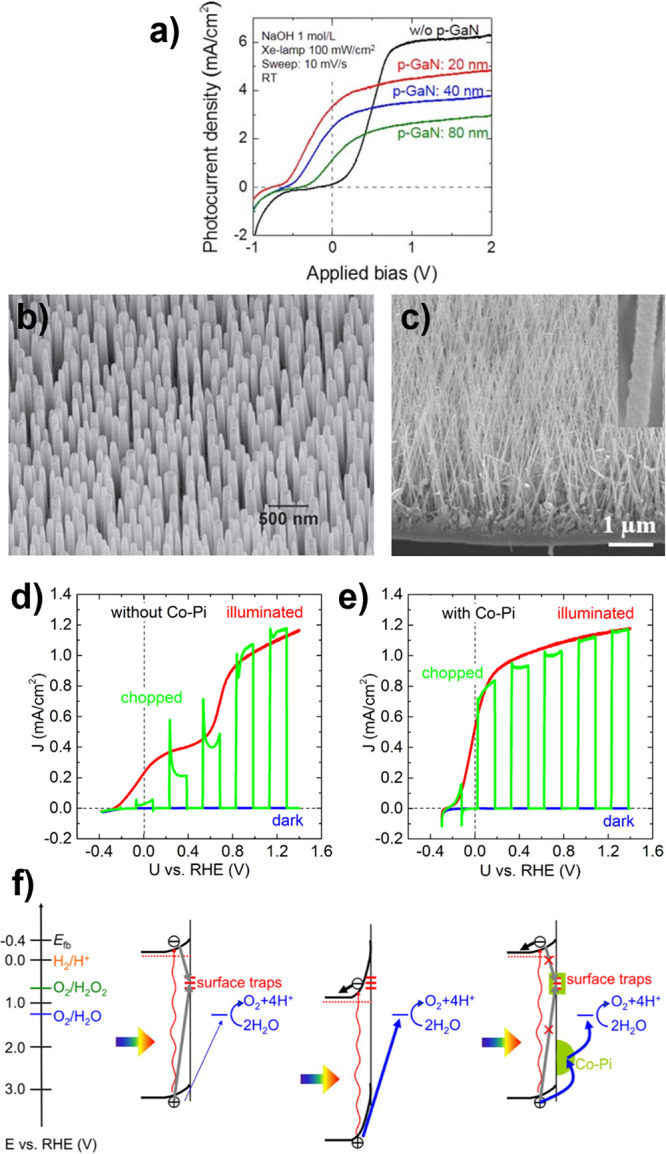
(a) Changes to photocurrent and photovoltage generated by a InGaN photoanode with increasing thickess of p-GaN (color traces) and without p-GaN (black trace). Adapted with permission from ref 282. Copyright 2019 IOP Publishing. (b) SEM image of top-down fabricated GaN nanopillars, fabricated by etching for 120s using 5 nm Ni particles as a mask. Adapted with permission from ref 284. Copyright 2017 IOP Publishing. (c) SEM image of bottom-up synthesized GaN nanowires, synthesized by vapor–liquid–solid MOCVD, with single nanowire inset. Adapted with permission from ref 285. Copyright 2021 American Chemical Society. (d,e) PEC characteristics of n-GaN photoanode without and with Co-Pi catalyst applied to the surface; the difference in behavior is explained by (f) band diagrams depicting the role of surface traps at low applied potentials in samples without catalyst. (d–f) Adapted with permission from from ref 286. Copyright 2017 American Chemical Society.

As with many of the wide *E*_g_ semiconductors described previously, nanostructuring GaN is a common strategy to improve light absorption and charge extraction in a photoelectrode, although different synthesis methods for nanostructures present different challenges in controlling doping and morphology. Top-down synthesis by dry etching of a metal–organic chemical vapor deposition (MOCVD) grown GaN layer enables nanopillar arrays with highly controlled dopant densities, where the diameter and length of the nanopillars can be controlled by tuning the size of Ni nanoparticles used as an etching mask (Figure 18b).^284^ When used as photoanodes, the photocurrent of the nanopillar arrays was approximately doubled from their planar counterparts, driven by enhanced optical absorption due to decreased reflection and a larger semiconductor|electrolyte interface for charge extraction, with longer nanopillars of smaller diameter providing the most benefit. Coupled with a NiO catalyst to increase hole extraction, these nanopillars were stable under OER conditions for 2 h at ∼0.75 mA cm^–2^.^284^ Alternatively, GaN nanowires can be grown by bottom-up methods such as Au-catalyzed vapor–liquid–solid MOCVD (Figure 18c). This can yield very long nanowires in dense arrays that can be employed as photoanodes, particularly with the application of plasmonic Au nanoparticles, which were shown to increase the photocurrent of the bottom-up GaN nanowires by 55%, up to nearly 1 mA cm^–2^.^285^

Extraction of charges to the electrolyte and prevention of surface recombination are major challenges for all GaN-based photoelectrodes, particularly when high surface areas are used to increase light absorption. GaN can be passivated by other semiconductors, noncatalytically active treatments, or catalysts. The same bottom-up method of fabricating GaN nanowires by Au-catalyzed growth was later expanded with the addition of ZnS passivation layers by atomic layer deposition (ALD), which improved charge extraction to the electrolyte. The resulting GaN|Au|ZnS photoanodes achieved a photocurrent density of 1.15 mA cm^–2^ and operated for 14 h with a 76% photocurrent retention at zero applied bias.^287^ This strategy can also be applied to planar GaN photoanodes by simply creating a p–n junction using MOCVD.^288^ Compared to n-GaN, the p–n GaN photoanode had improved PEC performance, with photocurrent onset −0.2 V_RHE_ and 0.16 mA cm^–2^ at 1.23 V_RHE_, while the n-GaN only achieved 0.10 mA cm^–2^ at that potential. The addition of a NiO_*x*_ catalyst slightly improved the photocurrent onset and improved the stability of the photoelectrode, but the largest benefit to performance came from an order of magnitude decrease in the charge transfer resistance at the semiconductor|electrolyte junction from n-GaN to pn-GaN, as measured by EIS.^288^

Another route toward passivating GaN surfaces is chemical treatment where the treatment is not intended to act as a catalyst or additional photoabsorber in the final photoelectrode. Treating nanoporous GaN by etching in 3 M KOH at 50 °C for 1 min followed by soaking for 15 min in 0.5 M NH_4_Cl was shown to replace dangling Ga–O surface bonds with Ga–Cl bonds by XPS.^289^ This restructuring of surface bonds and filling of trap states improved the ABPE of a nanoporous GaN photoanode to ∼0.37%, compared to the 0.1% efficiency of an untreated planar GaN photoanode.^289^ Hydrogen plasma can similarly be used as a passivating treatment. Etching in hydrogen followed by thermal annealing improved the performance of GaN nanorod photocathodes with InGaN quantum wells, increasing the photocurrent to 5.0 mA cm^–2^ at 1.4 V_RHE_, 3.5 times higher than the pristine nanorods in 0.5 M H_2_SO_4_.^290^ In this case, photoluminescence (PL) indicated that hydrogen treatment passivated surface defects, based on the observed decrease in the yellow emissions related to the defect.

The application of catalysts to GaN-based photoelectrodes can passivate recombination sites and improve stability by slowing corrosion, in addition to catalyzing fuel-forming reactions. NiOOH films, produced by sol–gel NiO_*x*_ deposition followed by anodic treatment, improve interfacial charge carrier separation and reduce photocorrosion in planar n-GaN photocathodes while catalyzing OER.^291^ Conversion of the catalyst to the oxyhydroxide lowered the OER onset potential by 100 mV, enabling the photoanode to achieve a saturated photocurrent density at a lower applied potential than bare GaN or GaN|NiO_*x*_. The NiOOH film also served to reduce pitting and cavity formation in the GaN photoanode from minimizing hole accumulation in the GaN, which may have resulted from the formation of a pn junction between the n-GaN and the catalyst.^291^ A similar effect was observed for n-In_0.09_Ga_0.91_N with a Ni­(OH)_2_ catalyst deposited on top of a NiO seed layer. Photoanodes fully covered by NiO|Ni­(OH)_2_ had improved extraction of charge carriers compared to bare n-In_0.09_Ga_0.91_N, resulting in higher saturated photocurrent density and decreased photocorrosion.^292^

Not all catalysts impart stability to GaN: Co-Pi photodeposited on n-GaN improved the photoanode current density to 1.2 mA cm^–2^, three times that of pristine n-GaN, but did not prevent etching of the n-GaN surface over 1 h at 1.2 V_RHE_ in a neutral phosphate buffered electrolyte (Figure 18d–f).^286^ The Co-Pi appeared to passivate surface trap states on the n-GaN that compete with OER for use of photogenerated holes at low applied potentials, improving photocurrent, but its porous nature allowed photocorrosion to continue at the n-GaN–electrolyte interface. The decrease in interfacial recombination indicates that GaN photocorrosion in neutral electrolyte is driven by photogenerated electrons rather than by holes, indicating a route for decreasing photocorrosion by increasing the driving force against electron diffusion to the surface.^286^ Such a force could be provided by hot electron injection from plasmonic Au nanoparticles, which have been shown to increase the photocurrent density of n-GaN photoanodes for overall water splitting.^293^ Nanoparticles were formed on the n-GaN surface by depositing thin layers of Au of various thickness and then annealing at 450 °C. The Au nanoparticle-modified n-GaN achieved a η_STH_ of 1.14% due to extraction of hot electrons from Au nanoparticles and the field created by the surface plasmon resonance of the Au that helped reduce charge carrier recombination at the surface.^293^

#### 2.2.2 Tantalum Nitride (Ta_3_N_5_)

Tantalum nitride has a bixbyite crystal structure, with Ta^5+^ cations surrounded by distorted corner and edge sharing octahedra of N^3–^ anions (Figure 17a). Its ∼2.1 eV band gap has edges straddling both HER and OER, in principle enabling independent overall water splitting with a maximum current density of 12.9 mA cm^–2^.^294^ In practice, Ta_3_N_5_ is n-doped and despite many studies experimentally determining *E*_fb_ near 0 V_RHE_, the photocurrent onset for OER remains near 0.6 V_RHE_.^78^ Thus, Ta_3_N_5_ has been investigated as a semitransparent photoanode for OER that could be coupled with a photocathode to provide additional voltage and achieve overall water splitting. Ta_3_N_5_ has limited native catalytic activity and is nearly always coupled with a catalyst to facilitate OER. If its surface is left unprotected, Ta_3_N_5_ will rapidly photocorrode to form insulating TaO_*x*_, as its self-oxidation potential lies within the band gap.^41^ Even with these issues, Ta_3_N_5_ stands out as an exemplary photoelectrode material because of its recent rapid development, which broadly follows the path laid out in Figure 5. Ta_3_N_5_ photoanodes have reached 10.96 mA cm^–2^ at 1.23 V_RHE_ ^84^ in the span of only a few years, a result of targeted and systematic work addressing synthesis, defects, catalyst integration, and nanostructuring.

Ta_3_N_5_ is primarily synthesized by nitridation of Ta or TaO_*x*_ via NH_3_ flow at high temperatures for at least several hours. Standard conductive substrates such as FTO and ITO degrade in these conditions,^295^ leading to the use of alternative substrates. Synthesis of Ta_3_N_5_ directly on Ta foil by oxidation and subsequent nitridation under NH_3_ flow is straightforward and provides a highly conductive, intimate contact layer for the Ta_3_N_5_ photoelectrodes.^296^ Dopant elements can be added as fluxes at the oxide stage for eventual incorporation into the nitride.^297^ Thin films of Ta or Ta_2_O_5_ can also be deposited by PVD methods and subsequently nitridated on diverse substrates including Nb foil, GaN, and even quartz (as the Ta_3_N_5_ product can be conductive enough to act as a photoelectrode without a conductive substrate, Figure 19b).^298–300^ In the case of Ta_2_O_5_ deposition, dopants can be added to the film in their oxide forms via cosputtering prior to nitridation.^301^ Conversion to Ta_3_N_5_ from an oxide is accompanied by a reduction in lattice volume that can result in voids and films that are less than fully dense (Figure 19c).^300^

**Figure 19:**
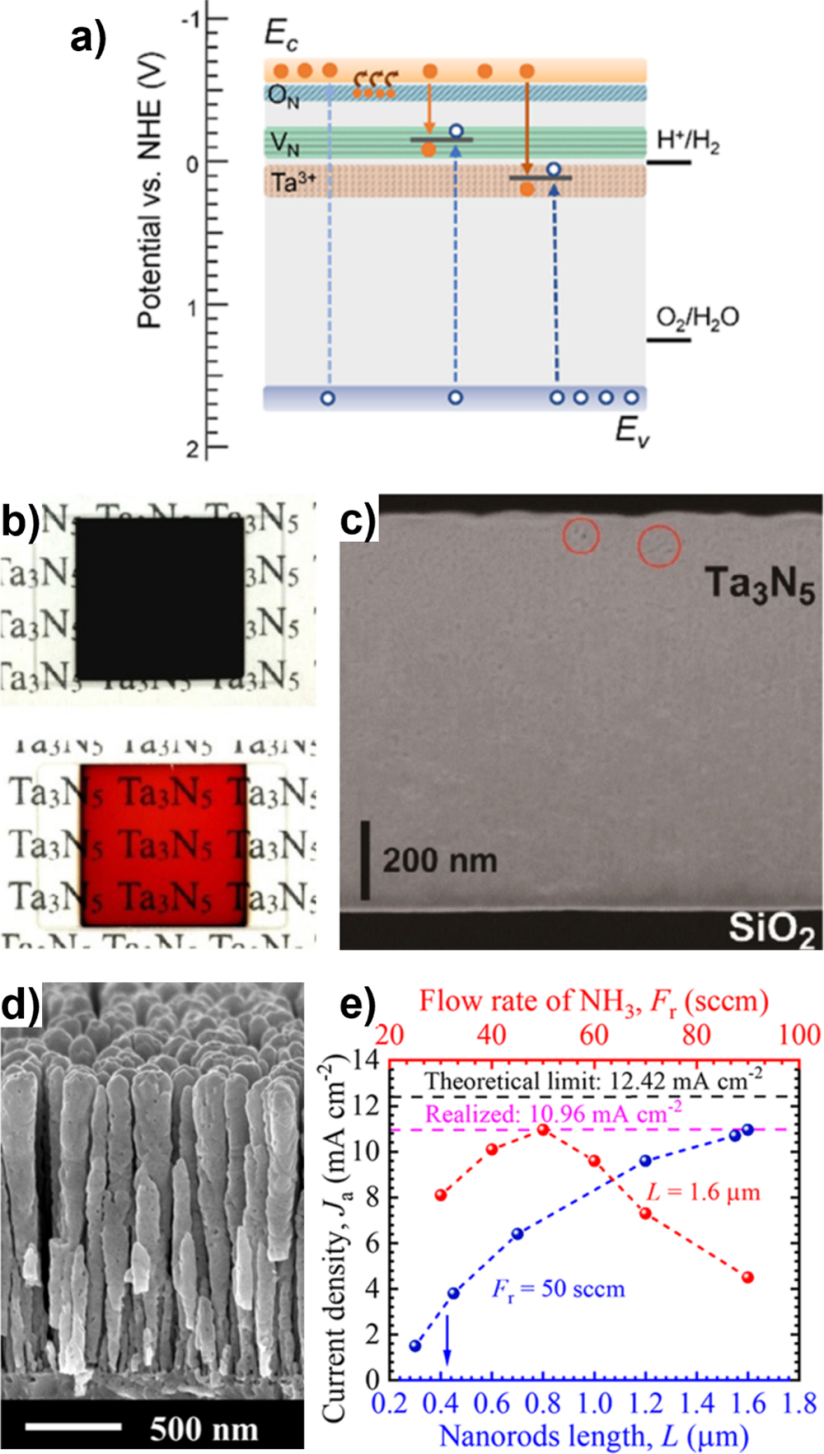
(a) Diagram of charge-trapping defect levels for O_N_, V_N_, and Ta^3+^ in the Ta_3_N_5_ band gap, with the redox potentials for HER and OER given for reference. Adapted with permission from ref 300. Copyright 2020 American Chemical Society. (b) Photographs of a metallic Ta precursor (top, black) and annealed Ta_3_N_5_ film (bottom, red) on SiO_2_ substrate. (c) Cross-sectional dark-field scanning transmission electron microscopy (STEM) image of Ta_3_N_5_ thin film following annealing, with red circles indicating voids in the film resulting from the volume change during annealing. (b,c) Adapted with permission from ref 299. Copyright 2020 Wiley-VCH. (d) Ta_3_N_5_ nanorods with length *L* = 1.6 μm. (e) Comparison of observed current densities toward OER for varied Ta_3_N_5_ nanorod lengths and NH_3_ flow rates during synthesis. (d,e) Adapted with permission from ref 84. Copyright 2023 American Chemical Society.

Common defects in Ta_3_N_5_ are oxygen on nitrogen sites (O_N_), nitrogen vacancies (V_N_), and reduced Ta species. Such defects limit PEC performance and hinder identification of the true optoelectronic properties of the semiconductor. DFT calculations have shown that oxygen impurities are shallow donors (responsible for n-doping of Ta_3_N_5_ as in many emerging nitrides) while *V*_N_ and reduced Ta are considered deep traps (Figure 19a).^300^ Increasing NH_3_ flow rates at a single temperature has been shown to decrease the concentration of these primary defects, and also to decrease the density of voids as the incorporation of N^3–^ into the lattice improves. A high NH_3_ flow rate resulted in 7.3 mA cm^–2^ at 1.23 V_RHE_ under simulated AM 1.5G illumination from a Ta_3_N_5_ photoelectrode with a borate-intercalated mixed nickel cobalt iron oxyhydroxide catalyst, which was further improved to 8.2 mA cm^–2^ by etching the film with H_2_O_2_ to fully oxidize reduced Ta near the surface.^300^ To determine the real band gap of Ta_3_N_5_, one study nitridated very thin (∼60 nm) amorphous tantalum oxynitride films at increasing temperatures. Because the films were thin, nitridation eliminated O_N_ defects without generating voids. The low optical scattering of these films enabled use of photothermal deflection spectroscopy with Tauc analysis to determine that Ta_3_N_5_ has an indirect band gap of 2.18 eV. Ta_3_N_5_ often shows PL, which has hindered conclusive identification of the band gap as direct or indirect, but this work showed that the PL decreased with increasing annealing temperatures, and importantly, Ta_*x*_O_*y*_ content of the films, supporting the conclusion that the band gap is indirect.^302^ The correlated reduction of point (chemical) defects and crystalline (void) defects is a substantial benefit for Ta_3_N_5_ investigation, and will likely continue to benefit the development of this semiconductor into the future as its intrinsic properties are investigated without confounding defects.

Another strategy for limiting the deleterious effects of defects in Ta_3_N_5_ has been incorporation of dopant elements at high concentrations (>10 atom %). Some notable examples have been incorporation of Mg and La. Addition of ∼13% Mg in Ta_3_N_5_ thin films was found to decrease the concentrations of Ta in TaO_*x*_N_*y*_ and Ta^3+^ forms and to shift *E*_fb_ cathodically, possibly due to the introduction of O with the Mg.^301^ Gradient incorporation of the Mg was used to further improve PEC performance by matching and effectively reducing the gradient of native film defects.^301^ Surface incorporation of La also improves PEC performance, this time by effectively forming LaTaON_2_ and creating a heterojunction which enables better charge transport at that interface.^297^ While these approaches have been successful, they have not been the enablers of high performance of Ta_3_N_5_ photoanodes, in part because even small concentrations of O_N_ generally provide sufficient doping for charge transport. Further development of doping schemes, possibly at lower concentrations, may provide better control over the charge transport properties of Ta_3_N_5_.

The surface of Ta_3_N_5_ is easily oxidizes under PEC operation,^278^ causing Fermi level pinning that limits the photocurrent onset potential at low defect concentrations^300,303^ and shutting current flow off entirely in the case of Ta_*x*_O_*y*_ formation.^295,304^ Multiple strategies specifically target surface defects, including treatment with H_2_O_2_,^300^ passivation using pyridine,^296^ doping,^297,301^ and two-step flame annealing precursor films prior to nitridation,^305^ but these generally do not impart durable protection from oxidation. Deposition of a catalyst thin film, necessary due to the limited catalytic activity of Ta_3_N_5_, can also slow surface oxidation. Most OER studies on Ta_3_N_5_ are in alkaline environments, and Ni–Fe–Co oxyhydroxide-type catalysts are primarily used. One study illustrated the importance of catalyst continuity and uniformity by comparing drop- or spincast NiFeO_*x*_ catalysts, showing that nonuniform protection from the dropcast catalyst resulted in rapid loss of photoanode performance due to oxidation, such that the Ta_3_N_5_ degraded after ∼30 min, rather than the ∼90 min of stability afforded by uniform catalyst deposition.^295^ Modeling of Ta_3_N_5_ photocurrent degradation has shown that photocurrent loss can occur even with uniform catalyst loading intended to prevent self-oxidation, a result of oxide growth driven by the local electric field during PEC operation.^278^ Although most Ta_3_N_5_ studies have focused on alkaline conditions, IrO_*x*_ has been studied for use as an OER catalyst in acidic and neutral conditions; spin-cast IrO_*x*_ was found to enable facile charge transfer from the Ta_3_N_5_ and protect the surface for up to 4 h, although self-oxidation still ultimately occurred.^304^ Despite the fact that Ni–Fe–Co oxyhydroxide catalysts are popularly used on Ta_3_N_5_, there does not appear to be substantial work confirming that this is an optimal pairing; detailed investigation might allow for a more rational catalyst selection.

While unintentional nanostructuring in the form of voids has been shown to have a negative effect on Ta_3_N_5_ photoanodes, deliberate surface structuring is an active area of research for this semiconductor as it is for many others, beneficially increasing surface area and often, charge carrier collection. One strategy for creating nanostructured Ta_3_N_5_ *relies* on the oxide-to-nitride lattice volume contraction, nitridating KTaO_3_ single-crystals in one or two steps to generate porous Ta_3_N_5_ monoliths.^306,307^ The resulting structures are still single-crystalline with few grain boundaries and retain the orientation of the parent crystal, enabling facet-dependent property studies with low carrier recombination at crystalline defects. Ta_3_N_5_ can also be synthesized in the classic nanorod form: glancing angle deposition, where a substrate is held at a specific angle, enables reactive sputtering of Ta in an oxygen and nitrogen containing environment, yielding a TaO_*x*_N_*y*_ precursor which is then nitridated in the same manner as a Ta_3_N_5_ thin film.^84^ Deposition on Ta foil provides a conductive substrate in good contact with the resulting Ta_3_N_5_ nanorods. Long nanorods nitridated under an optimized NH_3_ flow (Figure 19d,e) provided high photocurrent densities, a result of improved light absorption, increase in carrier diffusion length, and reduced charge carrier recombination.^84^ Synthesis of nanorods by this method has recently been used in conjunction with the other strategies discussed here to produce a high-performing Ta_3_N_5_ photoelectrode with a Ta_3_N_5_ connecting layer on GaN used in tandem with dual CuInSe_2_ PVs for overall water-splitting.^308^ A two-step deposition process for a FeNiCoO_*x*_ catalyst enabled conformal deposition on the Ta_3_N_5_ nanorods and protected the Ta_3_N_5_ from oxidation for 6.7 h. Detailed simulations showed that ∼ 75% of generated charge carriers were extracted from the nanorods for water splitting, and the integrated photoanode/PV system achieved an unbiased η_STH_ of 12.1%.^308^

#### 2.2.3 Oxynitride Perovskites

Perovskite-structured oxynitrides with the generic formula AB­(O,N)_3_ have been targeted as semitransparent photoanodes due to their smaller band gaps and improved light absorption over many oxides (Figure 17), resulting from hybridization between O and N 2p orbitals.^309,310^ Within this chemically diverse group, LaTiO_2_N, BaTaO_2_N, SrNbO_2_N, and CaTaO_2_N have been widely studied. When operated as photoanodes, these semiconductors can have photocurrent onsets far negative of that of Ta_3_N_5_,^78,311^ but photocurrent densities are quite low. This has been attributed to short photogenerated carrier lifetimes^311^ and diffusion lengths,^312^ but routes to improving these properties have not yet been identified. At this point, there is insufficient understanding of the perovskite oxynitrides to determine if this class will become relevant for PEC OER the way that Ta_3_N_5_ has. Many oxynitride perovskite compounds are also actively being investigated for purely photocatalytic water splitting, although their catalytic action has been less of a focus of PEC investigations. The parallel development of both PEC and PC using these semiconductors presents unique opportunities for understanding changes to optoelectronic and catalytic properties resulting from varied synthetic approaches, compared to other candidate photoelectrode materials.

Thin film perovskite oxynitrides can be obtained by many nitride chemical or physical vapor deposition methods.^313,314^ PVD methods employing reactive gases are particularly effective, such as reactive sputtering of SrNbO_2_N from metallic targets in nitrogen and postannealing to control oxygen content^310^ or pulsed reactive crossed-beam laser ablation (PRCLA), a modified PLD technique where the injection of NH_3_ incorporates N during growth.^314^ An oxide containing the correct cations can also be synthesized from a sol–gel with subsequent ammonolysis to the oxynitride.^309,315,316^ Achieving anion stoichiometry in these quaternary compounds, however, can be difficult; some perovskite oxynitrides can form both oxygen- and nitrogen-rich phases (ABO_2_N vs ABON_2_),^310^ and realized anion contents are often off-stoichiometry or inadequately characterized. Changes in N content can dramatically change optical absorption properties, making characterization of anion stoichiometry critical to assessment of e.g. IPCE.^314^ Similarly, assessing intrinsic perovskite oxynitride performance is challenged when the semiconductors are grown on Ta-based substrates,^317^ which frequently grow semiconducting Ta_*x*_N_*y*_ and Ta_*x*_O_*y*_ secondary phases. Furthermore, different perovskite oxynitrides display varying degrees of tiling or long-range ordering (Figure 17a),^71,313^ which can change optoelectronic properties but is hard to assess in thin-film formats. Thus, while these semiconductors are synthesizable, further development is needed for property and defect characterization and control.

One recent study illustrated some of these issues by comparing photoanodes of LaTiO_*x*_N_*y*_, BaTaO_*x*_N_*y*_, and CaNbO_*x*_N_*y*_ synthesized as thin films by PRCLA and as particulates electrophoretically deposited on a conductive substrate (Figure 20a).^318^ The three synthesized compounds were chemically similar between the two methods, with the particulate synthesis yielding a higher nitrogen context for the LaTiO_*x*_N_*y*_ only (*y* = 0.8 for particle, 0.34 for thin film). Normalization by surface area and optical absorption, both of which favored the particulate photoelectrodes, revealed that the thin film samples had slightly higher photocurrent densities, although all materials had <1 μA cm^–2^ at 1.23 V_RHE_.^318^ The thin-film geometry also facilitated better comparisons between the perovskite oxynitrides, as the thin films of BaTaO_*x*_N_*y*_ and LaTiO_*x*_N_*y*_ had similar photocurrent densities as a result of a lack of grain boundaries that in the particulate films artificially deflated the BaTaO_*x*_N_*y*_ current density due to very small particle size.^318^ This work highlights the importance of comparing perovskite oxynitrides of similar N content, even for photoanodes generated by the same method.

**Figure 20:**
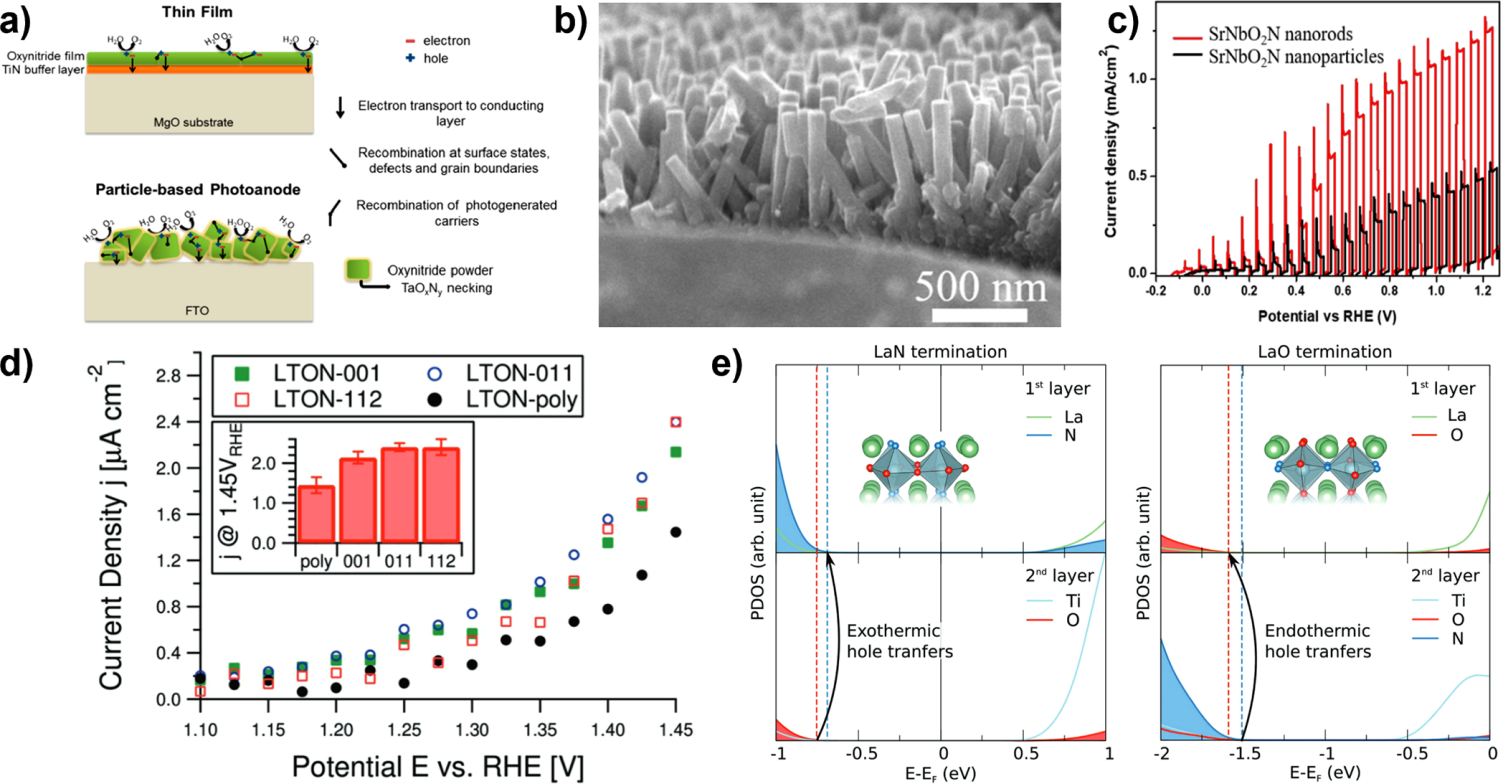
(a) Schematic comparison of thin-film and particle oxynitride photoanodes, noting pathways for photogenerated carrier recombination. Adapted with permission from ref 318. Copyright 2019 American Chemical Society. (b) Side-view SEM of SrNbO_2_N nanorods grown on a metallic Nb substrate. (c) Comparison of chopped illumination current–potential behavior of SrNbO_2_N nanorods (red) and nanoparticles (black) in a 0.1 M Na_2_SO_4_ aqueous electrolyte, pH = 13. (b,c) Adapted with permission from ref 319. Copyright 2021 Wiley-VCH. (d) Comparison of current densities from four LaTiO_*x*_N_*y*_ samples with different thin-film orientations, including polycrystalline LaTiO_*x*_N_*y*_. Note that each current–voltage point was acquired from a separate chronopotentiometric experiment. (e) Comparison of the electronic densities of states for the first and second layer of atoms in LaTiO_*x*_N_*y*_ for regions with LaN termination (left) and LaO termination (right), showing exothermic hole migration toward the LaN-terminated face which is hypothesized to improve the ability of photogenerated holes to perform OER. (d,e) Adapted with permission from ref 314. Copyright 2017 Wiley-VCH.

Like other semiconductors with short minority carrier diffusion lengths, nanostructures are attractive to improve the PEC performance of perovskite oxynitrides. This strategy has been successful for SrNbO_2_N prepared as nanowires by hydrothermal treatment of Nb foil followed by nitridation (Figure 20b,c).^319^ Using CoO_*x*_ nanoparticles as a cocatalyst, the nanowires achieved 1.3 mA cm^–2^ at 1.23 V_RHE_ under AM 1.5G illumination −2.6 times higher than a similar nanoparticle thin film electrode and outperforming a thin-film photoelectrode (synthesized by nitridation of a drop-cast film) due to shortening of the hole diffusion length.^319^ An inverse opal structure, made by sol–gel infiltration of polystyrene with sequential pyrolysis to generate an oxide and ammonolysis to generate the nitride, has similarly improved CaTaO_2_N performance. The resulting 3D CaTaO_2_N had improved light harvesting at the 480 nm absorption onset, a negative photocurrent onset of −0.3 V_RHE_, and photocurrent density of 0.25 mA cm^–2^ at 1.23 V_RHE_, dramatically outperforming a particulate film due to improved charge transfer pathways through the inverse opal structure.^316^

Although substantial work remains to elucidate the fundamental properties of oxynitride perovskites, their stability during PEC operation, particularly regarding N retention, is already being investigated. Work on LaTiO_*x*_N_*y*_ has shown that (001)-oriented films have a higher IPCE at 405 nm than (112)-oriented or polycrystalline films, attributed to La–N terminations favorable for photogenerated hole transfer for OER (Figure 20d,e).^314^ Post-PEC characterization by XPS showed that the LaTiO_*x*_N_*y*_ surface became slightly O-enriched without substantial N loss, and it was suggested that refining anion stoichiometry toward high N content films could improve PEC performance.^314^ Further study using neutron reflectometry and grazing incidence absorption spectroscopy showed the development of a 3 nm oxidized region at the LaTiO_*x*_N_*y*_ surface after PEC operation in 0.5 M NaOH, attributed to oxidation of the La cations and N movement off lattice sites.^320^ These changes from operation were much less pronounced for LaTiO_*x*_N_*y*_ with an IrO_2_ nanoparticle catalyst, indicating OER on the LaTiO_*x*_N_*y*_ surface utilizes a pathway which is detrimental to the semiconductor.^320^ Follow-up work confirmed the increased oxidation of La via the addition of La–O 2p states in the valence band during PEC operation, as well as the formation of an electron accumulation layer at the surface of LaTiO_*x*_N_*y*_, likely leading to charge trapping and recombination.^321^ Similar partial oxidation of the A-site cation and possible loss of N were observed at the surface of SrTaO_*x*_N_*y*_ under OER; unlike LaTiO_*x*_N_*y*_, some photocorrosion was observed for SrTaO_*x*_N_*y*_, indicating different mechanisms of degradation can be at play across this semiconductor class.^322^ Additionally, changes to chemisorbed N-containing species suggested that nitrogen oxidation might compete with OER at the surface of SrTaO_*x*_N_*y*_, a critical point for future investigation.^322^ This body of surface characterization studies highlights the need to develop complete pictures of OER mechanisms for this group of semiconductors in order to improve stability.

As semitransparent semiconductors, there is substantial interest in integrating perovskite oxynitrides directly with other photoelectrode materials in tandem configurations. However, the high temperatures required for ammonolysis treatments can severely limit integration with photoelectrodes with low processing tolerance, such as PV-grade Si. Recent work has shown that metallic TaN can act as a diffusion barrier between SrNbO_2_N and a Si substrate to prevent diffusion between the two layers,^312^ although the 840 °C ammonolysis anneal would still negatively impact the optoelectronic properties of a high-quality Si substrate. Investigations in this vein are important to enable the use of oxynitride perovskites as semitransparent photoanodes for smaller band gap photocathodes, and are likely to become more prevalent as these materials continue to be developed.

### 2.3 Chalcogenides

Chalcogenides, comprising late transition metal *d*-block and groups III, IV, and V *p*-block cations with chalcogenide anions (e.g., S, Se, Te), provide a flexible chemical framework by which to design materials with a broad array of optical and catalytic properties.^323^ Several themes persist across the chalcogenide semiconductors, despite having disparate crystallographic structures and compositions (Figure 21a). The narrow band gaps of these semiconductors, with conduction and valence band edges straddling hydrogen evolution potentials, make for photocathodes with promising solar absorption cross section (Figure 21b). The intrinsic instability of chalcogenides impedes assessment of intrinsic catalytic properties, requiring instead use of a protective layer (commonly TiO_2_) to prevent exposure of the photoabsorber to electrolyte. Formation of heterojunctions provides rectifying interfaces that both increase photovoltage and lower the barrier for interfacial charge transport, limiting recombination losses.^324^ Misalignment of photoabsorber and protective layer valence and conduction bands, however, results in excessive charge carrier recombination, attenuating photovoltages and photocurrents. Similarly, alignment of back contact band levels is frequently neglected.^211^ Fundamental investigations of charge transfer limitations at solid|solid interfaces will provide a more robust framework for designing the electronic structure of future catalyst architectures extending beyond just chalcogenides. Here we discuss photoelectrode examples built from a wide range of chalcogenide structures, providing a perspective on the current state of the art and highlighting areas needing further investigation, primarily the influence of the of heterojunctions and protective layers on charge recombination and photocurrent generation. Several reviews exist discussing the influence of composition and defect chemistry on optoelectronic properties.^325–327^

**Figure 21:**
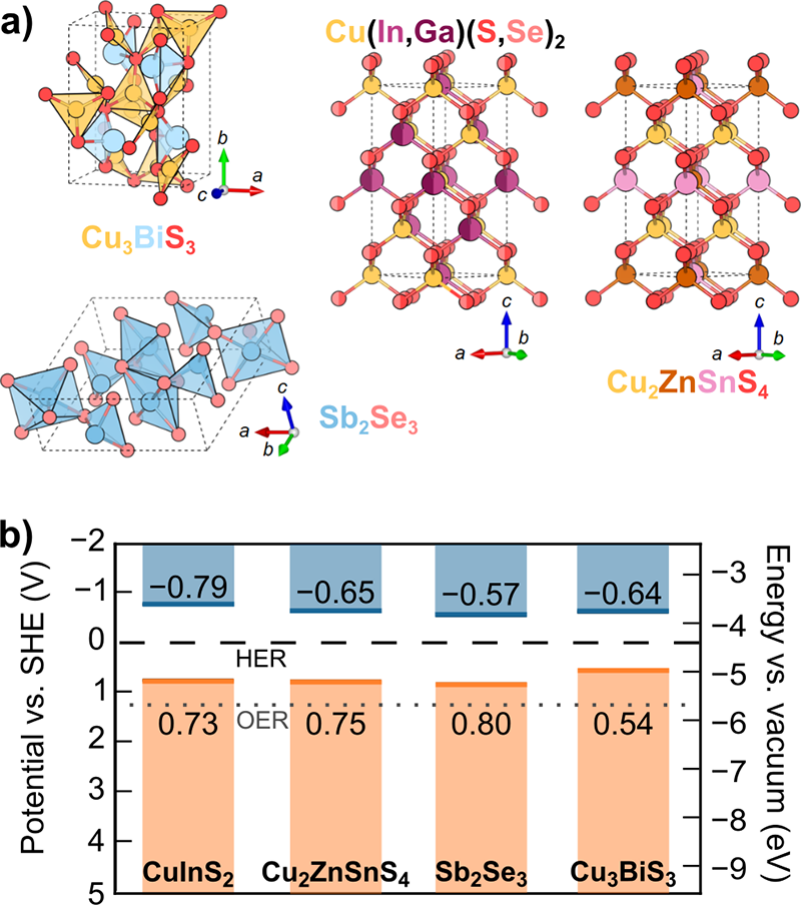
Selected chalcogenide semiconductors for photoelectrodes. (a) Unit cells of Cu_3_BiS_3_, Sb_2_Se_3_, Cu­(In,Ga)­(S,Se_2_), and Cu_2_ZnSnS_4_. (b) Valence band edge maximum (orange line) and conduction band edge minimum (blue line) for each semiconductor with the thermodynamic potentials for HER (black dashed line) and OER (gray dotted line). See SI Tables S7 and S8 for details.

#### 2.3.1 Chalcopyrites (Cu­(In,Ga)­(S,Se)_2_) and Kesterites (Cu­(Zn,Sn)­S_2_)

Chalcopyrites (Cu­(In,Ga)­(S,Se)_2_; I–III–VI_2_) and kesterites (Cu­(Zn,Sn)­S_2_ or CZTS; I_2_–II–IV–VI_4_) are two p-type semiconductor groups that are isostructural with the zinc-blende crystal structure common to mature PV technologies (section 2.4), though with differences in composition and oxidation state of their constituent ions (Figure 21a). Correspondingly, the optoelectronic properties of these materials (mobility ∼10–100 cm^2^ V^–1^ s^–1^ and band gaps from 1.00 eV for CuInSe_2_ (CISe) to 2.43 eV for CuGaS_2_ (CGS))^328–330^ are well-suited to capture appreciable fractions of the solar spectrum with sufficiently high conduction bands to facilitate HER (Figure 21b).^327,331^ However, *V*_OC_ is limited, particularly in kesterites, by a high concentration of intrinsic point defects and trap states within the band gap.^328,332^ PVD (e.g., thermal evaporation and laser ablation) and CVD (e.g., ALD) deposition techniques are well established, with heterojunctions and/or protective layers typically grown by wet chemical methods (e.g., chemical bath deposition and electrodeposition).^327,333–335^ The Fermi levels of chalcopyrites and kesterites are typically located within a few hundred meV of 0.0 V_RHE_, limiting achievable photovoltages.^331^ Poor stability further impedes applied use of these semiconductors as photocathodes, although some current benchmarks achieve several weeks of performance.^336,337^ Developing strategies to enhance photovoltage and stability therefore represent the major barrier toward adoption of chalcopyrites and kesterites as viable photocathodes.

Charge transfer at solid|solid and photocathode|electrolyte interfaces limit HER performance in chalcopyrites and kesterites. Large differences between flat band (0.65 V_RHE_) and hydrogen evolution onset potentials (0.1 V_RHE_) of unprotected CuIn_0.3_Ga_0.7_S_2_ photocathodes are attributed to Fermi level pinning (Figure 22a).^338^ However, confirmation of Fermi level pinning requires observation of band bending being independent of solution potential, and is typically probed using several different facile redox pairs that do not convolute the rates of proton and electron transfer.^72^ Measurements in ferri/ferrocyanide solution of the bare CIGS surface indicate no limitations in charge transfer from the electrode to the electrolyte (Figure 22a), suggesting that proton transfer kinetics limit hydrogen evolution (perhaps being limited by the Volmer step of H^+^ adsorption^339^ as is common for Cu and *p*-block elements^340^). Observation of pH-dependent hydrogen evolution kinetics for CZTS|HfO_2_|CdS|HfO_2_|Pt photocathodes reinforce this conclusion (Figure 22b).^341^ Decreasing photon limited current densities from pH 3.0 to 11.0 suggest the intrinsic kinetics of hydrogen evolution decrease with decreasing proton concentration, manifesting as increased charge carrier recombination at the electrode|electrolyte interface. However, assessment of the pH-dependent hydrogen evolution kinetics considered here may be convoluted by differences in mass-transfer limitations;^253^ phosphate, a facile proton donor,^343^ can nominally donate 2.12 H^+^ per phosphate anion at pH 3.0 and 0.96 H^+^ per phosphate anion at pH 11. While these examples provide significant insight into intrinsic kinetics of charge transfer at the CIGS|CTZS-electrolyte interface, each is convoluted by effects of the *electrolyte*. Fundamental investigations of charge transfer with facile redox pairs in nonaqueous electrolytes (i.e., acetonitrile), or aqueous electrolytes with nonproton-donating species,^252^ may provide greater insight on the fundamental limitations of CIGS and CZTS photocathodes.^345^

**Figure 22:**
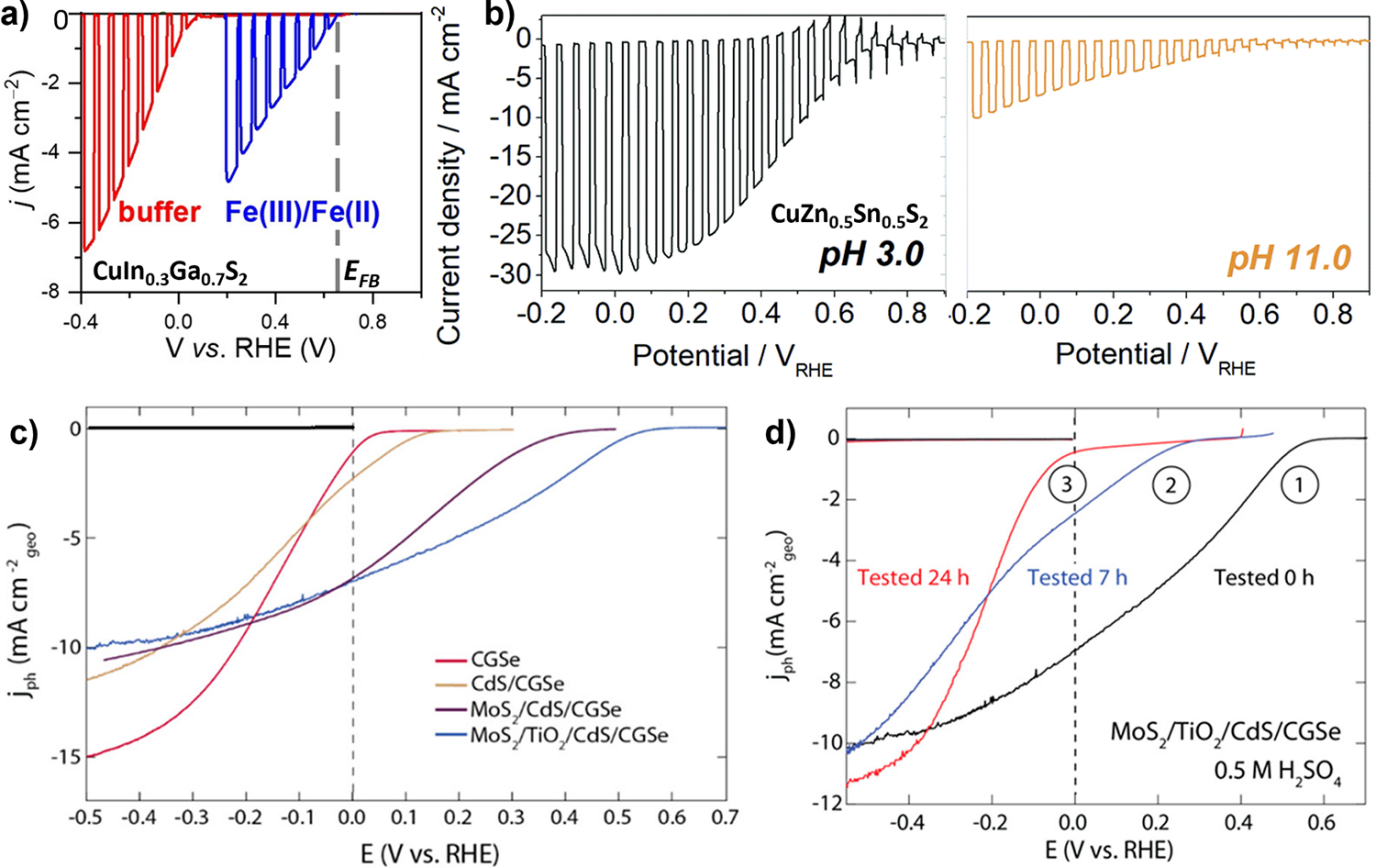
(a) Chopped light linear sweep voltammogram of CuIn_0.3_Ga_0.7_S_2_ (CIGS) in 0.5 M Na_2_SO_4_ with 0.5 M K_2_HPO_4_ solution (pH 6.1) without (red) and with (blue) 0.5 M [Fe­(CN)_6_]^3–^, recorded at 20 mV s^–1^. Adapted with permission from ref 338. Copyright 2021 Wiley-VCH. (b) Chopped light linear sweep voltammogram of Cu_2_ZnSnS_4_|HfO_2_|CdS|HfO_2_–Pt (CZTS) in pH 3.0 (black, left) and 11.0 (orange, right) 0.2 M Na_2_HPO_4_ buffer, recorded at an unknown sweep rate. Adapted with permission from ref 341. Copyright 2021 Royal Society of Chemistry. (c) Linear sweep voltammograms in the dark (black line at upper left) and under AM 1.5G illumination of CuGaSe_2_ (CGSe, red), CGSe|CdS (orange), CGSe|CdS|MoS_2_ (purple), CGSe|CdS|TiO_2_|MoS_2_ (blue) in H_2_-sparged 0.5 M H_2_SO_4_ at 10 mV s^–1^ sweep rate. (d) Linear sweep voltammograms of CGSe|CdS|TiO_2_|MoS_2_ at 0 (1, black), 7 (2, blue), and 24 h (3, red), illustrating destabilization of the surface layers and voltametric behavior comparable to the bare CGSe photoanode after 24 h. (c,d) All linear sweep voltammograms are anodic-going and conducted at 10 mV s^–1^ in H_2_-saturated 0.5 M H_2_SO_4_. Adapted with permission from ref 346. Copyright 2019 American Chemical Society.

Heterojunctions (e.g., CdS,^341,346–349^ ZnS^349^) and protective layers (MoS_2_,^346^ TiO_2_,^346–348^ and WO_3_ ^336^) are commonly employed to enhance photovoltage and protect CIGS photoabsorbers from degradation, in line with the common theme of photocathode protection. Heterojunctions can further increase photovoltage with appropriate band alignment (Figure 22c).^346,348^ Device stability is then limited to the lifetime of protective layers, nominally several hours or days (Figure 22d), where understanding the mechanism of protective layer degradation is crucial to designing more stable photoelectrodes. One investigation of CIGSe|CdS|TiO_2_|Pt found complete removal of the TiO_2_ layer and redistribution of Pt cocatalyst to the CdS layer after 30 linear sweep voltammograms from −0.7 to 0.7 V_RHE_.^348^ The complete removal of chemically stable TiO_2_ suggests a mechanochemical degradation mechanism; poor adhesion, delamination, or pinholes cause pitting and dissolution of the *underlying* CdS and CIGSe substrate. Similar observations were made when substituting CZTS as the photoabsorber.^347,350^

#### 2.3.2 Antimony Selenide (Sb_2_Se_3_)

Stibnite (Sb_2_Se_3_), having distinctly unique crystal structure relative to chalcopyrites and kesterites (Figure 21a), maintains moderate charge transport properties (anisotropic mobility between 0.69 to 2.59 cm^2^ V^–1^ s^–1^).^351^ Sb_2_Se_3_ has indirect (1.03 eV) and direct (1.17 eV) band gaps,^352^ facilitating appreciable solar spectrum absorption and charge transfer to the photoelectrode|electrolyte interface (theoretical maximum ∼40 mA cm^–2^).^353^ Flat band potentials increase from 0.4 to 0.6 V_RHE_ ^354,355^ with increasing annealing temperatures.^355^ Sb_2_S_3_ photocathodes have larger band gaps (1.7 eV) and poorer flat band alignment than their selenide anion counterparts, yielding overall poorer photovoltages and photocurrents (∼25% that of benchmark Sb_2_Se_3_).^356,357^ Additional information on optoelectronic properties and synthesis exist in reviews focused specifically on Sb_2_Se_3_.^358^

The modest electronic properties of Sb_2_Se_3_ result in poor transport of photogenerated charge carriers to protective layers and ultimately to the solid|electrolyte interface. Increased photocurrent with larger grain size indicates recombination within the bulk photoabsorber (e.g., at grain boundaries) limits charge transport.^353^ Manifold physical and solution-based growth techniques exist for Sb_2_Se_3_ with PVD techniques such as close-spaced sublimation and rapid thermal evaporation generating greater quantum efficiencies.^359^ Sequential spin coating of [Sb_4_Se_7_]^2–^-containing precursor inks to form a bottom monolayer and a top nanorod array structure (hierarchical bilayer) has also been shown to be an effective fabrication approach to increase photocurrents by enhancing the light absorbed within the diffusion length of excited charge carriers (Figure 23).^360^ Growth of Sb_2_Se_3_ in a vapor phase selenium atmosphere similarly produced enhanced photocurrent,^354^ where the presence of selenium metal (a photoconductor) likely serves to enhance conductivity, mobilizing photogenerated charge toward the surface. However, photoelectrode stability is limited to several hours in the absence of a protective layers.^361^

**Figure 23:**
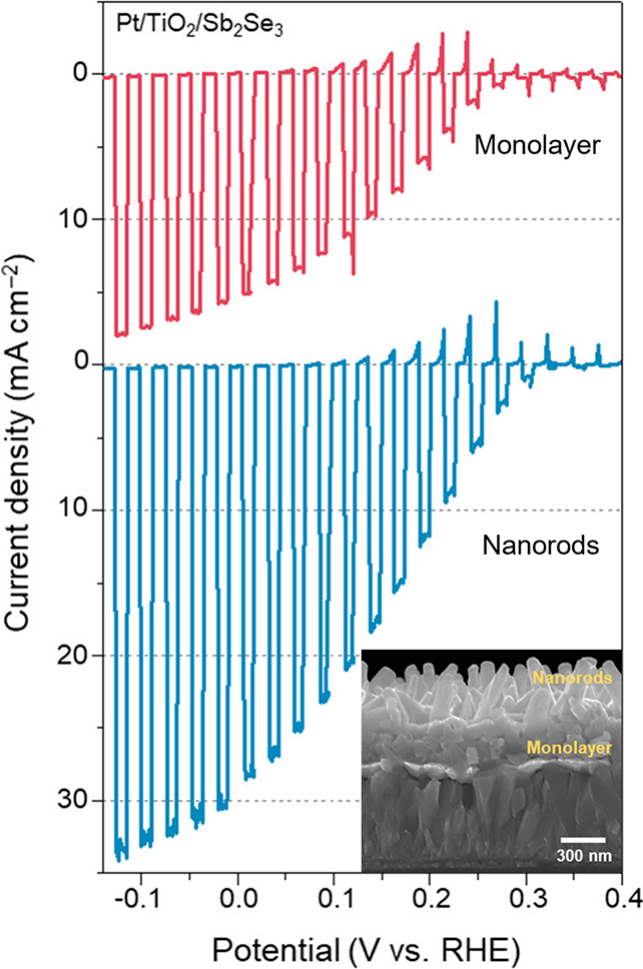
Chopped light linear sweep voltammogram of TiO_2_|Pt protective layer over either a dense Sb_2_Se_3_ film (red, monolayer) or a nanostructured Sb_2_Se_3_ film atop a dense Sb_2_Se_3_ film (blue, nanorods), measured in 0.1 M H_2_SO_4_ at an unknown sweep rate. Enhanced photocurrents are ascribed to greater charge separation efficiency, which may be a manifestation of a greater number of photons absorbed within the effective charge carrier diffusion length of the surface. Adapted with permission from ref 360. Copyright 2020 American Chemical Society.

#### 2.3.3 Copper Bismuth Sulfide (Cu_3_BiS_3_)

Wittichenite Cu_3_BiS_3_ achieves the highest illuminated onset potentials for HER of the chalcogenides considered here (up to ∼0.9 V_SHE_),^362^ having appropriate band alignment to facilitate hydrogen evolution (*E*_CB_ at −0.64 V_SHE_ ^363^ with flat band potentials ca. 0.7 V_RHE_).^364^ Cryogenic optical absorption, supported by density functional theory calculations, of evaporated thin films capture an indirect absorption onset of 1.18 eV, followed by much brighter direct transitions at 1.45 and 1.57 eV,^363^ explaining the broad range of reported band gaps (between 1.2 and 1.7 eV).^362,363,365,366^ However, photocathode stabilities of ca. <1 h limit practical implementation of this semiconductor.^366^ Similar to the other chalcogenides, incorporation of heterojunction (CdS), protective (TiO_2_), and cocatalyst (Pt) layers increase stability, photovoltage, and photocurrents.^362,365^ While incorporation of a CdS top heterojunction enhances photovoltage and marginally increases photocurrent relative to the bare Cu_3_BiS_3_|Pt, addition of a topmost TiO_2_ protective layer provides ca. 6 mA cm^–2^ (∼3× enhancement) of photon limited current density for Cu_3_BiS_3_|CdS|TiO_2_|Pt.^362^ Substituting Cd with In decreases the conduction band offset between Cu_3_BiS_3_ and In_*x*_Cd_1–*x*_S from 0.7 eV (*x* = 0) down to 0.15 eV (*x* = 0.6) before increasing again for stoichiometric In_2_S_3_ (0.49 eV conduction band offset).^365^ The corresponding achieved *j*_SC_ (ca. 11 mA cm^–2^ for In_0.6_Cd_0.4_S and 7 mA cm^–2^ for In_2_S_3_ as the rectifying heterojunctions) suggests that either the rectifying or charge transport properties of the Cu_3_BiS_3_|In_*x*_Cd_1–*x*_S interface are optimized. While charge carrier transport is enhanced, changes in hydrogen evolution onset of ∼200 mV suggest changes in rectifying behavior may also play a role.^365^ The band positions of Cu_3_BiS_3_ manifest in large p-type photovoltages, contrasting the fairly small photovoltages associated with smaller-gap chalcopyrites and kesterites. Developing chalcopyrite| or kesterite–Cu_3_BiS_3_ heterojunctions may provide increased photovoltages that enhance η_STF_ for the chalcopyrite field.

### 2.4 Mature PV Semiconductors

Any discussion of photoelectrode materials would be incomplete without the semiconductors used in PV, including Si, III–Vs (in particular, binary GaAs and ternary alloy GaInP_2_), and CdTe (Figure 24). These semiconductors were some of the platforms for the initial development of semiconductor–liquid junctions,^72–77^ and Si especially continues to be a commonly used photoelectrode platform for emerging concepts and demonstrations in photoelectrochemistry. Despite their development for PV devices, these semiconductors have not provided the breakthroughs needed to commercialize PEC primarily due to fundamental limitations on their stability.^6,66^ Because they are primarily under development for PV applications, we also discuss hybrid organic–inorganic perovskite semiconductors as photoelectrodes in this section, despite their inherent differences from inorganic semiconductors.

**Figure 24:**
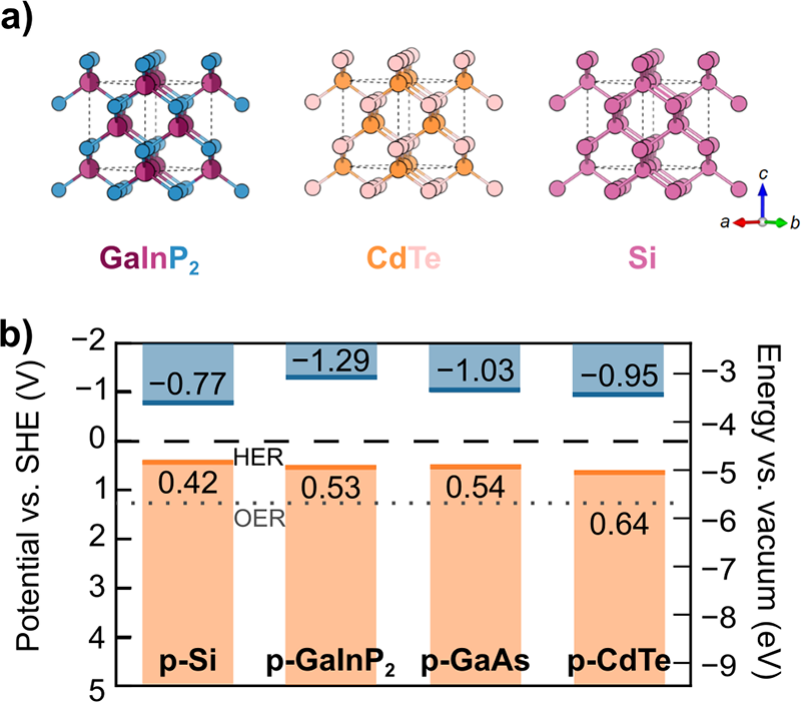
Selected mature PV semiconductors for photoelectrodes. (a) Unit cells of Si, CdTe, and GaInP_2_. (b) Valence band edge maximum (orange line) and conduction band edge minimum (blue line) for each semiconductor with the thermodynamic potentials for HER (black dashed line) and OER (gray dotted line). See SI Table S7 and S8 for details.

While these technologically mature semiconductors have enabled some of the highest efficiency PEC demonstrations, recent years have seen a disconnect between the photovoltages, photocurrents, and efficiencies achievable by PEC and those demonstrated for highly developed PV. Consider state-of-the-art Si PV: minority carrier lifetimes now range from tens of ms to 1 s.^367^ With almost no routes for photoexcited carrier recombination in the bulk, the architecture of Si PV has moved from “traditional” diffused pn homojunctions to structures with no net field across the bulk of the absorber, highly carrier selective contacts, and passivating layers at all interfaces.^367–369^ Such device structures have enabled the demonstration of ever-increasing efficiency, and recent reports have highlighted the competition for improvements as efficiencies for Si-based solar cells approach the detailed balance limit.^370^ However, these efficiencies have not been realized in PEC water splitting because they require both the highest quality Si and high levels of interfacial passivation requiring cleanroom fabrication. The optoelectronic properties of Si sourced for use as photoelectrode substrates are often overlooked and under-reported. Significant improvements in PEC performance may be had borrowing the intentional focus on high-quality semiconductor substrates and engineering interfaces for lossless carrier collection from PV while addressing stability in PEC operating conditions, which has routinely been demonstrated in III–V-based PEC devices, as will be described below.

The inherent instability of PV semiconductors in aqueous electrolyte remains a critical challenge. Unfortunately, the zinc-blende derived structures of these high-performance semiconductors for PV (which enable their optoelectronic properties) are nominally unstable in aqueous electrolytes and typically comprised of catalytically inactive late *d*- and *p*-block elements. This has led to the development of protective schemes for these semiconductors as photoelectrodes, but even the most robust protective layers ultimately fail, resulting in pinholes that can rapidly degrade photoelectrodes.^371,372^ Thus, development of protective layers and catalysis schemes is still needed to enable PV semiconductors as stable photoelectrodes. The past decade has seen multiple examples of protective layers that borrow lessons from PV, such as so-called “leaky” TiO_2_, a protective layer which is also a hole-selective contact for Si and III-Vs.^373^ While further development is needed for protective schemes that can enable these semiconductors to operate as photoelectrodes in the long term, attention should be paid to advances in PV devices and the way that those could be leveraged in the search for materials in PEC.

#### 2.4.1 Silicon (Si)

Silicon is one of the most common substrates for photoelectrode design. The Si *E*_g_ = 1.1 eV is too narrow to drive overall water splitting without additional voltage and is well-positioned for efficient tandem photoelectrode design as a bottom cell.^374,375^ Decades of advances in Si fabrication and use in the PV and microelectronics industry have made high-quality Si readily available, although many PEC studies utilize Czochralski growth Si wafers rather than the longer carrier lifetime float-zone wafers that are now standard for PV devices.^367^ The broad availability of high-quality Si has contributed to the prevalence of this semiconductor in PEC studies, and advances and expertise in dopant control, fabrication and processing has made Si well-suited as a demonstration platform for various strategies for semiconductor interface and structural designs. This can be seen by Si microwire and nanowire-based designs^376^ to decouple light absorption and charge carrier collection lengths, that can serve to inform other semiconductor designs, including core–shell wire structures.^377^ This is further underlined by the wide range of tandem structures that have been developed using Si as a long-wavelength light absorber. Although the Si surface itself is not catalytically active for either HER or OER,^378^ its well-understood nature has made it an ideal testing ground for many new catalysts, which would be difficult to catalog here. The variety of uses and studies for Si within the solar fuels community have been highlighted in many cogent literature reviews.^6,378,379^

One strategy that has recently attracted significant attention for Si-based photoelectrodes is to use metal–insulator–semiconductor (MIS) interfaces at the semiconductor–liquid junction, similar to the passivating contact approaches used in PV.^369^ The MIS architecture opens opportunities for novel and complex combinations catalysts, interfacial layers, and catalysts. It also offers a unique route to photovoltage control by offering differential control of majority and minority carrier interfacial transfer, and has been explored in the past both for PV and PEC devices.^380–384^ Building on this foundational work, there has been significant effort directed toward developing deeper understanding of the factors that dictate MIS performance and demonstration of these strategies for solar fuels production. Recent examples include a combination Al_2_O_3_|SiO_*x*_ insulating layer with a bilayer structured Pt|Ni metal overlayer that has been used to maximize the photovoltage of an MIS photoanode (Figure 25); this work highlights the importance of considering insulator defect states and provided guidelines toward earth-abundant photoanode structures.^385^ A HfO_2_ insulating interface layer has also been explored in an n-Si|HfO_2_|Ir structure, demonstrating the importance of precise insulator layer thickness during fabrication and design, balancing charge transfer rates for electrons and hole with the introduction of defect states in the insulator.^386,387^ Transient reflection spectroscopy (discussed more in section 3.1.2) has complimented investigation of charge transfer rates across these interfaces, highlighting the effect of interfacial SiO_*x*_ layer thickness on both charge transport rates and recombination at the MIS interface.^388^ Theoretical treatments of MIS structures have further discussed the importance of band edge offsets in these structures and the related effects on tunneling for interfacial electron/hole transfer.^389^ These MIS structures have been used to drive a number of solar-fuels relevant electrochemical reactions, including a photoactive p-Si|TiO_2_|Cu structure has been used for light-driven CO_2_ reduction to C_2+_ products^390^ as well as various other structures for OER.^391–394^ Looking forward, optimizing MIS structures for insulator thickness, minimized photon absorption losses and interfacial defect states, and semiconductor|insulator|semiconductor barrier heights promises an opportunity to mutually achieve improved stability and improved activity in next generation photoelectrodes.

**Figure 25:**
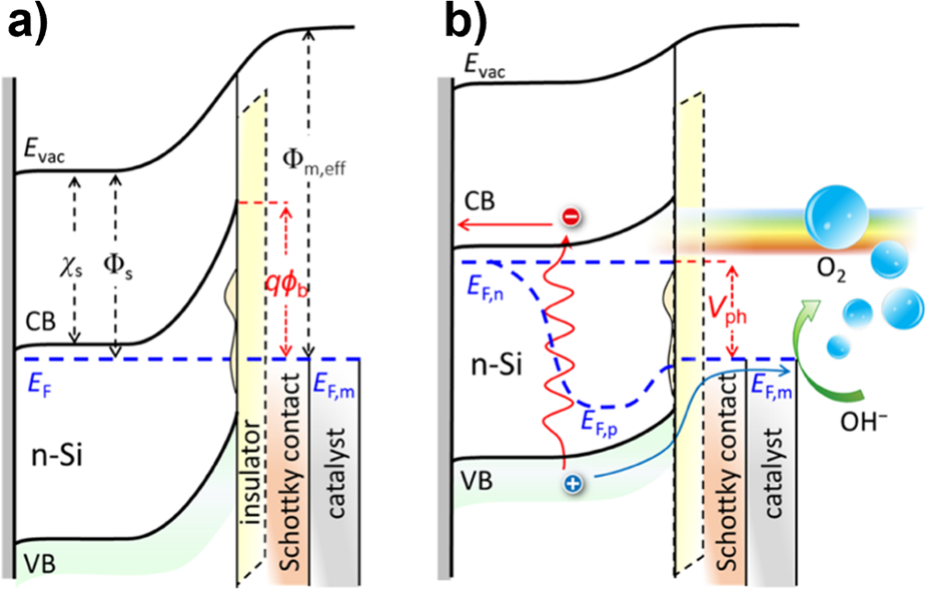
(a) Band energy diagram depicting a n-Si-based MIS junction under equilibrium conditions in the dark. (b) A band energy diagram depicting the MIS junction under illumination conditions in the dark. Here, *E*_F_ is the Fermi level of the electrons (*E*_Fn_) and holes (*E*_Fp_) in the silicon phase. *E*_Fm_ is the Fermi level in the catalyst and Schottky contact phase. *V*_ph_ is the observed photovoltage measured between *E*_Fm_ and *E*_Fn_ at the silicon “back” contact. χ_s_ is the electron affinity of the Si, Φ_s_ is the Si work function, Φ_M_ is the metallic Schottky contact work function, and Φ_b_ is the silicon|Schottky contact barrier height. Adapted with permission from ref 385. Copyright 2018 American Chemical Society.

Although Si is a well-developed semiconductor, it is inherently unstable in PEC conditions for both HER and for OER, where SiO_*x*_ forms rapidly and shuts off photocurrent.^66,378–380^ There is also currently a major focus on protecting Si photoelectrode with other, more stable semiconductors that are also under investigation as photoelectrodes, such as SrNbO_2_N and GaN. A recent study using a n-GaN thin film grown directly on a Si p–n junction as a photoelectrode demonstrated 150 h of stability under concentrated illumination in 0.5 M H_2_SO_4_ with near 100% η_F_ toward HER.^395^ Although the photocurrent was generated by the pn-Si in this photocathode, the performance of the device improved over time, which was correlated to the formation of an oxynitride at the GaN surface. The increased performance of the oxynitride was attributed to hybridization of N 2p and O 2p orbitals, passivating the surface of the GaN.^395^ The integration of Si with other, more stable semiconductors may prove a viable route to leveraging this well-developed semiconductor as part of a tandem photoelectrode for PEC, while its highly documented fundamental properties can also act as a test platform for new catalyst and charge transfer concepts.

#### 2.4.2 III–V Semiconductors

III–V semiconductors are attractive as photoelectrodes due to their moderate band gaps, which are well-aligned for solar spectrum capture and tunable through isostructural alloying, and structural compatibility within the group of III–Vs that enables the design and growth of multijunction absorbers.^81,396^ Epitaxial growth of III–Vs by MBE and MOCVD has enabled complex doping, lattice-matching schemes, and defect control that make III–V multijunctions the highest efficiency PV devices.^397^ State-of-the-art III–V PV utilizes both homogeneous junctions and heterojunctions with related compounds^397^ to separate photogenerated charges. In contrast to current PEC work using Si, III–V-based PEC has frequently benefited from advances in III–V PV. The first demonstration of unbiased water splitting on a single photocathode used a p–n GaAs|p-GaInP_2_ device, generating sufficiently energetic carriers with the combined 1.42 and 1.8 eV band gaps to overcome η_O_ associated with HER and OER.^398^ Because most of the fundamental work on III–V semiconductors has been accomplished through PV devices, many recent studies on III–V photoelectrodes utilize devices that are distinguished from their PV counterparts only by the addition of protection and catalyst layers, a result of the poor stability under PEC conditions and poor native catalytic activity of these semiconductors.^81^ However, single-crystal III–V substrates are often employed in studies of corrosion for simplicity, and prevention of corrosion is now one of the largest areas of research for these semiconductors in the PEC space.

Recent investigations of III–V photoelectrodes have primarily focused on overcoming corrosion, either by implementing a layered protection scheme or by developing catalysts which also serve to stabilize III–V surfaces in contact with electrolyte. Pt nanoparticles deposited directly on GaInP_2_ have been shown to both improve HER kinetics and reduce corrosion pathways on the photocathodes (Figure 26a). In acidic electrolyte, the nanoparticles inhibited formation of In^0^ on a p-GaInP_2_ photocathode surface during HER, enabling retention of photoactivity, although the photocathode still dissolved at 0.3 nm h^–1^ (by ICP-MS).^62^ When a p–n^+^ GaInP_2_ homojunction photocathode was studied with the Pt catalyst in both acidic and basic electrolyte, thinning of the n^+^ layer reduced the photovoltage over time as the homojunction was lost, and revealed crystal defects down to the GaAs substrate used to grow the photocathode.^399^

**Figure 26:**
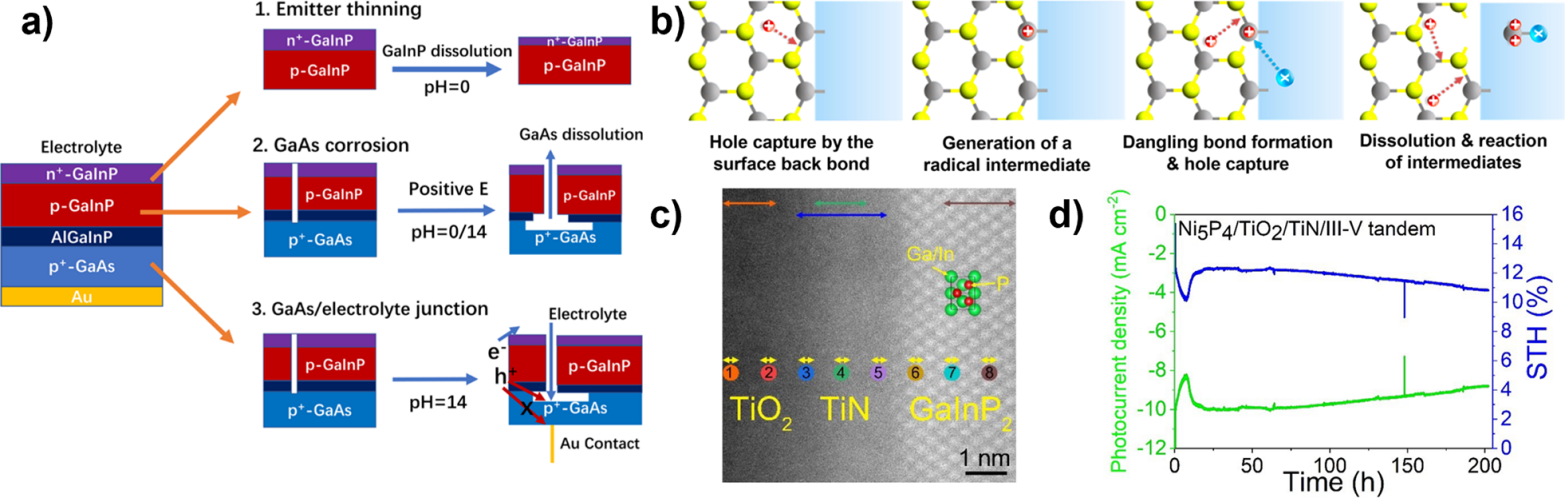
(a) Failure modes of a GaInP_2_-based photocathode under acidic and alkaline conditions. Note the complexity of the photoabsorber device structure, including pn^+^ homojunction and growth on a p^+^-GaAs substrate. Adapted with permission from ref 399. Copyright 2022 American Chemical Society. (b) Steps of anodic photocorrosion reactions on zinc blende-type semconductors (III–Vs and CdTe). Adapted with permission from ref 66. Copyright 2022 American Chemical Society. (c) High-angle annual dark field (HAADF) TEM cross-section of the GaInP_2_|TiN|TiO_2_ interface, with elemental mapping used to determine that there was not cross-diffusion of the layers. (d) Photocurrent density (green) and η_STH_ (blue) for the same protected tandem photocathode, showing good retention of photocurrent for over 200 h. (c,d) Adapted with permission from ref 400. Copyright 2024 American Chemical Society.

MoS_2_ has also been used as a protective catalyst for III–V photoelectrodes, both for tandem devices and for single-layer III-Vs. A graded catalytic-protective scheme, achieved by depositing TiO_2_ by ALD and MoS_2_ by electrodeposition with subsequent annealing at 450 °C, protected a p-GaInP_2_ photocathode from degradation in acidic electrolyte, enabling HER operation at 0 V_RHE_ for 20 h with only a 20% loss in photocurrent over that period.^401^ Similarly, direct photodeposition of a defect-rich MoS_2_ film on a p-GaInP_2_ photocathode extended the operating lifetime during HER from <5 h to >50 h,^402^ and another TiO_2_|MoS_2_ scheme protected GaP, deposited heteroepitaxially on Si, for 3 h of HER operation at pH 0.^403^ Finally, the addition of a conformal layer of MoS_2_ to a GaAs|GaInAsP photocathode provided both catalytic and barrier protection during HER, enabling the photocathode to operate for 10 h longer than an identical photocathode with Pt catalysts.^372^

TiO_2_ is the prototypical protective layer for III–V semiconductors following the demonstration of “leaky” TiO_2_.^373^ Typically, III–V photoabsorbers have not been used in photoanodes as these materials quickly corrode under oxidative conditions and in basic electrolytes. Computational modeling of the kinetics of binary III–V photoanode photocorrosion showed that stability is largely controlled by the rate of charge transfer from the semiconductor to the catalyst on its surface and by the surface back-bond potential of the semiconductor, suggesting routes for stabilization of some III–Vs as photoanodes (Figure 26b).^66^ Protection of n-GaAs by promoting OER with Ir catalysts has also been investigated, but pinholes rapidly developed in the Ir films regardless of deposition method, resulting in rapid GaAs photocorrosion.^404^ Even with protection, pinhole formation in TiO_2_ layers has been observed during OER operation of a TiO_2_-protected p^+^-GaAs photoanode in basic electrolyte.^405^

The success of leaky TiO_2_ as a protective layer also led to the use of TiO_2_ to protect III–V photocathodes such as p-InP. Very thin (10 nm) ALD TiO_2_ acts as both a protective layer and a carrier-selective contact, facilitating electron extraction to the electrolyte and reducing surface recombination, which can improve *V*_OC_ (boosting it by 180 mV to 0.81 V_RHE_)^406^ although the method used to deposit the TiO_2_ can have a profound impact, as plasma-enhanced ALD and some Ti precursors can damage the interface limiting photocathode performance.^407^ The quaternary III–V alloy InGaAsP, *E*_g_ = 0.92 eV, has also been demonstrated as a photocathode protected by TiO_2_, potentially providing a route to a stable bottom cell in contact with electrolyte for front-illuminated III–V multijunction photocathodes.^408^ In another recent example, a GaAs|GaInP_2_ III–V tandem photocathode with added layers of TiN, TiO_2_, and Ni_5_P_4_ performed unassisted HER for 202 h under 1 sun illumination, maintaining >10% η_STH_ during the entire test (Figure 26c,d).^400^ The Ni_5_P_4_ layer was electrolyte-facing and catalyzed HER, while the TiO_2_ layer (deposited by PLD) served as a barrier to prevent electrolyte contact with the III–V photoabsorber, and the TiN layer (also by PLD) prevented diffusion between the TiO_2_ and GaInP_2_ during a 375 °C anneal of the TiO_2_. This final study is notable both for the remarkable stability of the photocathode performance and for the attention to the stability of the semiconductor device itself,^400^ as many PV-grade semiconductors are easily damaged by high temperature thermal cycling used to deposit protective schemes.

Nanostructuring of III–V semiconductors has also been explored for photoelectrode applications. Although such nanostructures may not use p–n junctions, III–V semiconductors are highly sensitive to surface defects require passivation to improve photocurrent in such structures even if light capture is increased. Top-down synthesis of nanowires by dry etching of a p-GaInP_2_ layer with a mask of Ag nanoparticles improved both the photovoltage and photocurrent of the photocathode compared to a bare p-GaInP_2_ photocathode when properly passivated with (NH_4_)­S.^80^ The graded shape of the etched nanowires also slowly changed the index of refraction of the array, helping to suppress recombination. Similarly, top-down etching of p-InP and passivation with sulfur-dissolved oleylamine to form insoluble InPS_4_ improved the photocurrent of the photocathode compared to a planar layer, and stabilized HER at ∼33 mA cm^–2^ for nearly 10 h.^409^ Alternatively, TiO_2_ can also been used to protect III–V nanowire photoelectrodes, and has been shown to protect GaAs p–n core–shell nanowires under HER operation for 67 h.^410^ Protective layers will most likely continue to be the major focus of PEC research on III-Vs in the near future, with the intent of extending the operational lifetimes of these highly efficient photoelectrodes.

#### 2.4.3 Cadmium Telluride (CdTe)

CdTe, used in commercialized thin-film PV devices, has the appropriate optoelectronic properties to efficiently generate and transport charge carriers (1.5 eV band gap,^411,412^ large mobilities,^413^ and long charge carrier lifetimes^414^), and appropriate band alignment for both water reduction (when p-type) and oxidation (n-type).^415^ The susceptibility of CdTe to corrosion (particularly in light of the carcinogenic nature of Cd) diminishes its immediate value as a photoelectrode,^73,416^ requiring protective layers for stability and rectifying heterojunctions and cocatalysts for enhanced (photo)­electrochemical kinetics. Rectifying interfaces are required to improve the poor photovoltages.^417^ However, the defect-rich nature of such heterojunctions, particularly when formed over polycrystalline CdTe, compromises photocurrents.^418^ While grain boundaries in polycrystalline CdTe thin films enhance PV device carrier collection,^419^ in photoelectrodes they can act as defects for high local corrosion rates. Combining heterojunction design strategies developed on polycrystalline CdTe with the well-defined interfaces of CdTe single crystals may provide promising photoelectrode architectures.

Despite these challenges, both n- and p-type CdTe have demonstrated appreciable photovoltages and photocurrents for OER and HER, respectively. Single crystalline (111) n-CdTe produces ca. 20 mA cm^–2^ of light-limited current when capped with a Ni cocatalyst and 140 nm of amorphous TiO_2_ grown by atomic layer deposition (Figure 27a).^417^ However, the low photovoltage (OER onset of 1.1 V_RHE_) suggests flat band potentials near the water oxidation redox potential. In contrast, use of a polycrystalline CdTe layer severely compromises photocurrents, achieving only ca. 5 mA cm^–2^ by 1.2 V_RHE_.^418^ Addition of a rectifying heterojunction, protective layer, and cocatalyst in FTO|CdS|CdTe|MoO_*x*_|TiO_2_|Ni provides increased photovoltage (CdS|CdTe) and enhanced stability and OER kinetics (TiO_2_|Ni), with OER onset observed by ca. 0.3 V_RHE_.^418^

**Figure 27:**
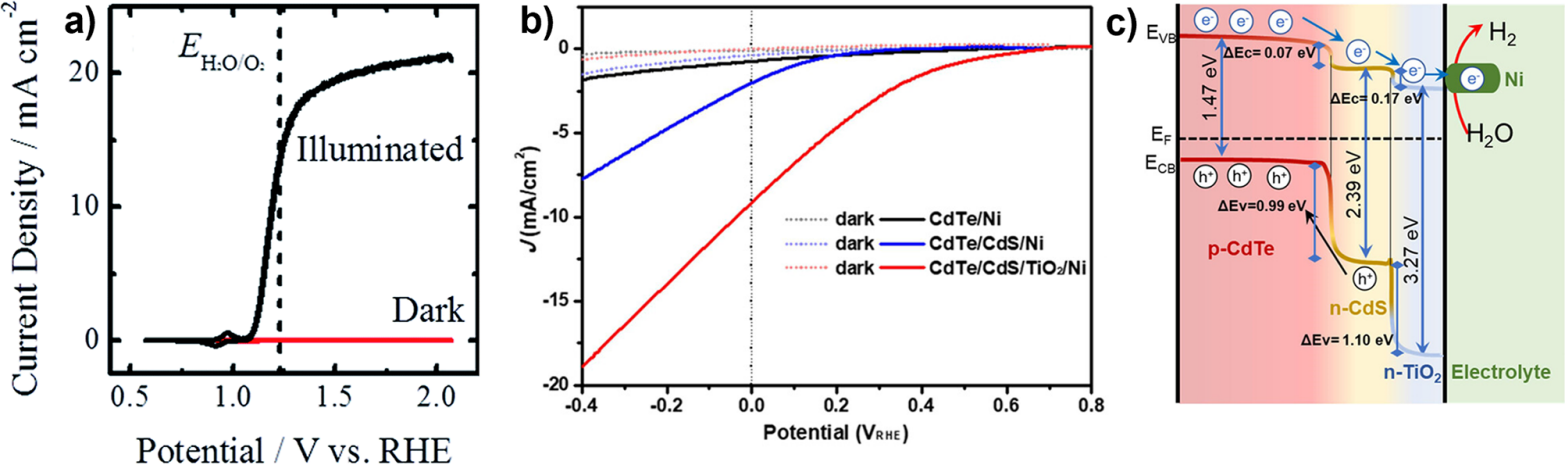
(a) Cyclic voltammetry of (111) n-CdTe, capped with 140 nm thick amorphous TiO_2_ and a Ni cocatalyst, measured in 1.0 M KOH under 100 mW cm^–2^ AM 1.5G illumination (black trace, Illuminated) and in the dark (red trace, Dark). Adapted with permission from ref 417. Copyright 2014 Royal Society of Chemistry. (b) Linear sweep voltammograms measured at 30 mV s^–1^ in the dark (dashed) and under 100 mW cm^–2^ AM 1.5G illumination (solid) of CdTe|Ni (black), CdTe|CdS|Ni (blue), and CdTe|CdS|TiO_2_|Ni (red) in 0.1 M NaH_2_PO_4_ buffer (pH 5). (c) Illustration of the type-II band alignment of p-CdTe with n-CdS and n-TiO_2_, demonstrating how appropriate band alignment of photoabsorber, rectifying, and protective layers enhance charge transport and limit interfacial recombination events. (b,c) Adapted with permission from ref 324. Copyright 2023 American Chemical Society.

A recent investigation of CdTe|CdS|TiO_2_|Ni photocathodes provides a systematic investigation of the influence of each capping layer on photocurrent and photovoltage (Figure 27b).^324^ TiO_2_ provides stability, though only increases photocurrent at potentials <0 V_RHE_. Incorporation of a CdTe|CdS rectifying junction pushes hydrogen evolution onset to ∼0.7 V_RHE_. The valence and conduction states of CdS lie intermediate of those of CdTe and TiO_2_, promoting interfacial charge transfer and mitigating recombination at solid–solid interfaces (Figure 27c). Addition of the Ni cocatalyst decreases kinetic photovoltage losses, manifesting in increased photovoltage and photocurrent. Band alignment of the back contact similarly influences charge transfer of photogenerated majority carriers, where back-contacting CdTe|CdS|Pt photocathodes to FTO|Au|Cu enhances both photovoltage and photocurrent relative to either FTO|Au or FTO.^420^

While the addition of additional semiconductor layers enhances stability and introduces photovoltage inducing rectifying interfaces, each of these interfaces also introduces sites for charge carrier recombination. Given the poorly defined optoelectronic properties of typical as-synthesized polycrystalline CdTe thin films, it is difficult to distinguish whether charge carrier recombination occurs at protective and rectifying layer interfaces or from within the bulk CdTe thin films. A simple solution to many of these issues is to use commercially available single crystals as benchmark substrates, providing a uniform photoabsorber around which changes to photoelectrode capping layer architecture can better be evaluated. Given the commercial availability of CdTe single crystals, incorporation of similar rectifying heterojunctions and protective layers discussed in ref 418 may indeed provide photoanodes capable of generating light-limited currents well over 10 mA cm^–2^ and photovoltages nearing 1 V.

#### 2.4.4 Halide Organic–Inorganic Perovskites

Hybrid organic–inorganic halide perovskites have quickly gained prominence over the last 15 years as new photoabsorbers for PV. With their long carrier lifetimes, large optical absorption coefficients, and ease of synthesis quickly enabling high efficiencies, it should be no surprise that there has also been substantial interest in applying these semiconductors to PEC fuel formation.^421–423^ In PEC systems, halide perovskites also offer band gaps that easily tuned by chemical substitution and band edge positions which are largely compatible with water splitting (as well as CO_2_RR).^421,423^ Because of the compositional and synthetic range presented by halide perovskites, their hybrid organic/inorganic nature, and because the PEC applications of halide perovskites have recently been reviewed in detail elsewhere,^421–424^ here we highlight only recent studies that address the primary factor hindering PEC applications of halide perovskites: stability.

The ease of synthesis of halide perovskites is directly correlated with the tendency of these semiconductors to degrade, even as PVs, in the presence of water.^421^ Recent PEC studies have focused on fully encapsulating the halide perovskite active layers from solution without impacting their optoelectronic properties, using a conductive adhesive paste: to interface with graphite coated with HER and OER catalysts for a side-by-side tandem water splitting system with 13.4% η_STH_ (Figure 28a,b);^425^ and to interface with Pt foil, enabling a stacked halide perovskite tandem that, coupled with a IrO_*x*_ anode, delivered 15% η_STH_ (Figure 28c,d).^426^ Another similar strategy has been to create stacked tandems with other semiconductors, providing additional protection for the halide perovskite by placing the sturdier semiconductor closer to the electrolyte, as when the conductive adhesive paste and graphite protection scheme was demonstrated for a halide perovskite|Si tandem with 20.8% η_STH_.^425^ Like studies on the other PV semiconductors discussed here, these photoelectrodes still grapple with the ultimate failure of protective schemes.

**Figure 28:**
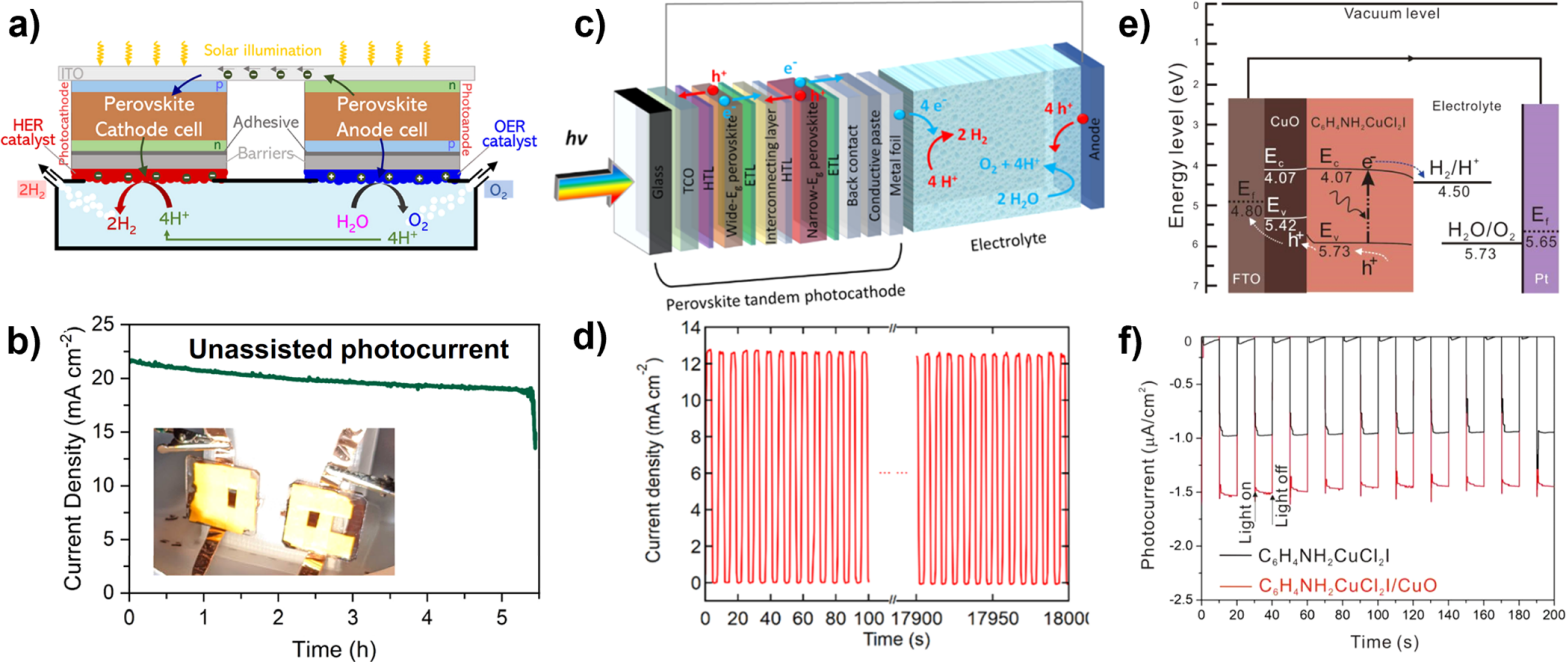
(a) An unassisted PEC water-splitting system comprised of two halide perovskite devices side-by-side with (b) the photocurrent performance of those devices. (a,b) Adapted with permission from ref 425. Copyright 2023 Nature Portfolio. (c) A tandem halide perovskite photocathode capable of driving overall water splitting with (d) its performance under chopped illumination over 5 h. (c,d) Adapted with permission from ref 426. Copyright 2023 American Chemical Society. (e) Schematic of C_6_H_4_NH_2_CuCl_2_I photoelectrode with CuO interlayer to improve photogenerated hole extraction and (f) performance under chopped illumination (11.5 mW cm^–2^). (e,f) Adapted with permission from ref 427. Copyright 2021 American Chemical Society.

Notably, there is also work that deals directly with the issue of halide perovskite stability in contact with an electrolyte, which can influence charge transport and transfer as well as stability. One investigation used C_6_H_4_NH_2_CuCl_2_I (*E*_g_ = 1.66 eV), a water-stable alternative to the prototypical CH_3_NH_3_PbI_3_, as a photocathode for water splitting.^427^ Impedance spectroscopies were used to extract charge carrier diffusion lengths, which were improved by the use of CuO as a hole extraction layer, improving both the photocurrent and photovoltage of the photocathode (Figure 28d,e). While the overall performance of the photocathode was very low (0.221% η_STH_ with the CuO layer), the C_6_H_4_NH_2_CuCl_2_I film was remarkably stable in both acidic and alkaline electrolytes, and remained effectively unchanged after 200 h immersed in water.^427^ Another study on the purely inorganic halide perovskite Cs_2_TeI_6_ tracked the formation of a surface in nonaqueous electrolyte to shed light on the degradation mechanism of this semiconductor in PEC environments.^428^ Should halide perovskites continue to be investigated as photoelectrodes without extensive encapsulation, further studies should investigate mechanisms of degradation, electrolyte infiltration, and surface transformations of these semiconductors.

### 2.5 Protective Layers: The Example of Transition-Metal Dichalcogenides

While this discussion of photoelectrode chemistry has featured challenges for the development of many different semiconductor classes and the material-specific strategies to improve, e.g., light absorption, stability, and charge transfer across the semiconductor–electrolyte interface, there have also been efforts to design cross-material strategies that could be more widely applied to multiple types of photoelectrodes. There have been several attempts to develop impermeable protective layers that can be applied postsynthesis and catalyze the desired fuel-forming reaction.^429–431^ One notable cross-material strategy is modification with transition metal dichalcogenides (TMDCs) as ultrathin coatings, which we present here as a vignette. TMDCs are attractive surface modifiers for photoelectrodes given their optoelectronic properties,^432–434^ electrolyte stability,^432^ and high surface-to-volume ratio providing additional catalyst sites.^432,434,435^ Semiconducting variants have the capability to absorb additional light and facilitate charge separation, while metallic counterparts can potentially contribute to the enhancement of catalytic sites on the TMDC surface. Figure 29 highlights various ways in which pristine and modified TMDC coatings can lead to improved photoelectrode performance that will be discussed here. Although this section is divided into the effects of (i) improved kinetics and charge transfer (ii) improved stability, often the two effects occur concomitantly.

**Figure 29:**
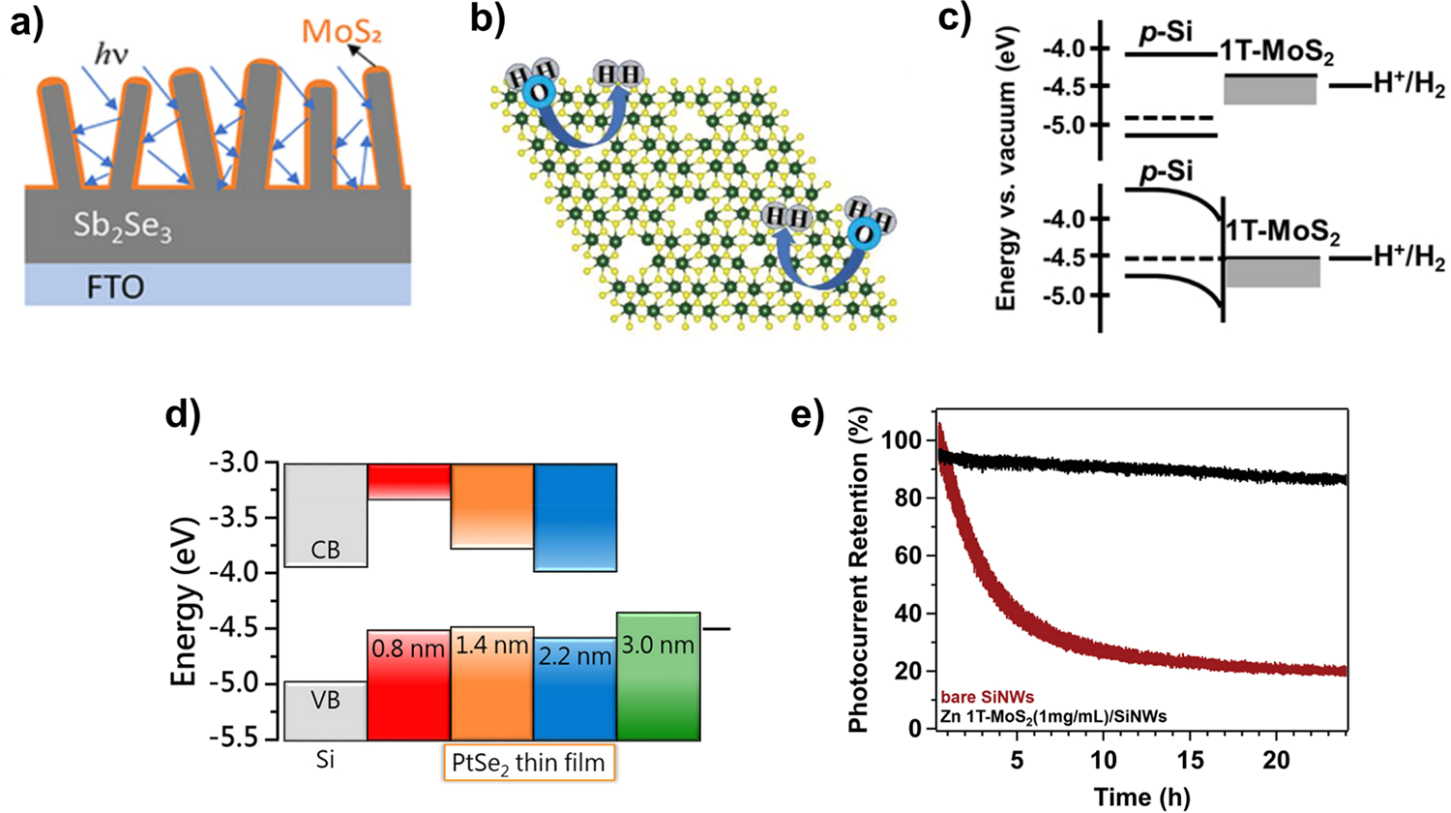
Surface modification with ultrathin TMDC coatings for enhanced PEC performance. (a) Direct coating of neat MoS_2_ on Sb_2_Se_3_ for PEC. The nanorod architecture allows efficient light capture as well as provides active sites for catalysis. Adapted with permission from ref 436. Copyright 2020 Wiley-VCH. (b) Schematic showing defect engineering in ReS_2_ by He ion bombardment, which can generate defects spatially distributed across a sample. Adapted with permission from ref 437. Copyright 2019 Wiley-VCH. (c) Phase engineering of 2H MoS_2_ coating to 1T. The 1T coating is more active to catalysis and offers reduced charge transfer resistance at the electrode|electrolyte interface. Thus, the phase engineered 1T MoS_2_ is ideal as a coating material for p-Si for PEC applications. Adapted with permission from ref 438. Copyright 2014 American Chemical Society. (d) PtSe_2_ showing modulation in band structure with thickness. As such layer engineered PtSe_2_ can be used for charge carrier separation from the bulk Si photoelectrode. The 2.2 nm (3 layers) PtSe_2_ with the “ideal” band structure results in best PEC performance. Adapted with permission from ref 439. Copyright 2021 American Chemical Society. (e) Improved photocurrent retention in Zn modified 1T-MoS_2_|Si nanowires compared to the bare Si nanowires. Adapted with permission from ref 440. Copyright 2022 Wiley-VCH.

The examples given here demonstrate how rational choice of surface coatings with TMDCs can help address some of the major drawbacks of bare semiconductor photoelectrodes. Not only do the coatings provide enhanced stability, but they can also lead to improved kinetics and charge separation. For instance, if a photoelectrode suffers from significant recombination of electron–hole pairs, a TMDC with an appropriate band alignment can act as a selective front contact, reducing recombination and increasing efficiency. Alternatively, the photoelectrode may have sluggish kinetics due to a limited number of active sites, which could be improved by addition of high-surface-area TMDCs. It should be noted that chemical vapor deposition at up to 1000 °C is the most common route for synthesizing TMDCs^441^ many of the semiconductors discussed here as photoelectrodes cannot withstand such high temperature treatment. If photoelectrodes are sensitive to high temperature processing, TMDCs can also be wet transferred following growth on a different substrate.

#### 2.5.1 Enhancing Charge Transfer and Kinetics

Despite the strong light absorption by bare semiconductors, PEC activity can often be significantly altered (enhanced) by applying ultrathin coatings to the photoelectrode, as the native surface typically offers a limited number of catalytically active sites. TMDCs with active sites on their basal planes are particularly interesting for this application, due to their large surface to volume ratio.^435^ However, the 2H phase of many TMDCs have inert basal planes, limiting additional active sites to the edges;^442,443^ such materials need additional modification to enable use on photoelectrodes for this purpose. To overcome the low activity of the basal plane, platelets of MoS_2_ can be grown vertically, exposing a high density of catalytically active edge sites. ALD-deposited vertical MoS_2_ has been shown to increase the current density of a hydrothermally grown CdS nanorod photoelectrode, improving the saturation current density of OER to 1.9 mA cm^–2^ at 0.8 V_RHE_, a nearly ∼50% improvement over the bare nanorods at the same potential.^444^ The MoS_2_ modified photoelectrode also showed a 2-fold increase in EQE to that of the pristine CdS nanorods on ITO due to favorable binding on the edge sites and improved charge separation, attributed to the formation of a heterojunction at the CdS|MoS_2_ interface. On the other hand, NbS_2_ has exceptional stability in acidic electrolytes in addition to having a catalytically active basal plane, making it a promising surface modifier to improve HER on kinetically slow photoelectrodes.^445^ Single-step CVD synthesis of 2D-NbS_2_ on Si nanowires has recently been shown to improve charge transfer kinetics for the photocathode, enhancing turn-on potential for HER from 0.06 to 0.34 V_RHE_ and reaching a *j*_SC_ density of −28 mA cm^–2^ at 0 V_RHE_ with the addition of NbS_2_. Exploiting the HER-active edge sites of ReS_2_ has also been shown to improve Si photocathode by providing both active sites and lower conduction and valence band positions than the Si, enabling electron flow to the electrolyte interface while blocking hole transfer that could cause recombination.^446^ The addition of ReS_2_ (which strongly absorbs in the visible range) increased photocurrent onset from 0.06 to 0.36 V_RHE_ in acidic conditions, and increased the photocurrent density at 0 V ∼23 times to −9 mA cm^–2^.

In another case, vertically grown TMDCs improved not only charge separation and active site density, but also light absorption in a photoelectrode. Sb_2_Se_3_ grown by close-spaced sublimation and subsequently coated with MoS_2_ by sputtering achieved −10 mA cm^–2^ toward HER at 0 V_RHE_ in a buffered near-neutral solution, compared to −4.2 mA cm^–2^ for the bare Sb_2_Se_3_.^436^ The nonplanar geometry of the photocathode helped with increased absorption by minimizing reflection (Figure 29a), while the MoS_2_ coating helped with charge separation as well as protection of the Sb_2_Se_3_ against photocorrosion.^436^ Electrostatic force microscopy (EFM) was used to compare the efficacy of charge separation between the neat Sb_2_Se_3_ nanorods and the MoS_2_ coated Sb_2_Se_3_ nanorods. The uniformity of EFM images confirm efficient charge separation/distribution post MoS_2_ coating due to the larger work function of MoS_2_ compared to Sb_2_Se_3_ resulting in efficient transfer of electrons.

Although vertical coating has enabled MoS_2_ integration with photoelectrodes, the inertness of the basal plane in the 2H phase (which is shared by other TMDCs such as WS_2_) poses a challenge to the use of these materials as catalysts, leading to the adaptation of multiple strategies for engineering of defects, phase, and band gaps of TMDCs for integration with photoelectrodes. While as-synthesized ReS_2_ has been shown to improve the performance of Si photocathodes, introduction of defects using argon beam bombardment in ReS_2_ (Figure 29b) further increased the density of active sites for HER, enhancing both the photocurrent density and photovoltage of a defective ReS_2_|Si photocathode compared to both the bare Si and control pristine ReS_2_|Si.^437^ Optimizing the defect density of the ReS_2_ by tuning bombardment time also afforded a low Tafel slope of 73.7 mV dec^–1^ compared to the bare Si (180.8 mV dec^–1^), pointing to lower charge transfer resistance at the semiconductor–electrolyte interface.^437^ Accessing different phases of TMDCs can also help improve PEC performance, if those phases can be stabilized. Intercalation of Zn^2+^ ions into MoS_2_ increases interlayer spacing and hydrophilicity by improving bonding with surrounding water molecules, stabilizing 1T MoS_2_ (Figure 29c) for use on photoelectrodes.^440^ The Zn-intercalated MoS_2_ was used on Si nanowires and demonstrated HER catalysis in acid that was comparable to adding Pt to nanowires.^440^ Finally, manipulating the band structures of TMDCs can improve band alignments with photoelectrodes to improve kinetics as the band gaps of some TMDCs, such as PtSe_2_, have semiconductor-to-semimetal transitions as the number of layers approaches bulk.^439^ PtSe_2_ films with different numbers of layers were applied to Si photocathodes to form heterojunctions to probe the effect of this transition, which revealed that three layers of the PtSe_2_ afforded the best band alignment with the Si and acted as a charge-selective layer (Figure 29d). While the bare Si showed an onset potential of – 0.24 V corresponding to a photocurrent density of 1 mA cm^–2^, the 2.2 nm (3 layers) PtSe_2_ coated Si showed a large shift to 0.27 V_RHE_ at the same photocurrent density demonstrating much improved performance.^439^

#### 2.5.2 Enhancing Stability

In addition to improving reaction kinetics, incorporation of TDMCs often simultaneously leads to improved photoelectrode stability. Many TDMCs are extremely stable^445^ in acidic solutions, making them ideal as protective coatings for HER in particular. Vertical (3D) MoS_2_ from metal organic chemical vapor deposition was used in conjunction with a TiO_2_ layer as a protective scheme for Si photocathodes, with the TiO_2_ layer enabling direct growth of the MoS_2_ on the Si by protecting the Si from oxidation and enhancing MoS_2_ nucleation.^447^ The 3D MoS_2_ coating improved both the photocurrent onset and maximum photocurrent of the photocathode, pushing them to 0.35 V and ca. −37 mA cm^–2^ respectively, compared to −0.41 V and −32 mA cm^–2^ for the bare Si and 0.2 V and 33 mA cm^–2^ for a planar Si|TiO_2_|MoS_2_ control. Even more impressively, the 3D MoS_2_ protected the photocathodes from degradation in acid for nearly 3 h, showing almost no change in photocurrent compared to a 63% photocurrent retention for Si|TiO_2_ and only a 13% photocurrent retention with rapid degradation for bare Si photocathodes.^447^ Another strategy to employ MoS_2_ as a protective scheme used amorphous MoS_*x*_ (with many defects and undercoordinated S atoms that provide higher activity than pristine MoS_2_ on the basal plane) in order to protect GaInP_2_ with TiO_2_ (somewhat similar to the demonstration of CGSe|CdS|TiO_2_|MoS_2_ in section 2.3.1 ^346^). An annealed, graded TiO_2_|MoO_*x*_|MoS_*x*_ scheme on the GaInP_2_ photocathode surface enabled high catalytic activity (11 mA cm^–2^ at 0 V_RHE_ under 1 sun illumination) and stability (80% photocurrent density retained over 20 h), while the photocurrent density of an unannealed, amorphous GaInP_2_|a-TiO_*x*_|MoS_*x*_ photoelectrode decreased from 10 mA cm^–2^ to 5 mA cm^–2^ over the same time period.^401^ Similarly, phase engineered 1T Zn intercalated MoS_2_ on Si nanowires enabled the retention of 66% of the initial photocurrent over 24 h of PEC operation (Figure 29e).^440^

## 3 In Situ Photoelectrode Characterization

As demonstrated in the previous section, the photoelectrodes used to drive PEC fuel-forming reactions span the full range of complexity from bare semiconductors in direct contact with electrolyte to complex, multilayered devices that rely on a delicate balance of charge transfer steps. In all cases, PEC fuel formation operates far from thermodynamic equilibrium, and despite the long history of this field, there are still many dynamic processes (e.g., dissolution, oxidation, *E*_f_ pinning) that are not fully understood. While disentangling the evolution over time of multiple surfaces and interfaces is complicated, understanding their individual role and dynamics is critical to predicting and improving PEC performance.

Electrochemical characterization tools beyond those for simple performance evaluation, e.g., dual working electrode (DWE), have been popularized recently for photoelectrodes and have had success probing the nature of interfacial charge transfer processes. Outside of evaluation of electrochemical properties on the basis of current density–voltage (*J*–*V*) or impedance signals, many PEC surfaces have been studied with great success via ex situ material characterization as a function of operation and/or testing condition (e.g., illumination, time, applied potential, electrolyte). However, in most cases, removing the photoelectrode from the PEC cell alters the surfaces under investigation, making it difficult to pinpoint correlations between photoelectrode materials changes and PEC *J*–*V* performance.

We review here recent progress on the in situ and operando study of PEC interfaces, highlighting some of the more common techniques that have been used to specifically probe material phenomena as a function of illumination, e.g., charge transfer kinetics, dissolution, structure, oxidation state, composition, and morphology (Figure 30). For more information, a variety of comprehensive reviews are available for understanding the recent progress in and comprehensive technical details of in situ characterization tools and techniques.^10,13,14^ By grouping studies by the dynamic process, aka, material phenomena uncovered during measurement (Figure 30), we hope to provide a tool for researchers to determine the suite of techniques to best understand the behavior and evolution of a photoelectrode and its solution interface, rather than a guide for how to best use a specialized instrument or technique.

**Figure 30:**
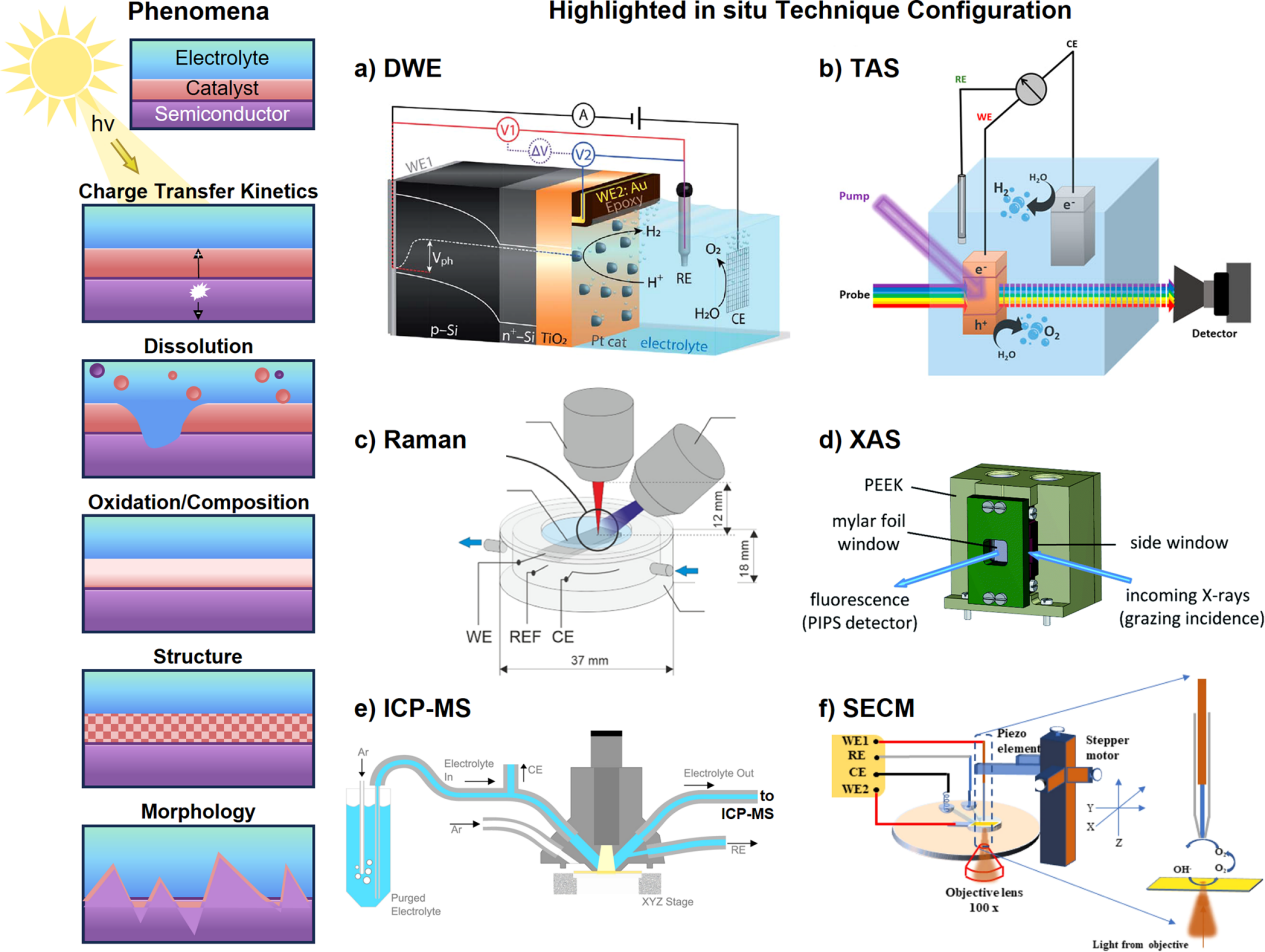
Commonly probed interfacial phenomena in PEC (left); schematics of common in situ techniques for the direct characterization of those phenomena (right). Some optimized configurations that have been used for in situ PEC techniques (a–f). (a) Dual working electrode (DWE) measurements. Adapted with permission from ref 448. Copyright 2018 Royal Society of Chemistry. (b) Transient absorption spectroscopy (TAS). Adapted with permission from ref 449. Copyright 2020 American Institute of Physics. (c) Raman spectroscopy. Adapted with permission from ref 450. Copyright 2022 Elsevier. (d) In situ X-ray absorption spectroscopy (XAS). Adapted with permission from ref 322. Copyright 2022 Royal Society of Chemistry. (e) Online inductively coupled plasma mass spectrometry (ICP-MS). Adapted with permission from ref 451. Copyright 2021 American Chemical Society. (f) Scanning electrochemical microscopy (SECM). Adapted with permission from ref 452. Copyright 2023 Nature Portfolio. This is not a comprehensive list of phenomena or techniques.

For the purposes of this discussion, we provide in the text below standard terms for evaluating material changes. For example, the term *corrosion* has been used colloquially to be synonymous with *degradation*. However, a standard definition of corrosion is the deterioration of a material that results in a chemical or electrochemical reaction with its environment.^453^ Therefore, corrosion encompasses dissolution, oxidation, and possibly even structural changes if they are the results of a reaction, but does not include delamination/detachment or other non-Faradaic, morphology-based degradation or poisoning process.^454^ Thus, for clarity, individual corrosion and degradation phenomena will be discussed separately.

We also provide these rigorous definitions: *ex situ* characterization takes place outside of the electrochemical cell; *in situ* characterization takes place under relevant electrochemical conditions *without* product detection; *operando* characterization takes place under relevant electrochemical conditions *with* product detection. For water splitting, e.g., HER and OER, product detection is often not standard, but has been shown to be important for decoupling activity and other Faradaic processes in electrocatalysis.^455^ Therefore, as rigorously defined, because operando characterization requires a level of product analysis that is often not deployed for PEC water-splitting, we will default to using the more general term in situ. Product detection is an interesting direction for in situ OER and HER PEC work and more critical to other fuel-forming reactions, as highlighted in the following section.

Although the techniques detailed here have often been previously explored for “dark” metallic electrocatalysis, in many cases, their translation to PEC systems is still developing and may not fully capture the behavior of a photoelectrode during full PEC operation. Our primary focus here is on techniques which have already demonstrated utility for direct measurement of PEC systems in operation, that is, in situ techniques which fully incorporate light. One important challenge in this field is developing cells or configurations that allow for the characterization of photoelectrode|electrolyte interfaces with in situ techniques that require ultrahigh vacuum, e.g., TEM or XPS. Specifically, an important direction is understanding the role of illumination, because while illuminated interfaces often bear many similarities to those in dark electrocatalysis, PEC interfaces have a set of requirements that are distinct from standard electrocatalyst performance that must be addressed to study the interface under representative conditions. In particular, each technique must be optimized to enable both white-light illumination of the photoelectrode to ensure realistic PEC operation as well as delivery and collection of the signal for the characterization methods (which may be a synchrotron X-ray, laser pulse, etc.), often with the additional complication of surface catalysts or protection layers at the semiconductor|electrolyte interface (Figure 30a–f).

### 3.1 Highlighted Techniques

A variety of techniques have been used to directly characterize surface and material dynamic phenomena on photoelectrodes in situ. Here, we provide condensed descriptions of a selection of such techniques for reference; more comprehensive and specific reviews of each technique are included in the relevant sections.^456–458^ Note that this is not an exhaustive list of the characterization tools available in the literature, but rather a highlight of some of the more common approaches.

#### 3.1.1 Dual Working Electrode (DWE)

A number of research groups have developed and utilized a DWE technique to measure the electrochemical potential at the photoelectrode surface under operation.^459–461^ The key requirement of the DWE technique is the presence of a conductive surface at the photoelectrode|electrolyte interface, often present as a thin conductive catalyst film or as a heavily doped semiconductor layer.^462^ Contacting this conductive surface allows for the direct measurement of the electrochemical potential at the photoelectrode|electrolyte interface and the disentanglement of the photovoltaic effects occurring in the light absorber from changes in catalyst state (e.g., structural, morphological, oxidation state and composition, dissolution effects) (Figure 30a).^448^ DWE can reveal important insights in PEC performance with respect to photocarrier loss pathways as well as catalytic and photovoltaic performance, giving valuable guidance on which interfaces should be targeted for further spectroscopic interrogation.

#### 3.1.2 Ultrafast Spectroscopies

Typical spectroscopic characterization for the early development of a new semiconductor involves steady state absorptivity and photoluminescence measurements; although these are important to develop a basic material understanding (see Figure 30b above), they are insufficient to describe the effects of trap states and defects in a photoelectrode that can be probed by time-resolved spectroscopies. Time-resolved photoluminescence (TRPL) measures the radiative lifetime of emissive materials and the decay provides information regarding both radiative and nonradiative decay processes, which in semiconductors can include trapping, charge transfer, and charge recombination.^463^ While assumptions can be made about the reasons for luminescence quenching, the data do not reveal the specific mechanisms of carrier quenching without support from other techniques.

Transient absorption spectroscopy (TAS) provides evidence for quenching mechanisms that cannot be gleaned through luminescence measurements. TAS is a pump–probe technique where a femtosecond excitation pulse promotes an electron from the valence band into the conduction band, creating an electron–hole pair (Figure 30b).^464–466^ A probe pulse, often broadband white light, follows, and a difference spectrum between ground and excited states is generated based on the changes in transmission before and after excitation. Both electrons and holes can have spectroscopic signatures that can be monitored through these techniques and their evolution over time reveals nonradiative quenching mechanisms and lifetimes of photogenerated carriers. As discussed below, TAS performed in the absence and presence of electrolyte reveals changes in behavior at the semiconductor|electrolyte interface. Additionally, in situ electrochemical measurements are often incorporated into TAS setups to probe the effects of an applied bias on the photoinduced kinetics for a more direct comparison to device measurements.^467^

Because TAS is an ensemble technique, bulk behavior may dominate the signal, preventing careful analysis of surface dynamics, particularly in emerging semiconductors with high concentrations of crystalline or chemical defects. Transient photoreflectance uses the same pump and probe as TAS, but in the case of transient photoreflectance, the probe is reflected off of the interface of interest into the detector.^468^ The angle of the sample dictates the penetration depth of the pump and probe based on the index of refraction of the material. Through transient photoreflectance, surface trapping or recombination due to defect formation or diffusion from the surface can be monitored. Continued advancements in transient spectroscopic techniques in combination with careful electrochemical and structural and product characterization will provide a complete picture of new semiconductors from the moment of photoexcitation through fuel production.

#### 3.1.3 Raman and IR Spectroscopies

Raman spectroscopy (Figure 30c) is a vibrational spectroscopy based on a scattering process where an incoming photon excites a target molecule or crystal to a virtual state. When the targeted material relaxes, it can relax to a different vibrational energy level resulting in a shift of the scattered emission from the material and often a change in polarizability. Only those vibrations where the polarizability changes are Raman active. In principle, vibrational spectroscopy can provide both qualitative and quantitative detection of all molecules with more than one atom, and inorganic semiconductors generally have a range of Raman active modes that can be probed using this technique.^469^ Furthermore, as these measurements are rapid and the Raman modes for water are weak,^470^ time sensitive analytes like transient reaction intermediates can also in principle be tracked.^469^ One of the major challenges of integrating optical characterization techniques in PEC systems where light plays an integral role in driving the reaction, is the possibility of interference of the light for characterization (in this case, a laser in the optical range) and driving the actual reactions (simulated solar light).^471^ Careful design of experiments in needed to ensure that the Raman probe does not alter the behavior of the PEC system, especially a concern given the high incident powers of lasers needed due to the low yield of the Raman process.

Infrared (IR) spectroscopy is another vibrational spectroscopy, where interaction with the photon results in absorption of light. Because of the low energy (IR range) of excitation, these photons do not result in electronic transitions (like UV–vis) but can couple with vibrational modes resulting in reorientation of the dipoles thus, exciting the molecule to a vibrationally excited state. This leads to attenuation of the IR signal of the transmitted light on the detector, leading to a molecular characteristic signal. IR spectroscopy is more popular for identification and quantification of polar molecules, e.g., with a strong dipole.^472,473^ For the purposes of studying illuminated and electrified liquid interfaces, the most popular IR spectroscopies include a Fourier Transform (FTIR) and an attenuated total reflectance (ATR-FTIR) accessory for surface sensitive identification of adsorbates.

#### 3.1.4 X-ray Absorption and Photoelectron Spectroscopy (XAS and XPS)

X-ray absorption spectroscopy (XAS) is a technique for probing the oxidation state, local coordination, and structure of materials.^474^ In situ XAS performed during electrochemistry has been developing over the past several decades and several different types of XAS based configurations exist for probing dark electrochemical systems.^475,476^ Coupled XAS with illuminated electrochemical systems have been less studied, but several cell configurations exist.^477^ In situ XAS requires a synchrotron (e.g., European Organization for Nuclear Research (CERN) 13 TeV), which has a higher energy and flux than benchtop X-ray sources (e.g., Cu Kα: 8.04 keV^478^) due to the electrolyte necessary to run an electrochemical measurement. A common configuration employs a fluorescence detection mode (Figure 30d) in which the X-ray hits the back of a cell.^7^ By not going through the electrolyte this configuration minimizes the loss of signal due to the aqueous layer. Typically, XAS is a bulk sensitive measurement with the surface changes averaged over the entire signal, however, in some cases the incoming angle can be tuned to enable optimal illumination of visible light and surface characterization (grazing incidence, GI-XAS).^322^

XPS is a very common tool for sensitive ex situ analysis of photoelectrodes to understand composition and oxidation state at the photoelectrode surface (the nominal semiconductor|electrolyte interface). However, due to the ultrahigh vacuum environment required to accurately detect the binding energy of electrons ejected from the surface of the material during XPS, a synchrotron radiation source is required for samples with a liquid interface;^479,480^ and even then, very special geometric considerations must be taken into account. With a liquid environment present, an ultrahigh vacuum is no longer possible. To account for this, ambient-pressure (AP)-XPS techniques, with chamber pressures ranging between 10–110 Torr (relative to atmospheric pressure of 760 Torr) have been pioneered. Because a vacuum chamber is still required, illumination with simulated sunlight for a PEC study is difficult in such a tight chamber. One promising AP-XPS technique, the dip and pull method, has been successfully used to study electrified surfaces of catalyst and/or semiconductor|electrolyte interfaces by dunking a material into electrolyte and strategically removing it partially from the cell so that it forms a thin layer of liquid over the surface that is in contact with a larger reservoir.^480,481^ This technique will become even more valuable for the operando study of PEC systems if consistent geometries enabling illumination of the semiconductor photoelectrode can be designed, such that changes to valence energies of surface species can be directly measured in the dark and in the light on operational photoelectrodes.

#### 3.1.5 Inductively Coupled Plasma Mass Spectrometry (ICP-MS)

Dissolution of photoelectrodes and catalysts can result in performance loss during PEC operation; by carefully probing effluent electrolyte, it is possible to directly measure the rate of dissolution. ICP-MS is an established analytical technique for probing the concentration of most elements from the periodic table in electrolyte. Light elements are generally not directly detectable although in some cases it is possible to complex the light element of interest for evaluation, e.g., F as a polyatomic ion complexed with Ba.^482^ Recently, an electrochemical flow cell was demonstrated that was connected to an ICP-MS and the resultant online ICP-MS measurement detected the real time dissolution as a function of potential or electrochemical condition.^483^ Modifying this system for integration with light can be done in a flow-cell configuration with front illumination (Figure 30e).^451^ While not yet widely utilized in PEC, the online ICP-MS setup is the most direct measurement of dissolution available. The main downsides of this technique for PEC are nonstandard cell geometries and that online ICP-MS measurement can be very harsh on the instrument. Specifically, to maintain proper cleanliness and high analytical resolution, ICP-MS instruments in shared facilities are typically only used to probe samples with dilute acid and would not be usable for >0.01 M acid or alkaline electrolytes common for PEC. Therefore, adapting ICP-MS instruments for online use generally requires researchers to have a dedicated tool that has frequent upkeep, which can be time-consuming and expensive.

#### 3.1.6 Atomic Force Microscopies (AFM)

At its most basic level, AFM is used for probing the surface morphology of a material while SECM spatially probes surface charge transfer.^484,485^ In situ AFM, or electrochemical AFM (EC-AFM) for PEC allows for surface morphology to be probed during illuminated electrochemical measurements. This could be done with an insulating AFM tip and in one of the most basic AFM configurations: contact (original mode for topography) or dynamic tapping (most common currently) mode, in which the tip is oscillating on the surface in a variety of different manners optimized as a function of material being studied. Alternatively to AFM, SECM spatially resolves electron movement/transfer processes on a surface, e.g. electrocatalysis, by using an ultramicroelectrode to scan across the surface. AFM and SECM can be used separately for example to study a surface and disentangle catalytic activity and particle agglomeration.^486^ PEC-SECM using a redox couple, e.g. O_2_, can be used to trace catalysis under directed illumination (Figure 30f). However, AFM and SECM can also be combined with an SECM-AFM probe, e.g., a conductive AFM tip that is a secondary working electrode.^487^ In this case the SECM-AFM probe measures the surface of the electrode electrochemically and can correlate morphological and potential changes along the surface (potential sensing electrochemical AFM, PS-EC-AFM).^488^

### 3.2 Probing Interfacial Photoelectrode Phenomena

Photoelectrochemical charge transfers occur at heterogenous photoelectrode|electrolyte interfaces in which a sufficient electrochemical potential difference has been developed.^489^ Unsurprisingly, these same conditions cause solid catalytic surfaces to undergo transformation of crystallography, morphology, composition, and oxidation state, with corresponding impacts on catalytic performance. Development of fundamental structure–property relationships rely on well-defined and characterized surfaces, where changes to actively catalytic surfaces convolute assessment of their performance. Recent advents in operando and in situ characterization techniques have allowed direct assessment of actively catalytic surfaces, providing new insight into fundamental processes: charge transfer, dissolution, composition and oxidation state, morphology and structure. Notably, because studying phenomena in situ is so complex, the majority of studies focus on developing or tuning methodology to study one specific material with one technique. An important future direction for in situ PEC will be running holistic studies that combine insights from multiple in situ techniques or a wide variety of photoelectrode materials for comparison. Below we outline recent progress on common methods for characterizing illuminated surfaces, including instances of in situ and operando investigations.

#### 3.2.1 Charge Transfer

The *J*–*V* relationship under defined illumination conditions (AM 1.5G, diurnal conditions, in the dark, etc.) is the basic descriptor of a PEC system and dictates η_STF_ conversion.^490^ Thus, understanding the factors that influence the *J*–*V* relationship in principle offers a path to rationally improved performance. However, one of the major challenges in understanding the *J*–*V* relationship of a PEC system arises from the complexity associated with development and characterization of an integrated unit capable of simultaneously driving both photovoltaic action and catalysis while also maintaining stability. For example, considering the *J*–*V* behavior of novel photoelectrodes with the various architectures shown in Figure 31, it may be initially unclear to what extent “nonideal” performance can be attributed to limitations due the semiconductor, the catalyst, and/or their associated interfaces.

**Figure 31:**
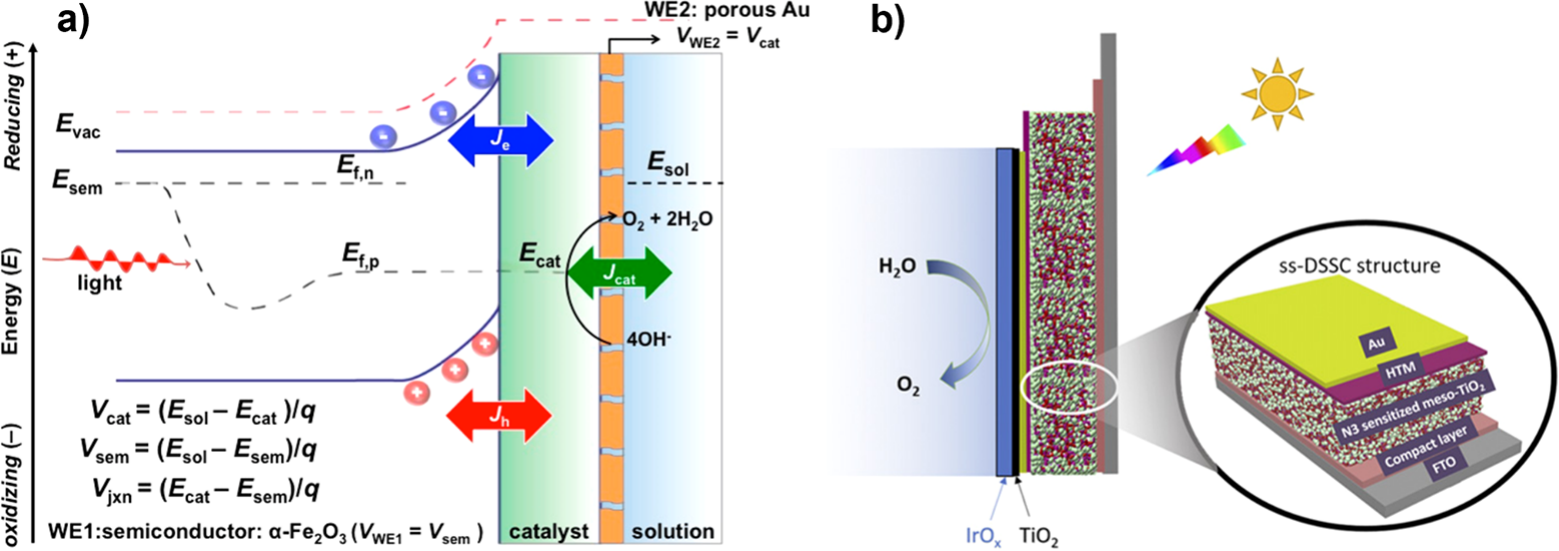
(a) A combination of interfaces between semiconductor, catalyst, metallic contact, and electrolyte can be used to interrogate and quantify charge transfer paths and photocarrier loss effects during PEC operation. Adapted with permission from ref 459. Copyright 2017 American Chemical Society. (b) The nature of a junction (e.g., adaptive vs buried; the effect of electrolyte on photovoltaic performance) in a solar fuels device (a dye-sensitized solar cell) can be quantified readily using DWE for structures that include accessible metallic contacts. Adapted with permission from ref 495. Copyright 2018 United States National Academy of Sciences.

Many (photo)­electrochemical techniques are used to probe *J*–*V* behavior and understand these limitations, from observing current density at constant potential and illumination to characterize stability, to varying illumination to identify current density derived from photogeneration, to scanning probe techniques to understand spatial variations in structure and charge transfer behavior.^13^ EIS techniques use varying potential and/or current waveforms to provide a range of insights; EIS techniques are most commonly represented in the field by “Mott–Schottky”-type analyses used to estimate flat band potentials, dopant densities, band edge positions, and interfacial charge structure.^74^ The complexity of experimental execution and data analysis when using EIS methods is often high and must be taken with care.^43,44,49^ Recent studies have used careful EIS measurements to gain new insights into the nature of accumulation region formation at photoelectrode|electrolyte interfaces, and advanced techniques have coupled varying light intensity with high frequency potential waveforms to understand the complex interactions of semiconductors and surface layers.^48,491^ The number of (photo)­electrochemical techniques employed to understand *J*–*V* behavior is large; enumerating all of them is beyond the scope of this work, but they have been highlighted, collated, and reviewed many times by various authors.^13,14,39,492–494^

The electrochemical potential of electrons (and holes) at the electrode|electrolyte interface is a foundational descriptor for understand many of the *J*–*V* behaviors of a photoelectrode, influencing charge transfer rates both to valuable and deleterious reactions at the interface. One key difference between a “metallic” electrochemical and PEC measurements is the ease with which one can directly measure an electrochemical potential at the electrode surface in contact with electrolyte. For standard electrochemical measurements, it is often assumed that the electrons within the conductor are approximately in equilibrium and thus the electrochemical potential at the electrode surface can be effectively measured from a “back contact”. The situation at a photoelectrode surface is more complex, illumination of the photoelectrode can generate nonequilibrium and spatially varying concentrations of electrons (and holes), complicating the relationship between the electrochemical potential at ”front” and “back” contacts of a photoelectrode.

DWE has been used to understand the nature of charge transfer between α-Fe_2_O_3_ or Si and an electrolyte permeable NiFeO_*x*_ catalyst layer, parsing out the effects of varying catalyst oxidation state on the semiconductor|catalyst junction as well as measuring the extent of hole transfer to the catalyst versus direct water oxidation by the Fe_2_O_3_ surface and transient current effects.^459,460^ DWEs have further been used to characterize the photovoltaic activity of photoelectrodes under diurnal CO_2_ reduction conditions, reinforcing the importance of recognizing PV and catalyst component interactions to predict selectivity under varying conditions.^461^ The DWE concept has been used to demonstrate the relationship between a p–n junction Si|TiO_2_|Pt interface and loss of fill factor with time to catalyst morphology changes as well as to reveal that loss of PEC performance in a Cu_2_O|Ga_2_O_3_ photoelectrode was the result of changes in photovoltage rather than corrosion at the electrolyte interface.^448^ It has also been used to evaluate the extent to which a solid state dye-sensitized solar cell exhibited ‘buried junction’ versus adaptive junction behavior during light-driven water oxidation, highlighting both the advantages of the DWE measurement and the ability to execute it when the appropriate conductive layer is present.^495^ These demonstrations highlight the ability of the DWE measurement to explain observed *J*–*V* behavior as a function of the specific components and interfaces present in a photoelectrode. Parsing these component effects via DWE and identifying operative photogenerated charge carrier, catalytic, and degradation pathways are invaluable to inform the rational design of PEC devices. As a result, expansion of DWE techniques to more photoelectrode systems will be very valuable in the future.

The origins of electrochemical behavior can also be identified by transient spectroscopic techniques. For example, work on β-Mn_2_V_2_O_7_ (β-MVO) used TAS to uncover the reason for poor photoactivity in PEC measurements.^496^ The spectroscopy revealed that β-MVO undergoes ultrafast hole trapping and surface recombination that prevents a majority of carriers from living longer than picoseconds, far shorter than the lifetimes necessary for photochemical transformations. This was confirmed by performing TAS experiments with electron and hole scavengers to ensure that the spectroscopic assignments were accurate. Combining spectroscopy and electrochemistry in the same measurement is even more impactful in correlating device measurements with photophysical mechanism. This is a useful approach in steady state measurements for identifying spectral signatures and in transient spectroscopy applications for changes in carrier lifetimes at varying applied potentials.^497^ Recreation of PEC conditions while performing transient spectroscopy offers a direct handle on photophysical mechanisms contributing or detracting from performance, providing feedback on design principles that can be modified in future iterations.

#### 3.2.2 Dissolution

One of the most robust measurement techniques for indirectly probing dissolution is ex situ XPS in which elemental ratios of species can be compared prior to and after electrochemical analysis. In this case, only ratios can be easily compared; therefore, if components dissolve stoichiometrically it could be difficult to determine if there are not distinct layers. One major limitation any type of pre- and postcharacterization is the lack of time or condition resolution for the dissolution process, which can be resolved, to some extent, by in situ characterization.

To measure dissolution in real time an inductively coupled mass spectrometer (ICP-MS) was connected to a PEC cell and the dissolution of W in WO_3_ photoanodes was monitored as a function of potential and light exposure.^451^ Max W dissolution was seen to coincide with onset of the saturation photocurrent and at 1.2 V_RHE_ the W dissolved less in the dark vs in the light. In a system irradiated at a fixed wavelength, WO_3_ powder on Au was analyzed in sulfuric acid and the dissolution was directly related to anodic photocurrent (Figure 32).^190^ A BiVO_4_ photoanode was tested in pH 7 citrate buffered solution and showed a potential and light independent preferential contact dissolution of V which was hypothesized to be due to a charge-neutral (chemical process) reaction of V_2_O_5_ in water.^498^ In relation to PEC, stoichiometric amounts of Bi and V dissolution was seen with more dissolution seen in the light with higher photocurrents. Dissolution was hypothesized to be due to PEC Bi­(III) oxidation, which leaves V_2_O_5_, which subsequently dissolves. This work used the time resolution capabilities of online ICP-MS to explain previous ex situ results on preferential V leaching by decoupling dissolution mechanisms that were PEC vs contact related. In another example, by probing ZnO photocathodes in acidic and alkaline conditions, stabilization of Zn was shown as a function of surface facet in which the ZnO (0001) was less stable than the inactive (101̅0) and all facets were stabilized during HER vs OER.^499^ In a study focused on IrO_*x*_, the Ir-modified WO_3_ photoanodes demonstrated the interplay between dissolution stabilization and light blocking with a thin layer of IrO*_x_* that simultaneously decreased dissolution and light saturation.^500^ Apart from online ICP-MS, dissolution can also be probed indirectly. Looking at a model BiVO_4_ system, PEC-ATR-FTIR spectroscopy showed preferential V dissolution from an illuminated surface at OCV, while with the application of a positive bias and illumination, both Bi and V dissolve.^472^ Overall, while both ex situ and indirect methods of dissolution characterization are possible, online ICP-MS has shown itself to be one of the most useful tools for the real-time measurement of material dissolution. Moving forward in developing enhanced tools for understanding dissolution, it will be important to focus on developing an array of device geometries that can feed into the online ICP-MS, because although the initial studies have paved the way for this promising technique, having PEC cells for online ICP-MS that can be tested in more realistic conditions is critical.

**Figure 32:**
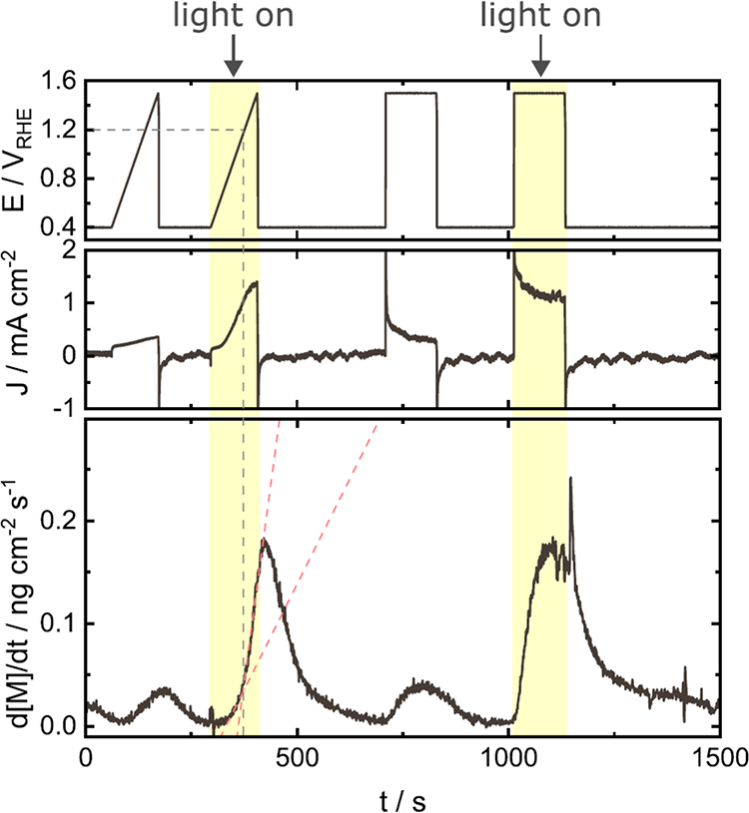
Dissolution of tungsten from WO_3_ observed with online ICP-MS with concurrent on/off illumination (1 Sun AM 1.5G) (top), resultant current (middle) and W dissolution rate (bottom). Adapted with permission from ref 451. Copyright 2021 American Chemical Society.

#### 3.2.3 Composition and Oxidation Changes

Similar to dissolution, oxidation state or composition changes from impurity accumulation on the electrode surface can be measured ex situ. XPS is one of the most robust methods for impurity analysis, but in cases where photoelectrode surfaces are air sensitive (e.g., nitrides that easily oxidize^501^) in situ characterization is imperative for determining true surface oxidation or reduction. To probe oxidation for example, operando infrared spectroscopy,^473^ (ATR-IR) can be employed and researchers noted oxidation via a peak associated with high valent (Fe­(IV)=O) species during PEC water-oxidation. In situ microphotoelectrochemical surface-enhanced Raman spectroscopy (SERS) and ambient pressure XPS have also been employed to look at oxidation, and Au–OH^+^ species were identified during PEC water oxidation in acid (0.1 M HClO_4_) on a gold microelectrode.^502^

XAS is one of the most common methods for determining oxidation state change in situ and is commonly used to understand ex situ oxidation states of photoelectrodes.^503^ While in situ electrochemical XAS measurements can be readily adapted to incorporate light, there are only limited examples in the literature. For example, in an in situ analysis of the W L_III_-edge, WO_3_ photoanodes (5 mW 400 nm LED, in 0.1 M Na_2_SO_4_) were analyzed by examining the edge shift and growth of the white line peak with illumination; the authors hypothesize that illumination fills the t_2g_ W orbitals and drives structural rearrangement.^504^ In another study examining an α-Fe_2_O_3_ photoanode decorated with a NiO_*x*_ catalyst, shifts in the Ni K-edge under illumination and applied potential indicated that holes photogenerated in α-Fe_2_O_3_ are responsible for Ni oxidation (>Ni^IV^).^505^ Similarly, the oxidation state of an Mn catalyst has been extensively studied via the Mn K-edge with a general agreement that Mn oxidizes under illumination on SrTiO_3_-based photoelectrodes.^506,507^ Interestingly, this trend of oxidation during catalysis extends to Ir: in a study of IrO_*x*_ on a Si p^+^-n junction light absorber, high-energy-resolution fluorescence detection (HERFD) was used to track the Ir oxidation state through the white line intensity of the Ir L_III_ edge.^508^ Linear oxidation of the Ir was seen as a function of applied potential in the dark and under illumination until the onset of OER at which point a plateau or reduction in the Ir oxidation state was seen on thicker (2 – 3 nm IrO_*x*_) and thinner (1 nm IrO_*x*_), respectively (Figure 33). Alternatively, however, examining Ta L_3_ and Sr K edges of SrTaO_*x*_N_*y*_ semiconductors the Ta oxidation state was shown to be independent of potential, while a reduction of Sr is seen with increasing potential which is correlated with leaching.^322^

**Figure 33:**
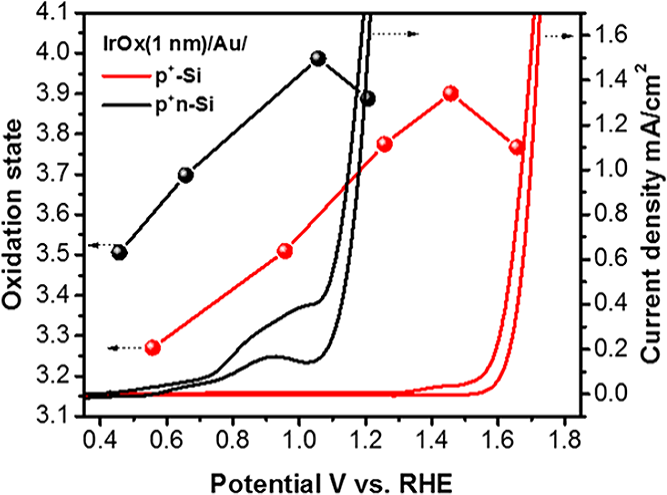
In situ monitoring of the oxidation states with in situ XAS of IrO_*x*_ showing calculated Ir oxidation states from L-edge in the dark (red) or under AM1.5 illumination (black) with corresponding *J*–*V* curves. Adapted with permission from ref 508. Copyright 2019 American Chemical Society.

Notably, one of the most difficult parts of tracking oxidation state with XAS, and especially using a simple analysis of the absorption edge, (X-ray absorption near edge structure, XANES) is that while the edge position and shape can be sensitive to the oxidation state, other factors including ligand identity can dramatically impact edge shape and position. For example, in a non-PEC study focused on assigning Ga oxidation states, the edge position between Ga­(I) and Ga­(III) was determined to be indistinguishable.^509^ This is an important lesson for XAS analysis of photoelectrode transformations: it is imperative that oxidation state assignments are made rigorously, and include clear comparisons to well-defined standards (e.g., linear combination fits), simulated spectra using an appropriate atomic model, and/or coanalysis with other techniques.

#### 3.2.4 Structure and Morphology Changes

Surface structural changes can be difficult to probe because common structural characterization techniques, such as XRD, require the resultant structure to be crystalline or ordered, rather than amorphous. In fact, structural changes to amorphous species or with small crystallites would be XRD transparent. X-ray reflectance (XRR) can be used in these cases where a thin amorphous surface layer forms; however, XRR is not chemical specific, so models are necessary to correlate the measured signal (“fringes”) to the layers in a photoelectrode. Another method to probe crystalline surfaces transforming to amorphous is Raman spectroscopy which can be used to probe structure changes if the material has Raman active vibrational modes.^510^ For example, analyzing the surface of a Ti|TiO_2_|Prussian Blue photoanode with in situ Raman indicated that reoxidation of the surface was only seen with photocurrent from the TiO_2_ and not just with applied potential.^450^ Furthermore, the oxidation process was shown to be time-dependent (>40 s). However, it is important to note that it can be difficult to distinguish how much photoexcitation is from the Raman laser vs illumination, as in situ work on BiVO_4_ has previously shown laser-induced phase transition from an amorphous phase to scheelite monoclinic structure.^511^ Ex situ scanning tunnelling microscopy has been used to examine the role of structure and to demonstrate the importance of the Bi:V ratio in comparable BiVO_4_ surface facets, with Bi rich surfaces leading to improved photocurrent density.^512^ While scanning tunnelling microscopy (STM) or TEM could theoretically also be used for structural characterization, coupling electrochemical processes, illumination, and TEM chambers has proven difficult.^513–515^ Developing experimental systems that allow for simulated solar illumination during such measurements, similar to the discussion for AP-XPS above, could enable improved understanding of photoelectrode behaviors in situ.

Morphologically, strategic ex situ analysis can be used effectively for correlating electrochemical conditions to surface changes. Unlike oxidation state, large topographical changes are less air sensitive and often withstand removal from electrolyte. Outside of standard AFM for morphology, neutron reflectometry (NR) can also add value in observing changes in the electrolyte ordering as well as interface layer changes. Looking at LaTiO_*x*_N_*y*_ using ex situ NR demonstrated structural changes in LaTiO_*x*_N_*y*_ photoelectrodes.^501^ Although ex situ morphology is fairly robust, it is very labor intensive to determine potential or time-based morphology changes. In situ EC-AFM on materials such as BiVO_4_ have been used to correlate dissolution to specific locations on the electrode and create a mechanism for dissolution as a function of topography.^516^ Scanning PEC microscopy of BiVO_4_, Mo-doped BiVO_4_, and TiO_2_ (0.1 M phosphate buffer, ranging pH 7.0–8.5) has been used to correlate morphology and performance.^517^ Optical microscopy has been used to track time-resolved degradation via defects in the images of a GaInAsP|GaAs tandem device.^372^ In an effort to correlate morphological and structural changes, photo-SECM has also been performed on a TEM grid (Figure 34).^518^ Using this technique, researchers were able to measure water oxidation current on a single illuminated ∼8 μm TiO_2_ nanorod, showcasing this technique’s resolution for studying individual small particles. Overall, in situ morphology techniques are useful because they can resolve time and condition (light, potential, etc.) phenomena that would otherwise be too labor intensive to assess. However, where in situ characterization becomes very important is with the direct mapping of electrochemical processes, as well as topography (e.g., Figure 34). Moving forward, it will be very important to continue improving the resolution and measurement speed of these processes, so that even smaller features can be analyzed quickly.

**Figure 34:**
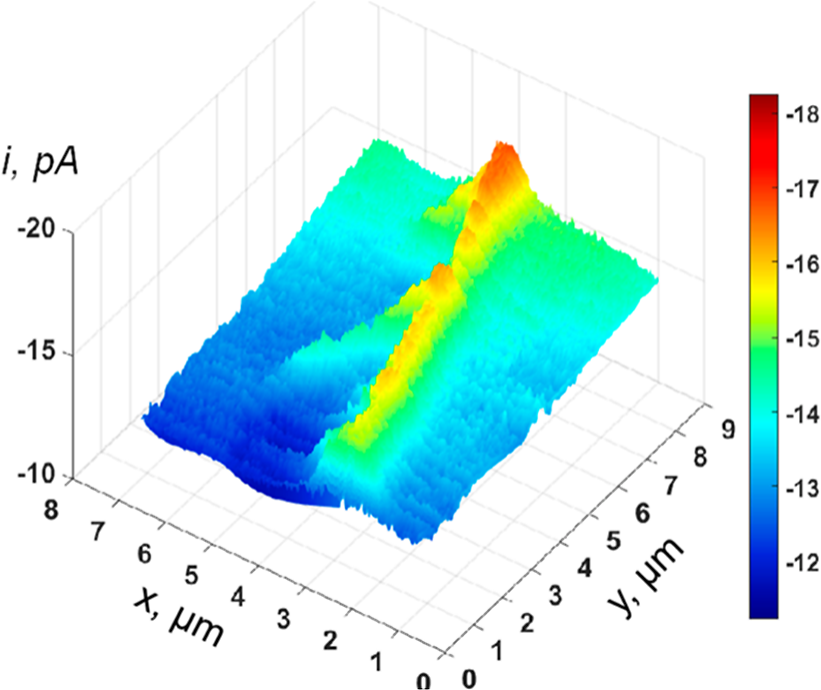
Electrochemical morphology using photo-SECM on a TEM grid of a TiO_2_ nanorod under illumination (200 W HgXe) with the SECM tip at −1.3 V vs ground and the TEM grid/substrate at 0.6 V vs Hg/Hg_2_SO_4_ with dissolved O_2_ in solution. Adapted with permission from ref 518. Copyright 2021 American Chemical Society.

## 4 Alternative Photoelectrochemical Fuel-Forming Reactions

We now turn to applications of photoelectrodes to PEC fuel-forming reactions beyond HER and OER, which are given in Table 1. Solar-driven generation of multicarbon chemicals from CO_2_RR, or ammonia from N_2_RR, would derive those chemicals from inert atmospheric feedstocks like CO_2_ and N_2_. Multielectron reduction reactions, however, present additional challenges in selectivity (Figure 35), both in terms of the array of reduction products and competition from the kinetically facile HER. C_n_ products (where *n* > 1) are the most desired products of CO_2_RR, but the C–C coupling step is especially difficult and examples of PEC systems capable of such transformations are rare.^17^ Similarly, adsorbing nonpolar N_2_ and then destabilizing the strong N≡N bond is challenging, resulting in low selectivity for N_2_RR for many photocathodes, especially in aqueous systems.^18,519^

**Table 1:** Standard Potentials and Half-Reactions for the Alternatives to HER and OER Discussed in This Section

Cathodic Half-Reactions				
HER	acid^a^	2H^+^ + 2e^–^ → H_2_	0.0 V_SHE_	^30^
	alkaline^b^	2H_2_O + 2e^–^ → H_2_ + 2OH^–^	–0.828 V_SHE_	^30^

CO_2_RR processes	pH 7	CO_2_ + 2H^+^ + 2e^–^ → CO + H_2_O	–0.52 V_SHE_	^30^
	pH 7	CO_2_ + 6H^+^ + 6e^–^ → CH_3_OH + H_2_O	–0.40 V_SHE_	^520^
	pH 7	CO_2_ + 8H^+^ + 8e^–^ → CH_4_ + 2H_2_O	–0.25 V_SHE_	^520^

N_2_RR	acid	N_2_ + 8H^+^ + 6e^–^ → 2NH_4_^+^	0.057 V_SHE_	^521^
	alkaline	N_2_ + 6H^+^ + 6e^–^ → 2NH_3_	–0.734 V_SHE_	^521^

NO_3_^–^RR	acid	NO_3_^–^ + 10H^+^ + 8e^–^ → NH_4_^+^ + 3H_2_O	0.88 V_SHE_	^522^
	alkaline	NO_3_^–^ + 6H_2_O + 8e^–^ → NH_3_ + 9OH^–^	–0.12 V_SHE_	^522^

Anodic Half-Reactions				
OER	acid	2H_2_O → O_2_ + 4H^+^ + 4e^–^	1.23 V_SHE_	^30^
	alkaline	4OH^–^ → O_2_ + 4e^–^ + 2H_2_O	0.40 V_SHE_	^30^

GOR processes	acid	C_3_H_8_O_3_ → C_3_H_6_O_3_ + 2H^+^ + 2e^–^	1.36 V_SHE_	^30^
	acid	C_3_H_8_O_3_ + 3H_2_O→ 3CO_2_ + 14H^+^ + 14e^–^	0.003 V_SHE_	^523^

halide oxidation	acid	2Cl^–^ → Cl_2_ + 2e^–^	1.36 V_SHE_	^30^
	acid	3I^–^ → I_3_^–^ + 2e^–^	0.54 V_SHE_	^30^

a By convention,^30^ pH is 0 for acidic electrolytes.

b By convention,^30^ pH is 14 for alkaline electrolytes.

**Figure 35:**
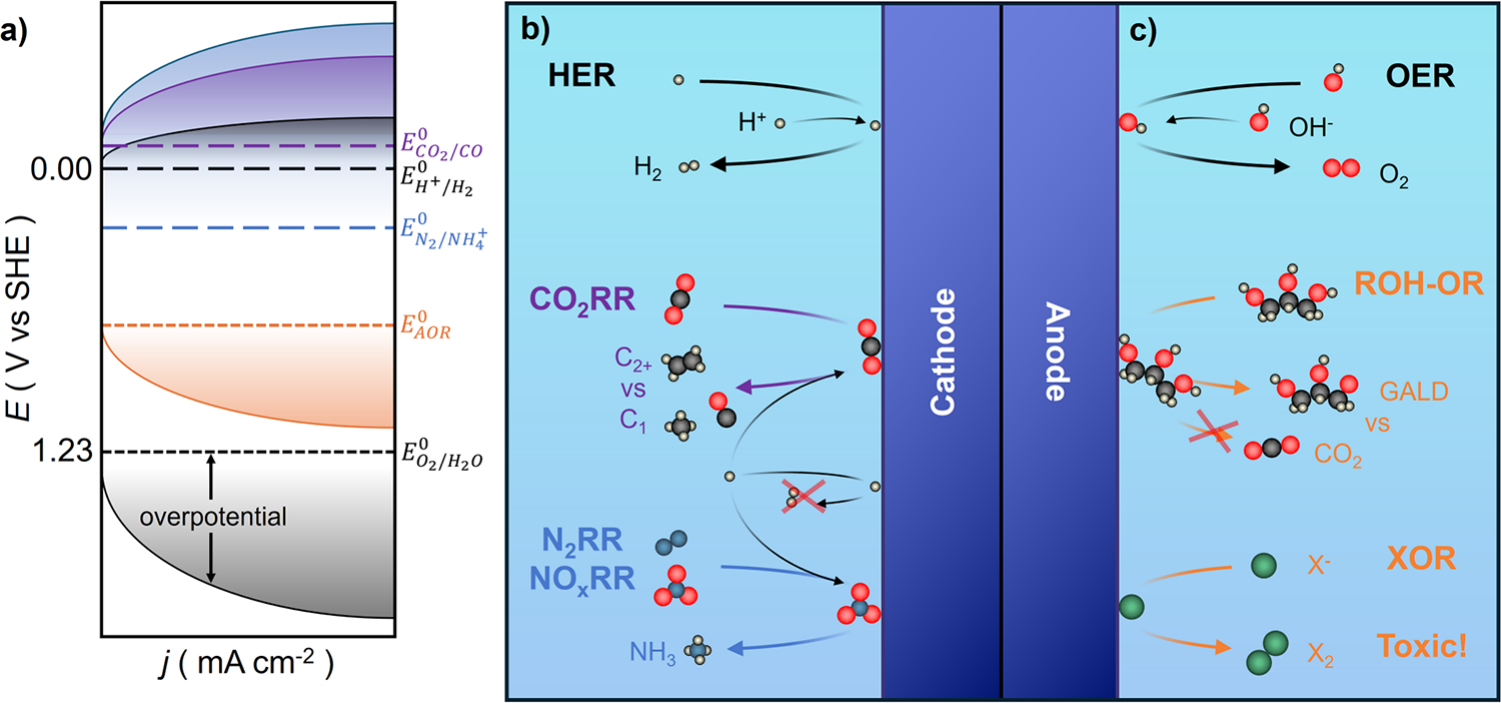
(a) Hypothetical nonilluminated (“dark”) electrocatalytic *J*–*V* response (thin traces with gradients) and equilibrium electrochemical potential of cathodic (long dashes) and anodic half-reactions (short dashes) discussed in this section, where AOR indicates alternative oxidation reactions. (b) Schematic illustrations of cathodic fuel-forming reactions and their key challenges. CO_2_RR, N_2_RR, and NO_3_^–^RR require protons to undergo proton-concerted electron transfer reactions and must address selectivity issues (e.g., C_2+_ product formation for CO_2_RR) while inhibiting the kinetically facile coupling of protons and electrons for HER. (c) Schematic illustrations of alternative oxidation reactions, which face less of a selectivity challenge against the sluggish OER, although oxidations of alcohols (such as glycerol oxidation, GOR) must selectively partially oxidize products to value-added products, while halide oxidations (XOR) yield highly corrosive products, introducing severe safety considerations.

The thermodynamic energy requirements and η_STF_ of fuel-forming reactions are additionally impacted by their coupled oxidation. Just as water splitting is limited by the kinetically difficult OER, so are CO_2_RR and N_2_RR in aqueous environments where OER is the default oxidative half-reaction. As discussed previously, OER has large kinetic barriers, requiring two water molecules to undergo four proton-coupled electron-transfer steps per O_2_ molecule turnover. Replacing OER with an alternative reaction, such as oxidation of an alcohol^524^ or a halide,^525^ can reduce the required whole-cell potential and/or improve kinetics (with fewer, kinetically easier reaction steps), and possibly allow for the production of value-added products at a photoanode (Figure 35).

In this section, we highlight recent advances for PEC CO_2_RR, N_2_RR, and alternative oxidation reactions, with an aim to elucidate overlaps with and differences from PEC water splitting with respect to the photoelectrodes needed to drive these reactions. Generally, the electrochemical conditions required to drive these reactions efficiently are different from HER or OER. For instance, CO_2_RR benefits from operation in neutral electrolytes, where selectivity toward the desired reaction is increased by limiting competition with HER as the concentration of hydronium ions decreases,^526,527^ which influences semiconductor selection for photocathodes. We also note that when reaction selectivity is at play, PEC systems may provide an advantage over coupled PV-EC systems operating at their maximum power points.^528^ Potential-dependent selectivity for e.g. C_2+_ products over H_2_ or valorized glycerol products over O_2_ can provide an advantage to PEC where the operating conditions are more adjustable than coupled PV-EC.

### 4.1 Carbon Dioxide Reduction (CO_2_RR)

PEC reduction of atmospheric CO_2_ has arisen as a promising source of fuels and chemical feedstocks such as carbon monoxide (CO), methane (CH_4_), formic acid (HCOOH), and ethylene (C_2_H_4_).^17^ However, activation of the inert CO_2_ molecule with minimal energy input remains a substantial challenge for PEC processes. The first step of activation involves injection of one electron into the CO_2_ π system, breaking linearity and severely destabilizing the molecule;^529^ the difficulty of this process is reflected in the highly negative *E* ^0^ of −1.90 V_SHE_ (Figure 36). Bypassing the high barrier for one electron reduction is imperative for efficient activation of CO_2_.^530^ For the series of C_1_ CO_2_RR products, as more electrons are fed to the molecule the global standard reduction potential for the process decreases, allowing more electron-rich products such as CH_4_ and CH_3_OH to be achieved with smaller energy inputs. C_2+_ products do not follow as neat a trend, but the majority require eight or more electrons with *E* ^0^ between those of CH_3_OH and CH_4_.^531^ Regardless of product, the theoretical reduction potentials for all PEC CO_2_RR necessitate use of photoelectrodes with high (very negative) conduction band energies.

**Figure 36:**
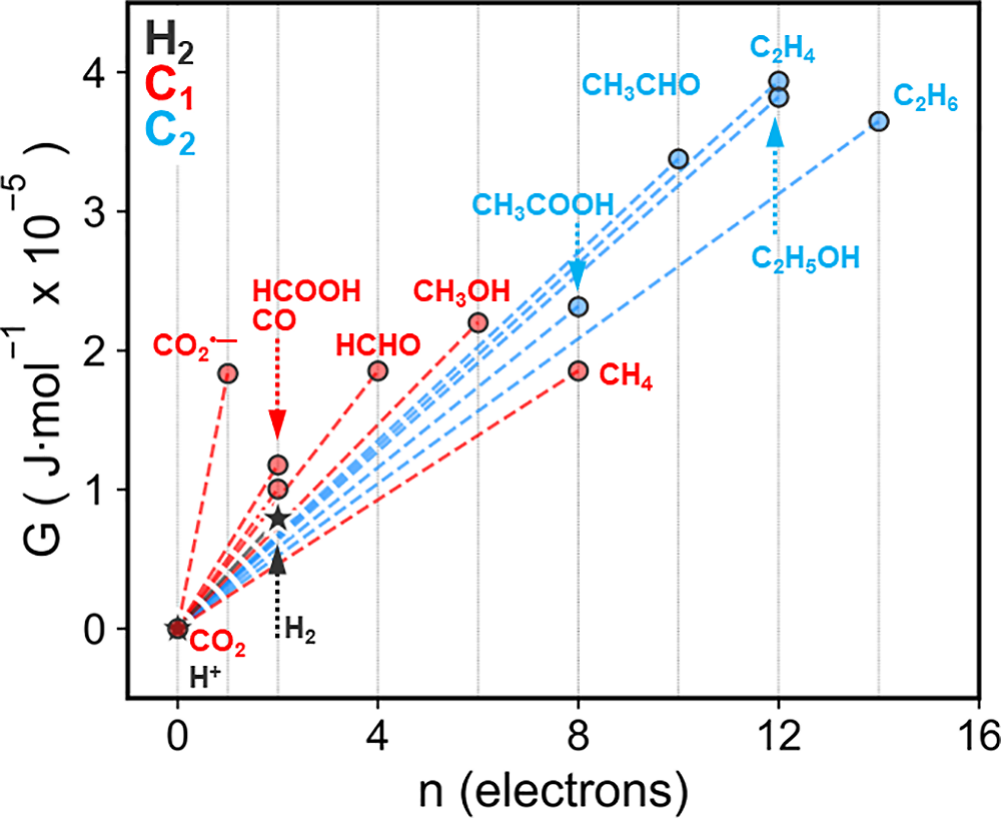
Free energy diagram for CO_2_ reduction to C_1_ (red) and C_2_ (blue) species, including the reduction of H^+^ to H_2_ for comparison (black star). The slope of the lines between points is the overall reduction potential for that process, with shallower slopes representing electrochemically “easier” total conversions. Some unstable intermediates are also illustrated, e.g., the CO_2_^•–^ radical anion is a high energy intermediate between CO_2_ and CO. Values are derived from literature *E* ^0^ at pH 7 ^531^ are presented in SI Table S9.

The diversity of possible products presents a generalized selectivity challenge for CO_2_RR, which is compounded by competition with HER in aqueous systems, as the two-electron HER is kinetically facile compared to CO_2_RR and CO_2_ is poorly soluble in water.^532–534^ Thus, control over not only the product distribution, but suppression of competing HER is vital for the realization of effective PEC CO_2_RR. There has been substantial progress toward promoting and selectively controlling C*_n_* product formation in dark electrocatalysis,^535–537^ but strategies such as electrolyte engineering have yet to be fully adopted in PEC systems.^538^ Similarly, specific functions of PEC systems, such as wavelength-dependent product selectivity, have yet to be pursued for CO_2_RR. It is common to report photon conversion as a function of wavelength in PEC CO_2_RR; however, relevant variations in product selectivity are not often reported and trends are not known, although they are beginning to be investigated for some materials.^539^ Thus, generalized strategies for tuning product specificity toward the more valuable C*_n_* outcomes would be a major step forward for the field. One recent modeling study highlights a paradigm wherein catalysts operating at a high current density near the potential required to achieve C_2+_ products, rather than at the maximum stable current density (as might be targeted for HER), are most desirable for CO_2_RR selectivity.^528^

Beyond the potential-dependent paradigm, several trends have arisen in PEC CO_2_RR. Many of the design principles for HER and OER photoelectrodes have been readily adapted with adjustments to catalysts in order to facilitate CO_2_RR (or suppress HER) and with a larger range of available photoelectrodes as CO_2_RR is generally performed in near-neutral conditions;^540^ only a small selection is presented here. In particular, Au,^541,542^ Sn,^543,544^ and Cu^528,545^ have proven excellent at electrochemical conversion of, respectively, CO_2_ to CO, CO_2_ to formate, or CO_2_ to multicarbon products (C_2+_), and thus are often featured as catalysts in PEC studies. As with HER, the sheathing of delicate surfaces with robust layers such as TiO_2_ or ZnS is widely utilized, not only for protective effects but also to modulate interfacial electronics and charge distribution.^546^ Nanostructuring photocathodes is a popular and effective strategy for enhancing surface area, trapping light in complex surface morphologies, and exposing catalytically active edge and/or facet sites for direct CO_2_ reactivity.^547^ Plasmonically-derived hot charge carriers have been popularized as an effective method for inducing electron build up at the catalyst surface using low energy visible or near-IR irradiation.^548^ Our discussion on PEC CO_2_RR will cover recent advancements in the development of photocatalytic platforms, with an eye on chemically oriented, rational design of advanced photocathodes.

Photocorrosion remains a significant source of photoabsorber degradation in PEC CO_2_RR. As described in section 2.3.1, Cu_2_ZnSnS_4_ is a well-studied kesterite semiconductor; swapping Sn for Ge affords a new semiconductor, Cu_2_ZnGeS_4_, with a similar valence band maximum but a significantly higher energy conduction band minimum.^549^ This conduction-band-derived increase in band gap results in higher energy photoexcited electrons that are capable of overcoming the CO_2_RR energy barrier more readily and leads to higher CO_2_ reducing PEC activity. ZnS was found to be an effective protection layer for Cu_2_ZnGeS_4_, with a combination of ZnS active site incorporation, defect passivation, and/or band-edge tuning proposed as the mechanism for PEC CO_2_RR enhancement. The best reported CO_2_RR results showed the Cu_2_ZnGeS_4_|ZnS photocathode producing 0.28 μmol of CO after 2 h at an applied potential of –0.2 V_RHE_ with a 3.3% η_F_ (Figure 37). Cu_2_O, which has an attractive ∼2.0 eV band gap for CO_2_RR, also requires protective layers. A mixture of Sn and SnO_*x*_ was deposited on p-Cu_2_O with a Ga_2_O_3_ electron-transport layer and a TiO_2_ protective layer. This photoelectrode drove CO_2_RR at 0.44 V_RHE_, with *J* ∼ 0.5 mA cm^–2^ and η_F_ ∼ 60% for CO and HCOOH combined.^550^ Protecting a Cu_2_O based photoelectrode with SnO_*x*_, a Ga_2_O_3_ electron-transport layer and TiO_2_ capping layer produced a mixture of CO and formic acid with ∼60% FE at ∼0.5 mA cm^–2^.^550^

**Figure 37:**
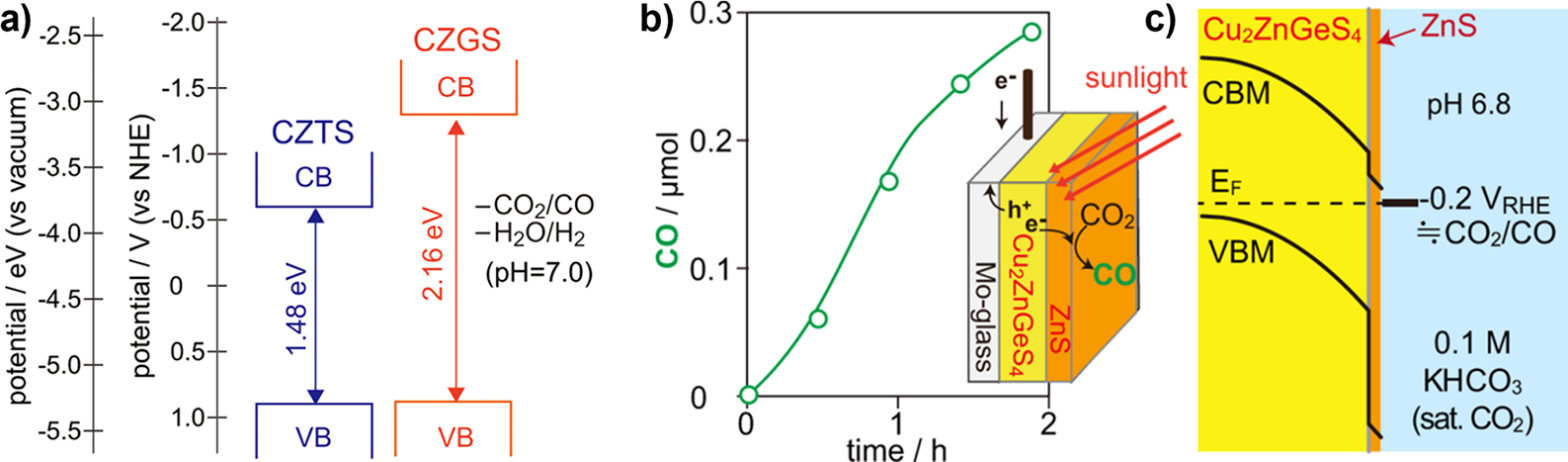
(a) Substitution of the Sn in Cu_2_ZnSnS_4_ (CZTS) for Ge, producing Cu_2_ZnGeS_4_ (CZGS), results in a relative increase in conduction band minimum (CBM) with little to no change in valence band maximum (VBM). (b) CO production by CZGS layered with ZnS and (c) band edge effects of ZnS passivation layer. Adapted with permission from ref 549. Copyright 2019 American Chemical Society.

As with PEC water splitting, nanostructuring photoelectrodes is popular for PEC CO_2_RR as it affords exceptionally high surface areas and the ability to improve optical absorption via light trapping. An effective strategy is to deposit catalytic nanoparticles onto a semiconductor nanorod array to couple catalytic and light harvesting activities. Given the importance of CO_2_RR product selectivity, in-depth mechanistic insights into the catalytic action of photocathodes are vital, as in a recent study of a nanorod InP|TiO_2_|Au photocathodes.^551^ Low-defect InP nanorods, generated by top-down etching of a commercial wafer, were coated in a thin protective layer of TiO_2_, followed by deposition of Au nanoparticulates. The InP photoabsorption was enhanced in the rod-like substructure, with long photocarrier lifetimes due to the lack of defect-dependent recombination pathways. The Au-TiO_2_ interface was computationally proposed to induce a positive charge on the basal layers of Au, increasing the binding strength of adsorbed CO_2_RR intermediates and enhancing overall PEC CO_2_RR. The overall product distribution between CO and H_2_ on the InP|TiO_2_|Au photocathodes was shown to be potential dependent with highest selectivity for CO at −0.11 V_RHE_ (84.2% FE) under one sun illumination and with current densities of 2 to 4 mA cm^–2^ over 4,000 min (Figure 38a–c).^551^ A similar strategy for controlling the CO:H_2_ ratio for PEC CO_2_RR was displayed on GaN nanowires with Au nanoparticles of various sizes with an underlying Si homojunction providing substantial photocurrent.^552^ Smaller nanoparticles (3 nm) provided the best overall current densities at catalytic potentials. However, H_2_ was the main product (>95% FE) with poor selectivity toward CO production from CO_2_RR (<5% FE). When larger Au nanoparticle catalysts (16 nm) were constructed, the overall current density dropped due to a decrease in available surface sites; product selectivity for CO was increased to just over 50% FE with roughly double the partial current density for CO (Figure 38d). The change in performance was explained by DFT: CO binds strongly to edge and corner sites (the dominant surface positions for small nanoparticles) poisoning the catalyst and limiting CO evolution. Alternately, large nanoparticles are comprised mostly of surface facet sites that release bound CO readily, enhancing CO_2_RR to CO.^552^ Similarly, the catalyst-on-GaN-nanorod system for combined CO and H_2_ generation was controlled by free energy tuning of a AgX (where X is a halide atom) catalytic layer. AgBr provided the best selectivity for CO_2_RR to CO at −0.4 V_RHE_ (82% FE for CO) followed closely by AgCl at the same potential (81% FE for CO). DFT studies were used to propose a mechanism wherein stabilization of a bound *COOH in the AgBr and AgCl photocathodes was responsible for the enhanced catalytic activity (Figure 38e).^553^

**Figure 38:**
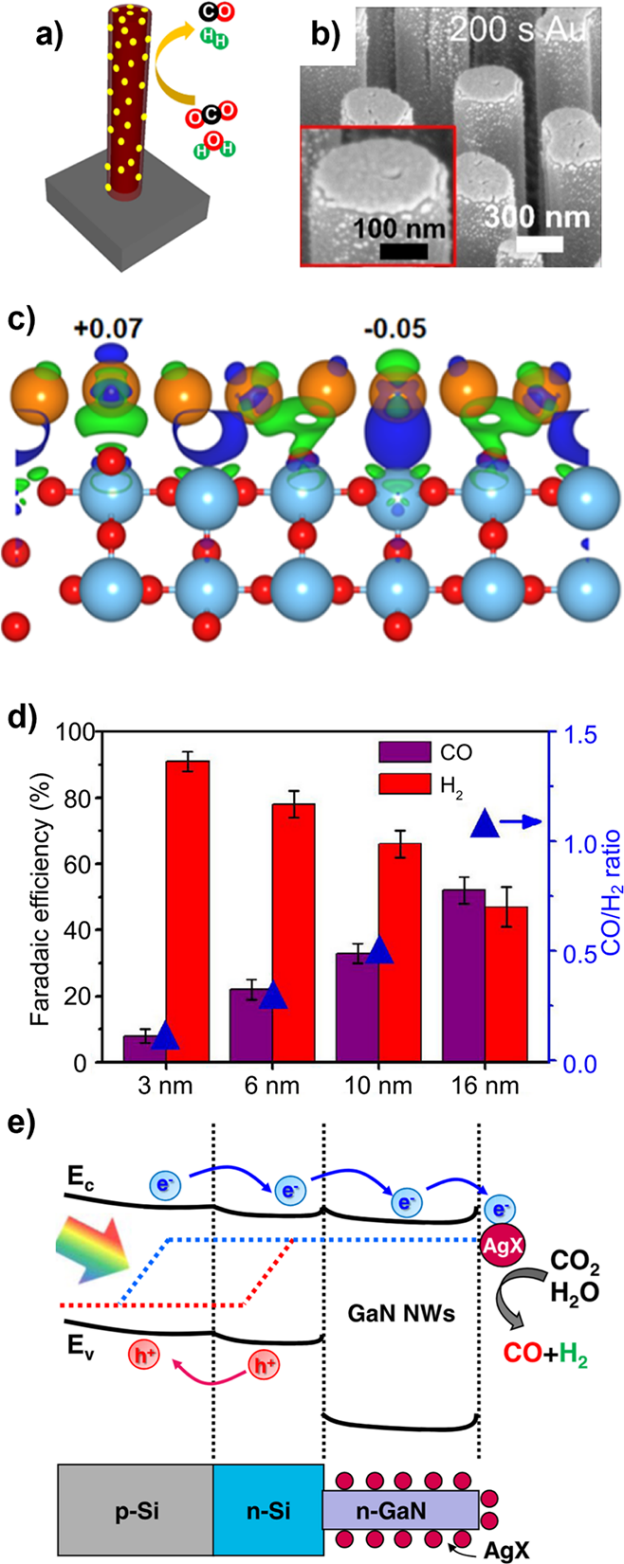
(a) Diagram of nano pillar array structure, (b) SEM of the InP nano pillar array after 200 s of electron beam evaporation Au deposition yielding a consistent thin film, (c) computationally predicted partial charge distributions of a Au(111) layer on InP. Blue represents positive partial charge which yields enhanced binding energies for CO with little effect on that of surface hydride binding, thus minimizing energy barriers for CO_2_RR relative to HER. (a–c) Adapted with permission from ref 551. Copyright 2021 American Chemical Society. d) Au nanoparticle size-dependent product control for CO_2_RR. Adapted with permission from ref 552. Copyright 2022 Elsevier. (e) Band diagram for the charge carrier flow of GaN nanowire photocathodes with AgX surface catalytic sites. Adapted with permission from ref 553. Copyright 2022 American Chemical Society.

Similar to nanowires, the construction of inverse opals from semiconductors is an effective way to improve photon interaction, carrier yields, and carrier mobility.^554,555^ The porous nature of the inverse opal structure also provides a high surface area and myriad exposed catalytic surface sites. Inverse opals have dramatically increased PEC CO_2_RR activity and photoresponsiveness in CuBi_2_O_4_ relative to planar material.^556^ The best performer possessed smaller void spaces (200 nm) in the inverse opal structure and reduced CO_2_ to CO at an applied potential of 0.6 V_RHE_ with an improved FE of 73% compared to the control material (49% FE for CO). A later study utilizing a similar CuBi_2_O_4_ photocathode achieved 92% FE for CO at an applied potential of 0.2 V_RHE_. They determined that destructive photocorrosion by a hole derived Cu–OH dependent pathway was kinetically dependent on electrolyte composition. They then constructed a composite material designed to limit destructive processes on the CuBi_2_O_4_ surface and enhance electron transfer kinetics to CO_2_. This system utilized a larger (800 nm) templating agent, yielding larger void spaces in the inverse opal structure, and decorated the surface with Ag nanoparticles.^557^

To achieve C_2+_ products in any CO_2_RR catalysis, a high local concentration of bound intermediate species is required.^545^ Unstabilized intermediates will lead to undersaturation of the surface and a spatial barrier to C–C bond forming steps between two such species. Stabilization and cooperative action by multimetallic surfaces is an effective strategy for precisely controlling the specific adsorption of reduced intermediates, and therefore promoting higher order hydrocarbon products, and has been reported several times for PEC CO_2_RR. One demonstration utilized mixed Cu–Ag thin films on a p-Si photoelectrode capped with SiO_2_.^558^ Grain boundary oxidation during photoelectrode preparation produced surfaces with high activity toward the HER, but microstructuring Ag-capped, large-grain Cu films, minimized grain boundary oxidation, and selective patterning of the metallic catalysts in the SiO_2_ retained the photocurrent in the p-Si. These photocathodes had high activity for CO_2_RR to CO (59.3% FE) at −1.0 V_RHE_ or CH_4_ (79.8% FE) at −1.4 V_RHE_.^558^ A similar photocathode was constructed of a Si p–n junction with a dendritic Cu-on-Ag catalyst, which produced mostly C_2_ products with ethylene and ethanol representing the highest FEs. An appreciable amount of C_3_ products were also detected in the mixture, along with the standard C_1_ products CO and formate.^559^

Certain metals and doped semiconductors have photoexcitable bulk motions in their electron clouds with favorable energetics for CO_2_RR. These surface plasmons can enhance the activity and selectivity of catalytic platforms in two ways.^548^ First, plasmonic excitation induces intense surface electric field effects that can be highly effective at influencing local bonds in bound CO_2_RR intermediates, providing a handle for the control of product selectivity.^560^ Second, plasmons provide a route for introducing high energy electrons to the catalytically active surface with the input of low energy visible light, either by the direct generation of hot electrons or recently, the extraction of hot holes. The traditional mechanism for plasmon-enhanced catalysis operates by hot electron extraction into a catalytic surface site for subsequent binding of CO_2_. However, the build-up of catalytically available electrons relies on the sluggish kinetics of hot electron migration through the junction into the catalyst (Figure 39a). Alternately, extraction of hot holes out of a plasmonic CO_2_RR material can provide a more efficient method for inducing build-up of available electrons at a catalytically active surface (Figure 39b).^561,562^ A major example of plasmon-enhanced CO_2_RR features a commercial p-GaN semiconductor decorated with Au nanoparticles, created by evaporating a thin layer of Au that was then annealed at 300 °C to form particles.^563^ The resulting photocathodes enhanced the CO evolution rate by 20% at an applied potential of −1.8 V_RHE_, with no observable change in the rate of HER (Figure 39c), demonstrating a strategy that improves photogenerated charge separation and delivery to reactant CO_2_ molecules. Follow-up work substituted p-GaN and Au with p-NiO and Cu; in this newer system, CO_2_RR by plasmonic excitation was also shown to diminish HER, thereby increasing product selectivity for CO and HCOOH (Figure 39d–f).^564^

**Figure 39:**
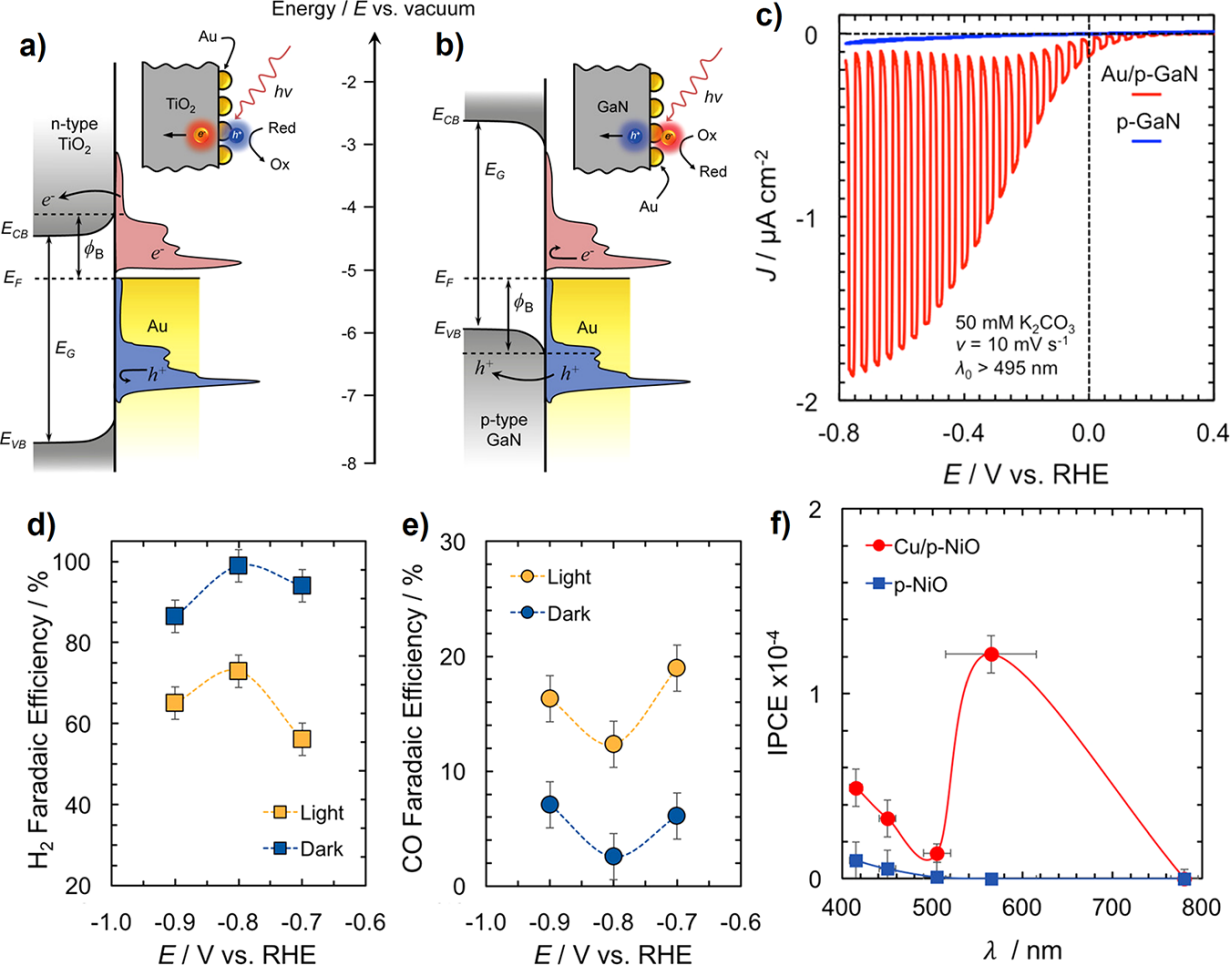
(a–c) Hot carrier collection from Au plasmonic nanoparticles on p-GaN for CO_2_RR. (a) Schematic depiction of hot electron collection across the junction between a plasmonic metal (Au) and n-type TiO_2_. The density of states (DOS) for hot electrons (red) above the conduction band Schottky barrier are small relative to the total DOS above the Fermi-level (*E*_F_). (b) Hot hole collection across the junction between a plasmonic metal (Au) and p-type GaN. Here, the DOS for hot holes (blue) below the valence band Schottky barrier outweigh the DOS for holes above the barrier that will remain on the plasmonic metal. (c) Photocurrent of p-GaN|Au composite (red) and native p-GaN (blue) under CO_2_RR conditions. (a–c) Adapted with permission from ref 563. Copyright 2018 American Chemical Society. (d–f) Hot carrier collection from Cu plasmonic nanoparticles on p-NiO for CO_2_RR, with FEs for both (d) H_2_ and (e) CO at 525 nm (yellow) or in the dark (blue), and (f) incident photon to current efficiency for p-NiO (blue) or p-NiO|Cu (red) as a function of wavelength indicating greater utilization of visible irradiation by p-NiO|Cu. (d–f) Adapted with permission from ref 564. Copyright 2020 American Chemical Society.

### 4.2 Dinitrogen Reduction (N_2_RR) and Nitrate Reduction (NO_3_^–^RR)

Ammonia (NH_3_) is a critical component for agricultural fertilizers, a building block for many commodity chemicals, and a promising energy-dense fuel. PEC N_2_RR may provide decentralized NH_3_ synthesis under benign conditions driven by solar energy, driving a surge in this research area over the past few years,^18^ but faces several challenges. First, photocathodes for PEC-N_2_RR must successfully bind and activate N_2_, which is challenging as N_2_ is both nonpolar and contains a strong N≡N triple bond. The standard reduction potential of the N_2_RR in acidic electrolyte is +0.275 V_SHE_ (Table 1). Coupling N_2_RR with the OER anodic reaction results in a minimum semiconductor band gap of 0.96 eV to drive unassisted PEC N_2_RR, but kinetic limitations of the multistep N_2_RR demand large overpotentials to drive the reaction at appreciable rates,^565^ so much larger band gaps are likely required for practical PEC N_2_RR. Second, although PEC-N_2_RR has been demonstrated in acidic, neutral and basic aqueous electrolytes at ambient temperature and pressure, N_2_ solubility is low in common solvents,^566^ and HER competes with N_2_RR in aqueous environments. As a result, the FE of most reported PEC-N_2_RR catalysts is low (i.e., <50%). Third, both PEC and electrochemical N_2_RR research in general are hindered by contaminating NH_3_ as well as other contaminating N species that can be reduced to NH_3_, such as NO_3_^–^. Thus, numerous controls for contamination and exhaustive control experiments must be carried out to avoid false-positive results. Although NO_3_^–^ can be an undesirable contaminant in N_2_RR, PEC NO_3_^–^RR represents an additional route to solar NH_3_ generation. NO_3_^–^ occurs abundantly in several wastewater sources, and conversion of NO_3_^–^ to NH_3_ achieves a circular waste-to-fertilizer (or fuel) nitrogen cycle.^567^ The solubility of NO_3_^–^ in aqueous solution is much greater than that of N_2_, and the bond dissociation energy of N=O (204 kJ mol^–1^) is less than a quarter of the bond dissociation energy of N≡N (941 kJ mol^–1^),^568^ potentially expanding the range of semiconductors that can be employed as photocathodes compared to N_2_RR (see Table 1).

Best practices (including recommended control experiments) for electrochemical N_2_RR are well documented in several recent reviews;^569,565,570,571^ these standards should be applied to both to PEC N_2_RR and NO_3_^–^RR. Reliable experiments should include purification of electrolyte and the N_2_ reactant gas to remove possible contaminants, and additional sources of contaminants, such as membranes, should be cleaned. Additionally, electrosynthesis control experiments should be utilized, including tests with a working electrode which is not active toward N_2_RR such as glassy carbon, and tests of the catalyst in Ar-purged electrolyte. Multiple detection methods for NH_3_ should be used, such as both the indophenol UV–vis assay and quantitative nuclear magnetic resonance (NMR).^572,573^ Finally, a catalyst should be tested with isotope-labeled ^15^N_2_, the ^15^N_2_ source should be purified of N contaminants,^574^ and the results of the ^15^N_2_ and ^14^N_2_ reduction experiments should be quantitatively similar. Including these control experiments in PEC-N_2_RR studies is critical to ensuring the accuracy of stated results.

Photocathode design strategies for N_2_RR focus on controlling light harvesting, providing active sites for N_2_ binding and activation, and ensuring catalyst durability. Semiconductors act as both photoabsorbers and catalysts, and in some cases plasmon absorbers are combined with semiconductors to promote photoabsorption and provide catalytic sites as in CO_2_RR. A diverse range of semiconductors and semiconductor junctions have been applied to PEC N_2_RR, and advances in PEC-N_2_RR catalyst development have frequently included generation of semiconductors rich in defects or dopants serving as especially active sites for N_2_ binding and activation, synthesis of high-surface area nano- and microstructured semiconductors and tuning of semiconductor junctions for efficient electron/hole separation.

Transition metal oxides are popular for N_2_RR photocathodes owing to the typically good stability of oxides in aqueous environments and the high catalytic activity of oxygen vacancy sites, which can bind and activate N_2_. Rutile TiO_2_ nanorods decorated with vacancy-rich anatase TiO_2_ and Ag nanoparticles have been used as a photocathode (Figure 40),^575^ with the proposed mechanism of photogenerated electron transfer from both the rutile TiO_2_ nanorods and the Ag nanoparticles to the vacancy-rich anatase TiO_2_, which contained the active site for N_2_ reduction. The composite TiO_2_ and Ag nanoparticle photocathode showed good stability, yielding NH_3_ in similar amounts over five consecutive 150 min trials, with consistent activity in the range of 10s of μg·h^–1^·cm^–2^.^575^ Hollow microspheres of Mo-doped WO_3_ capped with CdS and dropcast on carbon paper contact layers have also been used as a photocathode, with moderate-to-high catalytic activity toward N_2_RR (38.99 μg·h^–1^·mg_cat_^–1^) attributed to the presence of oxygen vacancies in the WO_3_.^576^ The Mo dopant in the Mo-doped WO_3_ was also credited as playing a critical role in the catalytic activity of the material, and DFT simulations showed that a Mo–W dimer site which is proximal to an oxygen vacancy is energetically favorable for N_2_ binding and activation.

**Figure 40:**
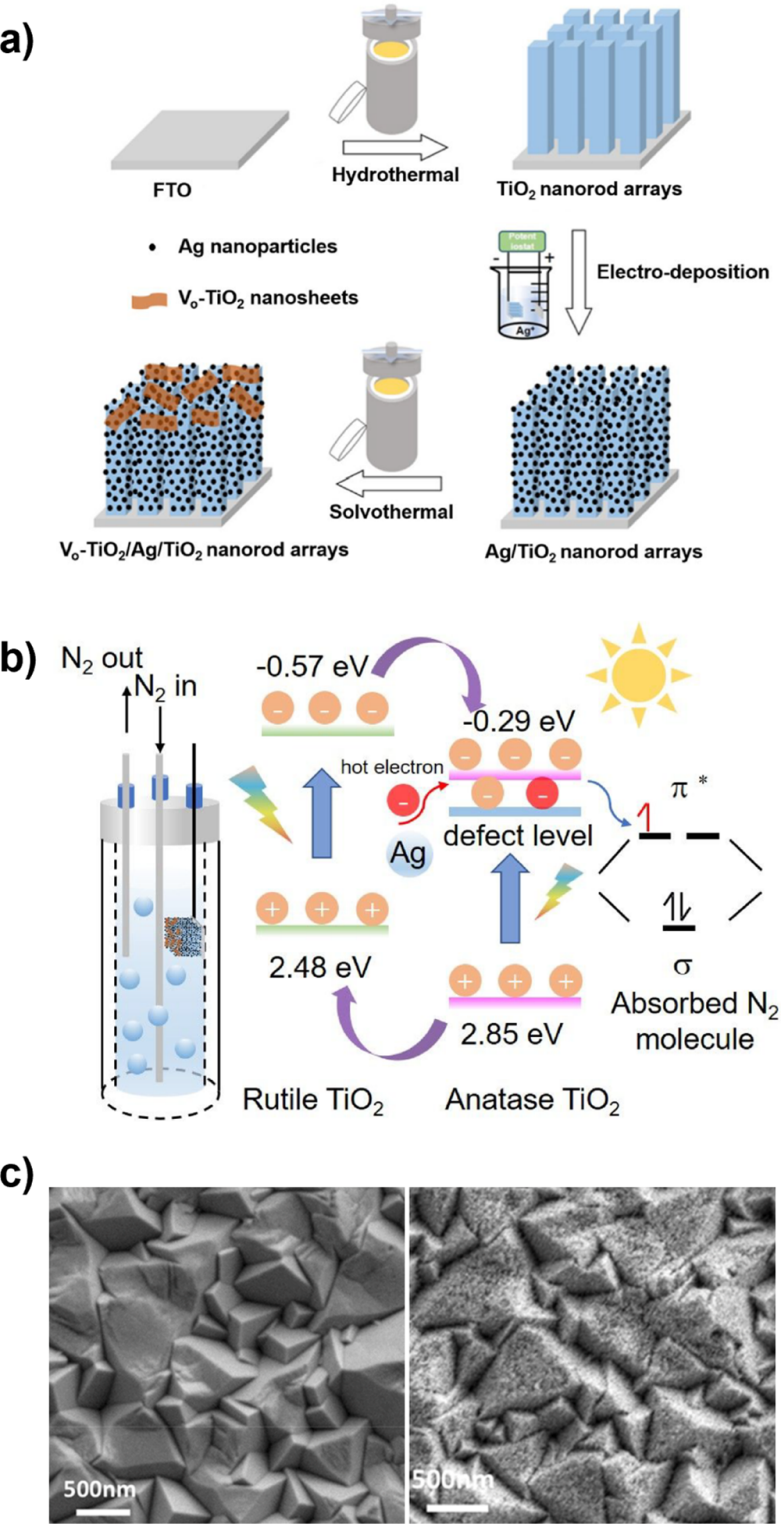
(a) Schematic illustration of photocathode synthesis showing, hydrothermal growth of rutile TiO_2_ nanorods, electrodeposition of Ag nanoparticles, and solvothermal growth of vacancy-rich anatase TiO_2_ sheets (TiO_2_|Ag|*v*_O_^••^-TiO_2_). (b) Proposed PEC-N_2_RR mechanism on TiO_2_|Ag|*v*_O_^••^-TiO_2_. (a,b) Adapted with permission from ref 575. Copyright 2023 American Chemical Society. (c) SEM images of Cu_2_O photocathode showing pitting photocorrosion of the photocathode before (left) and after (right) a 60 min PEC-N_2_RR chronoamperometry test with the following parameters: *E* = 0.4 V_RHE_, 0.1 M KOH electrolyte, 1 sun illumination. Adapted with permission from ref 214. Copyright 2020 American Chemical Society.

There are several reports of N_2_RR on Cu oxide photocathodes, but as discussed in section 2.1.5.1, these semiconductors face stability challenges. N_2_RR has been demonstrated directly on both CuO and Cu_2_O, with FEs toward N_2_RR of 17% and 20%, respectively, but photocorrosion side reactions accounted for the majority of the photocurrent for both semiconductors over a 1 h experiment.^214^ Interestingly, for both CuO and Cu_2_O the potentials required to reduce N_2_ were more positive than the thermodynamic reduction potential of N_2_, which provides strong evidence for the participation of photogenerated carriers and a PEC rather than EC mechanism. Another study similarly demonstrated PEC-N_2_RR activity on Cu_2_O and examined a Cu_2_O photocathode decorated with a Cu-based metal organic framework (MOF).^577^ The Cu_2_O|Cu-MOF photocathode showed higher N_2_RR activity than Cu_2_O alone, which was attributed to the presence of unsaturated Cu sites, and based on the finding of photocorrosion on Cu_2_O, it is possible that the Cu-MOF coating helped to prevent photocorrosion via catalytic protection. A similar phenomenon was observed on a microstructured Cu_2_O photocathode coated with Ni nanoparticles, where the Ni metal coating served to protect the Cu_2_O from degradation by acting as a physical barrier as well as providing catalytic protection, that is, by making the reaction of interest N_2_RR more facile than competing corrosive side reactions.^578^

Enabling additional light absorption, several types of plasmon absorbers, most commonly noble metals, have been coupled with semiconductors to make N_2_RR photocathodes. The active site for N_2_ binding and activation in this case is typically the plasmonic nanoparticle, which is in physical contact with the semiconductor; the semiconductor serves as the primary or auxiliary light absorber and path for current to the counter electrode. Plasmonic materials under illumination generate “hot” carriers, and in one case hot carrier generation on illuminated Au nanoparticles (with no semiconductor light absorber present) displayed selectivity toward N_2_RR over HER in comparison to nonilluminated electrochemistry.^579^ Plasmonic Ag has also been demonstrated to catalyze PEC-N_2_RR, such as in the case of a black (etched, high surface area) Si photocathode decorated with Ag nanoparticles.^580^ The high catalytic activity of the photocathode (2.87 μmol·h^–1^·cm^–2^) was attributed to the efficient light absorption and photoexcited electron transfer by the black Si, while the Ag nanoparticles provided active sites for N_2_ binding and activation. AuCoPd alloy nanoparticles have also been deposited on a Si photoelectrode protected with SiO_2_ and CoO_*x*_; in this case, photogenerated electrons from the Si layer transferred to the AuCoPd nanoparticles, and N_2_ binding and activation occurred at a bimetallic AuPd site.^581^ However, it was unclear from this report if the AuCoPd nanoparticles displayed a plasmon. The use of hot carriers from plasmon absorbers may provide a route to use light wavelength to tune N_2_RR selectivity, given that such absorbers have displayed wavelength-dependent N_2_RR *activity*.^582^ For example, carriers of different energy generated by varying wavelengths may show different selectivity toward N_2_RR versus HER.

As an alternative to combating the selectivity challenge of N_2_RR and semiconductor photocorrosion in aqueous environments, the Li-mediated N_2_RR pathway can also be driven photoelectrochemically. In this pathway, a chemical reaction occurs between metallic Li^0^ and N_2_ in organic electrolyte, forming Li_3_N, followed by protonation of Li_3_N by a proton donor (such as EtOH) to form NH_3_ and Li^+^, and finally, Li^+^ is electrochemically reduced back to Li^0^ to complete the cycle.^583^ The Li-mediated N_2_RR has been demonstrated on a p-Si photocathode in THF under 300 W irradiation from an Xe lamp, with the photogenerated electrons from Si driving the reductive regeneration of Li^+^ to Li^0^.^584^ The overall FE of this system toward N_2_RR was 95%. Investigation of the mechanism using in situ XRD showed that Li_3_N was present on the photocathode when the proton donor ethanol was absent, but not when it was present, providing evidence that Li_3_N was formed and that in the presence of ethanol it reacts to form NH_3_. The formation of a solid electrolyte interphase (SEI) layer composed partially of LiF (a product of the LiBF_4_ electrolyte reduction) was also observed by in situ XRD, which possibly played a role in facilitating Li^+^ ion transport to the photocathode surface.^584^ Li-mediated PEC N_2_RR was similarly demonstrated on a heterostructured Si|TiO_2_|PdCu photocathode; in that case, the composite photocathode with the PdCu catalyst present showed increased NH_3_ yield compared to the Si|TiO_2_ photocathode alone. This was attributed to the cocatalyst layer facilitating the adsorption and reduction of Li^+^ as well as facilitating the formation of a SEI layer.^585^

The reduction of NO_3_^–^ to NH_3_ is an additional route for solar NH_3_ generation, and several approaches to photocathodes have been employed to catalyze PEC NO_3_^–^RR. Two recent examples couple vacancy-rich semiconductors with p-type BiVO_4_, with the intent of using the vacancy sites as frustrated Lewis pairs (where the Lewis acid and base are in close proximity but sterically hindered from adduct formation) as the sites for binding and activation of NO_3_^–^.^586,587^ In the proposed mechanism, the frustrated Lewis pair generates a site between the Lewis acid and base which can activate small molecules by simultaneously accepting and donating electron density. One demonstration of this principle used carbon-doped, oxygen vacancy-rich CeO_2_, with an undercoordinated cerium atom and a neighboring oxygen atom playing the role of the Lewis acid and base (Figure 41).^586^ Evidence for frustrated Lewis pairs being the active site for NO_3_^–^ binding and activation was provided by DFT simulation of an oxygen-vacancy site in CeO_2_, which showed that electron density increases with proximity to the oxygen vacancy, suggesting that the oxygen vacancy site was conductive to nucleophilic attack by NO_3_^–^_._ The carbon-doped, oxygen vacancy-rich CeO_2_ showed a moderate-to-good NO_3_^–^RR activity maximum of 21.81 μg·h^–1^·cm^–2^.

**Figure 41:**
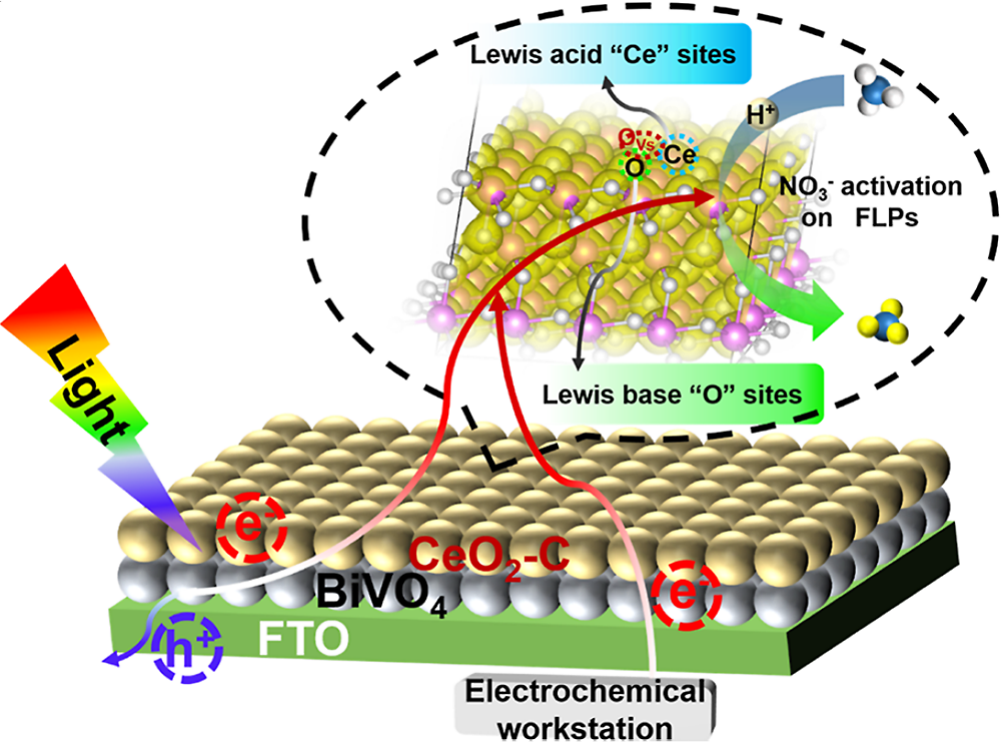
Schematic illustration of PEC-NO_3_^–^RR on a BiVO_4_|CeO_2_–C composite photocathode showing the proposed mechanism of NO_3_^–^ activation at a frustrated Lewis pair site consisting of an oxygen vacancy in CeO_2_. Adapted with permission from ref 586. Copyright 2023 American Chemical Society.

PEC NO_3_^–^RR has also been demonstrated using plasmonic catalysts integrated with semiconductors, including a photocathode composed of Si nanowires decorated with Au nanoparticles which showed high selectivity (95.6% at 0.2 V_RHE_) toward NO_3_^–^RR.^588^ In this case, limitation of the competing HER was cited as being crucial to the performance of the catalyst and this motivated the selection of Si and Au, which are both poor HER catalysts. Importantly, the Au nanoparticles were shown to dissolve on the time scale of hours in 0.5 M K_2_SO_4_ with 10 mM KNO_3_, resulting in loss of activity. This problem could be temporarily fixed by redepositing Au nanoparticles but represents a challenge to the use of Au nanoparticle plasmons for long-term PEC NO_3_^–^RR.

### 4.3 Alternative Oxidation Reactions

There has recently been increasing focus on alternatives to OER as the oxidative half-reaction in PEC fuel-forming systems, motivated not only by providing the electrons and protons for reduction half-reactions at a lower anodic overpotentials, but by identifying new means of producing valuable chemical products. Replacing OER with appropriate alternative oxidation reactions would decrease the energy requirement for a full PEC cell by either lowering the kinetic overpotential or decreasing the net thermodynamic barrier on the oxidation side. Lowering the thermodynamic requirements for driving a full PEC fuel-forming reaction allows for the use of smaller band gap semiconductors that cannot be used for overall water splitting. In principle, a more facile oxidation reaction could also raise operational current density by these means, leading to higher η_STF_ for a full system (Figure 42). On the other hand, the reactants for alternative oxidative half-reactions significantly change the parameters of the PEC working environment, e.g., the pH and corrosiveness of the electrolyte. This leads to very different stability questions for photoanodes, which might deteriorate despite high performance of the system compared to coupling with OER. With some alternative oxidation reaction reviews already in the literature,^19–21^ this section scrutinizes the recent developments in photoanodes for PEC alternative oxidation reactions.

**Figure 42:**
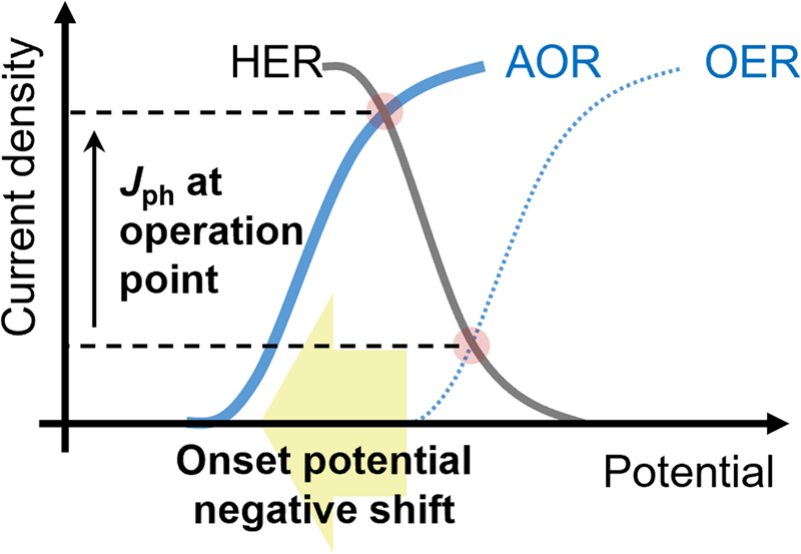
Replacing OER with a thermodynamically or kinetically favorable alternative oxidation reaction (AOR) can increase the operational current density of tandem device.

#### 4.3.1 Glycerol Oxidation Reactions (GOR)

Alcohols are good targets for alternative oxidation reactions because thermodynamically, they can be converted to aldehydes, ketones, or carboxylic acids at lower applied oxidative potentials than that required to drive OER. Of the possible alcohols for oxidation reactions, glycerol (C_3_H_5_(OH)_3_) is commonly used in industries from soap and cosmetics to demolitions and medicine,^589^ and the global availability of crude glycerol has been increasing (∼15% growth per year) since 2000.^589^ With a global excess of glycerol,^590^ research has turned to methods to transform glycerol to more valuable commodity chemicals or as a substitute for water coupled with HER.^591^ The partial oxidation of glycerol produces various value-added oxidation products, such as 1,3-dihydroxyacetone (DHA), glyceraldehyde (GALD), glyceric acid, and lactic acid. The complete oxidation of one mole of glycerol generates proportionally the largest amount of H_2_ (7 mol of H_2_) with an energy input of only 3.9 kJ mol^–1^,^523^ compared to the 1:1 ratio of H_2_ and O_2_ production from water splitting. However, all glycerol oxidation reactions (GOR), and alcohol oxidations in general, face several challenges. Catalyst durability is one issue, as prolonged anodization forms oxide layers that can partially deactivate or completely terminate the targeted reaction.^592^ These issues extend to the PEC oxidation of alcohols, where an excess of holes at the photoanode surface can lead to unfavorable oxidation of the photoanode semiconductor or catalyst. Some oxide layers can continue to perform alcohol oxidation at higher potentials, but the anodic process can become a mixture of alcohol oxidation and water oxidation with lower η_F_. Selectivity is another issue, particularly for GOR, where the operating potential and catalyst exert a strong influence on what product is formed.^593^ The more valuable C_3_ products from glycerol oxidation are produced at smaller applied potentials, where the C–C bonds of the glycerol backbone are unlikely to break.

While this means that simply increasing photovoltages and photocurrents does not necessarily produce more of the desired products as it does in water splitting, it also opens the door to the use of smaller band gap semiconductors to drive GOR coupled with HER (Figure 43). One recent example of using smaller band gap semiconductors for this purpose was the demonstration of simultaneous GOR and HER driven by two Si-based photoelectrodes without an applied bias.^594^ The photoanode was np^+^-Si coated with TiO_2_ as a protective layer and subsequently sputter coated with 4 nm AuPt. Pt functioned as the primary electrocatalyst for glycerol oxidation, while the addition of Au helped prevent CO poisoning; the onset of glycerol oxidation on this mixed metal system is nearly 1 V less oxidative than the onset of most OER electrocatalysts.^594^ The photocathode was an array of Si nanowires with a p–n^+^ junction protected with TiO_2_ with Pt as a HER catalyst. In a 1 M KOH electrolyte with 1 M glycerol added, the photoanode passed 10 mA cm^–2^ at 0.5 V_RHE_. The glycerol was mostly oxidized to glyceric acid (∼48% FE) and lactic acid (∼27% FE), with the rest of the products most likely being tartronic acid and carbonate. In a two-electrode, short-circuit configuration with the photocathode in a separate, acidic electrolyte chamber, the coupled photoanode and photocathode operated at 4 mA cm^–2^ for 4 days of diurnal cycling (12 h illumination followed by 12 h in the dark). The PEC activity dropped from day 4 to day 6, although separate experiments showed that refreshing the electrolyte restored PEC activity of the photoanode, suggesting that depletion of the electrolyte rather damage to the photoelectrode was the controlling factor in performance loss.^594^

**Figure 43:**
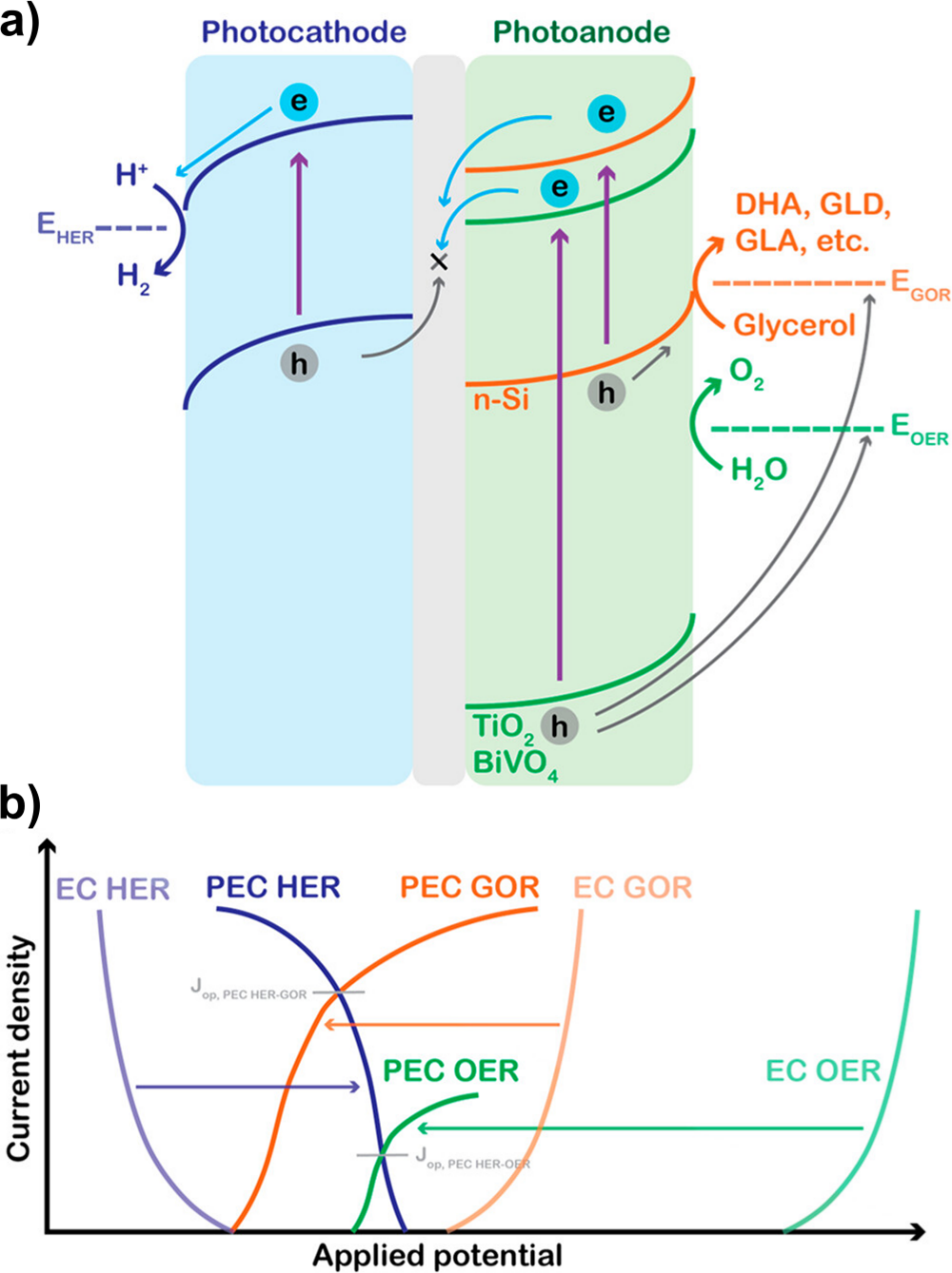
Illustration of benefits of PEC GOR as an alternative to OER. (a) Energy band diagrams of OER and GOR, where electrons drive HER in the photocathode and holes in the photoanode perform GOR (Si) or OER (BiVO_4_|TiO_2_). Note that wide band gap semiconductors can also drive GOR but selectivity compared to OER may be a challenge. (b) Schematic *J*–*V* curves of HER, GOR, and OER on separate (photo)­electrodes. Intersection of current density–voltage curves indicate predicted operating points for unbiased operation of PEC oxidation and reduction reactions. (a,b) Adapted with permission from ref 594. Copyright 2023 American Chemical Society.

BiVO_4_ has also emerged as a promising photoanode for GOR, due to its band gap of 2.4 eV having enough overpotential to drive GOR and its poor native OER kinetics. Several studies have explored electrochemical conditions for GOR and revealed that BiVO_4_ can selectively oxidize glycerol to DHA. In one example, porous BiVO_4_ nanoflake arrays, generated by reacting vanadyl acetylacetonate with BiOI on an FTO substrate and subsequently annealing, were tested for GOR performance in pH = 2, 5, 7, and 12 electrolytes with 0.1 M glycerol.^595^ Both the photocurrent of the BiVO_4_ photoanode and the GOR selectivity toward desired products were highest at pH 2, with 50% selectivity and 30% FE toward DHA, although at pH 12 the reaction had 99% FE for formic acid. DFT calculations suggested that this acidic electrolyte enabled a more stable radical formation on the C_2_ carbon of glycerol, which is then oxidized to give DHA. The nanoflake array was stable for 5 h at 1.2 V_RHE_ in pH 2 electrolyte with a current density of ∼4.5 mA cm^–2^.^595^ In another example, W-doped BiVO_4_ from an electrodeposition-calcination method was coupled with an ALD-deposited NiO_*x*_(OH)_*y*_ catalyst in pH 7 (0.5 M Na_2_SO_4_) and pH 9.3 (0.5 M potassium borate buffer) electrolytes with 0.1 M glycerol.^596^ Larger applied biases (1.2 V_RHE_) yielded improved DHA selectivity in both electrolytes, with better selectivity toward DHA in the buffered pH 9.3 electrolyte. The 0.5 M Na_2_SO_4_ electrolyte which acidified over the course of PEC GOR operation and afforded a range of products.^596^

BiVO_4_ photoanodes used for GOR still suffer from low overall light capture and charge transfer to electrolyte, as well as poor stability in acid. Although BiVO_4_ photoanodes derived from BiOI are often porous, annealing BiVO_4_ at 400 °C in air and in the presence of additional V_2_O_5_ (to prevent the formation of V-related defects) improved the structure of the BiVO_4_, enhancing light absorption, quantum efficiency and photocurrent toward GOR.^597^ However, the relative selectivity toward DHA compared to other GOR products in the 0.1 M glycerol, 0.5 M H_2_SO_4_, and 0.5 M Na_2_SO_4_ electrolyte was roughly the same compared to the standard porous BiVO_4_ control.^597^ Another study focused on improving the stability of BiVO_4_ in acid via Ta doping.^598^ Ta-BiVO_4_ photoanodes were stable under PEC operation at 1.0 V_RHE_ in 25 mM H_2_SO_4_ for 120 min, compared to undoped BiVO_4_ photoanodes which lost ∼25% photocurrent during that time. The Ta-BiVO_4_ photoanodes also had over 80% selectivity for glycerol to DHA in 100 mM H_2_SO_4_.^598^

GOR is an important valorization strategy for the excessive quantity of crude glycerol being produced worldwide today. Combining it in PEC fuel-forming systems provides a strategy to lower overall cell potential, bypass the potential required for water oxidation, and allow for valuable product generation at both the anode and cathode. Product selectivity remains the primary challenge in scaling glycerol oxidation systems, as at higher current densities (and potentials) it is difficult to prevent co-oxidation of glycerol and water. One recent study aimed to elucidate the role that illumination plays in reducing competition between GOR and OER, using rutile TiO_2_ and monoclinic BiVO_4_ as model photoelectrodes.^599^ Although illumination widens the gap between GOR and OER onset potentials, making GOR more favorable, it was not clear if these reaction rates and therefore selectivity were modified beyond a simple reduction of η_Ο_. Like CO_2_RR, potential-dependent selectivity is a challenge for GOR, one which may benefit from investigation of wavelength-dependent selectivity. Future work in this space should continue improving the selectivity for certain GOR products and maximizing the efficiency of small *E*_g_ semiconductors that cannot effectively be utilized in water splitting due to the larger thermodynamic requirement for that reaction.

#### 4.3.2 Halide Oxidation Reactions

Halide oxidations are promising alternative oxidation reactions for PEC, as two-electron oxidations (Table 1) with no coupled proton transfer that are kinetically simpler than the four-electron OER.^19^ The dissolution of a strong acid in water as an electrolyte provides reactants for the straightforward oxidation of halides even at very high concentration, thus halide oxidations represent a notable simplification of the system when coupled with HER compared to OER. However, halide oxidation reactions in strong acids impose stringent requirements on the stability of photoanodes, because most reported photoanodes studied for OER are thermodynamically unstable in strong acidic media.^600^ Diatomic chlorine is crucial for synthesizing polymers and producing disinfectants. Although the thermodynamics of the chlorine evolution reaction (CER) are unfavorable (*E* ^0^(Cl_2_/Cl^–^) = 1.36 V_SHE_) relative to the OER (*E* ^0^(O_2_/H_2_O) = 1.23 V_SHE_), the two-electron transfer kinetics of CER are more facile than the four proton-concerted electron transfer steps of OER, typically exhibiting lower overpotentials.^601^ The iodide oxidation reaction (IOR) is another prominent AOR candidate due to a low half-cell potential of 0.54 V_SHE_. Oxidation of iodide occurs via a similar two-electron transfer pathway, forming triiodide ions (I_3_^–^) as valuable sterilization agents.^602^ Due to the benefits of both thermodynamics and kinetics, the minimum ∼0.55 V needed to oxidize iodide under standard conditions can be provided by a single semiconductor with quite a small band gap relative to other overall PEC fuel-forming reactions, substantially widening the number of semiconductors which can be considered as a photoelectrode for IOR, including Si without coupling to a wide band gap semiconductor.^603^

Diatomic chlorine is the dominant product of CER below pH 3.0, while HOCl is produced between pH 3.0 and 7.5, and hypochlorite is produced above pH 7.5 (Figure 44a).^525^ As described in section 2.1.2, BiVO_4_ is widely used as a photoanode in solar water oxidation because of its advantageous band gap and the conduction band edge position. While BiVO_4_ remains stable in neutral or light basic pH conditions, the electrodeposition of an amorphous WO_3_ layer can protect BiVO_4_ from corrosion in an acidic chloride electrolyte (pH 1) and simultaneously promote efficient hole transport for the chloride oxidation.^604^ Under illumination, photogenerated holes in BiVO_4_ were delivered to the surface through the amorphous WO_3_ layer oxidizing chloride ions to chlorine and the counter electron simultaneously produced hydrogen at the cathode side (Figure 44b). Upon the durability test, a bare BiVO_4_ photoanode exhibited larger initial photocurrents in comparison with its WO_3_-coated counterpart, but the current significantly dropped within 20 min due to rapid photocorrosion. The BiVO_4_|WO_3_ photoanode maintained more than 95% of the initial photocurrent even after 3 h of operation under light (simulated AM1.5G). After 2 h of constant potential (1.42 V_RHE_) experiments, a high photocurrent-to-Cl_2_ FE of 85% was observed while near unity FE was observed for H_2_ production on the counter electrode (Figure 44c).^604^ In another study, optimized loading CoO_*x*_ onto the BiVO_4_|WO_3_ photoanode promoted HClO production and improved the HClO selectivity to almost 100% in pH 5.9 electrolyte (Figure 44d).^605^ The CoO_*x*_ catalyst loading on photoanodes assisted efficient charge separation at the semiconductor|electrolyte interface while the specific nature the Co–Cl interaction on the surface of CoO_*x*_ could also improve the selectivity of chloride oxidation. In a third example, Co atoms were doped into the BiVO_4_ lattice which decreased the charge transfer resistance and enhanced the CER kinetics by providing reaction sites for CER.^606^ A photocurrent-to-Cl_2_ FE of 92% was observed for the best active 0.05 mol % Co-doped BiVO_4_, corresponding to 190 μA cm^–2^ at 1.1 V_RHE_.

**Figure 44:**
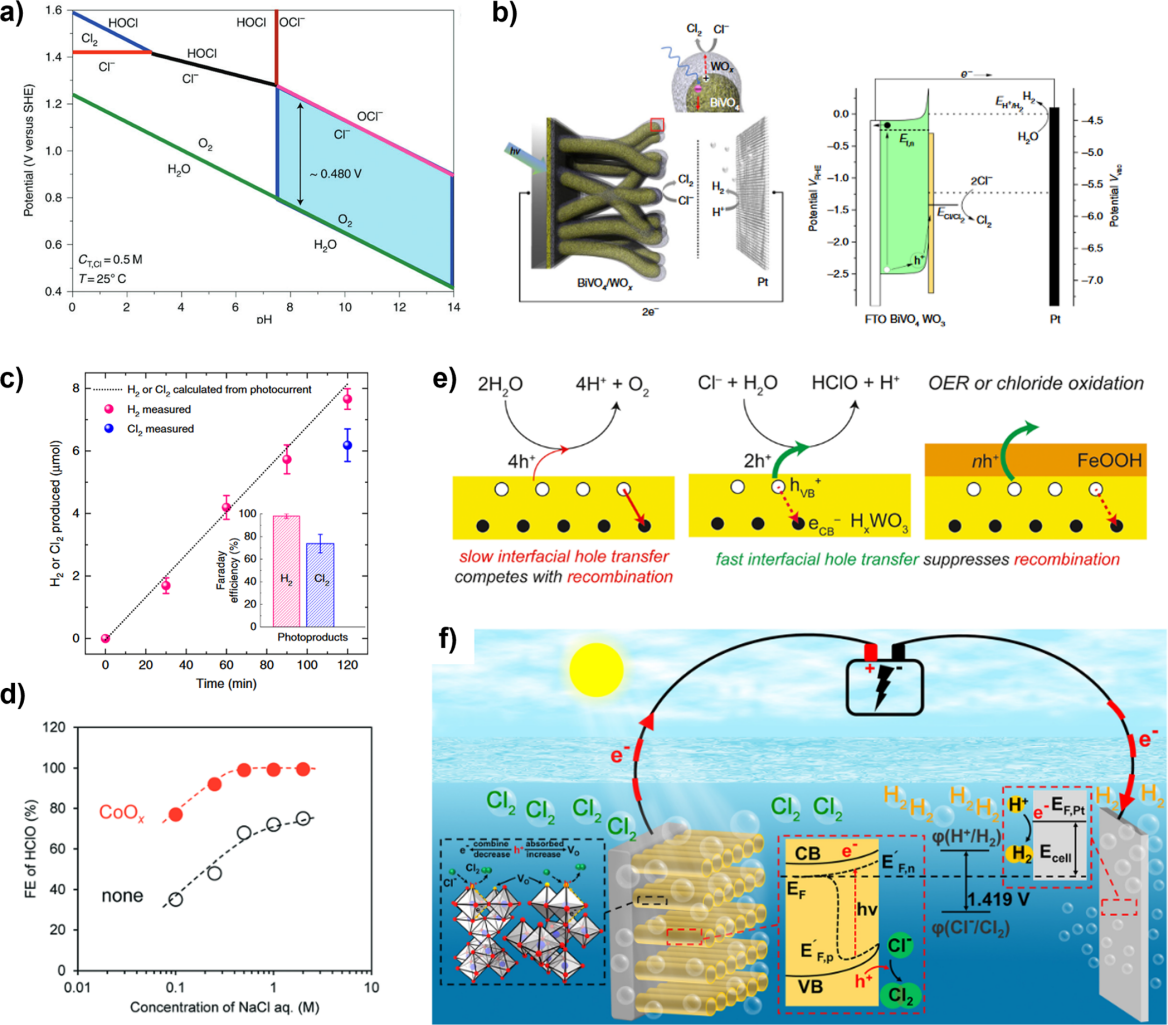
(a) Pourbaix diagram showing the thermodynamic potential-pH space where the CER and OER can occur. Adapted with permission from ref 525. Copyright 2016 Wiley-VCH. (b) Schematic diagram of the overall reaction and charge transfer process at the WO_3_|BiVO_4_ photoanode. (c) Total production of Cl_2_ and H_2_ as a function of duration under 1 sun illumination and corresponding Faradaic efficiency (inset). (b,c) Adapted with permission from ref 604. Copyright 2019 Nature Portfolio. (d) Effect of NaCl concentration on the PEC HClO production on WO_3_|BiVO_4_ photoanodes with and without CoO_*x*_. Adapted with permission from ref 605. Copyright 2021 Royal Society of Chemistry. (e) Interfacial hole transfer mechanism on various H_*x*_WO_3_ photoanodes. Adapted with permission from ref 607. Copyright 2021 American Chemical Society. (f) Scheme of the two-electrode cell for PEC production of H_2_ and Cl_2_. Adapted with permission from ref 608. Copyright 2023 Elsevier.

Another semiconductor targeted for photooxidation of Cl^–^ ions in acidic media is n-type WO_3_. In one study, it was experimentally demonstrated that chloride oxidation dominated compared to OER on H_*x*_WO_3_ photoanodes.^607^ The kinetics of chloride oxidation and OER were compared using NaNO_3_ and NaCl electrolytes. When interfacial hole transfer is sluggish, similar to the case of OER on H_*x*_WO_3_ with a redox-innocent NaNO_3_ electrolyte, photogenerated holes easily accumulate at the surface of H_*x*_WO_3_, followed by recombination with conduction-band electrons (Figure 44e), resulting in low FE of OER (87 ± 2%). Use of NaCl electrolyte or application of a FeOOH cocatalyst increases *j*_SC_, indicating rampant surface hole recombination under OER conditions. The H_*x*_WO_3_ photoanode exhibited 100% FE for chloride oxidation and much greater stability when oxidizing the chloride anion compared to water.^607^ Alternatively, TiO_2_ (in the form of nanotubes) is a great candidate to be a photoanode for PEC chlorine production due to its chemical stability.^608^ The introduction of oxygen vacancies in TiO_2_ allowed effective utilization of photogenerated holes and enhanced the PEC performance for chlorine production; the highest chlorine production rate was 37.12 μmol h^–1^ cm^–2^ with a FE of 73.2%. Contrasting the commonly cited 1.5–1.8 V whole-cell potential required for OER, a two-electrode cell with whole-cell potential <1.42 V is capable of performing unassisted hydrogen and chlorine evolution (Figure 44f).^608^

The IOR requires an even smaller potential than CER, which means that tandem photoelectrode configurations can drive coupled HER and IOR, possibly with the photocurrent density of PEC tandem devices reaching ∼10 mA cm^–2^ without external bias.^27^ Such configurations have recently been demonstrated using Sb_2_S_3_ as the photoanode, as Sb_2_S_3_ has relatively high stability in acidic electrolytes. In one example, a compact Sb_2_S_3_ layer was fabricated by spin coating from a molecular ink followed by an anneal, with subsequent layer-by-layer deposition of RuO_2_ nanosheets and polydiallyldimethylammonium chloride (PDDA) as a multilayered catalyst (Figure 45a).^609^ This photoanode coupled with a Si photocathode (Figure 45b), enabled the overall HI splitting reaction (Table 1). The operation point, determined by the crossing point of two linear sweep voltammetry (LSV) curves of Si photocathode and Sb_2_S_3_ photoanode, was ∼4.0 mA cm^–2^ at 0.25 V_SHE_ (Figure 45c). The actual bias-free photocurrent measured from the combined two-electrode tandem device under 1 sun illumination was also 4.0 mA cm^–2^, indicating the possibility of a practical solar-to-hydrogen conversion without applied bias. The Sb_2_S_3_ acted as a filter for the Si, absorbing wavelengths up to 775 nm (Figure 45d), and the calculated photocurrent density from integrating the IPCE spectra well-matched to ∼4 mA cm^–2^. XANES and extended X-ray absorption fine structure (EXAFS) analysis elucidated the roles of the PDDA|RuO_2_ catalyst, which was active toward iodide oxidation and passivated Sb_2_S_3_ surface states. In another example by the same group, a vertically oriented Sb_2_S_3_ photoanode was passivated with an amorphous WS_*x*_ layer and connected with a perovskite-based photocathode to demonstrate a bias-free parallel-configured tandem device for solar-hydrogen production (Figure 45e).^610^ The charge carrier lifetime of the Sb_2_S_3_ was enhanced after the passivation, suggesting the WS_*x*_ alleviated recombination at the photoelectrode|electrolyte interface and enabled facile charge transfer into the electrolyte.^610^ The current–potential profile overlap of photocathode and photoanode showed the operation current of 11.05 mA cm^–2^ (Figure 45f). However, the monitored photocurrent density under bias-free conditions was almost half, at 5.7 mA cm^–2^ because the active area was twice as large under the parallel illumination. Bias-free, coupled solar-driven HER and IOR was achieved for 2 h, maintaining 92% of the initial photocurrent density (Figure 45g). Replacing OER at the anode with halide oxidation can assist the overall solar fuel formation efficiency of the PEC system,^610^ although the nature of the electrolyte should be carefully considered to achieve reasonable stability of the photoelectrodes.

**Figure 45:**
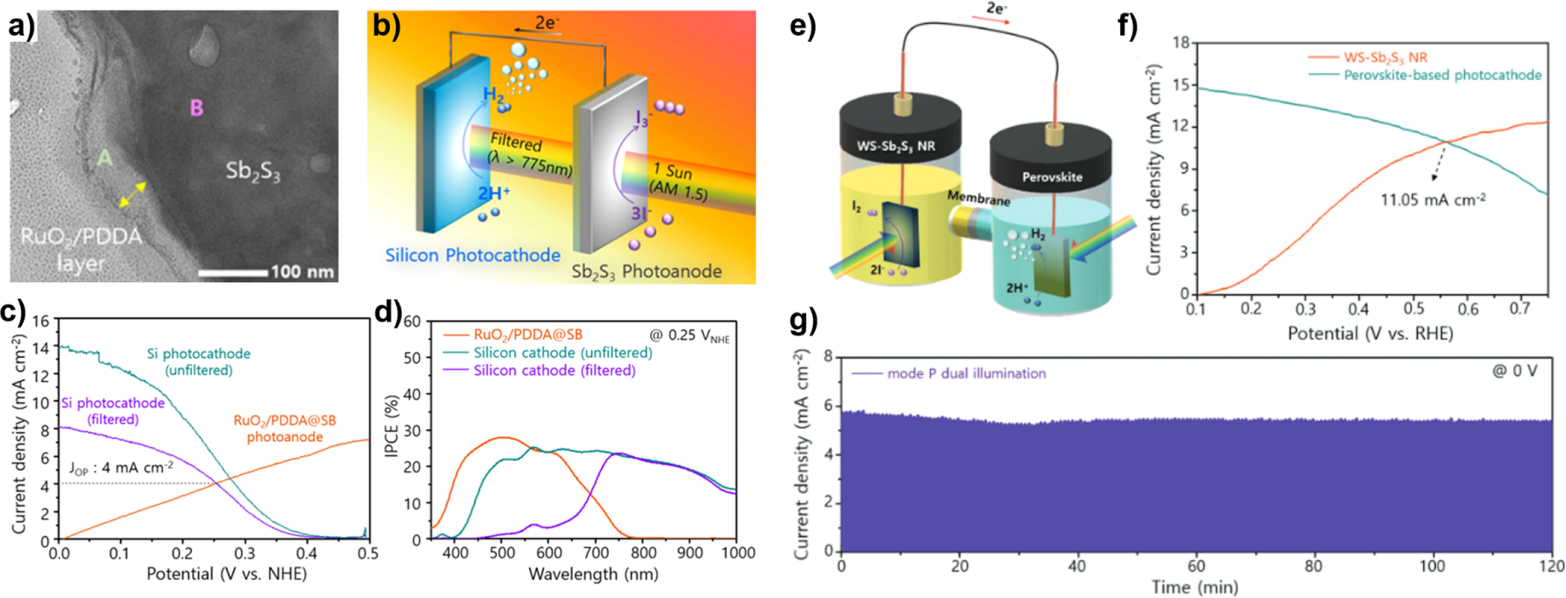
(a) TEM image of the Sb_2_S_3_|PDDA|RuO_2_ photoanode. (b) Scheme of a tandem device comprising a Sb_2_S_3_|PDDA|RuO_2_ photoanode and silicon photocathode operated in HI electrolyte (pH 1). (c) LSV curves for the Sb_2_S_3_|PDDA|RuO_2_ photoanode and silicon photocathode, showing the expected operating point and (d) corresponding IPCE spectra at 0.25 V_SHE_ for the Si||Sb_2_S_3_|PDDA|RuO_2_ tandem device. (a–d) Adapted with permission from ref 609. Copyright 2022 Royal Society of Chemistry. (e) Scheme of a parallel-illuminated tandem device composed of a WS_*x*_-passivated Sb_2_S_3_ photoanode for IOR in HI and perovskite-based photocathode for HER in H_2_SO_4_. (f) Corresponding LSV curves for each photoanode and photocathode and (g) the chronoamperometric test during parallel illumination without external bias. (e,f) Adapted with permission from ref 610. Copyright 2023 Wiley-VCH.

## 5 Conclusion

Addressing the growing demand for chemical fuels while leveraging inert atmospheric feedstocks is a grand scientific challenge of the 21st century. The promise of photoelectrochemistry, to channel the effectively unlimited energy of sunlight into fuels, is a solution to this problem. Although the last half-century has witnessed widespread adaptation of both photovoltaic and electrochemical technologies, efforts to couple these technologies for efficient and robust PEC applications remain hindered by poor optoelectronics or catalytic efficiency or stability.

The challenges for semiconductor photoelectrodes lie in developing and optimizing both their fundamental (synthesis, optoelectronics, and dopant) and applied (catalysis, stability, and morphology) characteristics. While the semiconductors utilized in commercialized PV devices afford excellent optoelectronic properties, they suffer from poor stability when exposed to electrolytes and often demonstrate poor catalytic properties. In contrast, stable and catalytically active metal oxides are generally compromised by poor optoelectronic properties (large band gaps and poor charge transport). Integration of additional materials for protection and charge extraction can enhance stability and catalytic performance, in select cases nearing theoretical maximum *j*_SC_, but additional solid|solid interfaces increase the number of factors that must be controlled, e.g., by introducing recombination centers that can reduce efficiency.

While photoelectrodes based on mature PV semiconductors can yield high η_STF_, some emerging semiconductors, derived from computational and empirical materials discovery efforts, show promise for successfully optimizing between optoelectronic properties, catalysis, and stability. Although photoanodes generally lag behind photocathodes in η_STF_ due to their intermediate band gaps, BiVO_4_ and Ta_3_N_5_ are two promising photoanode candidates that are nearing their theoretical efficiencies. BiVO_4_ is frequently coupled to promising photocathodes to demonstrate record overall η_STF_, where fabrication of semiconductor|semiconductor junctions can improve charge separation and transfer properties. Ta_3_N_5_ has seen a drastic rise in OER efficiency over the past decade, with *j*_SC_ now exceeding 10 mA cm^–2^. Perovskite oxynitride photoanodes likewise represent a phase space with significant potential for maturation, where the perovskite crystal structure space offers effectively unlimited tunability of intrinsic material properties, if defects and doping correlated to oxygen content can be sufficiently controlled. Similar progress has been made with novel photocathode semiconductors, with many chalcogenides (CIGS, Sb_2_Se_3_) approaching theoretical photocurrent limits. Ongoing materials discovery research has yielded promising photoelectrode candidates such as CuBi_2_O_4_ and other emerging oxides as well as nitrides, and will likely continue to do so using extant crystal structures as a starting point. For these semiconductors, rapid synthetic development to enable control of defects and elucidate properties will be critical to understanding how to improve catalytic efficiency and ensure stability. Although their respective band gaps and stability may prevent TiO_2_ and PV semiconductors from successful implementation in PEC systems, the depth of knowledge gained over 50 years of research on these semiconductors as photoelectrodes should be mined for lessons to improve the performance of emerging semiconductors. Utilizing a combined approach to address optoelectronics, catalysis, and stability is the path toward the development of a highly efficient photoelectrode for water splitting.

Recent advances in spectroscopic techniques have begun to lift the veil on the complex photoelectrode|electrolyte interface, informing on oxidation state, composition, and crystallography at active catalytic interfaces. Efforts are ongoing to further advance these techniques, and information garnered from illuminated, in situ measurements in particular will continue to drive fundamental understanding of PEC fuel formation. Informed by progress made in water splitting, continued investigation of PEC-driven, complex fuel-forming reactions (CO_2_RR, N_2_RR, and NO_3_^–^RR, and alternative oxidation reactions) may provide renewable sources of value-added products. PEC performance of these reactions can benefit not only from the lessons learned in the development of photoelectrodes for water splitting applications, but from semiconductors that are considered undesirable for HER or OER, because of, e.g., better stability at neutral pH than in strong electrolytes (for CO_2_RR) or small band gaps (for GOR). This may lead to rapid development of alternative fuel-forming reactions by PEC, if the challenge of selectivity can be overcome.

Although the last 50 years of PEC fuel-forming research has not yielded technoeconomic maturity in the form of a commercialized, stable and highly efficient photoelectrode, the chemical understanding of this field has progressed substantially. The design principles and understanding of photoelectrode semiconductors, as well as methods for their characterization, have reached previously unforeseen heights, and demonstrations of PEC-driven reactions beyond HER and OER are abundant. What is needed now is targeted strategies for improving the properties of individual semiconductors (many of which are discussed throughout this review), connected to full-device characterization of integrated photoelectrode architectures to identify failure modes and areas for improvement. With these approaches to improving the optoelectronics, catalysis, and stability of semiconductor photoelectrodes under way, researchers can consider reactor designs and real-world processes that can enhance PEC fuel production. Photoelectrochemistry is therefore poised to achieve its promise as a renewable method for producing fuels with the full integration of chemical knowledge acquired since its inception.

## Supplementary Material



## Notes

Comprehensive semiconductor valence and conduction band edge energies; tabulated theoretical and achieved photocurrents and first year of investigation; ICSD data for crystallographic illustrations; band edge energies and corresponding references; free energy data underlying Figure 36 (PDF)
