# Supplementary material for: Chemistry of Materials Underpinning Photoelectrochemical Solar Fuel Production
Source: Chem Rev. 2025 May 6;125(10):4768–839. doi: 10.1021/acs.chemrev.4c00258 (PMC12123630; doi:10.1021/acs.chemrev.4c00258)
Supplement: Supplementary file 1 [file cr4c00258_si_001.pdf]

Supplemental Information for:
Chemistry of Materials Underpinning Photoelectrochemical Solar Fuel Production

Zebulon G. Schichtl[†1]; O. Quinn Carvalho[†1]; Jeiwan Tan[1]; Simran S. Saund[1]; Debjit Ghoshal[1]; Logan M. Wilder[1]; Melissa K. Gish[1]; Adam C. Nielander[2]; Michaela Burke Stevens[2]; Ann L. Greenaway[1]*

[1]Materials Chemical and Computational Science Directorate, National Renewable Energy Laboratory, Golden, Colorado 80401, USA

[2]SUNCAT Center for Interface Science and Catalysis, SLAC National Accelerator Laboratory, 2575 Sand Hill Rd, Menlo Park, California 94025, USA

[†]indicates equal contribution
*corresponding author, ann.greenaway@nrel.gov

1. Supplemental Figures S-2
2. Supplemental Tables S-3
References S-8

1. Supplemental Figures

Figure S1. Compiled band edge information of the photoelectrodes discussed in the main text. The thermodynamic potentials for hydrogen evolution (HER) and oxygen evolution (OER) are denoted by the dashed black line and the dotted gray line, respectively. Table S8 lists the references for the obtaining experimental band edge information.

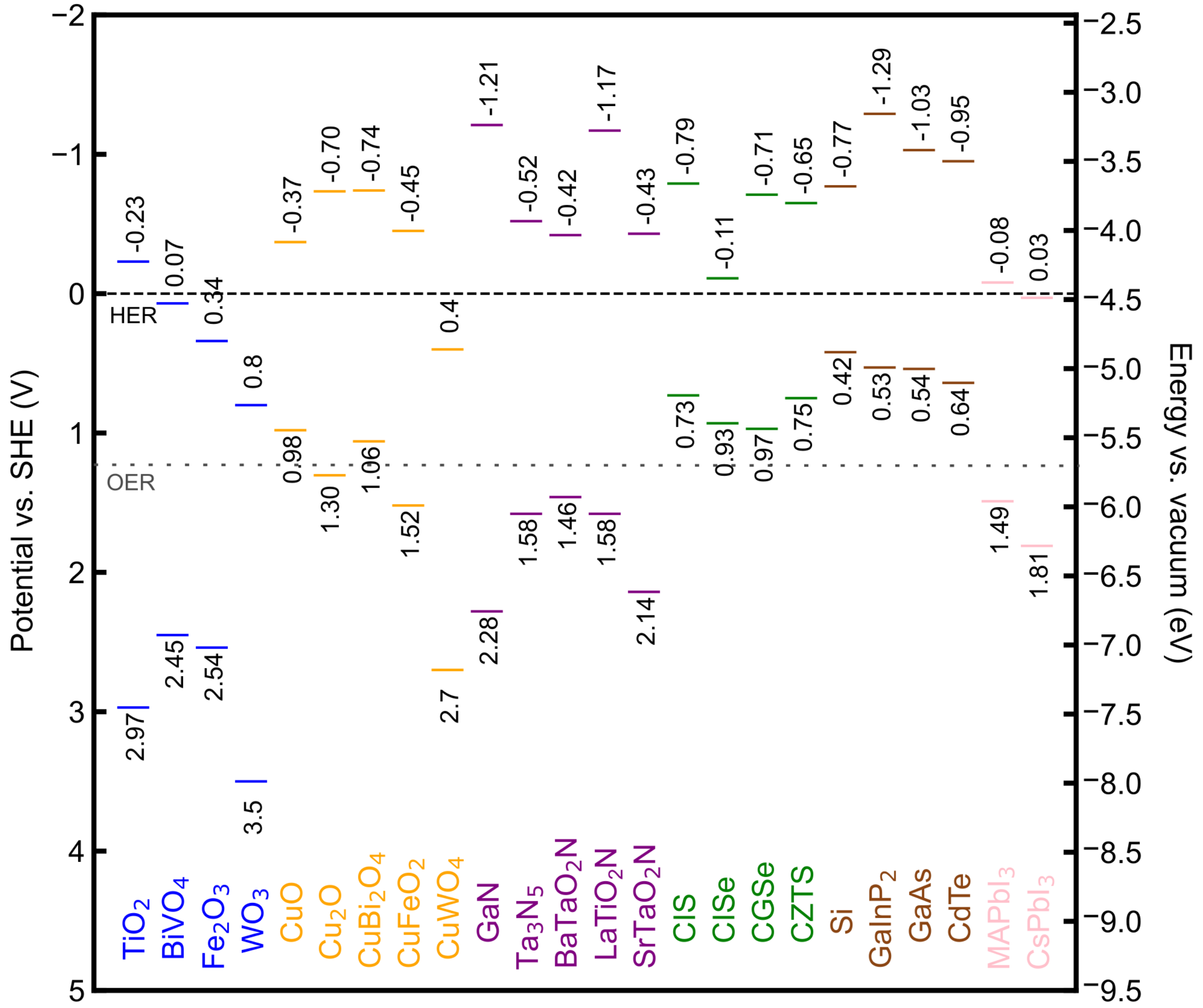

2. Supplemental Tables

Table S1. Theoretical and achieved short-circuit current density of metal oxides, their architecture and electrolyte, and first year published PEC investigation for data provided in Figure 4. Citations for achieved photocurrents and first year denoted in parentheses.

Class	Semiconductor	Photoanode/ Photocathode	Maximum j_{SC} mA cm^{-2}		First year
			Theoretical	Achieved	
Metal oxides	TiO_2 (rutile)	Photoanode	1.85	2.5 (1)	1972 (2)
		Architecture: TiO_{2-x}<br>Electrolyte: 1 M NaOH electrolyte			
	$BiVO_4$	Photoanode	7.5	6.1 (3)	2003 (4)
		Architecture: F:SnO_2|$BiVO_4$|CoPi<br>Electrolyte: pH 7 0.5 M H_3PO_4			
	Fe_2O_3	Photoanode	12.4	6 (5)	1976 (6)
		Architecture: Fe_2O_3|TiO_2|Co<br>Electrolyte: 1 M KOH			
	WO_3	Photoanode	6.05	4.5 (7)	1976 (8)
		Architecture: 0.4% Na:WO_3<br>Electrolyte: 1 M CH_3SO_3H with 0.1 mM $H_3PMo_{12}O_{40}$			

Table S2. Theoretical and achieved short-circuit current density of copper oxides, their architecture and electrolyte, and first year published PEC investigation for data provided in Figure 4. Citations for achieved photocurrents and first year denoted in parentheses.

Class	Semiconductor	Photoanode/ Photocathode	Maximum j_{SC} mA cm^{-2}		First year
			Theoretical	Achieved	
Copper oxides	Cu_2O	Photocathode	14.7	10 (9)	2000 (10)
		Architecture: F:SnO_2|*Cu_2O*|*Ga_2O_3*|TiO_2|RuO_x<br>Electrolyte: pH 5 0.5 M Na_2SO_4 with 0.1 M $Na_xH_{3-x}PO_4$			
	CuO	Photocathode	29.1	5.4 (11)	2000 (10)
		Architecture: *p-(CuO/Al:CuO)*|*n-Al:ZnO*|TiO_2|Au-Pd<br>Electrolyte: pH 5.84 0.1 M Na_2SO_4			
	$CuBi_2O_4$	Photocathode	19	1.55 (12)	2007 (13)
		Architecture: F:SnO_2|$CuBi_2O_4$<br>Electrolyte: 1 M NaOH			
	$CuFeO_2$	Photocathode	27	4.85 (14)	2005 (15)
		Architecture: F:SnO_2|$CuFeO_2$|C_{60}|(Co,Fe)OOH<br>Electrolyte: 1 M NaOH			
	$CuWO_4$	Photoanode	8.2	0.5 (16)	1982 (17)
		Architecture: F:SnO_2|$CuWO_4$|Co_3O_4<br>Electrolyte: pH 7 0.1 M $K_xH_{3-x}PO_4$			

Table S3. Theoretical and achieved short-circuit current density of nitrides, their architecture and electrolyte, and first year published PEC investigation for data provided in Figure 4. Citations for achieved photocurrents and first year denoted in parentheses.

Class	Semiconductor	Photoanode/ Photocathode	Maximum j_{SC} mA cm^{-2}		First year
			Theoretical	Achieved	
Nitrides	GaN	Photocathode	0.61	0.14 [18]	1995 [19]
		Architecture: Al_2O_3|GaN<br>Electrolyte: 1 M NaCl			
	Ta_3N_5	Photoanode	12.4	10.96 [20]	2002 [21]
		Architecture: Ta_3N_5|$FeNiCoO_x$<br>Electrolyte: ~0.4 M KOH			
	$BaTaO_2N$	Photoanode	17.5	1.65 [22]	2013 [23]
		Architecture: F:SnO_2|$BaTaO_2N$|CoO<br>Electrolyte: 0.1 M KOH			
	$CaTaO_2N$	Photoanode	5.89	0.25 [24]	2020 [25]
		Architecture: Al_2O_3|GaN|$CaTaO_2N$|$FeNiO_x$<br>Electrolyte: 0.1 M NaOH			
	$SrNbO_2N$	Photoanode	19.72	1.5 [26]	2011 [27]
		Architecture: Nb|Ti|$SrNbO_2N$<br>Electrolyte: pH 13 0.2 M $Na_xH_{3-x}PO_4$			

Table S4. Theoretical and achieved short-circuit current density of chalcogenides, their architecture and electrolyte, and first year published PEC investigation for data provided in Figure 4. Citations for achieved photocurrents and first year denoted in parentheses.

Class	Semiconductor	Photoanode/ Photocathode	Maximum j_{SC} mA cm^{-2}		First year
			Theoretical	Achieved	
Chalcogenides	$CuIn_{1-x}Ga_xSe_2$	Photocathode	40	34 [28]	2013 [29]
		Architecture: $Cu_{0.82}(In_{0.61}Ga_{0.39})Se_2$|*CdS*|*ZnO*|$TiO_2$|Pt<br>Electrolyte: 0.5 M H_2SO_4			
	$CuZn_{1-x}Sn_xS_2$	Photocathode	29	17 [30]	2010 [31]
		Architecture: $Cu_2Cd_{0.4}Zn_{0.6}SnS_4$|*CdS*|TiMo|Pt<br>Electrolyte: pH 7 1 M $K_xH_{3-x}PO_4$			
	Sb_2Se_3	Photocathode	38	30 [32]	2017 [33]
		Architecture: F:SnO_2|Au|Sb_2Se_3|TiO_2|Pt<br>Electrolyte: 0.1 M H_2SO_4			
	Cu_3BiS_3	Photocathode	20	7.8 [34]	2012 [35]
		Architecture: F:SnO_2|Au|Bi_2S_3-Cu_3BiS_3|*CdS*|TiO_2|Pt<br>Electrolyte: pH 7 0.5 M $K_xH_{3-x}PO_4$			

Table S5. Theoretical and achieved short-circuit current density of mature PV semiconductors, their architecture and electrolyte, and first year published PEC investigation for data provided in Figure 4. Citations for achieved photocurrents and first year denoted in parentheses.

Class	Semiconductor	Photoanode/ Photocathode	Maximum j_{SC} mA cm^{-2}		First year
			Theoretical	Achieved	
Mature PV Semiconductors	p-CdTe	Photocathode	29	22 (36)	1977 (37)
		Architecture: F:SnO_2|Au|Cu|*CdTe*|*CdS*|Pt<br>Electrolyte: pH 8 0.5 M $K_xH_{3-x}PO_4$			
	n-CdTe	Photoanode	29	18.5 (38)	1977 (37)
		Architecture: F:SnO_2|*CdS*|*CdTe*|TiO_2|NiO_x<br>Electrolyte: 1 M KOH			
	p-Si	Photocathode	43.96	35.5 (39)	1980 (40)
		Architecture: Al|n^+/p-Si|Ni-Mo<br>Electrolyte: 0.1 M H_2SO_4			
	n-Si	Photoanode	43.96	28 (41)	1980 (40)
		Architecture: n-Si|SiO_x|CoO_x|NiO_x<br>Electrolyte: 1 M KOH			
	GaAs	Photoanode	31.84	14.3 (42)	1965 (43)
		Architecture: np^+-GaAs|TiO_2|Ni<br>Electrolyte: 1 M KOH			

Table S6. Theoretical and achieved short-circuit current density of select tandem (two semiconductor photoabsorber) photoelectrode devices, their architecture and electrolyte, and first year published PEC investigation for data provided in Figure 4. Citations for achieved photocurrents denoted in parentheses. First year not considered for tandem device architectures, where the effectively infinite combinations of compositions and phases make such an assessment impracticable.

Class	Semiconductor	Photoanode/ Photocathode	Maximum j_{SC} mA cm^{-2}		First year
			Theoretical	Achieved	
Select Tandem Devices	WO_3|$BiVO_4$	Photoanode	7.5	6.72 (44)	---
		Architecture: ITO|Pt|ITO|WO_3|$BiVO_4$|CoPi<br>Electrolyte: pH 7 $K_xH_{3-x}PO_4$ of unknown strength			
	Si|*GaN*|*InGaN*	Photocathode	43.96	40.6 (45)	---
		Architecture: p^+-Si|*p-Si*|n^+-Si|n^{++}-GaN|$In_{0.4}Ga_{0.6}N$|p^{++}-GaN|*p-* $In_{0.4}Ga_{0.6}N$ |Pt<br>Electrolyte: 1 M HBr			
	$Ga_{0.51}In_{0.49}P$|*GaAs*	Photocathode	31.84	12.5 (46)	---
		Architecture: $Ga_{0.51}In_{0.49}P_2$|*GaAs*|Pt<br>Electrolyte: 0.5 M H_2SO_4			
	$Ga_{0.89}In_{0.11}As$|$Ga_{0.51}In_{0.49}P$	Photocathode	39.92	13.2 (47)	---
		Architecture: $Ga_{0.89}In_{0.11}As$|$Ga_{0.51}In_{0.49}P$|PtRu<br>Electrolyte: 3 M H_2SO_4			

Table S7. ICSD code and citations for crystal structures displayed throughout the main text.

Class	Stoichiometry	Structure	ICSD no.	Ref.
Metal oxides	TiO_2	Rutile	9161	[48]
	$BiVO_4$	Tetragonal	22261	[49]
	Fe_2O_3	Trigonal	15840	[50]
	WO_3	Triclinic	80056	[51]
Copper oxides	Cu_2O	Cubic	52043	[52]
	CuO	Monoclinic	16025	[53]
	$CuBi_2O_4$	Tetragonal	68815	[54]
	$CuFeO_2$	Trigonal	31918	[55]
	$CuWO_4$	Triclinic	24339	[56]
Nitrides	GaN	Hexagonal	34476	[57]
	Ta_3N_5	Orthorhombic	66533	[58]
	$BaTaO_2N$	Perovskite	122767	[59]
	$CaTaO_2N$	Perovskite	113383	[60]
	$SrNbO_2N$	Perovskite	24604	[61]
Chalcogenides	CIGSe	Tetrahedral	163563	[62]
	CZTS	Tetragonal	12710	[63]
	Sb_2Se_3	Orthorhombic	16680	[64]
	Cu_3BiS_3	Orthorhombic	14305	[65]
Mature PV Semiconductors	CdTe	Cubic	93942	[66]
	Si	Cubic	51688	[67]
	$GaInP_2$	Cubic	53576	[68]
	GaAs	Cubic	107946	[69]

Table S8. Valence band edge maximum (VBM), conduction band edge minimum (CBM), and bandgap energy values extracted from the literature.

Class	Stoichiometry	VBM (V_{SHE})	CBM (V_{SHE})	Bandgap (eV)	Ref.
Metal oxides	TiO_2 (rutile)	2.97	–0.23	3.2	[70]
	$BiVO_4$	2.45	0.07	2.38	[71]
	Fe_2O_3	2.54	0.34	2.2	[72]
	WO_3	3.50	0.80	2.7	[72]
Copper oxides	Cu_2O	1.30	–0.70	2.0	[73]
	CuO	0.98	–0.37	1.35	[74]
	$CuBi_2O_4$	1.06	–0.74	1.8	[75]
	$CuFeO_2$	1.52	–0.45	1.97	[76]
	$CuWO_4$	2.70	0.40	2.29	[77]
Nitrides	GaN	2.28	–1.21	3.50	[70]
	Ta_3N_5	1.58	–0.52	2.1	[78]
	$BaTaO_2N$	1.46	–0.42	1.88	[71]
	$LaTiO_2N$	1.58	–1.17	2.74	[79]
	$SrTaO_2N$	2.14	–0.43	2.57	[80]
Chalcogenides	CIS	0.73	–0.79	1.52	[81]
	CZTS	0.75	–0.65	1.4	[82]
	Sb_2Se_3	0.80	–0.57	1.37	[76]
	Cu_3BiS_3	0.54	–0.64	1.18	[83]
Mature PV Semiconductors	CdTe	0.64	–0.95	1.59	[70]
	Si	0.42	–0.77	1.19	[70]
	GaAs	0.54	–1.03	1.57	[70]
	$GaInP_2$	0.53	–1.29	1.82	[84]

Table S9. Formal reduction potentials for common CO_2RR products (data underlying Figure 36).

**Reaction**	**Products**	**E^{o} (V vs. SHE)**[85–87]
$CO_2 + 1e^- + 2H^+$	$CO_2^{\bullet-} + H_2O$	–1.90
$CO_2 + 2e^- + 2H^+$	$CO + H_2O$	–0.53
$CO_2 + 2e^- + 2H^+$	HCOOH	–0.61
$CO_2 + 4e^- + 4H^+$	HCHO	–0.48
$CO_2 + 6e^- + 6H^+$	$CH_3OH + H_2O$	–0.38
$CO_2 + 8e^- + 8H^+$	$CH_4 + 2H_2O$	–0.24
$2CO_2 + 8e^- + 8H^+$	$CH_3COOH + 2H_2O$	–0.30
$2CO_2 + 10e^- + 10H^+$	$CH_3CHO + 3H_2O$	–0.35
$2CO_2 + 12e^- + 12H^+$	$C_2H_5OH + 3H_2O$	–0.33
$2CO_2 + 12e^- + 12H^+$	$C_2H_4 + 4H_2O$	–0.34
$2CO_2 + 14e^- + 14H^+$	$C_2H_6 + 4H_2O$	–0.27
$3CO_2 + 18e^- + 18H^+$	$C_3H_7OH + 5H_2O$	–0.31
$3CO_2 + 20e^- + 20H^+$	$C_3H_8 + 6H_2O$	–0.33

References

(1) Wang, G.; Wang, H.; Ling, Y.; Tang, Y.; Yang, X.; Fitzmorris, R. C.; Wang, C.; Zhang, J. Z.; Li, Y. Hydrogen-Treated TiO_2 Nanowire Arrays for Photoelectrochemical Water Splitting. *Nano Lett.* **2011**, *11*, 3026–3033.

(2) Fujishima, A.; Honda, K. Electrochemical Photolysis of Water at a Semiconductor Electrode. *Nature* **1972**, *238*, 37–38.

(3) Han, H. S.; Shin, S.; Kim, D. H.; Park, I. J.; Kim, J. S.; Huang, P.-S.; Lee, J.-K.; Cho, I. S.; Zheng, X. Boosting the Solar Water Oxidation Performance of a BiVO4 Photoanode by Crystallographic Orientation Control. *Energy Environ. Sci.* **2018**, *11*, 1299–1306.

(4) Sayama, K.; Nomura, A.; Zou, Z.; Abe, R.; Abe, Y.; Arakawa, H. Photoelectrochemical Decomposition of Water on Nanocrystalline BiVO 4 Film Electrodes under Visible Light. *Chem. Commun.* **2003**, *0*, 2908–2909.

(5) Jeon, T. H.; Moon, G.; Park, H.; Choi, W. Ultra-Efficient and Durable Photoelectrochemical Water Oxidation Using Elaborately Designed Hematite Nanorod Arrays. *Nano Energy* **2017**, *39*, 211–218.

(6) Hardee, K. L.; Bard, A. J. Semiconductor Electrodes: X . Photoelectrochemical Behavior of Several Polycrystalline Metal Oxide Electrodes in Aqueous Solutions. *J. Electrochem. Soc.* **1977**, *124*, 215.

(7) Sarnowska, M.; Bienkowski, K.; Barczuk, P. J.; Solarska, R.; Augustynski, J. Highly Efficient and Stable Solar Water Splitting at (Na)WO_3 Photoanodes in Acidic Electrolyte Assisted by Non-Noble Metal Oxygen Evolution Catalyst. *Adv. Energy Mater.* **2016**, *6*, 1600526.

(8) Hodes, G.; Cahen, D.; Manassen, J. Tungsten Trioxide as a Photoanode for a Photoelectrochemical Cell (PEC). *Nature* **1976**, *260*, 312–313.

(9) Pan, L.; Kim, J. H.; Mayer, M. T.; Son, M.-K.; Ummadisingu, A.; Lee, J. S.; Hagfeldt, A.; Luo, J.; Grätzel, M. Boosting the Performance of Cu_2O Photocathodes for Unassisted Solar Water Splitting Devices. *Nat. Catal.* **2018**, *1*, 412–420.

(10) Yoon, K. H.; Choi, W. J.; Kang, D. H. Photoelectrochemical Properties of Copper Oxide Thin Films Coated on an *n*-Si Substrate. *Thin Solid Films* **2000**, *372*, 250–256.

(11) Masudy-Panah, S.; Kong Eugene, Y.-J.; Dasineh Khiavi, N.; Katal, R.; Gong, X. Aluminum-Incorporated p-CuO/n-ZnO Photocathode Coated with Nanocrystal-Engineered TiO_2 Protective Layer for Photoelectrochemical Water Splitting and Hydrogen Generation. *J. Mater. Chem. A* **2018**, *6*, 11951–11965.

(12) Li, J.; Griep, M.; Choi, Y.; Chu, D. Photoelectrochemical Overall Water Splitting with Textured CuBi2O4 as a Photocathode. *Chem. Commun.* **2018**, *54*, 3331–3334.

(13) Arai, T.; Konishi, Y.; Iwasaki, Y.; Sugihara, H.; Sayama, K. High-Throughput Screening Using Porous Photoelectrode for the Development of Visible-Light-Responsive Semiconductors. *J. Comb. Chem.* **2007**, *9*, 574–581.

(14) Oh, Y.; Yang, W.; Tan, J.; Lee, H.; Park, J.; Moon, J. Boosting Visible Light Harvesting in p-Type Ternary Oxides for Solar-to-Hydrogen Conversion Using Inverse Opal Structure. *Adv. Funct. Mater.* **2019**, *29*, 1900194.

(15) Younsi, M.; Aider, A.; Bouguelia, A.; Trari, M. Visible Light-Induced Hydrogen over CuFeO2 via S2O32− Oxidation. *Sol. Energy* **2005**, *78*, 574–580.

(16) Tian, C. M.; Jiang, M.; Tang, D.; Qiao, L.; Xiao, H. Y.; Oropeza, F. E.; Hofmann, J. P.; Hensen, E. J. M.; Tadich, A.; Li, W.; et al. Elucidating the Electronic Structure of $CuWO_4$

Thin Films for Enhanced Photoelectrochemical Water Splitting. *J. Mater. Chem. A* **2019**, *7*, 11895–11907.
(17) Benko, F. A.; MacLaurin, C. L.; Koffyberg, F. P. CuWO4 and Cu3WO6 as Anodes for the Photoelectrolysis of Water. *Mater. Res. Bull.* **1982**, *17*, 133–136.
(18) Pal, Y.; Anthony Raja, M.; Madhumitha, M.; Nikita, A.; Neethu, A. Fabrication and Characterization of Gallium Nitride Thin Film Deposited on a Sapphire Substrate for Photoelectrochemical Water Splitting Applications. *Optik* **2021**, *226*, 165410.
(19) Kocha, S. S.; Peterson, M. W.; Arent, D. J.; Redwing, J. M.; Tischler, M. A.; Turner, J. A. Electrochemical Investigation of the Gallium Nitride-Aqueous Electrolyte Interface. *J. Electrochem. Soc.* **1995**, *142*, L238.
(20) Pihosh, Y.; Nandal, V.; Shoji, R.; Bekarevich, R.; Higashi, T.; Nicolosi, V.; Matsuzaki, H.; Seki, K.; Domen, K. Nanostructured Tantalum Nitride for Enhanced Solar Water Splitting. *ACS Energy Lett.* **2023**, *8*, 2106–2112.
(21) Hitoki, G.; Takata, T.; Kondo, J. N.; Hara, M.; Kobayashi, H.; Domen, K. An Oxynitride, TaON, as an Efficient Water Oxidation Photocatalyst under Visible Light Irradiation (Λ≤ 500 Nm). *Chem. Commun.* **2002**, Number, 1698–1699.
(22) Wei, S.; Chang, S.; Yang, F.; Fu, Z.; Liu, G.; Xu, X. Stable and Efficient Solar-Driven Photoelectrochemical Water Splitting into H2 and O2 Based on a BaTaO2N Photoanode Decorated with CoO Microflowers. *Chem. Commun.* **2021**, *57*, 4412–4415.
(23) Higashi, M.; Domen, K.; Abe, R. Fabrication of an Efficient BaTaO2N Photoanode Harvesting a Wide Range of Visible Light for Water Splitting. *J. Am. Chem. Soc.* **2013**, *135*, 10238–10241.
(24) Lu, C.; Chen, J.; Piętak, K.; Rokicińska, A.; Kuśtrowski, P.; Dronskowski, R.; Yuan, J.; Budnyk, S.; Złotnik, S.; Coridan, R. H.; et al. Semi Transparent Three-Dimensional Macroporous Quaternary Oxynitride Photoanodes for Photoelectrochemical Water Oxidation. *Chem. Mater.* **2022**, *34*, 6902–6911.
(25) Ma, Z.; Jaworski, A.; George, J.; Rokicinska, A.; Thersleff, T.; Budnyak, T. M.; Hautier, G.; Pell, A. J.; Dronskowski, R.; Kuśtrowski, P.; et al. Exploring the Origins of Improved Photocurrent by Acidic Treatment for Quaternary Tantalum-Based Oxynitride Photoanodes on the Example of CaTaO2N. *J. Phys. Chem. C* **2020**, *124*, 152–160.
(26) Kodera, M.; Urabe, H.; Katayama, M.; Hisatomi, T.; Minegishi, T.; Domen, K. Effects of Flux Synthesis on SrNbO2N Particles for Photoelectrochemical Water Splitting. *J. Mater. Chem. A* **2016**, *4*, 7658–7664.
(27) Maeda, K.; Higashi, M.; Siritanaratkul, B.; Abe, R.; Domen, K. SrNbO2N as a Water-Splitting Photoanode with a Wide Visible-Light Absorption Band. *J. Am. Chem. Soc.* **2011**, *133*, 12334–12337.
(28) Luo, J.; Li, Z.; Nishiwaki, S.; Schreier, M.; Mayer, M. T.; Cendula, P.; Lee, Y. H.; Fu, K.; Cao, A.; Nazeeruddin, M. K.; et al. Targeting Ideal Dual-Absorber Tandem Water Splitting Using Perovskite Photovoltaics and CuInxGa1-xSe2 Photocathodes. *Adv. Energy Mater.* **2015**, *5*, 1501520.
(29) Fernández, A. M.; Dheree, N.; Turner, J. A.; Martínez, A. M.; Arriaga, L. G.; Cano, U. Photoelectrochemical Characterization of the Cu(In,Ga)S2 Thin Film Prepared by Evaporation. *Sol. Energy Mater. Sol. Cells* **2005**, *85*, 251–259.
(30) Tay, Y. F.; Kaneko, H.; Chiam, S. Y.; Lie, S.; Zheng, Q.; Wu, B.; Hadke, S. S.; Su, Z.; Bassi, P. S.; Bishop, D.; et al. Solution-Processed Cd-Substituted CZTS Photocathode for Efficient Solar Hydrogen Evolution from Neutral Water. *Joule* **2018**, *2*, 537–548.

(31) Yokoyama, D.; Minegishi, T.; Jimbo, K.; Hisatomi, T.; Ma, G.; Katayama, M.; Kubota, J.; Katagiri, H.; Domen, K. H2 Evolution from Water on Modified Cu2ZnSnS4 Photoelectrode under Solar Light. *Appl. Phys. Express* **2010**, *3*, 101202.
(32) Park, J.; Yang, W.; Tan, J.; Lee, H.; Yun, J. W.; Shim, S. G.; Park, Y. S.; Moon, J. Hierarchal Nanorod-Derived Bilayer Strategy to Enhance the Photocurrent Density of Sb_2Se_3 Photocathodes for Photoelectrochemical Water Splitting. *ACS Energy Lett.* **2020**, *5*, 136–145.
(33) Kim, J.; Yang, W.; Oh, Y.; Lee, H.; Lee, S.; Shin, H.; Kim, J.; Moon, J. Self-Oriented Sb_2Se_3 Nanoneedle Photocathodes for Water Splitting Obtained by a Simple Spin-Coating Method. *J. Mater. Chem. A* **2017**, *5*, 2180–2187.
(34) Moon, S.; Park, J.; Lee, H.; Yang, J. W.; Yun, J.; Park, Y. S.; Lee, J.; Im, H.; Jang, H. W.; Yang, W.; et al. Bi2S3-Cu3BiS3 Mixed Phase Interlayer for High-Performance Cu3BiS3-Photocathode for 2.33% Unassisted Solar Water Splitting Efficiency. *Adv. Sci.* **2023**, *10*, 2206286.
(35) Colombara, D.; Peter, L. M.; Hutchings, K.; Rogers, K. D.; Schäfer, S.; Dufton, J. T. R.; Islam, M. S. Formation of Cu3BiS3 Thin Films via Sulfurization of Bi–Cu Metal Precursors. *Thin Solid Films* **2012**, *520*, 5165–5171.
(36) Su, J.; Minegishi, T.; Kageshima, Y.; Kobayashi, H.; Hisatomi, T.; Higashi, T.; Katayama, M.; Domen, K. CdTe-Based Photoanode for Oxygen Evolution from Water under Simulated Sunlight. *J. Phys. Chem. Lett.* **2017**, *8*, 5712–5717.
(37) Ohashi, K.; McCann, J.; Bockris, J. O. Hydrogen and Electricity from Water and Light. *Int. J. Energy Res.* **1977**, *1*, 259–277.
(38) Chen, X.; Shen, X.; Shen, S.; Reese, M. O.; Hu, S. Stable CdTe Photoanodes with Energetics Matching Those of a Coating Intermediate Band. *ACS Energy Lett.* **2020**, *5*, 1865–1871.
(39) Vijselaar, W.; Westerik, P.; Veerbeek, J.; Tiggelaar, R. M.; Berenschot, E.; Tas, N. R.; Gardeniers, H.; Huskens, J. Spatial Decoupling of Light Absorption and Catalytic Activity of Ni–Mo-Loaded High-Aspect-Ratio Silicon Microwire Photocathodes. *Nat. Energy* **2018**, *3*, 185–192.
(40) Bookbinder, D. C.; Bruce, J. A.; Dominey, R. N.; Lewis, N. S.; Wrighton, M. S. Synthesis and Characterization of a Photosensitive Interface for Hydrogen Generation: Chemically Modified p-Type Semiconducting Silicon Photocathodes. *Proc. Natl. Acad. Sci.* **1980**, *77*, 6280–6284.
(41) Zhou, X.; Liu, R.; Sun, K.; Friedrich, D.; McDowell, M. T.; Yang, F.; Omelchenko, S. T.; Saadi, F. H.; Nielander, A. C.; Yalamanchili, S.; et al. Interface Engineering of the Photoelectrochemical Performance of Ni-Oxide-Coated n-Si Photoanodes by Atomic-Layer Deposition of Ultrathin Films of Cobalt Oxide. *Energy Environ. Sci.* **2015**, *8*, 2644–2649.
(42) Hu, S.; Shaner, M. R.; Beardslee, J. A.; Lichterman, M.; Brunschwig, B. S.; Lewis, N. S. Amorphous TiO_2 Coatings Stabilize Si, GaAs, and GaP Photoanodes for Efficient Water Oxidation. *Science* **2014**, *344*, 1005–1009.
(43) Gerischer, H.; Hoffmann-Perez, M.; Mindt, W. Über den chemischen Zustand und das elektrische Moment der Oberfläche von Germanium in Kontakt mit wäßrigen Elektrolytlösungen. *Berichte Bunsenges. Für Phys. Chem.* **1965**, *69*, 130–138.
(44) Pihosh, Y.; Turkevych, I.; Mawatari, K.; Uemura, J.; Kazoe, Y.; Kosar, S.; Makita, K.; Sugaya, T.; Matsui, T.; Fujita, D.; et al. Photocatalytic Generation of Hydrogen by Core-

Shell WO3/BiVO4 Nanorods with Ultimate Water Splitting Efficiency. *Sci. Rep.* **2015**, *5*, 11141.
(45) Fan, S.; AlOtaibi, B.; Woo, S. Y.; Wang, Y.; Botton, G. A.; Mi, Z. High Efficiency Solar-to-Hydrogen Conversion on a Monolithically Integrated InGaN/GaN/Si Adaptive Tunnel Junction Photocathode. *Nano Lett.* **2015**, *15*, 2721–2726.
(46) Lim, H.; Young, J. L.; Geisz, J. F.; Friedman, D. J.; Deutsch, T. G.; Yoon, J. High Performance III-V Photoelectrodes for Solar Water Splitting via Synergistically Tailored Structure and Stoichiometry. *Nat. Commun.* **2019**, *10*, 3388.
(47) Young, J. L.; Steiner, M. A.; Döscher, H.; France, R. M.; Turner, J. A.; Deutsch, T. G. Direct Solar-to-Hydrogen Conversion via Inverted Metamorphic Multi-Junction Semiconductor Architectures. *Nat. Energy* **2017**, *2*, 17028.
(48) Baur, W. H.; Khan, A. A. Rutile-Type Compounds. IV. SiO2, GeO2 and a Comparison with Other Rutile-Type Structures. *Acta Crystallogr. B* **1971**, *27*, 2133–2139.
(49) Qurashi, M. M.; Barnes, W. H. The Structure of Pucherite, BiVO4. *Am. Mineral.* **1953**, *38*, 489–500.
(50) Blake, R. L.; Hessevick, R. E.; Zoltai, T.; Finger, L. W. Refinement of the Hematite Structure. *Am. Mineral.* **1966**, *51*, 123–129.
(51) Woodward, P. M.; Sleight, A. W.; Vogt, T. Structure Refinement of Triclinic Tungsten Trioxide. *J. Phys. Chem. Solids* **1995**, *56*, 1305–1315.
(52) Kirfel, A.; Eichhorn, K. Accurate Structure Analysis with Synchrotron Radiation. The Electron Density in Al2O3 and Cu2O. *Acta Crystallogr. A* **1990**, *46*, 271–284.
(53) Åsbrink, S.; Norrby, L.-J. A Refinement of the Crystal Structure of Copper(II) Oxide with a Discussion of Some Exceptional e.s.d.'s. *Acta Crystallogr. B* **1970**, *26*, 8–15.
(54) Garcia-Munoz, J. L.; Rodriguez-Carvajal, J.; Sapina, F.; Sanchis, M. J.; Ibanez, R.; Beltran-Porter, D. Crystal and Magnetic Structures of Bi2CuO4. *J. Phys. Condens. Matter* **1990**, *2*, 2205.
(55) Shannon, R. D.; Prewitt, C. T.; Rogers, D. B. Chemistry of Noble Metal Oxides. II. Crystal Structures of Platinum Cobalt Dioxide, Palladium Cobalt Dioxide, Coppper Iron Dioxide, and Silver Iron Dioxide. *Inorg. Chem.* **1971**, *10*, 719–723.
(56) Gebert, E.; Kihlborg, L.; Santesson, J.; Cyvin, S. J.; Hagen, G. On the Crystal Structure of Copper Wolframate. *Acta Chem. Scand.* **1967**, *21*, 2575–2576.
(57) Schulz, H.; Thiemann, K. H. Crystal Structure Refinement of AlN and GaN. *Solid State Commun.* **1977**, *23*, 815–819.
(58) Brese, N. E.; O'Keeffe, M.; Rauch, P.; DiSalvo, F. J. Structure of Ta3N5 at 16 K by Time-of-Flight Neutron Diffraction. *Acta Crystallogr. C* **1991**, *47*, 2291–2294.
(59) Wang, C.-H.; Kennedy, B. J.; Menezes de Oliveira, A. L.; Polt, J.; Knight, K. S. The Impact of Anion Ordering on Octahedra Distortion and Phase Transitions in SrTaO2N and BaTaO2N. *Acta Crystallogr. Sect. B Struct. Sci. Cryst. Eng. Mater.* **2017**, *73*, 389–398.
(60) Wang, X.; Jiang, B.; Zhang, Y.; Kim, Y.-I.; Page, K. Influence of Cation Size on the Local Atomic Structure and Electronic Properties of Ta Perovskite Oxynitrides. *Inorg. Chem.* **2021**, *60*, 14190–14201.
(61) Fujii, K.; Shimada, K.; Yashima, M. Crystal-Structure and Electron-Density Analyses of the Perovskite-Type Oxynitrides BaNbO2N and SrNbO2N through Synchrotron X-Ray Powder Diffraction. *J. Ceram. Soc. Jpn.* **2017**, *125*, 808–810.

(62) Souilah, M.; Rocquefelte, X.; Lafond, A.; Guillot-Deudon, C.; Morniroli, J.-P.; Kessler, J. Crystal Structure Re-Investigation in Wide Band Gap CIGSe Compounds. *Thin Solid Films* **2009**, *517*, 2145–2148.
(63) Schorr, S.; Hoebler, H.-J.; Tovar, M. A Neutron Diffraction Study of the Stannite-Kesterite Solid Solution Series. *Eur. J. Mineral.* **2007**, Number, 65–73.
(64) Tideswell, N. W.; Kruse, F. H.; McCullough, J. D. The Crystal Structure of Antimony Selenide, Sb2Se3. *Acta Crystallogr.* **1957**, *10*, 99–102.
(65) Kocman, V.; Nuffield, E. W. The Crystal Structure of Wittichenite, Cu3BiS3. *Acta Crystallogr. B* **1973**, *29*, 2528–2535.
(66) Rabadanov, M. Kh.; Verin, I. A.; Ivanov, Yu. M.; Simonov, V. I. Refinement of the Atomic Structure of CdTe Single Crystals. *Crystallogr. Rep.* **2001**, *46*, 636–641.
(67) Többens, D. M.; Stüßer, N.; Knorr, K.; Mayer, H. M.; Lampert, G. E9: The New High-Resolution Neutron Powder Diffractometer at the Berlin Neutron Scattering Center. *Mater. Sci. Forum* **2001**, *378–381*, 288–293.
(68) Enstrom, R. E.; Zanzucchi, P. J.; Appert, J. R. Optical Properties of Vapor-grown Inx Ga1−xAs Epitaxial Films on GaAs and Inx Ga1−xP Substrates. *J. Appl. Phys.* **1974**, *45*, 300–306.
(69) Stevenson, A. W. Thermal Vibrations and Bonding in GaAs: An Extended-Face Crystal Study. *Acta Crystallogr. A* **1994**, *50*, 621–632.
(70) Van de Walle, C. G.; Neugebauer, J. Universal Alignment of Hydrogen Levels in Semiconductors, Insulators and Solutions. *Nature* **2003**, *423*, 626–628.
(71) Seo, J.; Nishiyama, H.; Yamada, T.; Domen, K. Visible-Light-Responsive Photoanodes for Highly Active, Stable Water Oxidation. *Angew. Chem. Int. Ed.* **2018**, *57*, 8396–8415.
(72) Xu, Y.; Schoonen, M. A. A. The Absolute Energy Positions of Conduction and Valence Bands of Selected Semiconducting Minerals. *Am. Mineral.* **2000**, *85*, 543–556.
(73) Wick, R.; Tilley, S. D. Photovoltaic and Photoelectrochemical Solar Energy Conversion with Cu2O. *J. Phys. Chem. C* **2015**, *119*, 26243–26257.
(74) Koffyberg, F. P.; Benko, F. A. A Photoelectrochemical Determination of the Position of the Conduction and Valence Band Edges of *p*-type CuO. *J. Appl. Phys.* **1982**, *53*, 1173–1177.
(75) Oropeza, F. E.; Dzade, N. Y.; Pons-Martí, A.; Yang, Z.; Zhang, K. H. L.; de Leeuw, N. H.; Hensen, E. J. M.; Hofmann, J. P. Electronic Structure and Interface Energetics of CuBi2O4 Photoelectrodes. *J. Phys. Chem. C* **2020**, *124*, 22416–22425.
(76) Chen, S.; Liu, T.; Zheng, Z.; Ishaq, M.; Liang, G.; Fan, P.; Chen, T.; Tang, J. Recent Progress and Perspectives on Sb_2Se_3-Based Photocathodes for Solar Hydrogen Production via Photoelectrochemical Water Splitting. *J. Energy Chem.* **2022**, *67*, 508–523.
(77) E. Yourey, J.; M. Bartlett, B. Electrochemical Deposition and Photoelectrochemistry of CuWO4 , a Promising Photoanode for Water Oxidation. *J. Mater. Chem.* **2011**, *21*, 7651–7660.
(78) Chun, W.-J.; Ishikawa, A.; Fujisawa, H.; Takata, T.; Kondo, J. N.; Hara, M.; Kawai, M.; Matsumoto, Y.; Domen, K. Conduction and Valence Band Positions of Ta2O5, TaON, and Ta3N5 by UPS and Electrochemical Methods. *J. Phys. Chem. B* **2003**, *107*, 1798–1803.
(79) Lawley, C.; Nachtegaal, M.; Stahn, J.; Roddatis, V.; Döbeli, M.; Schmidt, T. J.; Pergolesi, D.; Lippert, T. Examining the Surface Evolution of $LaTiO_xN_y$ an Oxynitride Solar Water Splitting Photocatalyst. *Nat. Commun.* **2020**, *11*, 1728.
(80) Lawley, C.; Tehrani, Z. P.; Clark, A. H.; Safonova, O. V.; Döbeli, M.; Strocov, V. N.; Schmidt, T. J.; Lippert, T.; Nachtegaal, M.; Pergolesi, D. Protagonists and Spectators during

Photocatalytic Solar Water Splitting with $SrTaO_xN_y$ Oxynitride. *J. Mater. Chem. A* **2022**, *10*, 2374–2387.
(81) Septina, W.; Gunawan; Ikeda, S.; Harada, T.; Higashi, M.; Abe, R.; Matsumura, M. Photosplitting of Water from Wide-Gap Cu(In,Ga)S2 Thin Films Modified with a CdS Layer and Pt Nanoparticles for a High-Onset-Potential Photocathode. *J. Phys. Chem. C* **2015**, *119*, 8576–8583.
(82) Huang, S.; Luo, W.; Zou, Z. Band Positions and Photoelectrochemical Properties of Cu2ZnSnS4 Thin Films by the Ultrasonic Spray Pyrolysis Method. *J. Phys. Appl. Phys.* **2013**, *46*, 235108.
(83) Whittles, T. J.; Veal, T. D.; Savory, C. N.; Yates, P. J.; Murgatroyd, P. A. E.; Gibbon, J. T.; Birkett, M.; Potter, R. J.; Major, J. D.; Durose, K.; et al. Band Alignments, Band Gap, Core Levels, and Valence Band States in Cu_3BiS_3 for Photovoltaics. *ACS Appl. Mater. Interfaces* **2019**, *11*, 27033–27047.
(84) Wang, H.; Deutsch, T.; Turner, J. A. A. Direct Water Splitting Under Visible Light with a Nanostructured Photoanode and GaInP2 Photocathode. *ECS Trans.* **2008**, *6*, 37–44.
(85) Albero, J.; Peng, Y.; García, H. Photocatalytic CO_2 Reduction to C2+ Products. *ACS Catal.* **2020**, *10*, 5734–5749.
(86) Du, C.; Wang, X.; Chen, W.; Feng, S.; Wen, J.; Wu, Y. A. CO_2 Transformation to Multicarbon Products by Photocatalysis and Electrocatalysis. *Mater. Today Adv.* **2020**, *6*, 100071.
(87) Lu, H.; Wang, Z.; Wang, L. Photocatalytic and Photoelectrochemical Carbon Dioxide Reductions toward Value-Added Multicarbon Products. *ACS EST Eng.* **2022**, *2*, 975–988.
